# A rare case of Colistin-resistant *Salmonella* Enteritidis meningitis in an HIV-seropositive patient

**DOI:** 10.1186/s12879-019-4391-7

**Published:** 2019-09-14

**Authors:** Roxanne Rule, Nontombi Mbelle, John Osei Sekyere, Marleen Kock, Anwar Hoosen, Mohamed Said

**Affiliations:** 1 Department of Medical Microbiology, Pathology Building, University of Pretoria, Prinshof Campus, Corner of Steve Biko Road and Dr Savage Road, Pretoria, 0084 South Africa; 2 Tshwane Academic Division, National Health Laboratory Service, Corner of Steve Biko Road and Dr Savage Road, Pretoria, 0084 South Africa; 3 Vermaak and Partners Pathologists, Unitas Hospital, Corner of Rabie Street and Clifton Avenue, Lyttelton Manor, Pretoria, 0157 South Africa

**Keywords:** *Salmonella* Enteritidis, Non-typhoidal *Salmonella*, Meningitis, HIV, Colistin resistance

## Abstract

**Background:**

Non-typhoidal salmonellae (NTS) have been associated with invasive disease, notably meningitis, in immunocompromised individuals. Infections of this nature carry high rates of morbidity and mortality. Colistin resistance in salmonellae is a rare finding, more so in an invasive isolate such as cerebrospinal fluid (CSF). Colistin resistance has important infection control implications and failure to manage this phenomenon may lead to the loss of our last line of defence against multi-drug resistant Gram-negative organisms. To our knowledge, this is the first reported clinical case of colistin-resistant *Salmonella* Enteritidis meningitis in South Africa.

**Case presentation:**

We report a case of a young male patient with advanced human immunodeficiency virus (HIV) infection who presented to hospital with symptoms of meningitis. Cerebrospinal fluid (CSF) cultured a *Salmonella* Enteritidis strain. Antimicrobial susceptibility testing (AST) of the isolate, revealed the strain to be colistin resistant. Despite early and aggressive antimicrobial therapy, the patient succumbed to the illness after a short stay in hospital. Subsequent genomic analysis of the isolate showed no presence of the *mcr* genes or resistance-conferring mutations in *phoPQ, pmrAB, pmrHFIJKLME/arnBCADTEF, mgrB,* and *acrAB* genes, suggesting the presence of a novel colistin resistance mechanism.

**Conclusion:**

Invasive non-typhoidal salmonellae infection should be suspected in patients with advanced immunosuppression who present with clinical features of meningitis. Despite early and appropriate empiric therapy, these infections are commonly associated with adverse outcomes to the patient. Combination therapy with two active anti-*Salmonella* agents may be a consideration in the future to overcome the high mortality associated with NTS meningitis. Colistin resistance in clinical *Salmonella* isolates, although a rare finding at present, has significant public health and infection control implications. The causative mechanism of resistance should be sought in all cases.

**Electronic supplementary material:**

The online version of this article (10.1186/s12879-019-4391-7) contains supplementary material, which is available to authorized users.

## Background

Although infrequent, non-typhoidal salmonellae are important causes of bacterial meningitis in immunocompromised patients [1–3]. The emergence of non-typhoidal *Salmonella* meningitis in HIV infected individuals poses a significant therapeutic challenge. These infections are associated with high rates of morbidity and mortality, despite prompt and appropriate antimicrobial therapy [1–3]. This is especially true for patients who present with a Glascow coma scale (GCS) score of ≤13 [3]. Colistin resistance in human non-typhoidal salmonellae isolates is rare, but has been increasingly reported in animal strains, posing a potential zoonotic risk to humans [4]. In addition, the increasing emergence of colistin resistance threatens the future utility of this essential antimicrobial agent [5]. We report an interesting case of fatal meningitis caused by a colistin resistant *Salmonella* Enteritidis strain in a patient with advanced immunosuppression due to HIV infection.

## Case presentation

A 34-year-old male presented to the emergency department at a tertiary hospital in Pretoria, South Africa with a one-month history of headache, non-productive cough, fever, loss of weight and generalised body pain. The symptoms worsened over the preceding week, notably the fever and headache with associated neck pain and acute confusion. It was not known if the patient had a history of diarrhoea preceding presentation to hospital.

He was diagnosed with HIV infection approximately 2 years prior. His CD4 count was 2 cells/μL on admission and HIV viral load was 49,925 copies/mL 6 months earlier. His accompanying relative reported that he had been taking fixed dose combination antiretroviral therapy i.e., tenofovir, emtricitibine and efavirenz, but had defaulted treatment.

On examination, vital signs were all within normal limits. He had severe oral candidiasis and was confused with a Glasgow coma scale (GCS) of 12/15 with meningism. There were no focal neurological deficits and the rest of the clinical examination was unremarkable. Chest X-ray showed clear lung fields with no abnormalities. No further radiological testing was performed.

A full septic workup was done on admission. All results were within normal parameters, with the exception of the following outliers: (i) The C reactive protein (CRP) and erythrocyte sedimentation rate (ESR) values were elevated at 130 mg/L and 125 mm/hr. respectively, suggesting an inflammatory process, (ii) Pre-renal impairment was evident by an elevated urea of 13.5 mmol/L and normal creatinine, (iii) Full blood count revealed a normal white cell count, but neutrophilia on the differential count of 8.49 × 10^9^/L. (iv) The cerebrospinal fluid was grossly purulent, with numerous gram negative bacilli and inflammatory cells on the gram stain. Biochemistry on the CSF revealed an elevated protein (9.90 g/L), reduced glucose (0.1 mmol/L) and markedly elevated adenosine deaminase (ADA) of > 120 IU/L. Gene Xpert MTB/Rif Ultra (Cepheid, Sunnyvale, CA, USA) on the CSF was negative.

Based on the above clinical and laboratory findings, the patient was diagnosed with acute bacterial meningitis. The exact causative agent was still to be determined. Empiric antibiotic therapy with intravenous ceftriaxone at a dose of 2 g 12 hourly was administered to cover for the common causes of bacterial meningitis. Intravenous corticosteroid therapy with dexamethasone at a dose of 8 mg 8 hourly was added to reduce intracranial inflammation and prevent long term neurological sequelae.

The following day, based on the elevated ADA and ESR results (despite a negative GeneXpert MTB/Rif Ultra result), oral first line anti-tuberculosis therapy was initiated for possible concomitant tuberculous meningitis. A combination tablet to be taken daily containing rifampicin 600 mg, isoniazid 300 mg, pyrazinamide 1600 mg and ethambutol 1100 mg was given. The patient was transferred to an isolation room with airborne precautions instituted. Antiretroviral treatment (ART) was not re-initiated for reasons unknown to us.

On day three, his condition deteriorated, with a decrease in GCS to 10/15. Antibiotic therapy was escalated to intravenous meropenem at a dose of 2 g 8 hourly to cover for a possible extended spectrum β-lactamase producing Gram-negative bacillary meningitis. The patient was kept on intravenous ceftriaxone despite advice from microbiology to escalate the patient to meropenem only. The CSF grew a non-lactose fermenter with colony morphology in keeping with a *Salmonella* species. The colonies were presumptively identified as *Salmonella* Group D with a latex agglutination assay (Wellcolex Colour *Salmonella* Agglutination (Remel, London, UK)). These findings were promptly communicated to the treating clinicians. Blood cultures taken on admission failed to yield any significant growth.

On day four of admission, the Vitek 2 (bioMérieux, France) automated system confirmed the CSF isolate to be *Salmonella* group. Serotyping was performed according to the White Kauffman Le Minor scheme, which revealed the organism to be a *Salmonella enterica* serovar Enteritidis strain. Susceptibility to ceftriaxone and ciprofloxacin, identified on the Vitek 2 system was confirmed with E-tests (bioMérieux, France), with MICs of 0.064 μg/mL and 0.023 μg/mL, respectively. The isolate was susceptible to meropenem on the Vitek 2 system, with an MIC of ≤0.25 μg/mL. Of note, the organism showed resistance to colistin on Vitek 2 which was confirmed by broth micro dilution with an MIC of 4 μg/ml. A stool specimen was not submitted for investigation of potential colistin-resistant Gram-negative enteric organisms which may have served as the source for colistin resistance through plasmid mediated transfer of *mcr* genes. The lack of active management from the side of the clinicians prevented further samples from reaching the laboratory.

Following release of culture and AST results, meropenem was stopped and ceftriaxone was continued. Unfortunately, despite appropriate antimicrobial treatment, the patient succumbed to his illness and demised on day six of admission. The outcome of this patient further supports the evidence found by Keddy et al. (2015) that a GCS of ≤13 on presentation to hospital is a strong predicter of mortality in patients with non-typhoidal *Salmonella* meningitis [3].

**Fig. 1:**
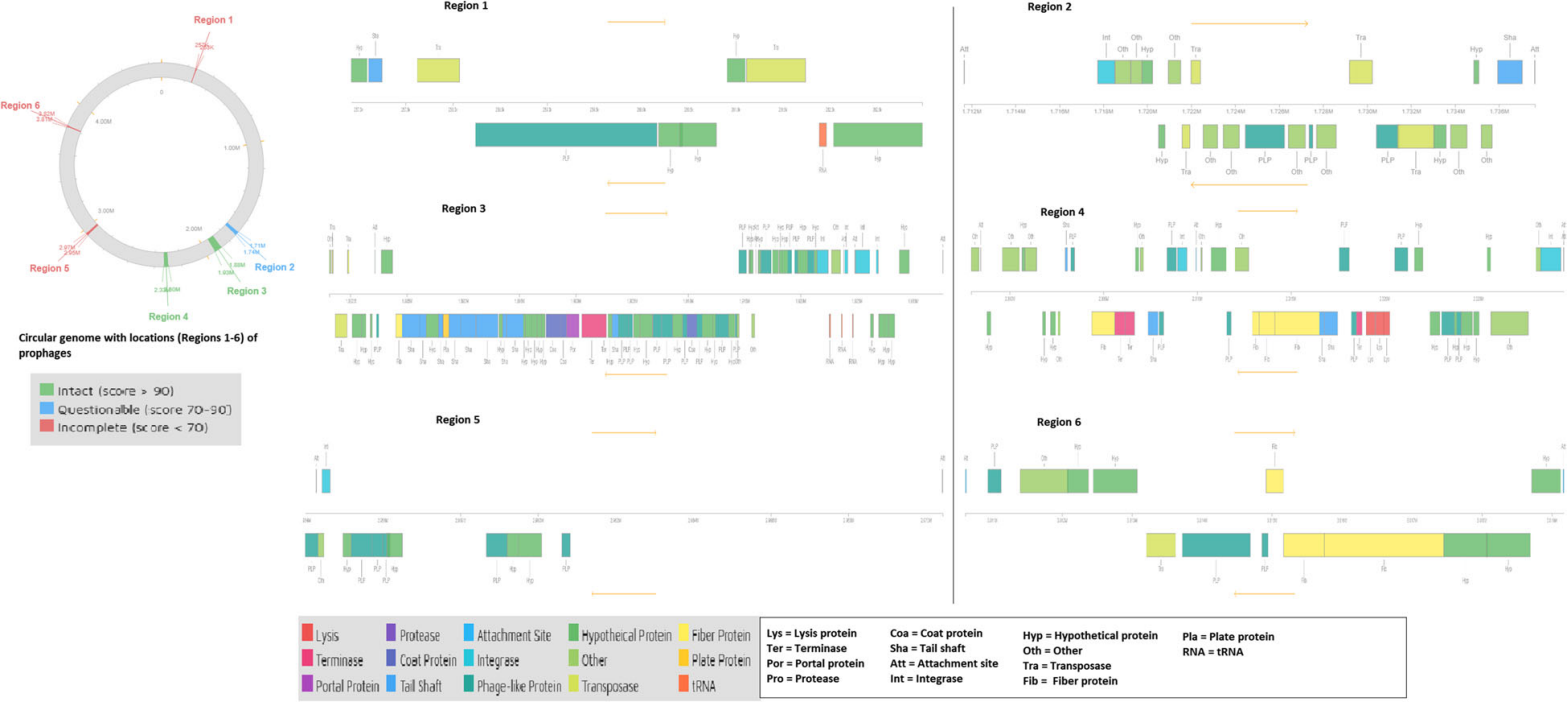
Prophages found in *S.* Enteritidis strain EC20120916. Six prophages were found in the strain, with only two being intact. Prophages are biomarker signatures that can be used to identify specific clones and strains of Salmonellae. Prophages reportedly found in African strains were found in this strain

An in-house PCR was performed on the isolate, but it was negative for the *mcr-1* gene. Whole genome sequencing (WGS) was performed with the Illumina MiSeq platform. No *mcr* (*mcr1–8*) or known colistin resistance-conferring mutations were found in the *pmrAB, pmrHFIJKLMD, arnE, arnC, phoPQ, mgrB* and *acrAB* genes (Additional file 1). Based on these findings, we can conclude that a novel mechanism is responsible for the colistin resistance in this *Salmonella* Enteritidis isolate. A MLST (multilocus sequence typing) analysis of the genome using MLST 2.0 (https://cge.cbs.dtu.dk/services/MLST/) showed that it was of ST11. The isolate was confirmed as a human pathogen by PathogenFinder [6], and no SNPs (single nucleotide polymorphism) or deletion of genes were found in the pathogenic/virulence gene repertoire (Additional file 1). The isolate contained five of the 12 known Salmonellae pathogenicity islands viz., *C63PI*, *SPI-5, SPI-13, SPI-3, SPI-14,* and two plasmid replicons i.e., IncFIB(S) and IncFII(S), suggesting the presence of at least one IncF-type plasmid (Additional file 1)*.* Six prophages were also found in the genome of the isolate, including Fels-2-like prophage genes (Fig. 1).

**Fig. 2:**
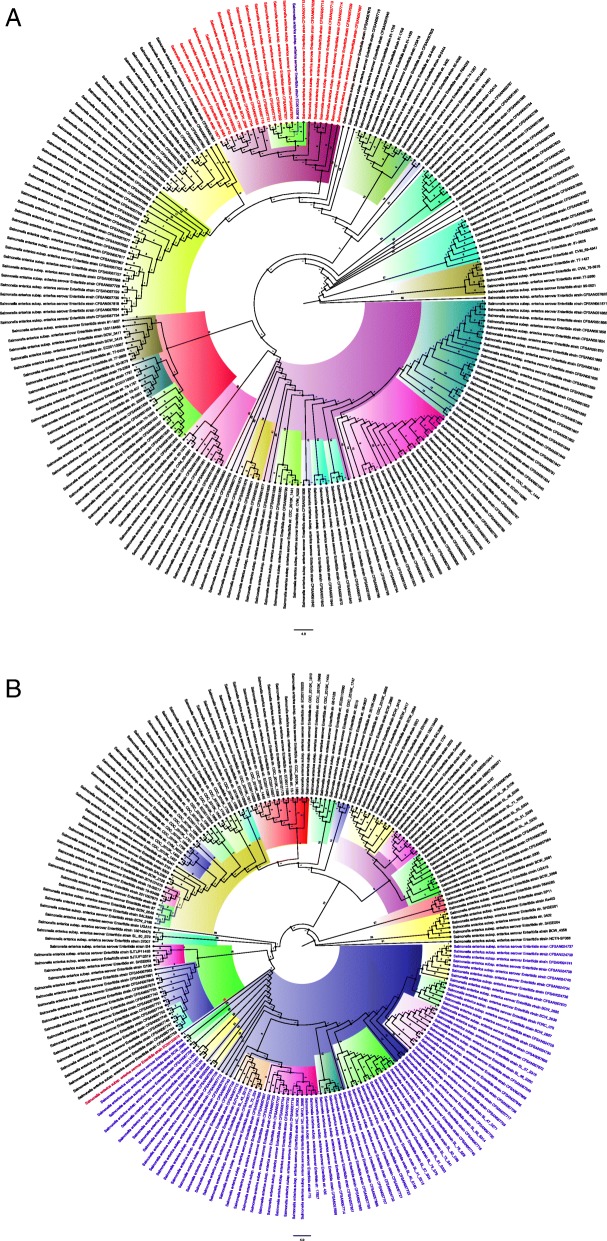
Phylogenetic tree of *S*. Enteritidis strains obtained worldwide. **a** Relationship between strain EC20120916 and *S*. Enteritidis strains of international origin isolated between 2005 and 2019. **b** Relationship between strain EC20120916 and *S*. Enteritidis strains of international origin isolated between 1953 and 2005. There was greater relationship between the strain EC20120916 and recent strains isolated between 2005 and 2019 than between those isolated from 1953 to 2005, showing that this strain has recently evolved. It’s closeness to Brazilian and USA strains of human origin suggests that is of the international epidemic clade and anthroponotic

A phylogenetic analysis of the isolate, with 398 other *S.* Enteritidis isolates obtained from NCBI/Patric (https://www.patricbrc.org/) that were isolated between 1953 and 2019 from humans, poultry, cattle, mice etc. in various countries and continents, was undertaken. The results are shown in Fig. 2.

The assembled and annotated genome sequence of this *S.* Enteritidis EC20120916 strain is available at Genbank under accession number SHPL00000000 (PRJNA PRJNA521953).

## Discussion and conclusions

The above patient was managed aggressively with a multidisciplinary approach, including input from internal medicine physicians and microbiologists. Laboratory results were communicated timeously to the treating clinicians and antibiotics were adjusted accordingly. A limitation to the approach, however, was the inability to provide a rapid microbiological result to the treating clinicians. Due to financial constraints in the public sector in South Africa, multiplex polymerase chain reaction (PCR) assays are not incorporated into routine diagnostic care. As a result, conventional culture was performed on this patients’ CSF, increasing the turnaround time to 4 days before final identification and susceptibility results were made available to the treating clinicians. This limitation, however, did not significantly impact on patient care, as the initial empiric antibiotic regimen that the patient was started on was found to be appropriate with subsequent antimicrobial susceptibility testing of the isolate.

The phylogenetic analyses shown in Fig. 2 show that the isolate was more anthroponotic than zoonotic as it was closely related to human strains obtained from Brazil and the USA [7], implicating a potential importation of the strain into South Africa (Additional file 2 and Additional file 3). Using the classification suggested by Feasey et al. (2016) [7], it is obvious that this isolate belongs to the global epidemic clade as no African isolate was found within its immediate phylogenetic environment. Moreover, SNPs or degenerations in pathogenic/virulence genes, as observed by Feasey et al. (2016) [7] in sub-Saharan African isolates, were not observed in this isolate. In addition, prophage ɸSE20, which is known to be essential for invasion of chicken and mice [7], was not found in this isolate while Fels-2-like prophage, which was associated with African clades [7], was present. These further suggest that the isolate was very anthroponotic.

In the immunocompromised host, NTS are able to overcome the crippled immune system and invade various organ systems, in particular, the central nervous system leading to meningitis [1, 2]. This condition is associated with a high morbidity and mortality [1, 3, 7]. More than 95% of invasive NTS infections in Africa have been found in patients with HIV infection, supporting the notion that an immunocompromised state is a key risk factor for developing invasive NTS infection [2].

A recent study conducted by Keddy et al. (2015) in South Africa, described a series of *Salmonella* meningitis over a 10-year period. In concordance with other literature [1, 2], a strong association of NTS meningitis and HIV infection was noted, with 76.1% of NTS meningitis cases having HIV co-infection [3]. HIV co-infection was found to be a major contributor for mortality, with a mortality rate of 42.6% in HIV infected individuals compared to 13.6% in HIV uninfected individuals with NTS meningitis. Moreover, a GCS score of ≤13 on presentation was found to be a strong predictor of mortality, with a mortality rate of 80% in this study [3]. Although access to antiretroviral therapy has been proven by Keddy et al. (2017) to be associated with a significant reduction in the incidence of invasive NTS infections caused by *S.* Typhimurium, the same trend has not been observed for *S.* Enteritidis in Gauteng, South Africa. As suggested by Keddy et al. a postulated reason for this may be the anthroponotic adaptation of some *S.* Enteritidis African strains and the lack of population immunity to these strains [8].

The increased risk of NTS invasion among advanced HIV-infected patients is postulated to be due to three important mechanisms: (i) loss of gastrointestinal mucosal integrity due to reduced levels of IL-17-producing CD4 T-cells, leading to reduced expression of antimicrobial peptides as well as failure to recruit neutrophils to the site of infection in the mucosa; (ii) the organism is able to persist in its intracellular niche as a result of dysregulated cytokine responses known to occur in HIV-infected individuals, predisposing these patients to frequent reactivation and relapses of bacteraemia and (iii) an excess production of immunoglobulin G towards the *Salmonella* lipopolysaccharide inhibits effective serum killing by blocking bactericidal antibodies that target the organism’s outer membrane proteins [1, 2].

Limited data exist on the association between elevated ADA and *Salmonella* infections. Ketavarapu et al. (2013), reported that serum ADA levels are elevated in patients presenting with typhoid fever as opposed to those presenting with fever due to other illnesses [9]. In this case, the CSF ADA was markedly elevated with a negative GeneXpert MTB/Rif Ultra result. The extent of ADA elevation due to *Salmonella* meningitis needs to be investigated further.

Empiric therapy of life threatening non-typhoidal *Salmonella* infections is a third generation cephalosporin or a fluoroquinolone [10]. Patients with meningitis should be managed with a parenteral cephalosporin (once susceptibilities are confirmed) for a minimum duration of 3 weeks [10, 11]. In this case, the patient was escalated to meropenem, while awaiting AST, to cover for an extended spectrum beta-lactamase (ESBL) producing strain, in light of the clinical deterioration whilst on the empiric third generation cephalosporin. The emergence of ESBL producing strains is of serious concern and an increase in the incidence of ESBL producing *Salmonella* isolates has been noticed at the Tshwane Academic Laboratory.

Colistin is a repurposed antibiotic largely reserved as an agent of last-resort for multi-drug resistant Gram-negative pathogens [5]. It’s role in infections caused by these organisms is under siege due to various mechanisms, including chromosomal mutations as well as the increasing threat of transmissible mobile genetic elements carrying resistance conferring genes [5].

Aside from its role in human medicine, colistin is also frequently used in the veterinary field for the treatment of gastrointestinal infections in animals [5]. Possession of the *mcr-1* plasmid-mediated colistin resistance gene has been detected in diverse strains of *Salmonella* species, including human and animal strains, suggesting a possible zoonotic risk [4].

To our knowledge, this is the first reported case of colistin-resistant *Salmonella* Enteritidis in South Africa. Enterobacteriaceae employ various mechanisms to confer resistance to colistin [12], including mutations in the *pmrHFIJKLM* operon, which are responsible for the biosynthesis of lipid A [12]. Of note, mutations in the *pmrA* and/or *pmrB* genes [13], as well as *pmrL* and *pmrM* genes [12] have been shown to confer resistance to colistin in *Salmonella* Typhimurium isolates. The *AcrAB* efflux pumps have also proven to confer resistance to colistin [12]. Detailed comparative genomic analyses of the isolate showed no mutations in any of these genes.

Plasmid-mediated resistance is of increasing concern. To date, nine variants of the *mcr* gene have been described [14]. These genes confer resistance by reducing the anionic charges of lipid A, thereby decreasing its binding affinity to colistin [12]. A risk factor for developing colistin resistance could not be determined in our patient. No *mcr* gene was present in our isolate.

The absence of colistin-resistance conferring mutations and plasmid-mediated colistin resistance genes in our isolate suggests the presence of a novel colistin resistance mechanism.

This case proves that NTS meningitis is a serious and emerging complication of *Salmonella* infection in HIV-infected individuals and is associated with a poor prognosis despite appropriate therapy. Combination therapy with ceftriaxone and a fluoroquinolone may be a possible therapeutic regimen for future cases [11], in an attempt to improve patient outcomes. Patients with HIV infection should be promptly initiated on ART, educated on the importance of compliance to ART and monitored closely. This strategy may drastically reduce the incidence of invasive NTS infections in this vulnerable population group. Failure in this regard will result in severely hampering the UNAIDS 2020 targets of 90% rate of ART coverage in HIV-diagnosed individuals and a 90% rate of virological suppression in patients on ART [15].

The emergence of colistin resistance is of serious concern and the underlying mechanisms attributable to this resistance should be investigated in all colistin resistant salmonellae isolates in order to optimise infection control practices and curb the spread of plasmid-mediated *mcr* genes. In addition, a One Health-based approach should be implemented to further preserve the efficacy of this critical antimicrobial agent.

## Additional files

Additional file 1: Annotation results of *S.* Enteritidis strain EC20120916 genome. MLST, pathogenicity island, plasmid typing, resistance gene and pathogenicity of the strain determined to be ST11, five (*C63PI, SPI-5, SPI-13, SPI-3, SPI-14*), IncFII(S) and IncFIB(S), 0.94, and *aac (6′)-Iaa* respectively*. (DOCX 460 kb)*

Additional file 2: Metadata of isolates used for phylogenetic tree Fig. 2A (XLSX 545 kb)

Additional file 3: Metadata of isolates used for phylogenetic tree Fig. 2B (XLSX 556 kb)

## Data Availability

The datasets generated and/or analysed during the current study are available at Genbank under accession number SHPL00000000 (PRJNA PRJNA521953).

## Abbreviations

ADA: Adenosine deaminase
ART: Antiretroviral therapy
AST: Antimicrobial susceptibility
CRP: C-reactive protein
CSF: Cerebrospinal fluid
ESBL: Extended spectrum beta-lactamase
ESR: Erythrocyte sedimentation rate
GCS: Glasgow coma scale
HIV: Human Immunodeficiency Virus
MALDI-TOF MS: Matrix-assisted laser desorption/ionisation – time of flight mass spectrometry
MIC: Minimum inhibitory concentration
MLST: Multilocus sequence typing
NTS: Non-typhoidal *Salmonellae*
SNP: Single nucleotide polymorphism
WGS: Whole genome sequencing
