# Supplementary material for: A rare case of Colistin-resistant Salmonella Enteritidis meningitis in an HIV-seropositive patient
Source: BMC Infect Dis. 2019 Sep 14;19:806. doi: 10.1186/s12879-019-4391-7 (PMC6744686; doi:10.1186/s12879-019-4391-7)
Supplement: Supplementary file 1 — Annotation results of S. Enteritidis strain EC20120916 genome. MLST, pathogenicity island, plasmid typing, resistance gene and pathogenicity of the strain determined to be ST11, five (C63PI, SPI-5, SPI-13, SPI-3, SPI-14), IncFII(S) and IncFIB(S), 0.94, and aac (6′)-Iaa respectively. (DOCX 460 kb) [file 12879_2019_4391_MOESM1_ESM.docx]

**Dataset**


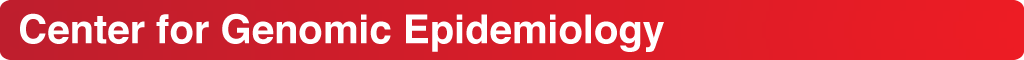


**MLST-2.0 Server - Results**

**mlst Profile:  *senterica***

**Organism:  *Salmonella enterica***

**Sequence Type:  *11***

| **Locus** | **Identity** | **Coverage** | **Alignment Length** | **Allele Length** | **Gaps** | **Allele** |
| --- | --- | --- | --- | --- | --- | --- |
| aroC | 100 | 100 | 501 | 501 | 0 | aroC_5 |
| dnaN | 100 | 100 | 501 | 501 | 0 | dnaN_2 |
| hemD | 100 | 100 | 432 | 432 | 0 | hemD_3 |
| hisD | 100 | 100 | 501 | 501 | 0 | hisD_7 |
| purE | 100 | 100 | 399 | 399 | 0 | purE_6 |
| sucA | 100 | 100 | 501 | 501 | 0 | sucA_6 |
| thrA | 100 | 100 | 501 | 501 | 0 | thrA_11 |

extended output

**Input Files: *181210_RH01221661_contigs.fa***


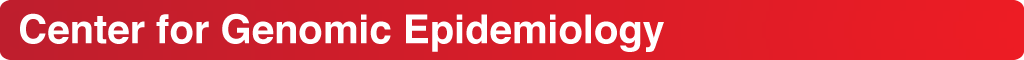


**PlasmidFinder-2.0 Server - Results**

**Organism(s):  *Enterobacteriaceae***

| **Enterobacteriaceae** | | | | | | |
| --- | --- | --- | --- | --- | --- | --- |
| **Plasmid** | **Identity** | **Query / Template length** | **Contig** | **Position in contig** | **Note** | **Accession number** |
| IncFIB(S) | 98.91 | 643 / 643 | SM-Roxy-RH01221661_S26_L001_R1_001_(paired)_contig_16 | 879..1521 |  | [FN432031](http://www.ncbi.nlm.nih.gov/nuccore/FN432031) |
| IncFII(S) | 100 | 262 / 262 | SM-Roxy-RH01221661_S26_L001_R1_001_(paired)_contig_56 | 1927..2188 |  | [CP000858](http://www.ncbi.nlm.nih.gov/nuccore/CP000858) |

***IncFIB(S)_FN432031***

template GGACAAGGACAATCTGGACATAAAAAAGTTGTTTGTAGAGGTGGATAAATCCTCCGGTGA

query GGACAAGGACAATCTGGACATAAAAAAGTTGTTTGAAGAGGTGGATAAATCCTCCGGTGA

template AATCGTAAATCTCACTCCTAATGCCAGTAATACCGTGCAGCCTGTAGCTCTGATGCGGCT

query AATCGTAAATCTCACTCCTAATGCCAGTAATACCGTGCAGCCTGTAGCTCTGATGCGGCT

template TGGAGTATTTGTTCCTACGCTTAAGTCACTGAAAAACCGTAAAAAGAACACGCTTTCGCG

query TGGAGTATTTGTTCCTACGCTTAAGTCACTGAAAAACCGTAAAAAGAACACACTTTCGCG

template TACTGATGCGTCGGAAGAACTGACGCGTCTGTCTCTCGCCAGGGCCGAGGGGTTTGATAA

query TACTGATGCGTCGGAAGAACTGACGCGTCTGTCTCTCGCCAGGGCCGAGGGGTTTGATAA

template AGTGGAGATCACTGGTCCCCGCCTGGATATGGATAATGACTTCAAGACCTGGGTAGGGGT

query AGTGGAGATCACCGGTCCCCGCCTGGATATGGATAATGACTTCAAGACCTGGGTAGGGGT

template GATTCATTCCTTTGCCCGGCATAAAGTTATTGGCGATAAAGTTGAGCTGTCGTTTGTCGA

query GATCCATTCCTTTGCCCGACATAAAGTTATTGGCGATAAAGTTGAGCTGCCGTTTGTCGA

template ATTCGCCAAACTCTGTGGGATCCCCTCCAGTCAGTCATCCCGGAAGCTCCGGGAACGTAT

query ATTCGCCAAACTCTGTGGGATCCCCTCCAGTCAGTCATCCCGGAAGCTCCGGGAACGTAT

template CAGTCCTTCACTTAAGCGTATCGCGGGTACTGTGATCTCGTTTTCCCGCACAACGGAGAA

query CAGTCCTTCACTTAAGCGTATCGCGGGTACTGTGATCTCGTTTTCCCGCACAACGGAGAA

template GCACACCAAAGAGTACATTACGCATCTGGTACAGTCGGCTTACTATGATACGGAAAAAGA

query GCACACCAAAGAGTACATTACGCATCTGGTACAGTCGGCTTACTATGATACGGAAAAAGA

template TATCGTTCAGCTGCAAGCCGATCCGCGCCTCTTTGAACTCTACCAGTTTGACCGGAAAGT

query TATCGTTCAACTGCAAGCCGATCCGCGCCTCTTTGAACTCTACCAGTTTGACCGGAAAGT

template GCTGCTGCAGCTCAAGGCCATCAATGCCCTGAAGCGCCGGGAG

query GCTGCTGCAGCTCAAGGCCATCAATGCCCTGAAGCGCCGGGAG

***IncFII(S)_CP000858***

template CTAAAGAATTTTGATGGCTGGCCACGCCGTAAGGTGGCAGGGAACTGGTTCTGATGAGGT

query CTAAAGAATTTTGATGGCTGGCCACGCCGTAAGGTGGCAGGGAACTGGTTCTGATGAGGT

template GTCTACCCGGGACCAGAAAAGCAAAAACCCCGATAATCTTCTCATTTCTTGGCGGGAACG

query GTCTACCCGGGACCAGAAAAGCAAAAACCCCGATAATCTTCTCATTTCTTGGCGGGAACG

template AAAGATTAACGGGGCCTACTTAAACTGTATAGCCACCAATCAGGCTATGCAGGGAGTATA

query AAAGATTAACGGGGCCTACTTAAACTGTATAGCCACCAATCAGGCTATGCAGGGAGTATA

template GTTTTATGCTCAGAAAATTTCAATACTTGTTTCTGTGGCATTTACTCCTTCCGTGCATTG

query GTTTTATGCTCAGAAAATTTCAATACTTGTTTCTGTGGCATTTACTCCTTCCGTGCATTG

template TAAGTGCAGGCAGAAGTGACTG

query TAAGTGCAGGCAGAAGTGACTG

**Input Files: *181210_RH01221661_contigs.fa***

Top of Form

Bottom of Form


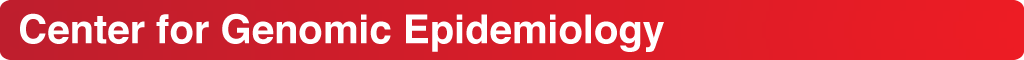


**ResFinder-3.1 Server - Results**

**Input Files: *181210_RH01221661_contigs.fa***

Show Acquired antimicrobial resistance results

**Acquired antimicrobial resistance gene - Results**

| **Aminoglycoside** | | | | | | |
| --- | --- | --- | --- | --- | --- | --- |
| **Resistance gene** | **Identity** | **Query/HSP** | **Contig** | **Position in contig** | **Phenotype** | **Accession no.** |
| aac(6')-Iaa | 96.35 | 438/438 | SM-Roxy-RH01221661_S26_L001_R1_001_(paired)_contig_25 | 219409..219846 | Aminoglycoside resistance | [NC_003197](http://www.ncbi.nlm.nih.gov/nuccore/NC_003197) |

| **Beta-lactam** | | | | | | |
| --- | --- | --- | --- | --- | --- | --- |
| No resistance genes found. |  |  |  |  |  |  |

| **Colistin** | | | | | | |
| --- | --- | --- | --- | --- | --- | --- |
| No resistance genes found. |  |  |  |  |  |  |

| **Fluoroquinolone** | | | | | | |
| --- | --- | --- | --- | --- | --- | --- |
| No resistance genes found. |  |  |  |  |  |  |

| **Fosfomycin** | | | | | | |
| --- | --- | --- | --- | --- | --- | --- |
| No resistance genes found. |  |  |  |  |  |  |

| **Fusidic Acid** | | | | | | |
| --- | --- | --- | --- | --- | --- | --- |
| No resistance genes found. |  |  |  |  |  |  |

| **Glycopeptide** | | | | | | |
| --- | --- | --- | --- | --- | --- | --- |
| No resistance genes found. |  |  |  |  |  |  |

| **MLS - Macrolide, Lincosamide and Streptogramin B** | | | | | | |
| --- | --- | --- | --- | --- | --- | --- |
| No resistance genes found. |  |  |  |  |  |  |

| **Nitroimidazole** | | | | | | |
| --- | --- | --- | --- | --- | --- | --- |
| No resistance genes found. |  |  |  |  |  |  |

| **Oxazolidinone** | | | | | | |
| --- | --- | --- | --- | --- | --- | --- |
| No resistance genes found. |  |  |  |  |  |  |

| **Phenicol** | | | | | | |
| --- | --- | --- | --- | --- | --- | --- |
| No resistance genes found. |  |  |  |  |  |  |

| **Rifampicin** | | | | | | |
| --- | --- | --- | --- | --- | --- | --- |
| No resistance genes found. |  |  |  |  |  |  |

| **Sulphonamide** | | | | | | |
| --- | --- | --- | --- | --- | --- | --- |
| No resistance genes found. |  |  |  |  |  |  |

| **Tetracycline** | | | | | | |
| --- | --- | --- | --- | --- | --- | --- |
| No resistance genes found. |  |  |  |  |  |  |

| **Trimethoprim** | | | | | | |
| --- | --- | --- | --- | --- | --- | --- |
| No resistance genes found. |  |  |  |  |  |  |

**Selected %ID threshold:  *90 %***

**Selected minimum length:  *60 %***

Top of Form

**Chromosomal point mutations - Results**

**Species:*salmonella***

**Known Mutations**

| **gyrA** | | | | |
| --- | --- | --- | --- | --- |
| No mutations found in gyrA |  |  |  |  |

| **parC** | | | | |
| --- | --- | --- | --- | --- |
| No mutations found in parC |  |  |  |  |

| **parE** | | | | |
| --- | --- | --- | --- | --- |
| No mutations found in parE |  |  |  |  |

| **pmrB** | | | | |
| --- | --- | --- | --- | --- |
| No mutations found in pmrB |  |  |  |  |

| **gyrB** | | | | |
| --- | --- | --- | --- | --- |
| No mutations found in gyrB |  |  |  |  |

| **23S** | | | | |
| --- | --- | --- | --- | --- |
| No known mutations found in 23S |  |  |  |  |

| **pmrA** | | | | |
| --- | --- | --- | --- | --- |
| No mutations found in pmrA |  |  |  |  |

| **16S_rrsD** | | | | |
| --- | --- | --- | --- | --- |
| No known mutations found in 16S_rrsD |  |  |  |  |

**Unknown Mutations**

| **23S** | | | | |
| --- | --- | --- | --- | --- |
| **Mutation** | **Nucleotide change** |  |  |  |
| 23S r.56A>G | A ➝ G |  |  |  |
| 23S r.78T>C | T ➝ C |  |  |  |
| 23S r.113T>A | T ➝ A |  |  |  |
| 23S r.114T>C | T ➝ C |  |  |  |
| 23S r.137T>A | T ➝ A |  |  |  |
| 23S r.142A>T | A ➝ T |  |  |  |
| 23S r.264C>G | C ➝ G |  |  |  |
| 23S r.284T>C | T ➝ C |  |  |  |
| 23S r.285G>A | G ➝ A |  |  |  |
| 23S r.348A>G | A ➝ G |  |  |  |
| 23S r.349T>C | T ➝ C |  |  |  |
| 23S r.353C>T | C ➝ T |  |  |  |
| 23S r.354A>G | A ➝ G |  |  |  |
| 23S r.766T>C | T ➝ C |  |  |  |
| 23S r.877A>T | A ➝ T |  |  |  |
| 23S r.884T>C | T ➝ C |  |  |  |
| 23S r.892A>G | A ➝ G |  |  |  |
| 23S r.1171G>A | G ➝ A |  |  |  |
| 23S r.1173delT | del ➝ T |  |  |  |
| 23S r.1175A>G | A ➝ G |  |  |  |
| 23S r.1178C>T | C ➝ T |  |  |  |
| 23S r.1219T>G | T ➝ G |  |  |  |
| 23S r.1220G>C | G ➝ C |  |  |  |
| 23S r.1229C>G | C ➝ G |  |  |  |
| 23S r.1230A>T | A ➝ T |  |  |  |
| 23S r.1387A>G | A ➝ G |  |  |  |
| 23S r.1400T>C | T ➝ C |  |  |  |
| 23S r.1480delC | del ➝ C |  |  |  |
| 23S r.1482_1483insT | ins ➝ T |  |  |  |
| 23S r.1485T>G | T ➝ G |  |  |  |
| 23S r.1493C>T | C ➝ T |  |  |  |
| 23S r.1501_1502insTT | ins ➝ TT |  |  |  |
| 23S r.1502A>C | A ➝ C |  |  |  |
| 23S r.1504A>C | A ➝ C |  |  |  |
| 23S r.1505A>T | A ➝ T |  |  |  |
| 23S r.1507C>T | C ➝ T |  |  |  |
| 23S r.1510G>C | G ➝ C |  |  |  |
| 23S r.1511G>A | G ➝ A |  |  |  |
| 23S r.1523T>C | T ➝ C |  |  |  |
| 23S r.1712T>C | T ➝ C |  |  |  |
| 23S r.1714T>C | T ➝ C |  |  |  |
| 23S r.1723G>A | G ➝ A |  |  |  |
| 23S r.1726C>G | C ➝ G |  |  |  |
| 23S r.1727C>A | C ➝ A |  |  |  |
| 23S r.1728C>T | C ➝ T |  |  |  |
| 23S r.1730C>T | C ➝ T |  |  |  |
| 23S r.1731G>A | G ➝ A |  |  |  |
| 23S r.1733G>T | G ➝ T |  |  |  |
| 23S r.1734G>C | G ➝ C |  |  |  |
| 23S r.1746A>G | A ➝ G |  |  |  |
| 23S r.1865T>C | T ➝ C |  |  |  |
| 23S r.1882T>C | T ➝ C |  |  |  |
| 23S r.2203T>G | T ➝ G |  |  |  |
| 23S r.2211A>T | A ➝ T |  |  |  |
| 23S r.2213T>C | T ➝ C |  |  |  |
| 23S r.2793C>G | C ➝ G |  |  |  |
| 23S r.2794C>A | C ➝ A |  |  |  |
| 23S r.2798T>A | T ➝ A |  |  |  |
| 23S r.2799A>G | A ➝ G |  |  |  |
| 23S r.2802G>T | G ➝ T |  |  |  |
| 23S r.2803G>C | G ➝ C |  |  |  |

| **16S_rrsD** | | | | |
| --- | --- | --- | --- | --- |
| **Mutation** | **Nucleotide change** |  |  |  |
| 16S_rrsD r.4G>T | G ➝ T |  |  |  |
| 16S_rrsD r.37G>T | G ➝ T |  |  |  |
| 16S_rrsD r.40G>T | G ➝ T |  |  |  |
| 16S_rrsD r.1004C>G | C ➝ G |  |  |  |
| 16S_rrsD r.1005T>A | T ➝ A |  |  |  |
| 16S_rrsD r.1006T>A | T ➝ A |  |  |  |
| 16S_rrsD r.1021G>T | G ➝ T |  |  |  |
| 16S_rrsD r.1132A>G | A ➝ G |  |  |  |
| 16S_rrsD r.1138T>C | T ➝ C |  |  |  |

Top of Form

Bottom of Form


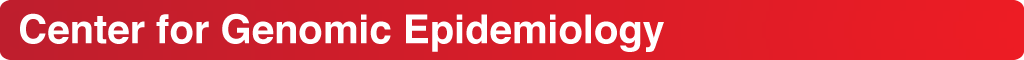


# SPIFinder-1.0 Server - Results

| **Pathogenic islands** | | | | | | | | | |
| --- | --- | --- | --- | --- | --- | --- | --- | --- | --- |
| **Gene** | **Origin** | **%Identity** | **HSP/Query length** | **Contig** | **Position in contig** | **Insertion location** | **Function category** | **Genome Accession** | **SPI Accession** |
| *C63PI* | Salmonella Typhimurium SL1344 | 99.12 | 4000 / 4000 | SM-Roxy-RH01221661_S26_L001_R1_001_(paired)_contig_11 | 346335..350334 | fhlA | 1 |  | [AF128999](http://www.ncbi.nlm.nih.gov/nuccore/AF128999) |
| *SPI-5* | Salmonella Typhimurium LT2 | 99.08 | 9069 / 9069 | SM-Roxy-RH01221661_S26_L001_R1_001_(paired)_contig_42 | 50646..59710 | tRNA-serT | 18 | [NC_003197](http://www.ncbi.nlm.nih.gov/nuccore/NC_003197) | [gi|16763390:1175321-1184389](http://www.ncbi.nlm.nih.gov/nuccore/16763390/?from=1175321&to=1184389) |
| *SPI-13* | Salmonella Gallinarum SGD-3 | 99.41 | 338 / 338 | SM-Roxy-RH01221661_S26_L001_R1_001_(paired)_contig_11 | 73088..73425 | tRNA-pheV | 9 |  | [AY956832](http://www.ncbi.nlm.nih.gov/nuccore/AY956832) |
| *SPI-13* | Salmonella Gallinarum SGG-1 | 99.75 | 404 / 404 | SM-Roxy-RH01221661_S26_L001_R1_001_(paired)_contig_11 | 73733..74136 | tRNA-pheV | 11 |  | [AY956833](http://www.ncbi.nlm.nih.gov/nuccore/AY956833) |
| *SPI-13* | Salmonella Gallinarum SGA-10 | 100.00 | 341 / 341 | SM-Roxy-RH01221661_S26_L001_R1_001_(paired)_contig_11 | 75504..75844 | tRNA-pheV | 10 |  | [AY956834](http://www.ncbi.nlm.nih.gov/nuccore/AY956834) |
| *SPI-3* | Salmonella Typhimurium LT2 | 98.73 | 12102 / 16616 | SM-Roxy-RH01221661_S26_L001_R1_001_(paired)_contig_15 | 873..12969 | tRNA-selC | 15 | [NC_003197](http://www.ncbi.nlm.nih.gov/nuccore/NC_003197) | [gi|16763390:3948576-3965191](http://www.ncbi.nlm.nih.gov/nuccore/16763390/?from=3948576&to=3965191) |
| *SPI-14* | Salmonella Gallinarum SGA-8 | 99.80 | 501 / 501 | SM-Roxy-RH01221661_S26_L001_R1_001_(paired)_contig_38 | 93557..94057 | Not_published | 12 |  | [AY956835](http://www.ncbi.nlm.nih.gov/nuccore/AY956835) |
| *SPI-14* | Salmonella Gallinarum SGC-8 | 99.55 | 441 / 441 | SM-Roxy-RH01221661_S26_L001_R1_001_(paired)_contig_38 | 99138..99578 | Not_published | 13 |  | [AY956836](http://www.ncbi.nlm.nih.gov/nuccore/AY956836) |


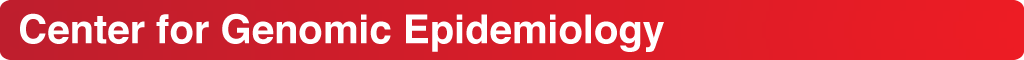


**The input organism was predicted as human pathogen**

| **Probability of being a human pathogen** | 0.94 |
| --- | --- |
| **Input proteome coverage (%)** | 26.36 |
| **Matched Pathogenic Families** | 1167 |
| **Matched Not Pathogenic Families** | 3 |

| **Sequences** | 4439 |
| --- | --- |
| **Total bpp** | 1363001 |
| **Longest seq** | 5560 |
| **Shortest seq** | 30 |
| **Avg seq lenght** | 307.0 |

| **Input Sequence** | SM-Roxy-RH01221661_S26_L001_R1_001_(paired)_contig_40_45 # 46729 # 58203 # -1 # ID=40_45;partial=00;start_type=ATG;rbs_motif=AGGAG;rbs_spacer=5-10bp;gc_cont=0.586 | | | | | | |
| --- | --- | --- | --- | --- | --- | --- | --- |
|  | PROJECT ID | ACCESSION ID | ORGANISMS | CLASS | PROTEIN FUNCTION | PROTEIN ID | %IDENTITY |
| **Matched Family** | [30687](http://www.ncbi.nlm.nih.gov/bioproject?db=bioproject&cmd=ShowDetailView&TermToSearch=30687) | [AM933172](http://www.ncbi.nlm.nih.gov/nuccore/AM933172) | Salmonella enterica subsp. enterica serovar Enteritidis str. P125109 complete genome. | Gammaproteobacteria | large repetitive protein | [CAR34191](http://www.ncbi.nlm.nih.gov/protein/CAR34191.1) | 100.0 |
|  | | | | | | | |
|  | | | | | | | |
| ------------------ | | | | | | | |
|  | | | | | | | |
|  | | | | | | | |
| **Input Sequence** | SM-Roxy-RH01221661_S26_L001_R1_001_(paired)_contig_58_4 # 9045 # 10331 # -1 # ID=58_4;partial=00;start_type=ATG;rbs_motif=None;rbs_spacer=None;gc_cont=0.545 | | | | | | |
|  | PROJECT ID | ACCESSION ID | ORGANISMS | CLASS | PROTEIN FUNCTION | PROTEIN ID | %IDENTITY |
| **Matched Family** | [30687](http://www.ncbi.nlm.nih.gov/bioproject?db=bioproject&cmd=ShowDetailView&TermToSearch=30687) | [AM933172](http://www.ncbi.nlm.nih.gov/nuccore/AM933172) | Salmonella enterica subsp. enterica serovar Enteritidis str. P125109 complete genome. | Gammaproteobacteria | putative exported protein (RatA) | [CAR34078](http://www.ncbi.nlm.nih.gov/protein/CAR34078.1) | 100.0 |
|  | | | | | | | |
|  | | | | | | | |
| ------------------ | | | | | | | |
|  | | | | | | | |
|  | | | | | | | |
| **Input Sequence** | SM-Roxy-RH01221661_S26_L001_R1_001_(paired)_contig_41_2 # 1675 # 6060 # -1 # ID=41_2;partial=00;start_type=ATG;rbs_motif=AGGA/GGAG/GAGG;rbs_spacer=11-12bp;gc_cont=0.528 | | | | | | |
|  | PROJECT ID | ACCESSION ID | ORGANISMS | CLASS | PROTEIN FUNCTION | PROTEIN ID | %IDENTITY |
| **Matched Family** | [30687](http://www.ncbi.nlm.nih.gov/bioproject?db=bioproject&cmd=ShowDetailView&TermToSearch=30687) | [AM933172](http://www.ncbi.nlm.nih.gov/nuccore/AM933172) | Salmonella enterica subsp. enterica serovar Enteritidis str. P125109 complete genome. | Gammaproteobacteria | putative exported protein | [CAR35092](http://www.ncbi.nlm.nih.gov/protein/CAR35092.1) | 100.0 |
|  | | | | | | | |
|  | | | | | | | |
| ------------------ | | | | | | | |
|  | | | | | | | |
|  | | | | | | | |
| **Input Sequence** | SM-Roxy-RH01221661_S26_L001_R1_001_(paired)_contig_42_143 # 156302 # 160423 # -1 # ID=42_143;partial=00;start_type=TTG;rbs_motif=GGAG/GAGG;rbs_spacer=5-10bp;gc_cont=0.587 | | | | | | |
|  | PROJECT ID | ACCESSION ID | ORGANISMS | CLASS | PROTEIN FUNCTION | PROTEIN ID | %IDENTITY |
| **Matched Family** | [30687](http://www.ncbi.nlm.nih.gov/bioproject?db=bioproject&cmd=ShowDetailView&TermToSearch=30687) | [AM933172](http://www.ncbi.nlm.nih.gov/nuccore/AM933172) | Salmonella enterica subsp. enterica serovar Enteritidis str. P125109 complete genome. | Gammaproteobacteria | cell division protein FtsK | [CAR32448](http://www.ncbi.nlm.nih.gov/protein/CAR32448.1) | 100.0 |
|  | | | | | | | |
|  | | | | | | | |
| ------------------ | | | | | | | |
|  | | | | | | | |
|  | | | | | | | |
| **Input Sequence** | SM-Roxy-RH01221661_S26_L001_R1_001_(paired)_contig_24_2 # 1585 # 5469 # -1 # ID=24_2;partial=00;start_type=ATG;rbs_motif=AGGAGG;rbs_spacer=3-4bp;gc_cont=0.593 | | | | | | |
|  | PROJECT ID | ACCESSION ID | ORGANISMS | CLASS | PROTEIN FUNCTION | PROTEIN ID | %IDENTITY |
| **Matched Family** | [30687](http://www.ncbi.nlm.nih.gov/bioproject?db=bioproject&cmd=ShowDetailView&TermToSearch=30687) | [AM933172](http://www.ncbi.nlm.nih.gov/nuccore/AM933172) | Salmonella enterica subsp. enterica serovar Enteritidis str. P125109 complete genome. | Gammaproteobacteria | enterobactin synthetase component F | [CAR32145](http://www.ncbi.nlm.nih.gov/protein/CAR32145.1) | 100.0 |
|  | | | | | | | |
|  | | | | | | | |
| ------------------ | | | | | | | |
|  | | | | | | | |
|  | | | | | | | |
| **Input Sequence** | SM-Roxy-RH01221661_S26_L001_R1_001_(paired)_contig_40_35 # 31736 # 35392 # -1 # ID=40_35;partial=00;start_type=ATG;rbs_motif=AGGAGG;rbs_spacer=3-4bp;gc_cont=0.591 | | | | | | |
|  | PROJECT ID | ACCESSION ID | ORGANISMS | CLASS | PROTEIN FUNCTION | PROTEIN ID | %IDENTITY |
| **Matched Family** | [19467](http://www.ncbi.nlm.nih.gov/bioproject?db=bioproject&cmd=ShowDetailView&TermToSearch=19467) | [CP001144](http://www.ncbi.nlm.nih.gov/nuccore/CP001144) | Salmonella enterica subsp. enterica serovar Dublin str. C | Gammaproteobacteria | 2021853, complete genome. | [ACH77810](http://www.ncbi.nlm.nih.gov/protein/ACH77810.1) | 100.0 |
|  | | | | | | | |
|  | | | | | | | |
| ------------------ | | | | | | | |
|  | | | | | | | |
|  | | | | | | | |
| **Input Sequence** | SM-Roxy-RH01221661_S26_L001_R1_001_(paired)_contig_22_121 # 120808 # 121524 # -1 # ID=22_121;partial=00;start_type=ATG;rbs_motif=GGA/GAG/AGG;rbs_spacer=5-10bp;gc_cont=0.548 | | | | | | |
|  | PROJECT ID | ACCESSION ID | ORGANISMS | CLASS | PROTEIN FUNCTION | PROTEIN ID | %IDENTITY |
| **Matched Family** | [18747](http://www.ncbi.nlm.nih.gov/bioproject?db=bioproject&cmd=ShowDetailView&TermToSearch=18747) | [CP001113](http://www.ncbi.nlm.nih.gov/nuccore/CP001113) | Salmonella enterica subsp. enterica serovar Newport str. SL254, complete genome. | Gammaproteobacteria | host specificity protein J | [ACF65560](http://www.ncbi.nlm.nih.gov/protein/ACF65560.1) | 100.0 |
|  | | | | | | | |
|  | | | | | | | |
| ------------------ | | | | | | | |
|  | | | | | | | |
|  | | | | | | | |
| **Input Sequence** | SM-Roxy-RH01221661_S26_L001_R1_001_(paired)_contig_2_70 # 76288 # 79428 # -1 # ID=2_70;partial=00;start_type=ATG;rbs_motif=AGGAG;rbs_spacer=5-10bp;gc_cont=0.584 | | | | | | |
|  | PROJECT ID | ACCESSION ID | ORGANISMS | CLASS | PROTEIN FUNCTION | PROTEIN ID | %IDENTITY |
| **Matched Family** | [30687](http://www.ncbi.nlm.nih.gov/bioproject?db=bioproject&cmd=ShowDetailView&TermToSearch=30687) | [AM933172](http://www.ncbi.nlm.nih.gov/nuccore/AM933172) | Salmonella enterica subsp. enterica serovar Enteritidis str. P125109 complete genome. | Gammaproteobacteria | exonuclease SbcC | [CAR31964](http://www.ncbi.nlm.nih.gov/protein/CAR31964.1) | 100.0 |
|  | | | | | | | |
|  | | | | | | | |
| ------------------ | | | | | | | |
|  | | | | | | | |
|  | | | | | | | |
| **Input Sequence** | SM-Roxy-RH01221661_S26_L001_R1_001_(paired)_contig_12_251 # 251225 # 254287 # -1 # ID=12_251;partial=00;start_type=ATG;rbs_motif=AGGAG;rbs_spacer=5-10bp;gc_cont=0.577 | | | | | | |
|  | PROJECT ID | ACCESSION ID | ORGANISMS | CLASS | PROTEIN FUNCTION | PROTEIN ID | %IDENTITY |
| **Matched Family** | [30687](http://www.ncbi.nlm.nih.gov/bioproject?db=bioproject&cmd=ShowDetailView&TermToSearch=30687) | [AM933172](http://www.ncbi.nlm.nih.gov/nuccore/AM933172) | Salmonella enterica subsp. enterica serovar Enteritidis str. P125109 complete genome. | Gammaproteobacteria | tetrathionate reductase subunit A | [CAR33244](http://www.ncbi.nlm.nih.gov/protein/CAR33244.1) | 100.0 |
|  | | | | | | | |
|  | | | | | | | |
| ------------------ | | | | | | | |
|  | | | | | | | |
|  | | | | | | | |
| **Input Sequence** | SM-Roxy-RH01221661_S26_L001_R1_001_(paired)_contig_9_39 # 42189 # 45221 # 1 # ID=9_39;partial=00;start_type=ATG;rbs_motif=GGAGG;rbs_spacer=3-4bp;gc_cont=0.541 | | | | | | |
|  | PROJECT ID | ACCESSION ID | ORGANISMS | CLASS | PROTEIN FUNCTION | PROTEIN ID | %IDENTITY |
| **Matched Family** | [30687](http://www.ncbi.nlm.nih.gov/bioproject?db=bioproject&cmd=ShowDetailView&TermToSearch=30687) | [AM933172](http://www.ncbi.nlm.nih.gov/nuccore/AM933172) | Salmonella enterica subsp. enterica serovar Enteritidis str. P125109 complete genome. | Gammaproteobacteria | putative viral enhancing factor | [CAR31804](http://www.ncbi.nlm.nih.gov/protein/CAR31804.1) | 100.0 |
|  | | | | | | | |
|  | | | | | | | |
| ------------------ | | | | | | | |
|  | | | | | | | |
|  | | | | | | | |
| **Input Sequence** | SM-Roxy-RH01221661_S26_L001_R1_001_(paired)_contig_2_48 # 55137 # 58151 # 1 # ID=2_48;partial=00;start_type=ATG;rbs_motif=AGGAG;rbs_spacer=5-10bp;gc_cont=0.466 | | | | | | |
|  | PROJECT ID | ACCESSION ID | ORGANISMS | CLASS | PROTEIN FUNCTION | PROTEIN ID | %IDENTITY |
| **Matched Family** | [30687](http://www.ncbi.nlm.nih.gov/bioproject?db=bioproject&cmd=ShowDetailView&TermToSearch=30687) | [AM933172](http://www.ncbi.nlm.nih.gov/nuccore/AM933172) | Salmonella enterica subsp. enterica serovar Enteritidis str. P125109 complete genome. | Gammaproteobacteria | puative autotransporter/virulence factor | [CAR31942](http://www.ncbi.nlm.nih.gov/protein/CAR31942.1) | 100.0 |
|  | | | | | | | |
|  | | | | | | | |
| ------------------ | | | | | | | |
|  | | | | | | | |
|  | | | | | | | |
| **Input Sequence** | SM-Roxy-RH01221661_S26_L001_R1_001_(paired)_contig_35_1 # 15 # 1877 # 1 # ID=35_1;partial=00;start_type=ATG;rbs_motif=None;rbs_spacer=None;gc_cont=0.544 | | | | | | |
|  | PROJECT ID | ACCESSION ID | ORGANISMS | CLASS | PROTEIN FUNCTION | PROTEIN ID | %IDENTITY |
| **Matched Family** | [30687](http://www.ncbi.nlm.nih.gov/bioproject?db=bioproject&cmd=ShowDetailView&TermToSearch=30687) | [AM933172](http://www.ncbi.nlm.nih.gov/nuccore/AM933172) | Salmonella enterica subsp. enterica serovar Enteritidis str. P125109 complete genome. | Gammaproteobacteria | putative chitinase | [CAR31609](http://www.ncbi.nlm.nih.gov/protein/CAR31609.1) | 100.0 |
|  | | | | | | | |
|  | | | | | | | |
| ------------------ | | | | | | | |
|  | | | | | | | |
|  | | | | | | | |
| **Input Sequence** | SM-Roxy-RH01221661_S26_L001_R1_001_(paired)_contig_21_207 # 198829 # 201819 # 1 # ID=21_207;partial=00;start_type=ATG;rbs_motif=GGA/GAG/AGG;rbs_spacer=5-10bp;gc_cont=0.538 | | | | | | |
|  | PROJECT ID | ACCESSION ID | ORGANISMS | CLASS | PROTEIN FUNCTION | PROTEIN ID | %IDENTITY |
| **Matched Family** | [30687](http://www.ncbi.nlm.nih.gov/bioproject?db=bioproject&cmd=ShowDetailView&TermToSearch=30687) | [AM933172](http://www.ncbi.nlm.nih.gov/nuccore/AM933172) | Salmonella enterica subsp. enterica serovar Enteritidis str. P125109 complete genome. | Gammaproteobacteria | putative membrane protein | [CAR33702](http://www.ncbi.nlm.nih.gov/protein/CAR33702.1) | 100.0 |
|  | | | | | | | |
|  | | | | | | | |
| ------------------ | | | | | | | |
|  | | | | | | | |
|  | | | | | | | |
| **Input Sequence** | SM-Roxy-RH01221661_S26_L001_R1_001_(paired)_contig_2_33 # 35271 # 38243 # 1 # ID=2_33;partial=00;start_type=ATG;rbs_motif=GGAG/GAGG;rbs_spacer=5-10bp;gc_cont=0.493 | | | | | | |
|  | PROJECT ID | ACCESSION ID | ORGANISMS | CLASS | PROTEIN FUNCTION | PROTEIN ID | %IDENTITY |
| **Matched Family** | [30687](http://www.ncbi.nlm.nih.gov/bioproject?db=bioproject&cmd=ShowDetailView&TermToSearch=30687) | [AM933172](http://www.ncbi.nlm.nih.gov/nuccore/AM933172) | Salmonella enterica subsp. enterica serovar Enteritidis str. P125109 complete genome. | Gammaproteobacteria | type III restriction-modification system enzyme (StyLTI) (ec 3.1.21.5) | [CAR31927](http://www.ncbi.nlm.nih.gov/protein/CAR31927.1) | 100.0 |
|  | | | | | | | |
|  | | | | | | | |
| ------------------ | | | | | | | |
|  | | | | | | | |
|  | | | | | | | |
| **Input Sequence** | SM-Roxy-RH01221661_S26_L001_R1_001_(paired)_contig_15_10 # 7053 # 9920 # 1 # ID=15_10;partial=00;start_type=ATG;rbs_motif=AGGAGG;rbs_spacer=3-4bp;gc_cont=0.529 | | | | | | |
|  | PROJECT ID | ACCESSION ID | ORGANISMS | CLASS | PROTEIN FUNCTION | PROTEIN ID | %IDENTITY |
| **Matched Family** | [30687](http://www.ncbi.nlm.nih.gov/bioproject?db=bioproject&cmd=ShowDetailView&TermToSearch=30687) | [AM933172](http://www.ncbi.nlm.nih.gov/nuccore/AM933172) | Salmonella enterica subsp. enterica serovar Enteritidis str. P125109 complete genome. | Gammaproteobacteria | putative autotransported protein (MisL) | [CAR35157](http://www.ncbi.nlm.nih.gov/protein/CAR35157.1) | 100.0 |
|  | | | | | | | |
|  | | | | | | | |
| ------------------ | | | | | | | |
|  | | | | | | | |
|  | | | | | | | |
| **Input Sequence** | SM-Roxy-RH01221661_S26_L001_R1_001_(paired)_contig_15_26 # 24783 # 27581 # -1 # ID=15_26;partial=00;start_type=ATG;rbs_motif=None;rbs_spacer=None;gc_cont=0.548 | | | | | | |
|  | PROJECT ID | ACCESSION ID | ORGANISMS | CLASS | PROTEIN FUNCTION | PROTEIN ID | %IDENTITY |
| **Matched Family** | [13086](http://www.ncbi.nlm.nih.gov/bioproject?db=bioproject&cmd=ShowDetailView&TermToSearch=13086) | [CP000026](http://www.ncbi.nlm.nih.gov/nuccore/CP000026) | Salmonella enterica subsp. enterica serovar Paratyphi A str. ATCC 9150, complete genome. | Gammaproteobacteria | putative NtrC family transcriptional regulators, ATPase domain | [AAV79421](http://www.ncbi.nlm.nih.gov/protein/AAV79421.1) | 100.0 |
|  | | | | | | | |
|  | | | | | | | |
| ------------------ | | | | | | | |
|  | | | | | | | |
|  | | | | | | | |
| **Input Sequence** | SM-Roxy-RH01221661_S26_L001_R1_001_(paired)_contig_12_260 # 261731 # 264493 # -1 # ID=12_260;partial=00;start_type=ATG;rbs_motif=GGAGG;rbs_spacer=5-10bp;gc_cont=0.425 | | | | | | |
|  | PROJECT ID | ACCESSION ID | ORGANISMS | CLASS | PROTEIN FUNCTION | PROTEIN ID | %IDENTITY |
| **Matched Family** | [19467](http://www.ncbi.nlm.nih.gov/bioproject?db=bioproject&cmd=ShowDetailView&TermToSearch=19467) | [CP001144](http://www.ncbi.nlm.nih.gov/nuccore/CP001144) | Salmonella enterica subsp. enterica serovar Dublin str. C | Gammaproteobacteria | 2021853, complete genome. | [ACH75069](http://www.ncbi.nlm.nih.gov/protein/ACH75069.1) | 100.0 |
|  | | | | | | | |
|  | | | | | | | |
| ------------------ | | | | | | | |
|  | | | | | | | |
|  | | | | | | | |
| **Input Sequence** | SM-Roxy-RH01221661_S26_L001_R1_001_(paired)_contig_20_37 # 42666 # 45401 # -1 # ID=20_37;partial=00;start_type=ATG;rbs_motif=None;rbs_spacer=None;gc_cont=0.542 | | | | | | |
|  | PROJECT ID | ACCESSION ID | ORGANISMS | CLASS | PROTEIN FUNCTION | PROTEIN ID | %IDENTITY |
| **Matched Family** | [30687](http://www.ncbi.nlm.nih.gov/bioproject?db=bioproject&cmd=ShowDetailView&TermToSearch=30687) | [AM933172](http://www.ncbi.nlm.nih.gov/nuccore/AM933172) | Salmonella enterica subsp. enterica serovar Enteritidis str. P125109 complete genome. | Gammaproteobacteria | Two-component sensor protein histidine protein kinase. | [CAR35218](http://www.ncbi.nlm.nih.gov/protein/CAR35218.1) | 100.0 |
|  | | | | | | | |
|  | | | | | | | |
| ------------------ | | | | | | | |
|  | | | | | | | |
|  | | | | | | | |
| **Input Sequence** | SM-Roxy-RH01221661_S26_L001_R1_001_(paired)_contig_11_240 # 253198 # 255843 # -1 # ID=11_240;partial=00;start_type=ATG;rbs_motif=GGAG/GAGG;rbs_spacer=5-10bp;gc_cont=0.557 | | | | | | |
|  | PROJECT ID | ACCESSION ID | ORGANISMS | CLASS | PROTEIN FUNCTION | PROTEIN ID | %IDENTITY |
| **Matched Family** | [30687](http://www.ncbi.nlm.nih.gov/bioproject?db=bioproject&cmd=ShowDetailView&TermToSearch=30687) | [AM933172](http://www.ncbi.nlm.nih.gov/nuccore/AM933172) | Salmonella enterica subsp. enterica serovar Enteritidis str. P125109 complete genome. | Gammaproteobacteria | outer membrane usher protein | [CAR34374](http://www.ncbi.nlm.nih.gov/protein/CAR34374.1) | 100.0 |
|  | | | | | | | |
|  | | | | | | | |
| ------------------ | | | | | | | |
|  | | | | | | | |
|  | | | | | | | |
| **Input Sequence** | SM-Roxy-RH01221661_S26_L001_R1_001_(paired)_contig_11_252 # 270563 # 273226 # 1 # ID=11_252;partial=00;start_type=GTG;rbs_motif=GGAG/GAGG;rbs_spacer=5-10bp;gc_cont=0.504 | | | | | | |
|  | PROJECT ID | ACCESSION ID | ORGANISMS | CLASS | PROTEIN FUNCTION | PROTEIN ID | %IDENTITY |
| **Matched Family** | [30687](http://www.ncbi.nlm.nih.gov/bioproject?db=bioproject&cmd=ShowDetailView&TermToSearch=30687) | [AM933172](http://www.ncbi.nlm.nih.gov/nuccore/AM933172) | Salmonella enterica subsp. enterica serovar Enteritidis str. P125109 complete genome. | Gammaproteobacteria | conserved hypothetical protein | [CAR34362](http://www.ncbi.nlm.nih.gov/protein/CAR34362.1) | 100.0 |
|  | | | | | | | |
|  | | | | | | | |
| ------------------ | | | | | | | |
|  | | | | | | | |
|  | | | | | | | |
| **Input Sequence** | SM-Roxy-RH01221661_S26_L001_R1_001_(paired)_contig_9_54 # 58547 # 61204 # -1 # ID=9_54;partial=00;start_type=ATG;rbs_motif=GGA/GAG/AGG;rbs_spacer=11-12bp;gc_cont=0.550 | | | | | | |
|  | PROJECT ID | ACCESSION ID | ORGANISMS | CLASS | PROTEIN FUNCTION | PROTEIN ID | %IDENTITY |
| **Matched Family** | [30687](http://www.ncbi.nlm.nih.gov/bioproject?db=bioproject&cmd=ShowDetailView&TermToSearch=30687) | [AM933172](http://www.ncbi.nlm.nih.gov/nuccore/AM933172) | Salmonella enterica subsp. enterica serovar Enteritidis str. P125109 complete genome. | Gammaproteobacteria | outer membrane usher protein stfc (putative fimbrial outer membrane usher) | [CAR31789](http://www.ncbi.nlm.nih.gov/protein/CAR31789.1) | 100.0 |
|  | | | | | | | |
|  | | | | | | | |
| ------------------ | | | | | | | |
|  | | | | | | | |
|  | | | | | | | |
| **Input Sequence** | SM-Roxy-RH01221661_S26_L001_R1_001_(paired)_contig_35_5 # 4626 # 7247 # 1 # ID=35_5;partial=00;start_type=ATG;rbs_motif=None;rbs_spacer=None;gc_cont=0.545 | | | | | | |
|  | PROJECT ID | ACCESSION ID | ORGANISMS | CLASS | PROTEIN FUNCTION | PROTEIN ID | %IDENTITY |
| **Matched Family** | [30687](http://www.ncbi.nlm.nih.gov/bioproject?db=bioproject&cmd=ShowDetailView&TermToSearch=30687) | [AM933172](http://www.ncbi.nlm.nih.gov/nuccore/AM933172) | Salmonella enterica subsp. enterica serovar Enteritidis str. P125109 complete genome. | Gammaproteobacteria | fimbrial usher protein | [CAR31613](http://www.ncbi.nlm.nih.gov/protein/CAR31613.1) | 100.0 |
|  | | | | | | | |
|  | | | | | | | |
| ------------------ | | | | | | | |
|  | | | | | | | |
|  | | | | | | | |
| **Input Sequence** | SM-Roxy-RH01221661_S26_L001_R1_001_(paired)_contig_24_34 # 34658 # 37270 # -1 # ID=24_34;partial=00;start_type=ATG;rbs_motif=GGA/GAG/AGG;rbs_spacer=5-10bp;gc_cont=0.559 | | | | | | |
|  | PROJECT ID | ACCESSION ID | ORGANISMS | CLASS | PROTEIN FUNCTION | PROTEIN ID | %IDENTITY |
| **Matched Family** | [19467](http://www.ncbi.nlm.nih.gov/bioproject?db=bioproject&cmd=ShowDetailView&TermToSearch=19467) | [CP001144](http://www.ncbi.nlm.nih.gov/nuccore/CP001144) | Salmonella enterica subsp. enterica serovar Dublin str. C | Gammaproteobacteria | 2021853, complete genome. | [ACH77796](http://www.ncbi.nlm.nih.gov/protein/ACH77796.1) | 100.0 |
|  | | | | | | | |
|  | | | | | | | |
| ------------------ | | | | | | | |
|  | | | | | | | |
|  | | | | | | | |
| **Input Sequence** | SM-Roxy-RH01221661_S26_L001_R1_001_(paired)_contig_2_14 # 11006 # 13567 # -1 # ID=2_14;partial=00;start_type=ATG;rbs_motif=GGA/GAG/AGG;rbs_spacer=5-10bp;gc_cont=0.542 | | | | | | |
|  | PROJECT ID | ACCESSION ID | ORGANISMS | CLASS | PROTEIN FUNCTION | PROTEIN ID | %IDENTITY |
| **Matched Family** | [30687](http://www.ncbi.nlm.nih.gov/bioproject?db=bioproject&cmd=ShowDetailView&TermToSearch=30687) | [AM933172](http://www.ncbi.nlm.nih.gov/nuccore/AM933172) | Salmonella enterica subsp. enterica serovar Enteritidis str. P125109 complete genome. | Gammaproteobacteria | outer membrane fimbrial usher protein | [CAR31907](http://www.ncbi.nlm.nih.gov/protein/CAR31907.1) | 100.0 |
|  | | | | | | | |
|  | | | | | | | |
| ------------------ | | | | | | | |
|  | | | | | | | |
|  | | | | | | | |
| **Input Sequence** | SM-Roxy-RH01221661_S26_L001_R1_001_(paired)_contig_23_87 # 89031 # 91568 # -1 # ID=23_87;partial=00;start_type=GTG;rbs_motif=AGGA;rbs_spacer=5-10bp;gc_cont=0.471 | | | | | | |
|  | PROJECT ID | ACCESSION ID | ORGANISMS | CLASS | PROTEIN FUNCTION | PROTEIN ID | %IDENTITY |
| **Matched Family** | [30687](http://www.ncbi.nlm.nih.gov/bioproject?db=bioproject&cmd=ShowDetailView&TermToSearch=30687) | [AM933172](http://www.ncbi.nlm.nih.gov/nuccore/AM933172) | Salmonella enterica subsp. enterica serovar Enteritidis str. P125109 complete genome. | Gammaproteobacteria | Outer membrane fimbrial usher protein | [CAR35902](http://www.ncbi.nlm.nih.gov/protein/CAR35902.1) | 100.0 |
|  | | | | | | | |
|  | | | | | | | |
| ------------------ | | | | | | | |
|  | | | | | | | |
|  | | | | | | | |
| **Input Sequence** | SM-Roxy-RH01221661_S26_L001_R1_001_(paired)_contig_4_14 # 10879 # 13407 # 1 # ID=4_14;partial=00;start_type=ATG;rbs_motif=AGGA;rbs_spacer=5-10bp;gc_cont=0.554 | | | | | | |
|  | PROJECT ID | ACCESSION ID | ORGANISMS | CLASS | PROTEIN FUNCTION | PROTEIN ID | %IDENTITY |
| **Matched Family** | [30687](http://www.ncbi.nlm.nih.gov/bioproject?db=bioproject&cmd=ShowDetailView&TermToSearch=30687) | [AM933172](http://www.ncbi.nlm.nih.gov/nuccore/AM933172) | Salmonella enterica subsp. enterica serovar Enteritidis str. P125109 complete genome. | Gammaproteobacteria | outer membrane usher protein (LpfC) | [CAR35038](http://www.ncbi.nlm.nih.gov/protein/CAR35038.1) | 100.0 |
|  | | | | | | | |
|  | | | | | | | |
| ------------------ | | | | | | | |
|  | | | | | | | |
|  | | | | | | | |
| **Input Sequence** | SM-Roxy-RH01221661_S26_L001_R1_001_(paired)_contig_25_156 # 150267 # 152795 # -1 # ID=25_156;partial=00;start_type=ATG;rbs_motif=GGAG/GAGG;rbs_spacer=5-10bp;gc_cont=0.542 | | | | | | |
|  | PROJECT ID | ACCESSION ID | ORGANISMS | CLASS | PROTEIN FUNCTION | PROTEIN ID | %IDENTITY |
| **Matched Family** | [19467](http://www.ncbi.nlm.nih.gov/bioproject?db=bioproject&cmd=ShowDetailView&TermToSearch=19467) | [CP001144](http://www.ncbi.nlm.nih.gov/nuccore/CP001144) | Salmonella enterica subsp. enterica serovar Dublin str. C | Gammaproteobacteria | 2021853, complete genome. | [ACH77850](http://www.ncbi.nlm.nih.gov/protein/ACH77850.1) | 100.0 |
|  | | | | | | | |
|  | | | | | | | |
| ------------------ | | | | | | | |
|  | | | | | | | |
|  | | | | | | | |
| **Input Sequence** | SM-Roxy-RH01221661_S26_L001_R1_001_(paired)_contig_8_7 # 5365 # 7875 # -1 # ID=8_7;partial=00;start_type=ATG;rbs_motif=4Base/6BMM;rbs_spacer=13-15bp;gc_cont=0.547 | | | | | | |
|  | PROJECT ID | ACCESSION ID | ORGANISMS | CLASS | PROTEIN FUNCTION | PROTEIN ID | %IDENTITY |
| **Matched Family** | [30687](http://www.ncbi.nlm.nih.gov/bioproject?db=bioproject&cmd=ShowDetailView&TermToSearch=30687) | [AM933172](http://www.ncbi.nlm.nih.gov/nuccore/AM933172) | Salmonella enterica subsp. enterica serovar Enteritidis str. P125109 complete genome. | Gammaproteobacteria | salmonella atypical fimbria outer membrane usher | [CAR31870](http://www.ncbi.nlm.nih.gov/protein/CAR31870.1) | 100.0 |
|  | | | | | | | |
|  | | | | | | | |
| ------------------ | | | | | | | |
|  | | | | | | | |
|  | | | | | | | |
| **Input Sequence** | SM-Roxy-RH01221661_S26_L001_R1_001_(paired)_contig_60_13 # 15331 # 17832 # -1 # ID=60_13;partial=00;start_type=ATG;rbs_motif=GGAG/GAGG;rbs_spacer=5-10bp;gc_cont=0.578 | | | | | | |
|  | PROJECT ID | ACCESSION ID | ORGANISMS | CLASS | PROTEIN FUNCTION | PROTEIN ID | %IDENTITY |
| **Matched Family** | [30687](http://www.ncbi.nlm.nih.gov/bioproject?db=bioproject&cmd=ShowDetailView&TermToSearch=30687) | [AM933172](http://www.ncbi.nlm.nih.gov/nuccore/AM933172) | Salmonella enterica subsp. enterica serovar Enteritidis str. P125109 complete genome. | Gammaproteobacteria | phosphoenolpyruvate-protein phosphotransferase (ec 2.7.3.9) (phosphotransferase system, enzyme i) (enzyme i-ani) | [CAR35478](http://www.ncbi.nlm.nih.gov/protein/CAR35478.1) | 100.0 |
|  | | | | | | | |
|  | | | | | | | |
| ------------------ | | | | | | | |
|  | | | | | | | |
|  | | | | | | | |
| **Input Sequence** | SM-Roxy-RH01221661_S26_L001_R1_001_(paired)_contig_11_162 # 161277 # 163760 # 1 # ID=11_162;partial=00;start_type=ATG;rbs_motif=None;rbs_spacer=None;gc_cont=0.608 | | | | | | |
|  | PROJECT ID | ACCESSION ID | ORGANISMS | CLASS | PROTEIN FUNCTION | PROTEIN ID | %IDENTITY |
| **Matched Family** | [19467](http://www.ncbi.nlm.nih.gov/bioproject?db=bioproject&cmd=ShowDetailView&TermToSearch=19467) | [CP001144](http://www.ncbi.nlm.nih.gov/nuccore/CP001144) | Salmonella enterica subsp. enterica serovar Dublin str. C | Gammaproteobacteria | 2021853, complete genome. | [ACH76777](http://www.ncbi.nlm.nih.gov/protein/ACH76777.1) | 100.0 |
|  | | | | | | | |
|  | | | | | | | |
| ------------------ | | | | | | | |
|  | | | | | | | |
|  | | | | | | | |
| **Input Sequence** | SM-Roxy-RH01221661_S26_L001_R1_001_(paired)_contig_28_7 # 3263 # 5707 # -1 # ID=28_7;partial=00;start_type=ATG;rbs_motif=GGA/GAG/AGG;rbs_spacer=5-10bp;gc_cont=0.353 | | | | | | |
|  | PROJECT ID | ACCESSION ID | ORGANISMS | CLASS | PROTEIN FUNCTION | PROTEIN ID | %IDENTITY |
| **Matched Family** | [19467](http://www.ncbi.nlm.nih.gov/bioproject?db=bioproject&cmd=ShowDetailView&TermToSearch=19467) | [CP001144](http://www.ncbi.nlm.nih.gov/nuccore/CP001144) | Salmonella enterica subsp. enterica serovar Dublin str. C | Gammaproteobacteria | 2021853, complete genome. | [ACH75604](http://www.ncbi.nlm.nih.gov/protein/ACH75604.1) | 100.0 |
|  | | | | | | | |
|  | | | | | | | |
| ------------------ | | | | | | | |
|  | | | | | | | |
|  | | | | | | | |
| **Input Sequence** | SM-Roxy-RH01221661_S26_L001_R1_001_(paired)_contig_38_76 # 78179 # 80611 # -1 # ID=38_76;partial=00;start_type=ATG;rbs_motif=GGAG/GAGG;rbs_spacer=5-10bp;gc_cont=0.558 | | | | | | |
|  | PROJECT ID | ACCESSION ID | ORGANISMS | CLASS | PROTEIN FUNCTION | PROTEIN ID | %IDENTITY |
| **Matched Family** | [20063](http://www.ncbi.nlm.nih.gov/bioproject?db=bioproject&cmd=ShowDetailView&TermToSearch=20063) | [CP001138](http://www.ncbi.nlm.nih.gov/nuccore/CP001138) | Salmonella enterica subsp. enterica serovar Agona str. SL483, complete genome. | Gammaproteobacteria | formate C-acetyltransferase 3 | [ACH51765](http://www.ncbi.nlm.nih.gov/protein/ACH51765.1) | 100.0 |
|  | | | | | | | |
|  | | | | | | | |
| ------------------ | | | | | | | |
|  | | | | | | | |
|  | | | | | | | |
| **Input Sequence** | SM-Roxy-RH01221661_S26_L001_R1_001_(paired)_contig_43_8 # 5915 # 8281 # -1 # ID=43_8;partial=00;start_type=ATG;rbs_motif=GGA/GAG/AGG;rbs_spacer=5-10bp;gc_cont=0.555 | | | | | | |
|  | PROJECT ID | ACCESSION ID | ORGANISMS | CLASS | PROTEIN FUNCTION | PROTEIN ID | %IDENTITY |
| **Matched Family** | [19467](http://www.ncbi.nlm.nih.gov/bioproject?db=bioproject&cmd=ShowDetailView&TermToSearch=19467) | [CP001144](http://www.ncbi.nlm.nih.gov/nuccore/CP001144) | Salmonella enterica subsp. enterica serovar Dublin str. C | Gammaproteobacteria | 2021853, complete genome. | [ACH76993](http://www.ncbi.nlm.nih.gov/protein/ACH76993.1) | 100.0 |
|  | | | | | | | |
|  | | | | | | | |
| ------------------ | | | | | | | |
|  | | | | | | | |
|  | | | | | | | |
| **Input Sequence** | SM-Roxy-RH01221661_S26_L001_R1_001_(paired)_contig_21_149 # 130671 # 133019 # 1 # ID=21_149;partial=00;start_type=ATG;rbs_motif=AGGA;rbs_spacer=5-10bp;gc_cont=0.476 | | | | | | |
|  | PROJECT ID | ACCESSION ID | ORGANISMS | CLASS | PROTEIN FUNCTION | PROTEIN ID | %IDENTITY |
| **Matched Family** | [30687](http://www.ncbi.nlm.nih.gov/bioproject?db=bioproject&cmd=ShowDetailView&TermToSearch=30687) | [AM933172](http://www.ncbi.nlm.nih.gov/nuccore/AM933172) | Salmonella enterica subsp. enterica serovar Enteritidis str. P125109 complete genome. | Gammaproteobacteria | secreted protein SopA | [CAR33644](http://www.ncbi.nlm.nih.gov/protein/CAR33644.1) | 100.0 |
|  | | | | | | | |
|  | | | | | | | |
| ------------------ | | | | | | | |
|  | | | | | | | |
|  | | | | | | | |
| **Input Sequence** | SM-Roxy-RH01221661_S26_L001_R1_001_(paired)_contig_47_57 # 56675 # 58993 # -1 # ID=47_57;partial=00;start_type=ATG;rbs_motif=AGGA;rbs_spacer=5-10bp;gc_cont=0.564 | | | | | | |
|  | PROJECT ID | ACCESSION ID | ORGANISMS | CLASS | PROTEIN FUNCTION | PROTEIN ID | %IDENTITY |
| **Matched Family** | [30687](http://www.ncbi.nlm.nih.gov/bioproject?db=bioproject&cmd=ShowDetailView&TermToSearch=30687) | [AM933172](http://www.ncbi.nlm.nih.gov/nuccore/AM933172) | Salmonella enterica subsp. enterica serovar Enteritidis str. P125109 complete genome. | Gammaproteobacteria | putative glycosyl hydrolase | [CAR35149](http://www.ncbi.nlm.nih.gov/protein/CAR35149.1) | 100.0 |
|  | | | | | | | |
|  | | | | | | | |
| ------------------ | | | | | | | |
|  | | | | | | | |
|  | | | | | | | |
| **Input Sequence** | SM-Roxy-RH01221661_S26_L001_R1_001_(paired)_contig_10_15 # 17985 # 20255 # 1 # ID=10_15;partial=00;start_type=ATG;rbs_motif=None;rbs_spacer=None;gc_cont=0.539 | | | | | | |
|  | PROJECT ID | ACCESSION ID | ORGANISMS | CLASS | PROTEIN FUNCTION | PROTEIN ID | %IDENTITY |
| **Matched Family** | [18747](http://www.ncbi.nlm.nih.gov/bioproject?db=bioproject&cmd=ShowDetailView&TermToSearch=18747) | [CP001113](http://www.ncbi.nlm.nih.gov/nuccore/CP001113) | Salmonella enterica subsp. enterica serovar Newport str. SL254, complete genome. | Gammaproteobacteria | biodegradative arginine decarboxylase | [ACF63192](http://www.ncbi.nlm.nih.gov/protein/ACF63192.1) | 100.0 |
|  | | | | | | | |
|  | | | | | | | |
| ------------------ | | | | | | | |
|  | | | | | | | |
|  | | | | | | | |
| **Input Sequence** | SM-Roxy-RH01221661_S26_L001_R1_001_(paired)_contig_38_31 # 34034 # 34870 # 1 # ID=38_31;partial=00;start_type=ATG;rbs_motif=GGA/GAG/AGG;rbs_spacer=5-10bp;gc_cont=0.421 | | | | | | |
|  | PROJECT ID | ACCESSION ID | ORGANISMS | CLASS | PROTEIN FUNCTION | PROTEIN ID | %IDENTITY |
| **Matched Family** | [18747](http://www.ncbi.nlm.nih.gov/bioproject?db=bioproject&cmd=ShowDetailView&TermToSearch=18747) | [CP001113](http://www.ncbi.nlm.nih.gov/nuccore/CP001113) | Salmonella enterica subsp. enterica serovar Newport str. SL254, complete genome. | Gammaproteobacteria | leucine-rich repeat protein | [ACF64457](http://www.ncbi.nlm.nih.gov/protein/ACF64457.1) | 100.0 |
|  | | | | | | | |
|  | | | | | | | |
| ------------------ | | | | | | | |
|  | | | | | | | |
|  | | | | | | | |
| **Input Sequence** | SM-Roxy-RH01221661_S26_L001_R1_001_(paired)_contig_9_60 # 66577 # 68820 # -1 # ID=9_60;partial=00;start_type=ATG;rbs_motif=GGA/GAG/AGG;rbs_spacer=5-10bp;gc_cont=0.516 | | | | | | |
|  | PROJECT ID | ACCESSION ID | ORGANISMS | CLASS | PROTEIN FUNCTION | PROTEIN ID | %IDENTITY |
| **Matched Family** | [20063](http://www.ncbi.nlm.nih.gov/bioproject?db=bioproject&cmd=ShowDetailView&TermToSearch=20063) | [CP001138](http://www.ncbi.nlm.nih.gov/nuccore/CP001138) | Salmonella enterica subsp. enterica serovar Agona str. SL483, complete genome. | Gammaproteobacteria | ferrichrome-iron receptor | [ACH51827](http://www.ncbi.nlm.nih.gov/protein/ACH51827.1) | 100.0 |
|  | | | | | | | |
|  | | | | | | | |
| ------------------ | | | | | | | |
|  | | | | | | | |
|  | | | | | | | |
| **Input Sequence** | SM-Roxy-RH01221661_S26_L001_R1_001_(paired)_contig_67_15 # 16572 # 18452 # 1 # ID=67_15;partial=00;start_type=ATG;rbs_motif=GGA/GAG/AGG;rbs_spacer=3-4bp;gc_cont=0.533 | | | | | | |
|  | PROJECT ID | ACCESSION ID | ORGANISMS | CLASS | PROTEIN FUNCTION | PROTEIN ID | %IDENTITY |
| **Matched Family** | [19467](http://www.ncbi.nlm.nih.gov/bioproject?db=bioproject&cmd=ShowDetailView&TermToSearch=19467) | [CP001144](http://www.ncbi.nlm.nih.gov/nuccore/CP001144) | Salmonella enterica subsp. enterica serovar Dublin str. C | Gammaproteobacteria | 2021853, complete genome. | [ACH75028](http://www.ncbi.nlm.nih.gov/protein/ACH75028.1) | 100.0 |
|  | | | | | | | |
|  | | | | | | | |
| ------------------ | | | | | | | |
|  | | | | | | | |
|  | | | | | | | |
| **Input Sequence** | SM-Roxy-RH01221661_S26_L001_R1_001_(paired)_contig_3_32 # 30356 # 32545 # 1 # ID=3_32;partial=00;start_type=ATG;rbs_motif=GGA/GAG/AGG;rbs_spacer=5-10bp;gc_cont=0.509 | | | | | | |
|  | PROJECT ID | ACCESSION ID | ORGANISMS | CLASS | PROTEIN FUNCTION | PROTEIN ID | %IDENTITY |
| **Matched Family** | [30687](http://www.ncbi.nlm.nih.gov/bioproject?db=bioproject&cmd=ShowDetailView&TermToSearch=30687) | [AM933172](http://www.ncbi.nlm.nih.gov/nuccore/AM933172) | Salmonella enterica subsp. enterica serovar Enteritidis str. P125109 complete genome. | Gammaproteobacteria | putative membrane protein | [CAR33982](http://www.ncbi.nlm.nih.gov/protein/CAR33982.1) | 100.0 |
|  | | | | | | | |
|  | | | | | | | |
| ------------------ | | | | | | | |
|  | | | | | | | |
|  | | | | | | | |
| **Input Sequence** | SM-Roxy-RH01221661_S26_L001_R1_001_(paired)_contig_40_32 # 27205 # 29379 # 1 # ID=40_32;partial=00;start_type=ATG;rbs_motif=GGA/GAG/AGG;rbs_spacer=5-10bp;gc_cont=0.510 | | | | | | |
|  | PROJECT ID | ACCESSION ID | ORGANISMS | CLASS | PROTEIN FUNCTION | PROTEIN ID | %IDENTITY |
| **Matched Family** | [30687](http://www.ncbi.nlm.nih.gov/bioproject?db=bioproject&cmd=ShowDetailView&TermToSearch=30687) | [AM933172](http://www.ncbi.nlm.nih.gov/nuccore/AM933172) | Salmonella enterica subsp. enterica serovar Enteritidis str. P125109 complete genome. | Gammaproteobacteria | TonB-dependent outer membrane siderophore receptor protein | [CAR34203](http://www.ncbi.nlm.nih.gov/protein/CAR34203.1) | 100.0 |
|  | | | | | | | |
|  | | | | | | | |
| ------------------ | | | | | | | |
|  | | | | | | | |
|  | | | | | | | |
| **Input Sequence** | SM-Roxy-RH01221661_S26_L001_R1_001_(paired)_contig_21_92 # 75552 # 77720 # 1 # ID=21_92;partial=00;start_type=TTG;rbs_motif=AGGAG;rbs_spacer=5-10bp;gc_cont=0.431 | | | | | | |
|  | PROJECT ID | ACCESSION ID | ORGANISMS | CLASS | PROTEIN FUNCTION | PROTEIN ID | %IDENTITY |
| **Matched Family** | [19467](http://www.ncbi.nlm.nih.gov/bioproject?db=bioproject&cmd=ShowDetailView&TermToSearch=19467) | [CP001144](http://www.ncbi.nlm.nih.gov/nuccore/CP001144) | Salmonella enterica subsp. enterica serovar Dublin str. C | Gammaproteobacteria | 2021853, complete genome. | [ACH76603](http://www.ncbi.nlm.nih.gov/protein/ACH76603.1) | 100.0 |
|  | | | | | | | |
|  | | | | | | | |
| ------------------ | | | | | | | |
|  | | | | | | | |
|  | | | | | | | |
| **Input Sequence** | SM-Roxy-RH01221661_S26_L001_R1_001_(paired)_contig_25_193 # 196122 # 198266 # 1 # ID=25_193;partial=00;start_type=ATG;rbs_motif=AGGA/GGAG/GAGG;rbs_spacer=11-12bp;gc_cont=0.572 | | | | | | |
|  | PROJECT ID | ACCESSION ID | ORGANISMS | CLASS | PROTEIN FUNCTION | PROTEIN ID | %IDENTITY |
| **Matched Family** | [30687](http://www.ncbi.nlm.nih.gov/bioproject?db=bioproject&cmd=ShowDetailView&TermToSearch=30687) | [AM933172](http://www.ncbi.nlm.nih.gov/nuccore/AM933172) | Salmonella enterica subsp. enterica serovar Enteritidis str. P125109 complete genome. | Gammaproteobacteria | putative virulence effector protein | [CAR33039](http://www.ncbi.nlm.nih.gov/protein/CAR33039.1) | 100.0 |
|  | | | | | | | |
|  | | | | | | | |
| ------------------ | | | | | | | |
|  | | | | | | | |
|  | | | | | | | |
| **Input Sequence** | SM-Roxy-RH01221661_S26_L001_R1_001_(paired)_contig_9_15 # 11158 # 13299 # -1 # ID=9_15;partial=00;start_type=ATG;rbs_motif=AGGAG;rbs_spacer=5-10bp;gc_cont=0.520 | | | | | | |
|  | PROJECT ID | ACCESSION ID | ORGANISMS | CLASS | PROTEIN FUNCTION | PROTEIN ID | %IDENTITY |
| **Matched Family** | [30687](http://www.ncbi.nlm.nih.gov/bioproject?db=bioproject&cmd=ShowDetailView&TermToSearch=30687) | [AM933172](http://www.ncbi.nlm.nih.gov/nuccore/AM933172) | Salmonella enterica subsp. enterica serovar Enteritidis str. P125109 complete genome. | Gammaproteobacteria | lysine decarboxylase | [CAR31829](http://www.ncbi.nlm.nih.gov/protein/CAR31829.1) | 100.0 |
|  | | | | | | | |
|  | | | | | | | |
| ------------------ | | | | | | | |
|  | | | | | | | |
|  | | | | | | | |
| **Input Sequence** | SM-Roxy-RH01221661_S26_L001_R1_001_(paired)_contig_21_219 # 217756 # 219867 # 1 # ID=21_219;partial=00;start_type=ATG;rbs_motif=AGGA;rbs_spacer=5-10bp;gc_cont=0.536 | | | | | | |
|  | PROJECT ID | ACCESSION ID | ORGANISMS | CLASS | PROTEIN FUNCTION | PROTEIN ID | %IDENTITY |
| **Matched Family** | [30687](http://www.ncbi.nlm.nih.gov/bioproject?db=bioproject&cmd=ShowDetailView&TermToSearch=30687) | [AM933172](http://www.ncbi.nlm.nih.gov/nuccore/AM933172) | Salmonella enterica subsp. enterica serovar Enteritidis str. P125109 complete genome. | Gammaproteobacteria | hypothetical protein | [CAR33713](http://www.ncbi.nlm.nih.gov/protein/CAR33713.1) | 100.0 |
|  | | | | | | | |
|  | | | | | | | |
| ------------------ | | | | | | | |
|  | | | | | | | |
|  | | | | | | | |
| **Input Sequence** | SM-Roxy-RH01221661_S26_L001_R1_001_(paired)_contig_2_39 # 42654 # 44744 # 1 # ID=2_39;partial=00;start_type=ATG;rbs_motif=GGAG/GAGG;rbs_spacer=5-10bp;gc_cont=0.527 | | | | | | |
|  | PROJECT ID | ACCESSION ID | ORGANISMS | CLASS | PROTEIN FUNCTION | PROTEIN ID | %IDENTITY |
| **Matched Family** | [30687](http://www.ncbi.nlm.nih.gov/bioproject?db=bioproject&cmd=ShowDetailView&TermToSearch=30687) | [AM933172](http://www.ncbi.nlm.nih.gov/nuccore/AM933172) | Salmonella enterica subsp. enterica serovar Enteritidis str. P125109 complete genome. | Gammaproteobacteria | ferrioxamine B receptor precursor | [CAR31933](http://www.ncbi.nlm.nih.gov/protein/CAR31933.1) | 100.0 |
|  | | | | | | | |
|  | | | | | | | |
| ------------------ | | | | | | | |
|  | | | | | | | |
|  | | | | | | | |
| **Input Sequence** | SM-Roxy-RH01221661_S26_L001_R1_001_(paired)_contig_23_111 # 114820 # 116919 # 1 # ID=23_111;partial=00;start_type=ATG;rbs_motif=AGGA;rbs_spacer=5-10bp;gc_cont=0.511 | | | | | | |
|  | PROJECT ID | ACCESSION ID | ORGANISMS | CLASS | PROTEIN FUNCTION | PROTEIN ID | %IDENTITY |
| **Matched Family** | [30687](http://www.ncbi.nlm.nih.gov/bioproject?db=bioproject&cmd=ShowDetailView&TermToSearch=30687) | [AM933172](http://www.ncbi.nlm.nih.gov/nuccore/AM933172) | Salmonella enterica subsp. enterica serovar Enteritidis str. P125109 complete genome. | Gammaproteobacteria | putative chitinase | [CAR31608](http://www.ncbi.nlm.nih.gov/protein/CAR31608.1) | 100.0 |
|  | | | | | | | |
|  | | | | | | | |
| ------------------ | | | | | | | |
|  | | | | | | | |
|  | | | | | | | |
| **Input Sequence** | SM-Roxy-RH01221661_S26_L001_R1_001_(paired)_contig_11_298 # 315935 # 317992 # 1 # ID=11_298;partial=00;start_type=GTG;rbs_motif=AGGA;rbs_spacer=5-10bp;gc_cont=0.455 | | | | | | |
|  | PROJECT ID | ACCESSION ID | ORGANISMS | CLASS | PROTEIN FUNCTION | PROTEIN ID | %IDENTITY |
| **Matched Family** | [13086](http://www.ncbi.nlm.nih.gov/bioproject?db=bioproject&cmd=ShowDetailView&TermToSearch=13086) | [CP000026](http://www.ncbi.nlm.nih.gov/nuccore/CP000026) | Salmonella enterica subsp. enterica serovar Paratyphi A str. ATCC 9150, complete genome. | Gammaproteobacteria | possible secretory protein (associated with virulence) | [AAV78611](http://www.ncbi.nlm.nih.gov/protein/AAV78611.1) | 100.0 |
|  | | | | | | | |
|  | | | | | | | |
| ------------------ | | | | | | | |
|  | | | | | | | |
|  | | | | | | | |
| **Input Sequence** | SM-Roxy-RH01221661_S26_L001_R1_001_(paired)_contig_11_312 # 329656 # 331713 # 1 # ID=11_312;partial=00;start_type=ATG;rbs_motif=GGAG/GAGG;rbs_spacer=5-10bp;gc_cont=0.480 | | | | | | |
|  | PROJECT ID | ACCESSION ID | ORGANISMS | CLASS | PROTEIN FUNCTION | PROTEIN ID | %IDENTITY |
| **Matched Family** | [30687](http://www.ncbi.nlm.nih.gov/bioproject?db=bioproject&cmd=ShowDetailView&TermToSearch=30687) | [AM933172](http://www.ncbi.nlm.nih.gov/nuccore/AM933172) | Salmonella enterica subsp. enterica serovar Enteritidis str. P125109 complete genome. | Gammaproteobacteria | pathogenicity island 1 effector protein (function unknown) | [CAR34301](http://www.ncbi.nlm.nih.gov/protein/CAR34301.1) | 100.0 |
|  | | | | | | | |
|  | | | | | | | |
| ------------------ | | | | | | | |
|  | | | | | | | |
|  | | | | | | | |
| **Input Sequence** | SM-Roxy-RH01221661_S26_L001_R1_001_(paired)_contig_25_18 # 10304 # 12349 # 1 # ID=25_18;partial=00;start_type=ATG;rbs_motif=GGAG/GAGG;rbs_spacer=5-10bp;gc_cont=0.465 | | | | | | |
|  | PROJECT ID | ACCESSION ID | ORGANISMS | CLASS | PROTEIN FUNCTION | PROTEIN ID | %IDENTITY |
| **Matched Family** | [18747](http://www.ncbi.nlm.nih.gov/bioproject?db=bioproject&cmd=ShowDetailView&TermToSearch=18747) | [CP001113](http://www.ncbi.nlm.nih.gov/nuccore/CP001113) | Salmonella enterica subsp. enterica serovar Newport str. SL254, complete genome. | Gammaproteobacteria | secretion system apparatus protein SsaV | [ACF62685](http://www.ncbi.nlm.nih.gov/protein/ACF62685.1) | 100.0 |
|  | | | | | | | |
|  | | | | | | | |
| ------------------ | | | | | | | |
|  | | | | | | | |
|  | | | | | | | |
| **Input Sequence** | SM-Roxy-RH01221661_S26_L001_R1_001_(paired)_contig_35_24 # 27183 # 29222 # -1 # ID=35_24;partial=00;start_type=ATG;rbs_motif=GGAGG;rbs_spacer=5-10bp;gc_cont=0.544 | | | | | | |
|  | PROJECT ID | ACCESSION ID | ORGANISMS | CLASS | PROTEIN FUNCTION | PROTEIN ID | %IDENTITY |
| **Matched Family** | [19467](http://www.ncbi.nlm.nih.gov/bioproject?db=bioproject&cmd=ShowDetailView&TermToSearch=19467) | [CP001144](http://www.ncbi.nlm.nih.gov/nuccore/CP001144) | Salmonella enterica subsp. enterica serovar Dublin str. C | Gammaproteobacteria | 2021853, complete genome. | [ACH74046](http://www.ncbi.nlm.nih.gov/protein/ACH74046.1) | 100.0 |
|  | | | | | | | |
|  | | | | | | | |
| ------------------ | | | | | | | |
|  | | | | | | | |
|  | | | | | | | |
| **Input Sequence** | SM-Roxy-RH01221661_S26_L001_R1_001_(paired)_contig_5_27 # 27398 # 29434 # -1 # ID=5_27;partial=00;start_type=ATG;rbs_motif=GGAG/GAGG;rbs_spacer=5-10bp;gc_cont=0.550 | | | | | | |
|  | PROJECT ID | ACCESSION ID | ORGANISMS | CLASS | PROTEIN FUNCTION | PROTEIN ID | %IDENTITY |
| **Matched Family** | [30687](http://www.ncbi.nlm.nih.gov/bioproject?db=bioproject&cmd=ShowDetailView&TermToSearch=30687) | [AM933172](http://www.ncbi.nlm.nih.gov/nuccore/AM933172) | Salmonella enterica subsp. enterica serovar Enteritidis str. P125109 complete genome. | Gammaproteobacteria | putative glycosyl hydrolase | [CAR35381](http://www.ncbi.nlm.nih.gov/protein/CAR35381.1) | 100.0 |
|  | | | | | | | |
|  | | | | | | | |
| ------------------ | | | | | | | |
|  | | | | | | | |
|  | | | | | | | |
| **Input Sequence** | SM-Roxy-RH01221661_S26_L001_R1_001_(paired)_contig_41_29 # 34691 # 36718 # -1 # ID=41_29;partial=00;start_type=ATG;rbs_motif=AGGA;rbs_spacer=5-10bp;gc_cont=0.555 | | | | | | |
|  | PROJECT ID | ACCESSION ID | ORGANISMS | CLASS | PROTEIN FUNCTION | PROTEIN ID | %IDENTITY |
| **Matched Family** | [30687](http://www.ncbi.nlm.nih.gov/bioproject?db=bioproject&cmd=ShowDetailView&TermToSearch=30687) | [AM933172](http://www.ncbi.nlm.nih.gov/nuccore/AM933172) | Salmonella enterica subsp. enterica serovar Enteritidis str. P125109 complete genome. | Gammaproteobacteria | alpha-amylase | [CAR35065](http://www.ncbi.nlm.nih.gov/protein/CAR35065.1) | 100.0 |
|  | | | | | | | |
|  | | | | | | | |
| ------------------ | | | | | | | |
|  | | | | | | | |
|  | | | | | | | |
| **Input Sequence** | SM-Roxy-RH01221661_S26_L001_R1_001_(paired)_contig_3_46 # 48600 # 50606 # 1 # ID=3_46;partial=00;start_type=ATG;rbs_motif=GGA/GAG/AGG;rbs_spacer=5-10bp;gc_cont=0.537 | | | | | | |
|  | PROJECT ID | ACCESSION ID | ORGANISMS | CLASS | PROTEIN FUNCTION | PROTEIN ID | %IDENTITY |
| **Matched Family** | [30687](http://www.ncbi.nlm.nih.gov/bioproject?db=bioproject&cmd=ShowDetailView&TermToSearch=30687) | [AM933172](http://www.ncbi.nlm.nih.gov/nuccore/AM933172) | Salmonella enterica subsp. enterica serovar Enteritidis str. P125109 complete genome. | Gammaproteobacteria | phosphoglycerate transport system sensor protein PgtB | [CAR33969](http://www.ncbi.nlm.nih.gov/protein/CAR33969.1) | 100.0 |
|  | | | | | | | |
|  | | | | | | | |
| ------------------ | | | | | | | |
|  | | | | | | | |
|  | | | | | | | |
| **Input Sequence** | SM-Roxy-RH01221661_S26_L001_R1_001_(paired)_contig_30_134 # 135735 # 137717 # -1 # ID=30_134;partial=00;start_type=ATG;rbs_motif=None;rbs_spacer=None;gc_cont=0.526 | | | | | | |
|  | PROJECT ID | ACCESSION ID | ORGANISMS | CLASS | PROTEIN FUNCTION | PROTEIN ID | %IDENTITY |
| **Matched Family** | [13086](http://www.ncbi.nlm.nih.gov/bioproject?db=bioproject&cmd=ShowDetailView&TermToSearch=13086) | [CP000026](http://www.ncbi.nlm.nih.gov/nuccore/CP000026) | Salmonella enterica subsp. enterica serovar Paratyphi A str. ATCC 9150, complete genome. | Gammaproteobacteria | RNA polymerase sigma-70 factor | [AAV78914](http://www.ncbi.nlm.nih.gov/protein/AAV78914.1) | 100.0 |
|  | | | | | | | |
|  | | | | | | | |
| ------------------ | | | | | | | |
|  | | | | | | | |
|  | | | | | | | |
| **Input Sequence** | SM-Roxy-RH01221661_S26_L001_R1_001_(paired)_contig_44_89 # 90082 # 92064 # 1 # ID=44_89;partial=00;start_type=GTG;rbs_motif=AGGAG;rbs_spacer=5-10bp;gc_cont=0.513 | | | | | | |
|  | PROJECT ID | ACCESSION ID | ORGANISMS | CLASS | PROTEIN FUNCTION | PROTEIN ID | %IDENTITY |
| **Matched Family** | [30687](http://www.ncbi.nlm.nih.gov/bioproject?db=bioproject&cmd=ShowDetailView&TermToSearch=30687) | [AM933172](http://www.ncbi.nlm.nih.gov/nuccore/AM933172) | Salmonella enterica subsp. enterica serovar Enteritidis str. P125109 complete genome. | Gammaproteobacteria | invasin-like protein | [CAR32941](http://www.ncbi.nlm.nih.gov/protein/CAR32941.1) | 100.0 |
|  | | | | | | | |
|  | | | | | | | |
| ------------------ | | | | | | | |
|  | | | | | | | |
|  | | | | | | | |
| **Input Sequence** | SM-Roxy-RH01221661_S26_L001_R1_001_(paired)_contig_24_13 # 14170 # 16140 # -1 # ID=24_13;partial=00;start_type=ATG;rbs_motif=AGGA;rbs_spacer=5-10bp;gc_cont=0.563 | | | | | | |
|  | PROJECT ID | ACCESSION ID | ORGANISMS | CLASS | PROTEIN FUNCTION | PROTEIN ID | %IDENTITY |
| **Matched Family** | [30687](http://www.ncbi.nlm.nih.gov/bioproject?db=bioproject&cmd=ShowDetailView&TermToSearch=30687) | [AM933172](http://www.ncbi.nlm.nih.gov/nuccore/AM933172) | Salmonella enterica subsp. enterica serovar Enteritidis str. P125109 complete genome. | Gammaproteobacteria | outer membrane esterase | [CAR32134](http://www.ncbi.nlm.nih.gov/protein/CAR32134.1) | 100.0 |
|  | | | | | | | |
|  | | | | | | | |
| ------------------ | | | | | | | |
|  | | | | | | | |
|  | | | | | | | |
| **Input Sequence** | SM-Roxy-RH01221661_S26_L001_R1_001_(paired)_contig_2_32 # 33303 # 35261 # 1 # ID=2_32;partial=00;start_type=ATG;rbs_motif=GGAG/GAGG;rbs_spacer=5-10bp;gc_cont=0.466 | | | | | | |
|  | PROJECT ID | ACCESSION ID | ORGANISMS | CLASS | PROTEIN FUNCTION | PROTEIN ID | %IDENTITY |
| **Matched Family** | [30687](http://www.ncbi.nlm.nih.gov/bioproject?db=bioproject&cmd=ShowDetailView&TermToSearch=30687) | [AM933172](http://www.ncbi.nlm.nih.gov/nuccore/AM933172) | Salmonella enterica subsp. enterica serovar Enteritidis str. P125109 complete genome. | Gammaproteobacteria | type III restriction-modification system enzyme (StyLTI) modification methylase | [CAR31926](http://www.ncbi.nlm.nih.gov/protein/CAR31926.1) | 100.0 |
|  | | | | | | | |
|  | | | | | | | |
| ------------------ | | | | | | | |
|  | | | | | | | |
|  | | | | | | | |
| **Input Sequence** | SM-Roxy-RH01221661_S26_L001_R1_001_(paired)_contig_41_14 # 19394 # 21349 # -1 # ID=41_14;partial=00;start_type=ATG;rbs_motif=AGGAG;rbs_spacer=5-10bp;gc_cont=0.573 | | | | | | |
|  | PROJECT ID | ACCESSION ID | ORGANISMS | CLASS | PROTEIN FUNCTION | PROTEIN ID | %IDENTITY |
| **Matched Family** | [30687](http://www.ncbi.nlm.nih.gov/bioproject?db=bioproject&cmd=ShowDetailView&TermToSearch=30687) | [AM933172](http://www.ncbi.nlm.nih.gov/nuccore/AM933172) | Salmonella enterica subsp. enterica serovar Enteritidis str. P125109 complete genome. | Gammaproteobacteria | conserved hypothetical protein | [CAR35080](http://www.ncbi.nlm.nih.gov/protein/CAR35080.1) | 100.0 |
|  | | | | | | | |
|  | | | | | | | |
| ------------------ | | | | | | | |
|  | | | | | | | |
|  | | | | | | | |
| **Input Sequence** | SM-Roxy-RH01221661_S26_L001_R1_001_(paired)_contig_14_19 # 18256 # 20184 # 1 # ID=14_19;partial=00;start_type=ATG;rbs_motif=AGGA;rbs_spacer=5-10bp;gc_cont=0.501 | | | | | | |
|  | PROJECT ID | ACCESSION ID | ORGANISMS | CLASS | PROTEIN FUNCTION | PROTEIN ID | %IDENTITY |
| **Matched Family** | [20063](http://www.ncbi.nlm.nih.gov/bioproject?db=bioproject&cmd=ShowDetailView&TermToSearch=20063) | [CP001138](http://www.ncbi.nlm.nih.gov/nuccore/CP001138) | Salmonella enterica subsp. enterica serovar Agona str. SL483, complete genome. | Gammaproteobacteria | putative sigma-54 dependent transcriptional regulator | [ACH50577](http://www.ncbi.nlm.nih.gov/protein/ACH50577.1) | 100.0 |
|  | | | | | | | |
|  | | | | | | | |
| ------------------ | | | | | | | |
|  | | | | | | | |
|  | | | | | | | |
| **Input Sequence** | SM-Roxy-RH01221661_S26_L001_R1_001_(paired)_contig_3_50 # 54551 # 56473 # -1 # ID=3_50;partial=00;start_type=TTG;rbs_motif=GGAG/GAGG;rbs_spacer=5-10bp;gc_cont=0.334 | | | | | | |
|  | PROJECT ID | ACCESSION ID | ORGANISMS | CLASS | PROTEIN FUNCTION | PROTEIN ID | %IDENTITY |
| **Matched Family** | [30687](http://www.ncbi.nlm.nih.gov/bioproject?db=bioproject&cmd=ShowDetailView&TermToSearch=30687) | [AM933172](http://www.ncbi.nlm.nih.gov/nuccore/AM933172) | Salmonella enterica subsp. enterica serovar Enteritidis str. P125109 complete genome. | Gammaproteobacteria | putative lipopolysaccharide modification acyltransferase | [CAR33964](http://www.ncbi.nlm.nih.gov/protein/CAR33964.1) | 100.0 |
|  | | | | | | | |
|  | | | | | | | |
| ------------------ | | | | | | | |
|  | | | | | | | |
|  | | | | | | | |
| **Input Sequence** | SM-Roxy-RH01221661_S26_L001_R1_001_(paired)_contig_53_27 # 29158 # 31047 # 1 # ID=53_27;partial=00;start_type=ATG;rbs_motif=GGA/GAG/AGG;rbs_spacer=5-10bp;gc_cont=0.465 | | | | | | |
|  | PROJECT ID | ACCESSION ID | ORGANISMS | CLASS | PROTEIN FUNCTION | PROTEIN ID | %IDENTITY |
| **Matched Family** | [30687](http://www.ncbi.nlm.nih.gov/bioproject?db=bioproject&cmd=ShowDetailView&TermToSearch=30687) | [AM933172](http://www.ncbi.nlm.nih.gov/nuccore/AM933172) | Salmonella enterica subsp. enterica serovar Enteritidis str. P125109 complete genome. | Gammaproteobacteria | putative sulfatase | [CAR31673](http://www.ncbi.nlm.nih.gov/protein/CAR31673.1) | 100.0 |
|  | | | | | | | |
|  | | | | | | | |
| ------------------ | | | | | | | |
|  | | | | | | | |
|  | | | | | | | |
| **Input Sequence** | SM-Roxy-RH01221661_S26_L001_R1_001_(paired)_contig_42_35 # 32582 # 34468 # -1 # ID=42_35;partial=00;start_type=ATG;rbs_motif=GGAG/GAGG;rbs_spacer=5-10bp;gc_cont=0.579 | | | | | | |
|  | PROJECT ID | ACCESSION ID | ORGANISMS | CLASS | PROTEIN FUNCTION | PROTEIN ID | %IDENTITY |
| **Matched Family** | [30687](http://www.ncbi.nlm.nih.gov/bioproject?db=bioproject&cmd=ShowDetailView&TermToSearch=30687) | [AM933172](http://www.ncbi.nlm.nih.gov/nuccore/AM933172) | Salmonella enterica subsp. enterica serovar Enteritidis str. P125109 complete genome. | Gammaproteobacteria | membrane protein, suppressor for copper-sensitivity B precursor | [CAR32560](http://www.ncbi.nlm.nih.gov/protein/CAR32560.1) | 100.0 |
|  | | | | | | | |
|  | | | | | | | |
| ------------------ | | | | | | | |
|  | | | | | | | |
|  | | | | | | | |
| **Input Sequence** | SM-Roxy-RH01221661_S26_L001_R1_001_(paired)_contig_22_178 # 172848 # 174719 # 1 # ID=22_178;partial=00;start_type=ATG;rbs_motif=GGA/GAG/AGG;rbs_spacer=5-10bp;gc_cont=0.521 | | | | | | |
|  | PROJECT ID | ACCESSION ID | ORGANISMS | CLASS | PROTEIN FUNCTION | PROTEIN ID | %IDENTITY |
| **Matched Family** | [30687](http://www.ncbi.nlm.nih.gov/bioproject?db=bioproject&cmd=ShowDetailView&TermToSearch=30687) | [AM933172](http://www.ncbi.nlm.nih.gov/nuccore/AM933172) | Salmonella enterica subsp. enterica serovar Enteritidis str. P125109 complete genome. | Gammaproteobacteria | penicillin-binding protein | [CAR32676](http://www.ncbi.nlm.nih.gov/protein/CAR32676.1) | 100.0 |
|  | | | | | | | |
|  | | | | | | | |
| ------------------ | | | | | | | |
|  | | | | | | | |
|  | | | | | | | |
| **Input Sequence** | SM-Roxy-RH01221661_S26_L001_R1_001_(paired)_contig_21_126 # 109955 # 111787 # 1 # ID=21_126;partial=00;start_type=ATG;rbs_motif=AGGA;rbs_spacer=5-10bp;gc_cont=0.603 | | | | | | |
|  | PROJECT ID | ACCESSION ID | ORGANISMS | CLASS | PROTEIN FUNCTION | PROTEIN ID | %IDENTITY |
| **Matched Family** | [30687](http://www.ncbi.nlm.nih.gov/bioproject?db=bioproject&cmd=ShowDetailView&TermToSearch=30687) | [AM933172](http://www.ncbi.nlm.nih.gov/nuccore/AM933172) | Salmonella enterica subsp. enterica serovar Enteritidis str. P125109 complete genome. | Gammaproteobacteria | propanediol utilization protein | [CAR33621](http://www.ncbi.nlm.nih.gov/protein/CAR33621.1) | 100.0 |
|  | | | | | | | |
|  | | | | | | | |
| ------------------ | | | | | | | |
|  | | | | | | | |
|  | | | | | | | |
| **Input Sequence** | SM-Roxy-RH01221661_S26_L001_R1_001_(paired)_contig_11_343 # 358023 # 359849 # 1 # ID=11_343;partial=00;start_type=ATG;rbs_motif=GGA/GAG/AGG;rbs_spacer=5-10bp;gc_cont=0.613 | | | | | | |
|  | PROJECT ID | ACCESSION ID | ORGANISMS | CLASS | PROTEIN FUNCTION | PROTEIN ID | %IDENTITY |
| **Matched Family** | [30687](http://www.ncbi.nlm.nih.gov/bioproject?db=bioproject&cmd=ShowDetailView&TermToSearch=30687) | [AM933172](http://www.ncbi.nlm.nih.gov/nuccore/AM933172) | Salmonella enterica subsp. enterica serovar Enteritidis str. P125109 complete genome. | Gammaproteobacteria | formate hydrogenlyase subunit 3 | [CAR34271](http://www.ncbi.nlm.nih.gov/protein/CAR34271.1) | 100.0 |
|  | | | | | | | |
|  | | | | | | | |
| ------------------ | | | | | | | |
|  | | | | | | | |
|  | | | | | | | |
| **Input Sequence** | SM-Roxy-RH01221661_S26_L001_R1_001_(paired)_contig_2_76 # 86231 # 88048 # 1 # ID=2_76;partial=00;start_type=ATG;rbs_motif=GGAGG;rbs_spacer=5-10bp;gc_cont=0.577 | | | | | | |
|  | PROJECT ID | ACCESSION ID | ORGANISMS | CLASS | PROTEIN FUNCTION | PROTEIN ID | %IDENTITY |
| **Matched Family** | [30687](http://www.ncbi.nlm.nih.gov/bioproject?db=bioproject&cmd=ShowDetailView&TermToSearch=30687) | [AM933172](http://www.ncbi.nlm.nih.gov/nuccore/AM933172) | Salmonella enterica subsp. enterica serovar Enteritidis str. P125109 complete genome. | Gammaproteobacteria | maltodextrin glucosidase | [CAR31970](http://www.ncbi.nlm.nih.gov/protein/CAR31970.1) | 100.0 |
|  | | | | | | | |
|  | | | | | | | |
| ------------------ | | | | | | | |
|  | | | | | | | |
|  | | | | | | | |
| **Input Sequence** | SM-Roxy-RH01221661_S26_L001_R1_001_(paired)_contig_20_7 # 7287 # 9077 # -1 # ID=20_7;partial=00;start_type=TTG;rbs_motif=GGA/GAG/AGG;rbs_spacer=5-10bp;gc_cont=0.556 | | | | | | |
|  | PROJECT ID | ACCESSION ID | ORGANISMS | CLASS | PROTEIN FUNCTION | PROTEIN ID | %IDENTITY |
| **Matched Family** | [20045](http://www.ncbi.nlm.nih.gov/bioproject?db=bioproject&cmd=ShowDetailView&TermToSearch=20045) | [CP001120](http://www.ncbi.nlm.nih.gov/nuccore/CP001120) | Salmonella enterica subsp. enterica serovar Heidelberg str. SL476, complete genome. | Gammaproteobacteria | HTH-type transcriptional regulator SgrR | [ACF69018](http://www.ncbi.nlm.nih.gov/protein/ACF69018.1) | 100.0 |
|  | | | | | | | |
|  | | | | | | | |
| ------------------ | | | | | | | |
|  | | | | | | | |
|  | | | | | | | |
| **Input Sequence** | SM-Roxy-RH01221661_S26_L001_R1_001_(paired)_contig_11_309 # 325497 # 327278 # 1 # ID=11_309;partial=00;start_type=ATG;rbs_motif=AGGA;rbs_spacer=5-10bp;gc_cont=0.523 | | | | | | |
|  | PROJECT ID | ACCESSION ID | ORGANISMS | CLASS | PROTEIN FUNCTION | PROTEIN ID | %IDENTITY |
| **Matched Family** | [19467](http://www.ncbi.nlm.nih.gov/bioproject?db=bioproject&cmd=ShowDetailView&TermToSearch=19467) | [CP001144](http://www.ncbi.nlm.nih.gov/nuccore/CP001144) | Salmonella enterica subsp. enterica serovar Dublin str. C | Gammaproteobacteria | 2021853, complete genome. | [ACH74865](http://www.ncbi.nlm.nih.gov/protein/ACH74865.1) | 100.0 |
|  | | | | | | | |
|  | | | | | | | |
| ------------------ | | | | | | | |
|  | | | | | | | |
|  | | | | | | | |
| **Input Sequence** | SM-Roxy-RH01221661_S26_L001_R1_001_(paired)_contig_12_254 # 256219 # 257997 # 1 # ID=12_254;partial=00;start_type=GTG;rbs_motif=GGA/GAG/AGG;rbs_spacer=5-10bp;gc_cont=0.567 | | | | | | |
|  | PROJECT ID | ACCESSION ID | ORGANISMS | CLASS | PROTEIN FUNCTION | PROTEIN ID | %IDENTITY |
| **Matched Family** | [30687](http://www.ncbi.nlm.nih.gov/bioproject?db=bioproject&cmd=ShowDetailView&TermToSearch=30687) | [AM933172](http://www.ncbi.nlm.nih.gov/nuccore/AM933172) | Salmonella enterica subsp. enterica serovar Enteritidis str. P125109 complete genome. | Gammaproteobacteria | putative histidine kinase, two component regulatory protein | [CAR33241](http://www.ncbi.nlm.nih.gov/protein/CAR33241.1) | 100.0 |
|  | | | | | | | |
|  | | | | | | | |
| ------------------ | | | | | | | |
|  | | | | | | | |
|  | | | | | | | |
| **Input Sequence** | SM-Roxy-RH01221661_S26_L001_R1_001_(paired)_contig_11_215 # 222318 # 224093 # -1 # ID=11_215;partial=00;start_type=ATG;rbs_motif=GGAG/GAGG;rbs_spacer=5-10bp;gc_cont=0.572 | | | | | | |
|  | PROJECT ID | ACCESSION ID | ORGANISMS | CLASS | PROTEIN FUNCTION | PROTEIN ID | %IDENTITY |
| **Matched Family** | [19467](http://www.ncbi.nlm.nih.gov/bioproject?db=bioproject&cmd=ShowDetailView&TermToSearch=19467) | [CP001144](http://www.ncbi.nlm.nih.gov/nuccore/CP001144) | Salmonella enterica subsp. enterica serovar Dublin str. C | Gammaproteobacteria | 2021853, complete genome. | [ACH74330](http://www.ncbi.nlm.nih.gov/protein/ACH74330.1) | 100.0 |
|  | | | | | | | |
|  | | | | | | | |
| ------------------ | | | | | | | |
|  | | | | | | | |
|  | | | | | | | |
| **Input Sequence** | SM-Roxy-RH01221661_S26_L001_R1_001_(paired)_contig_16_17 # 12053 # 13828 # -1 # ID=16_17;partial=00;start_type=ATG;rbs_motif=AGGAG;rbs_spacer=5-10bp;gc_cont=0.519 | | | | | | |
|  | PROJECT ID | ACCESSION ID | ORGANISMS | CLASS | PROTEIN FUNCTION | PROTEIN ID | %IDENTITY |
| **Matched Family** | [241](http://www.ncbi.nlm.nih.gov/bioproject?db=bioproject&cmd=ShowDetailView&TermToSearch=241) | [AE006471](http://www.ncbi.nlm.nih.gov/nuccore/AE006471) | Salmonella enterica subsp. enterica serovar Typhimurium str. LT2 plasmid pSLT, complete sequence. | Gammaproteobacteria | Salmonella plasmid virulence: hydrophilic protein | [AAL23530](http://www.ncbi.nlm.nih.gov/protein/AAL23530.1) | 100.0 |
|  | | | | | | | |
|  | | | | | | | |
| ------------------ | | | | | | | |
|  | | | | | | | |
|  | | | | | | | |
| **Input Sequence** | SM-Roxy-RH01221661_S26_L001_R1_001_(paired)_contig_9_16 # 13375 # 15138 # -1 # ID=9_16;partial=00;start_type=ATG;rbs_motif=AGGAG;rbs_spacer=5-10bp;gc_cont=0.555 | | | | | | |
|  | PROJECT ID | ACCESSION ID | ORGANISMS | CLASS | PROTEIN FUNCTION | PROTEIN ID | %IDENTITY |
| **Matched Family** | [19467](http://www.ncbi.nlm.nih.gov/bioproject?db=bioproject&cmd=ShowDetailView&TermToSearch=19467) | [CP001144](http://www.ncbi.nlm.nih.gov/nuccore/CP001144) | Salmonella enterica subsp. enterica serovar Dublin str. C | Gammaproteobacteria | 2021853, complete genome. | [ACH74525](http://www.ncbi.nlm.nih.gov/protein/ACH74525.1) | 100.0 |
|  | | | | | | | |
|  | | | | | | | |
| ------------------ | | | | | | | |
|  | | | | | | | |
|  | | | | | | | |
| **Input Sequence** | SM-Roxy-RH01221661_S26_L001_R1_001_(paired)_contig_4_17 # 15255 # 16946 # 1 # ID=4_17;partial=00;start_type=ATG;rbs_motif=GGA/GAG/AGG;rbs_spacer=5-10bp;gc_cont=0.516 | | | | | | |
|  | PROJECT ID | ACCESSION ID | ORGANISMS | CLASS | PROTEIN FUNCTION | PROTEIN ID | %IDENTITY |
| **Matched Family** | [9618](http://www.ncbi.nlm.nih.gov/bioproject?db=bioproject&cmd=ShowDetailView&TermToSearch=9618) | [AE017220](http://www.ncbi.nlm.nih.gov/nuccore/AE017220) | Salmonella enterica subsp. enterica serovar Choleraesuis str. SC-B67, complete genome. | Gammaproteobacteria | putative membrane-associated, metal-dependent hydrolase | [AAX67473](http://www.ncbi.nlm.nih.gov/protein/AAX67473.1) | 100.0 |
|  | | | | | | | |
|  | | | | | | | |
| ------------------ | | | | | | | |
|  | | | | | | | |
|  | | | | | | | |
| **Input Sequence** | SM-Roxy-RH01221661_S26_L001_R1_001_(paired)_contig_11_69 # 70656 # 72395 # 1 # ID=11_69;partial=00;start_type=GTG;rbs_motif=GGAG/GAGG;rbs_spacer=5-10bp;gc_cont=0.482 | | | | | | |
|  | PROJECT ID | ACCESSION ID | ORGANISMS | CLASS | PROTEIN FUNCTION | PROTEIN ID | %IDENTITY |
| **Matched Family** | [30687](http://www.ncbi.nlm.nih.gov/bioproject?db=bioproject&cmd=ShowDetailView&TermToSearch=30687) | [AM933172](http://www.ncbi.nlm.nih.gov/nuccore/AM933172) | Salmonella enterica subsp. enterica serovar Enteritidis str. P125109 complete genome. | Gammaproteobacteria | arylsulfatase | [CAR34541](http://www.ncbi.nlm.nih.gov/protein/CAR34541.1) | 100.0 |
|  | | | | | | | |
|  | | | | | | | |
| ------------------ | | | | | | | |
|  | | | | | | | |
|  | | | | | | | |
| **Input Sequence** | SM-Roxy-RH01221661_S26_L001_R1_001_(paired)_contig_21_93 # 77777 # 79507 # 1 # ID=21_93;partial=00;start_type=ATG;rbs_motif=AGGA/GGAG/GAGG;rbs_spacer=11-12bp;gc_cont=0.464 | | | | | | |
|  | PROJECT ID | ACCESSION ID | ORGANISMS | CLASS | PROTEIN FUNCTION | PROTEIN ID | %IDENTITY |
| **Matched Family** | [30687](http://www.ncbi.nlm.nih.gov/bioproject?db=bioproject&cmd=ShowDetailView&TermToSearch=30687) | [AM933172](http://www.ncbi.nlm.nih.gov/nuccore/AM933172) | Salmonella enterica subsp. enterica serovar Enteritidis str. P125109 complete genome. | Gammaproteobacteria | putative exported protein | [CAR33586](http://www.ncbi.nlm.nih.gov/protein/CAR33586.1) | 100.0 |
|  | | | | | | | |
|  | | | | | | | |
| ------------------ | | | | | | | |
|  | | | | | | | |
|  | | | | | | | |
| **Input Sequence** | SM-Roxy-RH01221661_S26_L001_R1_001_(paired)_contig_42_147 # 164447 # 166168 # 1 # ID=42_147;partial=00;start_type=ATG;rbs_motif=AGGAG;rbs_spacer=5-10bp;gc_cont=0.569 | | | | | | |
|  | PROJECT ID | ACCESSION ID | ORGANISMS | CLASS | PROTEIN FUNCTION | PROTEIN ID | %IDENTITY |
| **Matched Family** | [30687](http://www.ncbi.nlm.nih.gov/bioproject?db=bioproject&cmd=ShowDetailView&TermToSearch=30687) | [AM933172](http://www.ncbi.nlm.nih.gov/nuccore/AM933172) | Salmonella enterica subsp. enterica serovar Enteritidis str. P125109 complete genome. | Gammaproteobacteria | transport ATP-binding protein CydC | [CAR32444](http://www.ncbi.nlm.nih.gov/protein/CAR32444.1) | 100.0 |
|  | | | | | | | |
|  | | | | | | | |
| ------------------ | | | | | | | |
|  | | | | | | | |
|  | | | | | | | |
| **Input Sequence** | SM-Roxy-RH01221661_S26_L001_R1_001_(paired)_contig_35_15 # 14153 # 15871 # 1 # ID=35_15;partial=00;start_type=ATG;rbs_motif=AGGAG;rbs_spacer=5-10bp;gc_cont=0.464 | | | | | | |
|  | PROJECT ID | ACCESSION ID | ORGANISMS | CLASS | PROTEIN FUNCTION | PROTEIN ID | %IDENTITY |
| **Matched Family** | [19467](http://www.ncbi.nlm.nih.gov/bioproject?db=bioproject&cmd=ShowDetailView&TermToSearch=19467) | [CP001144](http://www.ncbi.nlm.nih.gov/nuccore/CP001144) | Salmonella enterica subsp. enterica serovar Dublin str. C | Gammaproteobacteria | 2021853, complete genome. | [ACH74384](http://www.ncbi.nlm.nih.gov/protein/ACH74384.1) | 100.0 |
|  | | | | | | | |
|  | | | | | | | |
| ------------------ | | | | | | | |
|  | | | | | | | |
|  | | | | | | | |
| **Input Sequence** | SM-Roxy-RH01221661_S26_L001_R1_001_(paired)_contig_35_21 # 23123 # 24838 # 1 # ID=35_21;partial=00;start_type=ATG;rbs_motif=GGAG/GAGG;rbs_spacer=5-10bp;gc_cont=0.499 | | | | | | |
|  | PROJECT ID | ACCESSION ID | ORGANISMS | CLASS | PROTEIN FUNCTION | PROTEIN ID | %IDENTITY |
| **Matched Family** | [19467](http://www.ncbi.nlm.nih.gov/bioproject?db=bioproject&cmd=ShowDetailView&TermToSearch=19467) | [CP001144](http://www.ncbi.nlm.nih.gov/nuccore/CP001144) | Salmonella enterica subsp. enterica serovar Dublin str. C | Gammaproteobacteria | 2021853, complete genome. | [ACH76026](http://www.ncbi.nlm.nih.gov/protein/ACH76026.1) | 100.0 |
|  | | | | | | | |
|  | | | | | | | |
| ------------------ | | | | | | | |
|  | | | | | | | |
|  | | | | | | | |
| **Input Sequence** | SM-Roxy-RH01221661_S26_L001_R1_001_(paired)_contig_4_120 # 140974 # 142689 # -1 # ID=4_120;partial=00;start_type=ATG;rbs_motif=AGGA;rbs_spacer=5-10bp;gc_cont=0.537 | | | | | | |
|  | PROJECT ID | ACCESSION ID | ORGANISMS | CLASS | PROTEIN FUNCTION | PROTEIN ID | %IDENTITY |
| **Matched Family** | [30687](http://www.ncbi.nlm.nih.gov/bioproject?db=bioproject&cmd=ShowDetailView&TermToSearch=30687) | [AM933172](http://www.ncbi.nlm.nih.gov/nuccore/AM933172) | Salmonella enterica subsp. enterica serovar Enteritidis str. P125109 complete genome. | Gammaproteobacteria | putative dehydratase | [CAR34931](http://www.ncbi.nlm.nih.gov/protein/CAR34931.1) | 100.0 |
|  | | | | | | | |
|  | | | | | | | |
| ------------------ | | | | | | | |
|  | | | | | | | |
|  | | | | | | | |
| **Input Sequence** | SM-Roxy-RH01221661_S26_L001_R1_001_(paired)_contig_68_21 # 16111 # 17823 # -1 # ID=68_21;partial=00;start_type=GTG;rbs_motif=AGGAG;rbs_spacer=5-10bp;gc_cont=0.538 | | | | | | |
|  | PROJECT ID | ACCESSION ID | ORGANISMS | CLASS | PROTEIN FUNCTION | PROTEIN ID | %IDENTITY |
| **Matched Family** | [30687](http://www.ncbi.nlm.nih.gov/bioproject?db=bioproject&cmd=ShowDetailView&TermToSearch=30687) | [AM933172](http://www.ncbi.nlm.nih.gov/nuccore/AM933172) | Salmonella enterica subsp. enterica serovar Enteritidis str. P125109 complete genome. | Gammaproteobacteria | putative membrane protein | [CAR32607](http://www.ncbi.nlm.nih.gov/protein/CAR32607.1) | 100.0 |
|  | | | | | | | |
|  | | | | | | | |
| ------------------ | | | | | | | |
|  | | | | | | | |
|  | | | | | | | |
| **Input Sequence** | SM-Roxy-RH01221661_S26_L001_R1_001_(paired)_contig_4_151 # 181591 # 183300 # 1 # ID=4_151;partial=00;start_type=ATG;rbs_motif=GGA/GAG/AGG;rbs_spacer=5-10bp;gc_cont=0.549 | | | | | | |
|  | PROJECT ID | ACCESSION ID | ORGANISMS | CLASS | PROTEIN FUNCTION | PROTEIN ID | %IDENTITY |
| **Matched Family** | [30687](http://www.ncbi.nlm.nih.gov/bioproject?db=bioproject&cmd=ShowDetailView&TermToSearch=30687) | [AM933172](http://www.ncbi.nlm.nih.gov/nuccore/AM933172) | Salmonella enterica subsp. enterica serovar Enteritidis str. P125109 complete genome. | Gammaproteobacteria | putative membrane protein | [CAR34900](http://www.ncbi.nlm.nih.gov/protein/CAR34900.1) | 100.0 |
|  | | | | | | | |
|  | | | | | | | |
| ------------------ | | | | | | | |
|  | | | | | | | |
|  | | | | | | | |
| **Input Sequence** | SM-Roxy-RH01221661_S26_L001_R1_001_(paired)_contig_42_96 # 96126 # 97835 # -1 # ID=42_96;partial=00;start_type=ATG;rbs_motif=GGxGG;rbs_spacer=5-10bp;gc_cont=0.494 | | | | | | |
|  | PROJECT ID | ACCESSION ID | ORGANISMS | CLASS | PROTEIN FUNCTION | PROTEIN ID | %IDENTITY |
| **Matched Family** | [30687](http://www.ncbi.nlm.nih.gov/bioproject?db=bioproject&cmd=ShowDetailView&TermToSearch=30687) | [AM933172](http://www.ncbi.nlm.nih.gov/nuccore/AM933172) | Salmonella enterica subsp. enterica serovar Enteritidis str. P125109 complete genome. | Gammaproteobacteria | Chimeric prophage tail protein (the product of a deletion event) | [CAR32496](http://www.ncbi.nlm.nih.gov/protein/CAR32496.1) | 100.0 |
|  | | | | | | | |
|  | | | | | | | |
| ------------------ | | | | | | | |
|  | | | | | | | |
|  | | | | | | | |
| **Input Sequence** | SM-Roxy-RH01221661_S26_L001_R1_001_(paired)_contig_25_74 # 64375 # 66075 # -1 # ID=25_74;partial=00;start_type=ATG;rbs_motif=AGGA;rbs_spacer=5-10bp;gc_cont=0.573 | | | | | | |
|  | PROJECT ID | ACCESSION ID | ORGANISMS | CLASS | PROTEIN FUNCTION | PROTEIN ID | %IDENTITY |
| **Matched Family** | [30687](http://www.ncbi.nlm.nih.gov/bioproject?db=bioproject&cmd=ShowDetailView&TermToSearch=30687) | [AM933172](http://www.ncbi.nlm.nih.gov/nuccore/AM933172) | Salmonella enterica subsp. enterica serovar Enteritidis str. P125109 complete genome. | Gammaproteobacteria | conserved hypothetical protein | [CAR33157](http://www.ncbi.nlm.nih.gov/protein/CAR33157.1) | 100.0 |
|  | | | | | | | |
|  | | | | | | | |
| ------------------ | | | | | | | |
|  | | | | | | | |
|  | | | | | | | |
| **Input Sequence** | SM-Roxy-RH01221661_S26_L001_R1_001_(paired)_contig_24_123 # 129240 # 130940 # 1 # ID=24_123;partial=00;start_type=ATG;rbs_motif=None;rbs_spacer=None;gc_cont=0.563 | | | | | | |
|  | PROJECT ID | ACCESSION ID | ORGANISMS | CLASS | PROTEIN FUNCTION | PROTEIN ID | %IDENTITY |
| **Matched Family** | [20045](http://www.ncbi.nlm.nih.gov/bioproject?db=bioproject&cmd=ShowDetailView&TermToSearch=20045) | [CP001120](http://www.ncbi.nlm.nih.gov/nuccore/CP001120) | Salmonella enterica subsp. enterica serovar Heidelberg str. SL476, complete genome. | Gammaproteobacteria | bacterial extracellular solute-binding protein, family 5 | [ACF70274](http://www.ncbi.nlm.nih.gov/protein/ACF70274.1) | 100.0 |
|  | | | | | | | |
|  | | | | | | | |
| ------------------ | | | | | | | |
|  | | | | | | | |
|  | | | | | | | |
| **Input Sequence** | SM-Roxy-RH01221661_S26_L001_R1_001_(paired)_contig_11_296 # 313107 # 314795 # 1 # ID=11_296;partial=00;start_type=ATG;rbs_motif=None;rbs_spacer=None;gc_cont=0.465 | | | | | | |
|  | PROJECT ID | ACCESSION ID | ORGANISMS | CLASS | PROTEIN FUNCTION | PROTEIN ID | %IDENTITY |
| **Matched Family** | [241](http://www.ncbi.nlm.nih.gov/bioproject?db=bioproject&cmd=ShowDetailView&TermToSearch=241) | [AE006468](http://www.ncbi.nlm.nih.gov/nuccore/AE006468) | Salmonella enterica subsp. enterica serovar Typhimurium str. LT2, complete genome. | Gammaproteobacteria | outer membrane invasion protein | [AAL21778](http://www.ncbi.nlm.nih.gov/protein/AAL21778.1) | 100.0 |
|  | | | | | | | |
|  | | | | | | | |
| ------------------ | | | | | | | |
|  | | | | | | | |
|  | | | | | | | |
| **Input Sequence** | SM-Roxy-RH01221661_S26_L001_R1_001_(paired)_contig_22_22 # 25604 # 27262 # 1 # ID=22_22;partial=00;start_type=ATG;rbs_motif=None;rbs_spacer=None;gc_cont=0.569 | | | | | | |
|  | PROJECT ID | ACCESSION ID | ORGANISMS | CLASS | PROTEIN FUNCTION | PROTEIN ID | %IDENTITY |
| **Matched Family** | [27803](http://www.ncbi.nlm.nih.gov/bioproject?db=bioproject&cmd=ShowDetailView&TermToSearch=27803) | [CP000886](http://www.ncbi.nlm.nih.gov/nuccore/CP000886) | Salmonella enterica subsp. enterica serovar Paratyphi B str. SPB7, complete genome. | Gammaproteobacteria | hypothetical protein | [ABX66852](http://www.ncbi.nlm.nih.gov/protein/ABX66852.1) | 100.0 |
|  | | | | | | | |
|  | | | | | | | |
| ------------------ | | | | | | | |
|  | | | | | | | |
|  | | | | | | | |
| **Input Sequence** | SM-Roxy-RH01221661_S26_L001_R1_001_(paired)_contig_42_57 # 55119 # 56804 # 1 # ID=42_57;partial=00;start_type=ATG;rbs_motif=4Base/6BMM;rbs_spacer=13-15bp;gc_cont=0.469 | | | | | | |
|  | PROJECT ID | ACCESSION ID | ORGANISMS | CLASS | PROTEIN FUNCTION | PROTEIN ID | %IDENTITY |
| **Matched Family** | [30687](http://www.ncbi.nlm.nih.gov/bioproject?db=bioproject&cmd=ShowDetailView&TermToSearch=30687) | [AM933172](http://www.ncbi.nlm.nih.gov/nuccore/AM933172) | Salmonella enterica subsp. enterica serovar Enteritidis str. P125109 complete genome. | Gammaproteobacteria | cell invasion protein | [CAR32537](http://www.ncbi.nlm.nih.gov/protein/CAR32537.1) | 100.0 |
|  | | | | | | | |
|  | | | | | | | |
| ------------------ | | | | | | | |
|  | | | | | | | |
|  | | | | | | | |
| **Input Sequence** | SM-Roxy-RH01221661_S26_L001_R1_001_(paired)_contig_4_28 # 29578 # 31257 # -1 # ID=4_28;partial=00;start_type=ATG;rbs_motif=GGA/GAG/AGG;rbs_spacer=5-10bp;gc_cont=0.554 | | | | | | |
|  | PROJECT ID | ACCESSION ID | ORGANISMS | CLASS | PROTEIN FUNCTION | PROTEIN ID | %IDENTITY |
| **Matched Family** | [19467](http://www.ncbi.nlm.nih.gov/bioproject?db=bioproject&cmd=ShowDetailView&TermToSearch=19467) | [CP001144](http://www.ncbi.nlm.nih.gov/nuccore/CP001144) | Salmonella enterica subsp. enterica serovar Dublin str. C | Gammaproteobacteria | 2021853, complete genome. | [ACH77944](http://www.ncbi.nlm.nih.gov/protein/ACH77944.1) | 100.0 |
|  | | | | | | | |
|  | | | | | | | |
| ------------------ | | | | | | | |
|  | | | | | | | |
|  | | | | | | | |
| **Input Sequence** | SM-Roxy-RH01221661_S26_L001_R1_001_(paired)_contig_24_89 # 91283 # 92959 # 1 # ID=24_89;partial=00;start_type=ATG;rbs_motif=AGGAG;rbs_spacer=5-10bp;gc_cont=0.572 | | | | | | |
|  | PROJECT ID | ACCESSION ID | ORGANISMS | CLASS | PROTEIN FUNCTION | PROTEIN ID | %IDENTITY |
| **Matched Family** | [236](http://www.ncbi.nlm.nih.gov/bioproject?db=bioproject&cmd=ShowDetailView&TermToSearch=236) | [XXX](http://www.ncbi.nlm.nih.gov/nuccore/XXX) | Salmonella enterica subsp. enterica serovar Typhi str. CT18 | Gammaproteobacteria | XXX |  | 100.0 |
|  | | | | | | | |
|  | | | | | | | |
| ------------------ | | | | | | | |
|  | | | | | | | |
|  | | | | | | | |
| **Input Sequence** | SM-Roxy-RH01221661_S26_L001_R1_001_(paired)_contig_11_318 # 334875 # 336536 # -1 # ID=11_318;partial=00;start_type=ATG;rbs_motif=3Base/5BMM;rbs_spacer=13-15bp;gc_cont=0.431 | | | | | | |
|  | PROJECT ID | ACCESSION ID | ORGANISMS | CLASS | PROTEIN FUNCTION | PROTEIN ID | %IDENTITY |
| **Matched Family** | [19467](http://www.ncbi.nlm.nih.gov/bioproject?db=bioproject&cmd=ShowDetailView&TermToSearch=19467) | [CP001144](http://www.ncbi.nlm.nih.gov/nuccore/CP001144) | Salmonella enterica subsp. enterica serovar Dublin str. C | Gammaproteobacteria | 2021853, complete genome. | [ACH77691](http://www.ncbi.nlm.nih.gov/protein/ACH77691.1) | 100.0 |
|  | | | | | | | |
|  | | | | | | | |
| ------------------ | | | | | | | |
|  | | | | | | | |
|  | | | | | | | |
| **Input Sequence** | SM-Roxy-RH01221661_S26_L001_R1_001_(paired)_contig_24_51 # 50291 # 51955 # -1 # ID=24_51;partial=00;start_type=ATG;rbs_motif=AGGAG;rbs_spacer=5-10bp;gc_cont=0.575 | | | | | | |
|  | PROJECT ID | ACCESSION ID | ORGANISMS | CLASS | PROTEIN FUNCTION | PROTEIN ID | %IDENTITY |
| **Matched Family** | [30687](http://www.ncbi.nlm.nih.gov/bioproject?db=bioproject&cmd=ShowDetailView&TermToSearch=30687) | [AM933172](http://www.ncbi.nlm.nih.gov/nuccore/AM933172) | Salmonella enterica subsp. enterica serovar Enteritidis str. P125109 complete genome. | Gammaproteobacteria | FdrA protein | [CAR32095](http://www.ncbi.nlm.nih.gov/protein/CAR32095.1) | 100.0 |
|  | | | | | | | |
|  | | | | | | | |
| ------------------ | | | | | | | |
|  | | | | | | | |
|  | | | | | | | |
| **Input Sequence** | SM-Roxy-RH01221661_S26_L001_R1_001_(paired)_contig_21_123 # 107060 # 108724 # 1 # ID=21_123;partial=00;start_type=ATG;rbs_motif=GGAG/GAGG;rbs_spacer=5-10bp;gc_cont=0.566 | | | | | | |
|  | PROJECT ID | ACCESSION ID | ORGANISMS | CLASS | PROTEIN FUNCTION | PROTEIN ID | %IDENTITY |
| **Matched Family** | [13086](http://www.ncbi.nlm.nih.gov/bioproject?db=bioproject&cmd=ShowDetailView&TermToSearch=13086) | [CP000026](http://www.ncbi.nlm.nih.gov/nuccore/CP000026) | Salmonella enterica subsp. enterica serovar Paratyphi A str. ATCC 9150, complete genome. | Gammaproteobacteria | glycerol dehydratase large subunit | [AAV76821](http://www.ncbi.nlm.nih.gov/protein/AAV76821.1) | 100.0 |
|  | | | | | | | |
|  | | | | | | | |
| ------------------ | | | | | | | |
|  | | | | | | | |
|  | | | | | | | |
| **Input Sequence** | SM-Roxy-RH01221661_S26_L001_R1_001_(paired)_contig_24_25 # 26897 # 28537 # 1 # ID=24_25;partial=00;start_type=ATG;rbs_motif=GGA/GAG/AGG;rbs_spacer=5-10bp;gc_cont=0.366 | | | | | | |
|  | PROJECT ID | ACCESSION ID | ORGANISMS | CLASS | PROTEIN FUNCTION | PROTEIN ID | %IDENTITY |
| **Matched Family** | [30687](http://www.ncbi.nlm.nih.gov/bioproject?db=bioproject&cmd=ShowDetailView&TermToSearch=30687) | [AM933172](http://www.ncbi.nlm.nih.gov/nuccore/AM933172) | Salmonella enterica subsp. enterica serovar Enteritidis str. P125109 complete genome. | Gammaproteobacteria | putative membrane protein | [CAR32121](http://www.ncbi.nlm.nih.gov/protein/CAR32121.1) | 100.0 |
|  | | | | | | | |
|  | | | | | | | |
| ------------------ | | | | | | | |
|  | | | | | | | |
|  | | | | | | | |
| **Input Sequence** | SM-Roxy-RH01221661_S26_L001_R1_001_(paired)_contig_12_118 # 114491 # 116125 # 1 # ID=12_118;partial=00;start_type=ATG;rbs_motif=4Base/6BMM;rbs_spacer=13-15bp;gc_cont=0.543 | | | | | | |
|  | PROJECT ID | ACCESSION ID | ORGANISMS | CLASS | PROTEIN FUNCTION | PROTEIN ID | %IDENTITY |
| **Matched Family** | [19467](http://www.ncbi.nlm.nih.gov/bioproject?db=bioproject&cmd=ShowDetailView&TermToSearch=19467) | [CP001144](http://www.ncbi.nlm.nih.gov/nuccore/CP001144) | Salmonella enterica subsp. enterica serovar Dublin str. C | Gammaproteobacteria | 2021853, complete genome. | [ACH75575](http://www.ncbi.nlm.nih.gov/protein/ACH75575.1) | 100.0 |
|  | | | | | | | |
|  | | | | | | | |
| ------------------ | | | | | | | |
|  | | | | | | | |
|  | | | | | | | |
| **Input Sequence** | SM-Roxy-RH01221661_S26_L001_R1_001_(paired)_contig_50_19 # 20777 # 22366 # 1 # ID=50_19;partial=00;start_type=ATG;rbs_motif=GGAG/GAGG;rbs_spacer=5-10bp;gc_cont=0.542 | | | | | | |
|  | PROJECT ID | ACCESSION ID | ORGANISMS | CLASS | PROTEIN FUNCTION | PROTEIN ID | %IDENTITY |
| **Matched Family** | [30687](http://www.ncbi.nlm.nih.gov/bioproject?db=bioproject&cmd=ShowDetailView&TermToSearch=30687) | [AM933172](http://www.ncbi.nlm.nih.gov/nuccore/AM933172) | Salmonella enterica subsp. enterica serovar Enteritidis str. P125109 complete genome. | Gammaproteobacteria | putative membrane protein | [CAR35549](http://www.ncbi.nlm.nih.gov/protein/CAR35549.1) | 100.0 |
|  | | | | | | | |
|  | | | | | | | |
| ------------------ | | | | | | | |
|  | | | | | | | |
|  | | | | | | | |
| **Input Sequence** | SM-Roxy-RH01221661_S26_L001_R1_001_(paired)_contig_23_1 # 124 # 1749 # -1 # ID=23_1;partial=00;start_type=ATG;rbs_motif=AGGAG;rbs_spacer=5-10bp;gc_cont=0.551 | | | | | | |
|  | PROJECT ID | ACCESSION ID | ORGANISMS | CLASS | PROTEIN FUNCTION | PROTEIN ID | %IDENTITY |
| **Matched Family** | [30687](http://www.ncbi.nlm.nih.gov/bioproject?db=bioproject&cmd=ShowDetailView&TermToSearch=30687) | [AM933172](http://www.ncbi.nlm.nih.gov/nuccore/AM933172) | Salmonella enterica subsp. enterica serovar Enteritidis str. P125109 complete genome. | Gammaproteobacteria | conserved hypothetical protein | [CAR35814](http://www.ncbi.nlm.nih.gov/protein/CAR35814.1) | 100.0 |
|  | | | | | | | |
|  | | | | | | | |
| ------------------ | | | | | | | |
|  | | | | | | | |
|  | | | | | | | |
| **Input Sequence** | SM-Roxy-RH01221661_S26_L001_R1_001_(paired)_contig_9_95 # 107460 # 109082 # 1 # ID=9_95;partial=00;start_type=ATG;rbs_motif=GGAG/GAGG;rbs_spacer=5-10bp;gc_cont=0.574 | | | | | | |
|  | PROJECT ID | ACCESSION ID | ORGANISMS | CLASS | PROTEIN FUNCTION | PROTEIN ID | %IDENTITY |
| **Matched Family** | [30687](http://www.ncbi.nlm.nih.gov/bioproject?db=bioproject&cmd=ShowDetailView&TermToSearch=30687) | [AM933172](http://www.ncbi.nlm.nih.gov/nuccore/AM933172) | Salmonella enterica subsp. enterica serovar Enteritidis str. P125109 complete genome. | Gammaproteobacteria | putative exported protein | [CAR31749](http://www.ncbi.nlm.nih.gov/protein/CAR31749.1) | 100.0 |
|  | | | | | | | |
|  | | | | | | | |
| ------------------ | | | | | | | |
|  | | | | | | | |
|  | | | | | | | |
| **Input Sequence** | SM-Roxy-RH01221661_S26_L001_R1_001_(paired)_contig_11_316 # 332714 # 334321 # 1 # ID=11_316;partial=00;start_type=TTG;rbs_motif=AGGAG;rbs_spacer=5-10bp;gc_cont=0.444 | | | | | | |
|  | PROJECT ID | ACCESSION ID | ORGANISMS | CLASS | PROTEIN FUNCTION | PROTEIN ID | %IDENTITY |
| **Matched Family** | [19467](http://www.ncbi.nlm.nih.gov/bioproject?db=bioproject&cmd=ShowDetailView&TermToSearch=19467) | [CP001144](http://www.ncbi.nlm.nih.gov/nuccore/CP001144) | Salmonella enterica subsp. enterica serovar Dublin str. C | Gammaproteobacteria | 2021853, complete genome. | [ACH74607](http://www.ncbi.nlm.nih.gov/protein/ACH74607.1) | 100.0 |
|  | | | | | | | |
|  | | | | | | | |
| ------------------ | | | | | | | |
|  | | | | | | | |
|  | | | | | | | |
| **Input Sequence** | SM-Roxy-RH01221661_S26_L001_R1_001_(paired)_contig_10_49 # 54987 # 56588 # -1 # ID=10_49;partial=00;start_type=ATG;rbs_motif=GGA/GAG/AGG;rbs_spacer=5-10bp;gc_cont=0.473 | | | | | | |
|  | PROJECT ID | ACCESSION ID | ORGANISMS | CLASS | PROTEIN FUNCTION | PROTEIN ID | %IDENTITY |
| **Matched Family** | [30687](http://www.ncbi.nlm.nih.gov/bioproject?db=bioproject&cmd=ShowDetailView&TermToSearch=30687) | [AM933172](http://www.ncbi.nlm.nih.gov/nuccore/AM933172) | Salmonella enterica subsp. enterica serovar Enteritidis str. P125109 complete genome. | Gammaproteobacteria | putative exported protein | [CAR35596](http://www.ncbi.nlm.nih.gov/protein/CAR35596.1) | 100.0 |
|  | | | | | | | |
|  | | | | | | | |
| ------------------ | | | | | | | |
|  | | | | | | | |
|  | | | | | | | |
| **Input Sequence** | SM-Roxy-RH01221661_S26_L001_R1_001_(paired)_contig_44_33 # 31562 # 33157 # -1 # ID=44_33;partial=00;start_type=ATG;rbs_motif=AGGAG;rbs_spacer=5-10bp;gc_cont=0.570 | | | | | | |
|  | PROJECT ID | ACCESSION ID | ORGANISMS | CLASS | PROTEIN FUNCTION | PROTEIN ID | %IDENTITY |
| **Matched Family** | [19467](http://www.ncbi.nlm.nih.gov/bioproject?db=bioproject&cmd=ShowDetailView&TermToSearch=19467) | [CP001144](http://www.ncbi.nlm.nih.gov/nuccore/CP001144) | Salmonella enterica subsp. enterica serovar Dublin str. C | Gammaproteobacteria | 2021853, complete genome. | [ACH75894](http://www.ncbi.nlm.nih.gov/protein/ACH75894.1) | 100.0 |
|  | | | | | | | |
|  | | | | | | | |
| ------------------ | | | | | | | |
|  | | | | | | | |
|  | | | | | | | |
| **Input Sequence** | SM-Roxy-RH01221661_S26_L001_R1_001_(paired)_contig_4_48 # 58852 # 60441 # 1 # ID=4_48;partial=00;start_type=ATG;rbs_motif=GGA/GAG/AGG;rbs_spacer=5-10bp;gc_cont=0.565 | | | | | | |
|  | PROJECT ID | ACCESSION ID | ORGANISMS | CLASS | PROTEIN FUNCTION | PROTEIN ID | %IDENTITY |
| **Matched Family** | [30687](http://www.ncbi.nlm.nih.gov/bioproject?db=bioproject&cmd=ShowDetailView&TermToSearch=30687) | [AM933172](http://www.ncbi.nlm.nih.gov/nuccore/AM933172) | Salmonella enterica subsp. enterica serovar Enteritidis str. P125109 complete genome. | Gammaproteobacteria | putative membrane protein | [CAR35003](http://www.ncbi.nlm.nih.gov/protein/CAR35003.1) | 100.0 |
|  | | | | | | | |
|  | | | | | | | |
| ------------------ | | | | | | | |
|  | | | | | | | |
|  | | | | | | | |
| **Input Sequence** | SM-Roxy-RH01221661_S26_L001_R1_001_(paired)_contig_2_19 # 17082 # 18656 # 1 # ID=2_19;partial=00;start_type=TTG;rbs_motif=GGAG/GAGG;rbs_spacer=5-10bp;gc_cont=0.487 | | | | | | |
|  | PROJECT ID | ACCESSION ID | ORGANISMS | CLASS | PROTEIN FUNCTION | PROTEIN ID | %IDENTITY |
| **Matched Family** | [30687](http://www.ncbi.nlm.nih.gov/bioproject?db=bioproject&cmd=ShowDetailView&TermToSearch=30687) | [AM933172](http://www.ncbi.nlm.nih.gov/nuccore/AM933172) | Salmonella enterica subsp. enterica serovar Enteritidis str. P125109 complete genome. | Gammaproteobacteria | putative rtn protein | [CAR31912](http://www.ncbi.nlm.nih.gov/protein/CAR31912.1) | 100.0 |
|  | | | | | | | |
|  | | | | | | | |
| ------------------ | | | | | | | |
|  | | | | | | | |
|  | | | | | | | |
| **Input Sequence** | SM-Roxy-RH01221661_S26_L001_R1_001_(paired)_contig_35_16 # 15917 # 17479 # -1 # ID=35_16;partial=00;start_type=ATG;rbs_motif=GGA/GAG/AGG;rbs_spacer=5-10bp;gc_cont=0.471 | | | | | | |
|  | PROJECT ID | ACCESSION ID | ORGANISMS | CLASS | PROTEIN FUNCTION | PROTEIN ID | %IDENTITY |
| **Matched Family** | [30687](http://www.ncbi.nlm.nih.gov/bioproject?db=bioproject&cmd=ShowDetailView&TermToSearch=30687) | [AM933172](http://www.ncbi.nlm.nih.gov/nuccore/AM933172) | Salmonella enterica subsp. enterica serovar Enteritidis str. P125109 complete genome. | Gammaproteobacteria | putative secreted 5'-nucleotidase | [CAR31624](http://www.ncbi.nlm.nih.gov/protein/CAR31624.1) | 100.0 |
|  | | | | | | | |
|  | | | | | | | |
| ------------------ | | | | | | | |
|  | | | | | | | |
|  | | | | | | | |
| **Input Sequence** | SM-Roxy-RH01221661_S26_L001_R1_001_(paired)_contig_42_50 # 47747 # 49309 # 1 # ID=42_50;partial=00;start_type=ATG;rbs_motif=GGAG/GAGG;rbs_spacer=5-10bp;gc_cont=0.527 | | | | | | |
|  | PROJECT ID | ACCESSION ID | ORGANISMS | CLASS | PROTEIN FUNCTION | PROTEIN ID | %IDENTITY |
| **Matched Family** | [18747](http://www.ncbi.nlm.nih.gov/bioproject?db=bioproject&cmd=ShowDetailView&TermToSearch=18747) | [CP001113](http://www.ncbi.nlm.nih.gov/nuccore/CP001113) | Salmonella enterica subsp. enterica serovar Newport str. SL254, complete genome. | Gammaproteobacteria | 4-hydroxyphenylacetate 3-monooxygenase, oxygenase component | [ACF62934](http://www.ncbi.nlm.nih.gov/protein/ACF62934.1) | 100.0 |
|  | | | | | | | |
|  | | | | | | | |
| ------------------ | | | | | | | |
|  | | | | | | | |
|  | | | | | | | |
| **Input Sequence** | SM-Roxy-RH01221661_S26_L001_R1_001_(paired)_contig_21_7 # 6775 # 8337 # -1 # ID=21_7;partial=00;start_type=ATG;rbs_motif=AGGA;rbs_spacer=5-10bp;gc_cont=0.485 | | | | | | |
|  | PROJECT ID | ACCESSION ID | ORGANISMS | CLASS | PROTEIN FUNCTION | PROTEIN ID | %IDENTITY |
| **Matched Family** | [30687](http://www.ncbi.nlm.nih.gov/bioproject?db=bioproject&cmd=ShowDetailView&TermToSearch=30687) | [AM933172](http://www.ncbi.nlm.nih.gov/nuccore/AM933172) | Salmonella enterica subsp. enterica serovar Enteritidis str. P125109 complete genome. | Gammaproteobacteria | putative phage tail fibre protein | [CAR33503](http://www.ncbi.nlm.nih.gov/protein/CAR33503.1) | 100.0 |
|  | | | | | | | |
|  | | | | | | | |
| ------------------ | | | | | | | |
|  | | | | | | | |
|  | | | | | | | |
| **Input Sequence** | SM-Roxy-RH01221661_S26_L001_R1_001_(paired)_contig_4_30 # 31442 # 33001 # -1 # ID=4_30;partial=00;start_type=GTG;rbs_motif=GGAG/GAGG;rbs_spacer=5-10bp;gc_cont=0.529 | | | | | | |
|  | PROJECT ID | ACCESSION ID | ORGANISMS | CLASS | PROTEIN FUNCTION | PROTEIN ID | %IDENTITY |
| **Matched Family** | [19467](http://www.ncbi.nlm.nih.gov/bioproject?db=bioproject&cmd=ShowDetailView&TermToSearch=19467) | [CP001144](http://www.ncbi.nlm.nih.gov/nuccore/CP001144) | Salmonella enterica subsp. enterica serovar Dublin str. C | Gammaproteobacteria | 2021853, complete genome. | [ACH77065](http://www.ncbi.nlm.nih.gov/protein/ACH77065.1) | 100.0 |
|  | | | | | | | |
|  | | | | | | | |
| ------------------ | | | | | | | |
|  | | | | | | | |
|  | | | | | | | |
| **Input Sequence** | SM-Roxy-RH01221661_S26_L001_R1_001_(paired)_contig_21_298 # 303778 # 305334 # 1 # ID=21_298;partial=00;start_type=ATG;rbs_motif=3Base/5BMM;rbs_spacer=13-15bp;gc_cont=0.520 | | | | | | |
|  | PROJECT ID | ACCESSION ID | ORGANISMS | CLASS | PROTEIN FUNCTION | PROTEIN ID | %IDENTITY |
| **Matched Family** | [30687](http://www.ncbi.nlm.nih.gov/bioproject?db=bioproject&cmd=ShowDetailView&TermToSearch=30687) | [AM933172](http://www.ncbi.nlm.nih.gov/nuccore/AM933172) | Salmonella enterica subsp. enterica serovar Enteritidis str. P125109 complete genome. | Gammaproteobacteria | rtn protein | [CAR33793](http://www.ncbi.nlm.nih.gov/protein/CAR33793.1) | 100.0 |
|  | | | | | | | |
|  | | | | | | | |
| ------------------ | | | | | | | |
|  | | | | | | | |
|  | | | | | | | |
| **Input Sequence** | SM-Roxy-RH01221661_S26_L001_R1_001_(paired)_contig_4_123 # 145625 # 147121 # 1 # ID=4_123;partial=00;start_type=ATG;rbs_motif=GGA/GAG/AGG;rbs_spacer=5-10bp;gc_cont=0.510 | | | | | | |
|  | PROJECT ID | ACCESSION ID | ORGANISMS | CLASS | PROTEIN FUNCTION | PROTEIN ID | %IDENTITY |
| **Matched Family** | [30687](http://www.ncbi.nlm.nih.gov/bioproject?db=bioproject&cmd=ShowDetailView&TermToSearch=30687) | [AM933172](http://www.ncbi.nlm.nih.gov/nuccore/AM933172) | Salmonella enterica subsp. enterica serovar Enteritidis str. P125109 complete genome. | Gammaproteobacteria | putative membrane protein | [CAR34928](http://www.ncbi.nlm.nih.gov/protein/CAR34928.1) | 100.0 |
|  | | | | | | | |
|  | | | | | | | |
| ------------------ | | | | | | | |
|  | | | | | | | |
|  | | | | | | | |
| **Input Sequence** | SM-Roxy-RH01221661_S26_L001_R1_001_(paired)_contig_24_112 # 117242 # 118792 # 1 # ID=24_112;partial=00;start_type=GTG;rbs_motif=AGGA;rbs_spacer=5-10bp;gc_cont=0.522 | | | | | | |
|  | PROJECT ID | ACCESSION ID | ORGANISMS | CLASS | PROTEIN FUNCTION | PROTEIN ID | %IDENTITY |
| **Matched Family** | [19467](http://www.ncbi.nlm.nih.gov/bioproject?db=bioproject&cmd=ShowDetailView&TermToSearch=19467) | [CP001144](http://www.ncbi.nlm.nih.gov/nuccore/CP001144) | Salmonella enterica subsp. enterica serovar Dublin str. C | Gammaproteobacteria | 2021853, complete genome. | [ACH74515](http://www.ncbi.nlm.nih.gov/protein/ACH74515.1) | 100.0 |
|  | | | | | | | |
|  | | | | | | | |
| ------------------ | | | | | | | |
|  | | | | | | | |
|  | | | | | | | |
| **Input Sequence** | SM-Roxy-RH01221661_S26_L001_R1_001_(paired)_contig_25_239 # 244618 # 245208 # 1 # ID=25_239;partial=00;start_type=ATG;rbs_motif=None;rbs_spacer=None;gc_cont=0.415 | | | | | | |
|  | PROJECT ID | ACCESSION ID | ORGANISMS | CLASS | PROTEIN FUNCTION | PROTEIN ID | %IDENTITY |
| **Matched Family** | [18747](http://www.ncbi.nlm.nih.gov/bioproject?db=bioproject&cmd=ShowDetailView&TermToSearch=18747) | [CP001113](http://www.ncbi.nlm.nih.gov/nuccore/CP001113) | Salmonella enterica subsp. enterica serovar Newport str. SL254, complete genome. | Gammaproteobacteria | putative inner membrane protein | [ACF63691](http://www.ncbi.nlm.nih.gov/protein/ACF63691.1) | 100.0 |
|  | | | | | | | |
|  | | | | | | | |
| ------------------ | | | | | | | |
|  | | | | | | | |
|  | | | | | | | |
| **Input Sequence** | SM-Roxy-RH01221661_S26_L001_R1_001_(paired)_contig_5_85 # 81625 # 83160 # 1 # ID=5_85;partial=00;start_type=ATG;rbs_motif=GGAGG;rbs_spacer=3-4bp;gc_cont=0.516 | | | | | | |
|  | PROJECT ID | ACCESSION ID | ORGANISMS | CLASS | PROTEIN FUNCTION | PROTEIN ID | %IDENTITY |
| **Matched Family** | [13086](http://www.ncbi.nlm.nih.gov/bioproject?db=bioproject&cmd=ShowDetailView&TermToSearch=13086) | [CP000026](http://www.ncbi.nlm.nih.gov/nuccore/CP000026) | Salmonella enterica subsp. enterica serovar Paratyphi A str. ATCC 9150, complete genome. | Gammaproteobacteria | putative ABC transporter ATP-binding protein | [AAV79682](http://www.ncbi.nlm.nih.gov/protein/AAV79682.1) | 100.0 |
|  | | | | | | | |
|  | | | | | | | |
| ------------------ | | | | | | | |
|  | | | | | | | |
|  | | | | | | | |
| **Input Sequence** | SM-Roxy-RH01221661_S26_L001_R1_001_(paired)_contig_21_186 # 174290 # 175768 # -1 # ID=21_186;partial=00;start_type=ATG;rbs_motif=GGAG/GAGG;rbs_spacer=5-10bp;gc_cont=0.518 | | | | | | |
|  | PROJECT ID | ACCESSION ID | ORGANISMS | CLASS | PROTEIN FUNCTION | PROTEIN ID | %IDENTITY |
| **Matched Family** | [19467](http://www.ncbi.nlm.nih.gov/bioproject?db=bioproject&cmd=ShowDetailView&TermToSearch=19467) | [CP001144](http://www.ncbi.nlm.nih.gov/nuccore/CP001144) | Salmonella enterica subsp. enterica serovar Dublin str. C | Gammaproteobacteria | 2021853, complete genome. | [ACH76246](http://www.ncbi.nlm.nih.gov/protein/ACH76246.1) | 100.0 |
|  | | | | | | | |
|  | | | | | | | |
| ------------------ | | | | | | | |
|  | | | | | | | |
|  | | | | | | | |
| **Input Sequence** | SM-Roxy-RH01221661_S26_L001_R1_001_(paired)_contig_19_14 # 11269 # 12798 # 1 # ID=19_14;partial=00;start_type=ATG;rbs_motif=GGA/GAG/AGG;rbs_spacer=5-10bp;gc_cont=0.590 | | | | | | |
|  | PROJECT ID | ACCESSION ID | ORGANISMS | CLASS | PROTEIN FUNCTION | PROTEIN ID | %IDENTITY |
| **Matched Family** | [30687](http://www.ncbi.nlm.nih.gov/bioproject?db=bioproject&cmd=ShowDetailView&TermToSearch=30687) | [AM933172](http://www.ncbi.nlm.nih.gov/nuccore/AM933172) | Salmonella enterica subsp. enterica serovar Enteritidis str. P125109 complete genome. | Gammaproteobacteria | citrate lyase alpha chain | [CAR32178](http://www.ncbi.nlm.nih.gov/protein/CAR32178.1) | 100.0 |
|  | | | | | | | |
|  | | | | | | | |
| ------------------ | | | | | | | |
|  | | | | | | | |
|  | | | | | | | |
| **Input Sequence** | SM-Roxy-RH01221661_S26_L001_R1_001_(paired)_contig_22_1 # 467 # 1996 # -1 # ID=22_1;partial=00;start_type=ATG;rbs_motif=AGGA;rbs_spacer=5-10bp;gc_cont=0.493 | | | | | | |
|  | PROJECT ID | ACCESSION ID | ORGANISMS | CLASS | PROTEIN FUNCTION | PROTEIN ID | %IDENTITY |
| **Matched Family** | [19467](http://www.ncbi.nlm.nih.gov/bioproject?db=bioproject&cmd=ShowDetailView&TermToSearch=19467) | [CP001144](http://www.ncbi.nlm.nih.gov/nuccore/CP001144) | Salmonella enterica subsp. enterica serovar Dublin str. C | Gammaproteobacteria | 2021853, complete genome. | [ACH76829](http://www.ncbi.nlm.nih.gov/protein/ACH76829.1) | 100.0 |
|  | | | | | | | |
|  | | | | | | | |
| ------------------ | | | | | | | |
|  | | | | | | | |
|  | | | | | | | |
| **Input Sequence** | SM-Roxy-RH01221661_S26_L001_R1_001_(paired)_contig_38_23 # 23745 # 25265 # 1 # ID=38_23;partial=00;start_type=ATG;rbs_motif=GGA/GAG/AGG;rbs_spacer=5-10bp;gc_cont=0.605 | | | | | | |
|  | PROJECT ID | ACCESSION ID | ORGANISMS | CLASS | PROTEIN FUNCTION | PROTEIN ID | %IDENTITY |
| **Matched Family** | [19467](http://www.ncbi.nlm.nih.gov/bioproject?db=bioproject&cmd=ShowDetailView&TermToSearch=19467) | [CP001144](http://www.ncbi.nlm.nih.gov/nuccore/CP001144) | Salmonella enterica subsp. enterica serovar Dublin str. C | Gammaproteobacteria | 2021853, complete genome. | [ACH74277](http://www.ncbi.nlm.nih.gov/protein/ACH74277.1) | 100.0 |
|  | | | | | | | |
|  | | | | | | | |
| ------------------ | | | | | | | |
|  | | | | | | | |
|  | | | | | | | |
| **Input Sequence** | SM-Roxy-RH01221661_S26_L001_R1_001_(paired)_contig_22_220 # 215276 # 216793 # -1 # ID=22_220;partial=00;start_type=ATG;rbs_motif=AGGA;rbs_spacer=5-10bp;gc_cont=0.446 | | | | | | |
|  | PROJECT ID | ACCESSION ID | ORGANISMS | CLASS | PROTEIN FUNCTION | PROTEIN ID | %IDENTITY |
| **Matched Family** | [30687](http://www.ncbi.nlm.nih.gov/bioproject?db=bioproject&cmd=ShowDetailView&TermToSearch=30687) | [AM933172](http://www.ncbi.nlm.nih.gov/nuccore/AM933172) | Salmonella enterica subsp. enterica serovar Enteritidis str. P125109 complete genome. | Gammaproteobacteria | flagellin | [CAR32634](http://www.ncbi.nlm.nih.gov/protein/CAR32634.1) | 100.0 |
|  | | | | | | | |
|  | | | | | | | |
| ------------------ | | | | | | | |
|  | | | | | | | |
|  | | | | | | | |
| **Input Sequence** | SM-Roxy-RH01221661_S26_L001_R1_001_(paired)_contig_53_18 # 19887 # 21404 # -1 # ID=53_18;partial=00;start_type=ATG;rbs_motif=GGA/GAG/AGG;rbs_spacer=5-10bp;gc_cont=0.537 | | | | | | |
|  | PROJECT ID | ACCESSION ID | ORGANISMS | CLASS | PROTEIN FUNCTION | PROTEIN ID | %IDENTITY |
| **Matched Family** | [18747](http://www.ncbi.nlm.nih.gov/bioproject?db=bioproject&cmd=ShowDetailView&TermToSearch=18747) | [CP001113](http://www.ncbi.nlm.nih.gov/nuccore/CP001113) | Salmonella enterica subsp. enterica serovar Newport str. SL254, complete genome. | Gammaproteobacteria | L-carnitine/gamma-butyrobetaine antiporter | [ACF64613](http://www.ncbi.nlm.nih.gov/protein/ACF64613.1) | 100.0 |
|  | | | | | | | |
|  | | | | | | | |
| ------------------ | | | | | | | |
|  | | | | | | | |
|  | | | | | | | |
| **Input Sequence** | SM-Roxy-RH01221661_S26_L001_R1_001_(paired)_contig_24_143 # 151605 # 153116 # -1 # ID=24_143;partial=00;start_type=ATG;rbs_motif=AGGAG;rbs_spacer=5-10bp;gc_cont=0.425 | | | | | | |
|  | PROJECT ID | ACCESSION ID | ORGANISMS | CLASS | PROTEIN FUNCTION | PROTEIN ID | %IDENTITY |
| **Matched Family** | [30687](http://www.ncbi.nlm.nih.gov/bioproject?db=bioproject&cmd=ShowDetailView&TermToSearch=30687) | [AM933172](http://www.ncbi.nlm.nih.gov/nuccore/AM933172) | Salmonella enterica subsp. enterica serovar Enteritidis str. P125109 complete genome. | Gammaproteobacteria | putative exported protein | [CAR32005](http://www.ncbi.nlm.nih.gov/protein/CAR32005.1) | 100.0 |
|  | | | | | | | |
|  | | | | | | | |
| ------------------ | | | | | | | |
|  | | | | | | | |
|  | | | | | | | |
| **Input Sequence** | SM-Roxy-RH01221661_S26_L001_R1_001_(paired)_contig_25_147 # 139213 # 140721 # -1 # ID=25_147;partial=00;start_type=ATG;rbs_motif=GGAG/GAGG;rbs_spacer=5-10bp;gc_cont=0.533 | | | | | | |
|  | PROJECT ID | ACCESSION ID | ORGANISMS | CLASS | PROTEIN FUNCTION | PROTEIN ID | %IDENTITY |
| **Matched Family** | [30687](http://www.ncbi.nlm.nih.gov/bioproject?db=bioproject&cmd=ShowDetailView&TermToSearch=30687) | [AM933172](http://www.ncbi.nlm.nih.gov/nuccore/AM933172) | Salmonella enterica subsp. enterica serovar Enteritidis str. P125109 complete genome. | Gammaproteobacteria | putative monooxygenase | [CAR33085](http://www.ncbi.nlm.nih.gov/protein/CAR33085.1) | 100.0 |
|  | | | | | | | |
|  | | | | | | | |
| ------------------ | | | | | | | |
|  | | | | | | | |
|  | | | | | | | |
| **Input Sequence** | SM-Roxy-RH01221661_S26_L001_R1_001_(paired)_contig_57_3 # 1760 # 3262 # -1 # ID=57_3;partial=00;start_type=ATG;rbs_motif=GGA/GAG/AGG;rbs_spacer=5-10bp;gc_cont=0.485 | | | | | | |
|  | PROJECT ID | ACCESSION ID | ORGANISMS | CLASS | PROTEIN FUNCTION | PROTEIN ID | %IDENTITY |
| **Matched Family** | [19459](http://www.ncbi.nlm.nih.gov/bioproject?db=bioproject&cmd=ShowDetailView&TermToSearch=19459) | [CP001127](http://www.ncbi.nlm.nih.gov/nuccore/CP001127) | Salmonella enterica subsp. enterica serovar Schwarzengrund str. CVM19633, complete genome. | Gammaproteobacteria | inner membrane transporter YjeM | [ACF91451](http://www.ncbi.nlm.nih.gov/protein/ACF91451.1) | 100.0 |
|  | | | | | | | |
|  | | | | | | | |
| ------------------ | | | | | | | |
|  | | | | | | | |
|  | | | | | | | |
| **Input Sequence** | SM-Roxy-RH01221661_S26_L001_R1_001_(paired)_contig_41_19 # 24790 # 26286 # -1 # ID=41_19;partial=00;start_type=ATG;rbs_motif=AGGA/GGAG/GAGG;rbs_spacer=11-12bp;gc_cont=0.610 | | | | | | |
|  | PROJECT ID | ACCESSION ID | ORGANISMS | CLASS | PROTEIN FUNCTION | PROTEIN ID | %IDENTITY |
| **Matched Family** | [30687](http://www.ncbi.nlm.nih.gov/bioproject?db=bioproject&cmd=ShowDetailView&TermToSearch=30687) | [AM933172](http://www.ncbi.nlm.nih.gov/nuccore/AM933172) | Salmonella enterica subsp. enterica serovar Enteritidis str. P125109 complete genome. | Gammaproteobacteria | putative L-xylulose kinase | [CAR35075](http://www.ncbi.nlm.nih.gov/protein/CAR35075.1) | 100.0 |
|  | | | | | | | |
|  | | | | | | | |
| ------------------ | | | | | | | |
|  | | | | | | | |
|  | | | | | | | |
| **Input Sequence** | SM-Roxy-RH01221661_S26_L001_R1_001_(paired)_contig_12_262 # 265297 # 266790 # 1 # ID=12_262;partial=00;start_type=ATG;rbs_motif=GGxGG;rbs_spacer=5-10bp;gc_cont=0.421 | | | | | | |
|  | PROJECT ID | ACCESSION ID | ORGANISMS | CLASS | PROTEIN FUNCTION | PROTEIN ID | %IDENTITY |
| **Matched Family** | [30687](http://www.ncbi.nlm.nih.gov/bioproject?db=bioproject&cmd=ShowDetailView&TermToSearch=30687) | [AM933172](http://www.ncbi.nlm.nih.gov/nuccore/AM933172) | Salmonella enterica subsp. enterica serovar Enteritidis str. P125109 complete genome. | Gammaproteobacteria | putative outer membrane secretory protein | [CAR33233](http://www.ncbi.nlm.nih.gov/protein/CAR33233.1) | 100.0 |
|  | | | | | | | |
|  | | | | | | | |
| ------------------ | | | | | | | |
|  | | | | | | | |
|  | | | | | | | |
| **Input Sequence** | SM-Roxy-RH01221661_S26_L001_R1_001_(paired)_contig_25_182 # 182718 # 184211 # 1 # ID=25_182;partial=00;start_type=ATG;rbs_motif=GGA/GAG/AGG;rbs_spacer=5-10bp;gc_cont=0.536 | | | | | | |
|  | PROJECT ID | ACCESSION ID | ORGANISMS | CLASS | PROTEIN FUNCTION | PROTEIN ID | %IDENTITY |
| **Matched Family** | [13086](http://www.ncbi.nlm.nih.gov/bioproject?db=bioproject&cmd=ShowDetailView&TermToSearch=13086) | [CP000026](http://www.ncbi.nlm.nih.gov/nuccore/CP000026) | Salmonella enterica subsp. enterica serovar Paratyphi A str. ATCC 9150, complete genome. | Gammaproteobacteria | L-asparagine permease | [AAV77233](http://www.ncbi.nlm.nih.gov/protein/AAV77233.1) | 100.0 |
|  | | | | | | | |
|  | | | | | | | |
| ------------------ | | | | | | | |
|  | | | | | | | |
|  | | | | | | | |
| **Input Sequence** | SM-Roxy-RH01221661_S26_L001_R1_001_(paired)_contig_12_144 # 133658 # 135151 # -1 # ID=12_144;partial=00;start_type=ATG;rbs_motif=3Base/5BMM;rbs_spacer=13-15bp;gc_cont=0.454 | | | | | | |
|  | PROJECT ID | ACCESSION ID | ORGANISMS | CLASS | PROTEIN FUNCTION | PROTEIN ID | %IDENTITY |
| **Matched Family** | [30687](http://www.ncbi.nlm.nih.gov/bioproject?db=bioproject&cmd=ShowDetailView&TermToSearch=30687) | [AM933172](http://www.ncbi.nlm.nih.gov/nuccore/AM933172) | Salmonella enterica subsp. enterica serovar Enteritidis str. P125109 complete genome. | Gammaproteobacteria | membrane protein | [CAR33350](http://www.ncbi.nlm.nih.gov/protein/CAR33350.1) | 100.0 |
|  | | | | | | | |
|  | | | | | | | |
| ------------------ | | | | | | | |
|  | | | | | | | |
|  | | | | | | | |
| **Input Sequence** | SM-Roxy-RH01221661_S26_L001_R1_001_(paired)_contig_35_18 # 18942 # 20435 # 1 # ID=35_18;partial=00;start_type=ATG;rbs_motif=AGGAG;rbs_spacer=5-10bp;gc_cont=0.525 | | | | | | |
|  | PROJECT ID | ACCESSION ID | ORGANISMS | CLASS | PROTEIN FUNCTION | PROTEIN ID | %IDENTITY |
| **Matched Family** | [18747](http://www.ncbi.nlm.nih.gov/bioproject?db=bioproject&cmd=ShowDetailView&TermToSearch=18747) | [CP001113](http://www.ncbi.nlm.nih.gov/nuccore/CP001113) | Salmonella enterica subsp. enterica serovar Newport str. SL254, complete genome. | Gammaproteobacteria | sulfatase | [ACF62172](http://www.ncbi.nlm.nih.gov/protein/ACF62172.1) | 100.0 |
|  | | | | | | | |
|  | | | | | | | |
| ------------------ | | | | | | | |
|  | | | | | | | |
|  | | | | | | | |
| **Input Sequence** | SM-Roxy-RH01221661_S26_L001_R1_001_(paired)_contig_30_14 # 12182 # 13672 # 1 # ID=30_14;partial=00;start_type=ATG;rbs_motif=GGAG/GAGG;rbs_spacer=5-10bp;gc_cont=0.554 | | | | | | |
|  | PROJECT ID | ACCESSION ID | ORGANISMS | CLASS | PROTEIN FUNCTION | PROTEIN ID | %IDENTITY |
| **Matched Family** | [18747](http://www.ncbi.nlm.nih.gov/bioproject?db=bioproject&cmd=ShowDetailView&TermToSearch=18747) | [CP001113](http://www.ncbi.nlm.nih.gov/nuccore/CP001113) | Salmonella enterica subsp. enterica serovar Newport str. SL254, complete genome. | Gammaproteobacteria | putative sialic acid transporter | [ACF64741](http://www.ncbi.nlm.nih.gov/protein/ACF64741.1) | 100.0 |
|  | | | | | | | |
|  | | | | | | | |
| ------------------ | | | | | | | |
|  | | | | | | | |
|  | | | | | | | |
| **Input Sequence** | SM-Roxy-RH01221661_S26_L001_R1_001_(paired)_contig_25_172 # 168879 # 170366 # -1 # ID=25_172;partial=00;start_type=ATG;rbs_motif=GGA/GAG/AGG;rbs_spacer=5-10bp;gc_cont=0.579 | | | | | | |
|  | PROJECT ID | ACCESSION ID | ORGANISMS | CLASS | PROTEIN FUNCTION | PROTEIN ID | %IDENTITY |
| **Matched Family** | [30687](http://www.ncbi.nlm.nih.gov/bioproject?db=bioproject&cmd=ShowDetailView&TermToSearch=30687) | [AM933172](http://www.ncbi.nlm.nih.gov/nuccore/AM933172) | Salmonella enterica subsp. enterica serovar Enteritidis str. P125109 complete genome. | Gammaproteobacteria | methyl viologen resistance protein SmvA | [CAR33060](http://www.ncbi.nlm.nih.gov/protein/CAR33060.1) | 100.0 |
|  | | | | | | | |
|  | | | | | | | |
| ------------------ | | | | | | | |
|  | | | | | | | |
|  | | | | | | | |
| **Input Sequence** | SM-Roxy-RH01221661_S26_L001_R1_001_(paired)_contig_42_176 # 196266 # 197753 # -1 # ID=42_176;partial=00;start_type=ATG;rbs_motif=GGAG/GAGG;rbs_spacer=5-10bp;gc_cont=0.517 | | | | | | |
|  | PROJECT ID | ACCESSION ID | ORGANISMS | CLASS | PROTEIN FUNCTION | PROTEIN ID | %IDENTITY |
| **Matched Family** | [20063](http://www.ncbi.nlm.nih.gov/bioproject?db=bioproject&cmd=ShowDetailView&TermToSearch=20063) | [CP001138](http://www.ncbi.nlm.nih.gov/nuccore/CP001138) | Salmonella enterica subsp. enterica serovar Agona str. SL483, complete genome. | Gammaproteobacteria | sulfatase | [ACH51092](http://www.ncbi.nlm.nih.gov/protein/ACH51092.1) | 100.0 |
|  | | | | | | | |
|  | | | | | | | |
| ------------------ | | | | | | | |
|  | | | | | | | |
|  | | | | | | | |
| **Input Sequence** | SM-Roxy-RH01221661_S26_L001_R1_001_(paired)_contig_19_17 # 14259 # 15722 # 1 # ID=19_17;partial=00;start_type=ATG;rbs_motif=GGAG/GAGG;rbs_spacer=5-10bp;gc_cont=0.541 | | | | | | |
|  | PROJECT ID | ACCESSION ID | ORGANISMS | CLASS | PROTEIN FUNCTION | PROTEIN ID | %IDENTITY |
| **Matched Family** | [30687](http://www.ncbi.nlm.nih.gov/bioproject?db=bioproject&cmd=ShowDetailView&TermToSearch=30687) | [AM933172](http://www.ncbi.nlm.nih.gov/nuccore/AM933172) | Salmonella enterica subsp. enterica serovar Enteritidis str. P125109 complete genome. | Gammaproteobacteria | citrate carrier | [CAR32175](http://www.ncbi.nlm.nih.gov/protein/CAR32175.1) | 100.0 |
|  | | | | | | | |
|  | | | | | | | |
| ------------------ | | | | | | | |
|  | | | | | | | |
|  | | | | | | | |
| **Input Sequence** | SM-Roxy-RH01221661_S26_L001_R1_001_(paired)_contig_25_4 # 1645 # 3099 # 1 # ID=25_4;partial=00;start_type=ATG;rbs_motif=AGGAG;rbs_spacer=5-10bp;gc_cont=0.460 | | | | | | |
|  | PROJECT ID | ACCESSION ID | ORGANISMS | CLASS | PROTEIN FUNCTION | PROTEIN ID | %IDENTITY |
| **Matched Family** | [19459](http://www.ncbi.nlm.nih.gov/bioproject?db=bioproject&cmd=ShowDetailView&TermToSearch=19459) | [CP001127](http://www.ncbi.nlm.nih.gov/nuccore/CP001127) | Salmonella enterica subsp. enterica serovar Schwarzengrund str. CVM19633, complete genome. | Gammaproteobacteria | translocation machinery component | [ACF92454](http://www.ncbi.nlm.nih.gov/protein/ACF92454.1) | 100.0 |
|  | | | | | | | |
|  | | | | | | | |
| ------------------ | | | | | | | |
|  | | | | | | | |
|  | | | | | | | |
| **Input Sequence** | SM-Roxy-RH01221661_S26_L001_R1_001_(paired)_contig_2_45 # 50168 # 51619 # 1 # ID=2_45;partial=00;start_type=ATG;rbs_motif=AGGAG;rbs_spacer=5-10bp;gc_cont=0.564 | | | | | | |
|  | PROJECT ID | ACCESSION ID | ORGANISMS | CLASS | PROTEIN FUNCTION | PROTEIN ID | %IDENTITY |
| **Matched Family** | [30687](http://www.ncbi.nlm.nih.gov/bioproject?db=bioproject&cmd=ShowDetailView&TermToSearch=30687) | [AM933172](http://www.ncbi.nlm.nih.gov/nuccore/AM933172) | Salmonella enterica subsp. enterica serovar Enteritidis str. P125109 complete genome. | Gammaproteobacteria | PrpD protein | [CAR31939](http://www.ncbi.nlm.nih.gov/protein/CAR31939.1) | 100.0 |
|  | | | | | | | |
|  | | | | | | | |
| ------------------ | | | | | | | |
|  | | | | | | | |
|  | | | | | | | |
| **Input Sequence** | SM-Roxy-RH01221661_S26_L001_R1_001_(paired)_contig_22_207 # 201458 # 202891 # -1 # ID=22_207;partial=00;start_type=ATG;rbs_motif=GGAG/GAGG;rbs_spacer=5-10bp;gc_cont=0.490 | | | | | | |
|  | PROJECT ID | ACCESSION ID | ORGANISMS | CLASS | PROTEIN FUNCTION | PROTEIN ID | %IDENTITY |
| **Matched Family** | [30687](http://www.ncbi.nlm.nih.gov/bioproject?db=bioproject&cmd=ShowDetailView&TermToSearch=30687) | [AM933172](http://www.ncbi.nlm.nih.gov/nuccore/AM933172) | Salmonella enterica subsp. enterica serovar Enteritidis str. P125109 complete genome. | Gammaproteobacteria | putative exported protein | [CAR32648](http://www.ncbi.nlm.nih.gov/protein/CAR32648.1) | 100.0 |
|  | | | | | | | |
|  | | | | | | | |
| ------------------ | | | | | | | |
|  | | | | | | | |
|  | | | | | | | |
| **Input Sequence** | SM-Roxy-RH01221661_S26_L001_R1_001_(paired)_contig_21_256 # 258050 # 259486 # -1 # ID=21_256;partial=00;start_type=ATG;rbs_motif=None;rbs_spacer=None;gc_cont=0.578 | | | | | | |
|  | PROJECT ID | ACCESSION ID | ORGANISMS | CLASS | PROTEIN FUNCTION | PROTEIN ID | %IDENTITY |
| **Matched Family** | [19467](http://www.ncbi.nlm.nih.gov/bioproject?db=bioproject&cmd=ShowDetailView&TermToSearch=19467) | [CP001144](http://www.ncbi.nlm.nih.gov/nuccore/CP001144) | Salmonella enterica subsp. enterica serovar Dublin str. C | Gammaproteobacteria | 2021853, complete genome. | [ACH76578](http://www.ncbi.nlm.nih.gov/protein/ACH76578.1) | 100.0 |
|  | | | | | | | |
|  | | | | | | | |
| ------------------ | | | | | | | |
|  | | | | | | | |
|  | | | | | | | |
| **Input Sequence** | SM-Roxy-RH01221661_S26_L001_R1_001_(paired)_contig_42_101 # 100977 # 102413 # -1 # ID=42_101;partial=00;start_type=ATG;rbs_motif=GGAG/GAGG;rbs_spacer=5-10bp;gc_cont=0.431 | | | | | | |
|  | PROJECT ID | ACCESSION ID | ORGANISMS | CLASS | PROTEIN FUNCTION | PROTEIN ID | %IDENTITY |
| **Matched Family** | [30687](http://www.ncbi.nlm.nih.gov/bioproject?db=bioproject&cmd=ShowDetailView&TermToSearch=30687) | [AM933172](http://www.ncbi.nlm.nih.gov/nuccore/AM933172) | Salmonella enterica subsp. enterica serovar Enteritidis str. P125109 complete genome. | Gammaproteobacteria | putative ion:amino acid symporter | [CAR32490](http://www.ncbi.nlm.nih.gov/protein/CAR32490.1) | 100.0 |
|  | | | | | | | |
|  | | | | | | | |
| ------------------ | | | | | | | |
|  | | | | | | | |
|  | | | | | | | |
| **Input Sequence** | SM-Roxy-RH01221661_S26_L001_R1_001_(paired)_contig_13_34 # 34940 # 36337 # 1 # ID=13_34;partial=00;start_type=ATG;rbs_motif=GGAG/GAGG;rbs_spacer=5-10bp;gc_cont=0.529 | | | | | | |
|  | PROJECT ID | ACCESSION ID | ORGANISMS | CLASS | PROTEIN FUNCTION | PROTEIN ID | %IDENTITY |
| **Matched Family** | [241](http://www.ncbi.nlm.nih.gov/bioproject?db=bioproject&cmd=ShowDetailView&TermToSearch=241) | [AE006468](http://www.ncbi.nlm.nih.gov/nuccore/AE006468) | Salmonella enterica subsp. enterica serovar Typhimurium str. LT2, complete genome. | Gammaproteobacteria | putative PTS enzyme IIsga subunit | [AAL23203](http://www.ncbi.nlm.nih.gov/protein/AAL23203.1) | 100.0 |
|  | | | | | | | |
|  | | | | | | | |
| ------------------ | | | | | | | |
|  | | | | | | | |
|  | | | | | | | |
| **Input Sequence** | SM-Roxy-RH01221661_S26_L001_R1_001_(paired)_contig_11_274 # 291038 # 292465 # -1 # ID=11_274;partial=00;start_type=ATG;rbs_motif=AGGAG;rbs_spacer=5-10bp;gc_cont=0.556 | | | | | | |
|  | PROJECT ID | ACCESSION ID | ORGANISMS | CLASS | PROTEIN FUNCTION | PROTEIN ID | %IDENTITY |
| **Matched Family** | [19459](http://www.ncbi.nlm.nih.gov/bioproject?db=bioproject&cmd=ShowDetailView&TermToSearch=19459) | [CP001127](http://www.ncbi.nlm.nih.gov/nuccore/CP001127) | Salmonella enterica subsp. enterica serovar Schwarzengrund str. CVM19633, complete genome. | Gammaproteobacteria | 4-hydroxybenzoate decarboxylase, subunit C | [ACF92543](http://www.ncbi.nlm.nih.gov/protein/ACF92543.1) | 100.0 |
|  | | | | | | | |
|  | | | | | | | |
| ------------------ | | | | | | | |
|  | | | | | | | |
|  | | | | | | | |
| **Input Sequence** | SM-Roxy-RH01221661_S26_L001_R1_001_(paired)_contig_29_29 # 27340 # 28767 # -1 # ID=29_29;partial=00;start_type=ATG;rbs_motif=GGAG/GAGG;rbs_spacer=5-10bp;gc_cont=0.523 | | | | | | |
|  | PROJECT ID | ACCESSION ID | ORGANISMS | CLASS | PROTEIN FUNCTION | PROTEIN ID | %IDENTITY |
| **Matched Family** | [19467](http://www.ncbi.nlm.nih.gov/bioproject?db=bioproject&cmd=ShowDetailView&TermToSearch=19467) | [CP001144](http://www.ncbi.nlm.nih.gov/nuccore/CP001144) | Salmonella enterica subsp. enterica serovar Dublin str. C | Gammaproteobacteria | 2021853, complete genome. | [ACH73670](http://www.ncbi.nlm.nih.gov/protein/ACH73670.1) | 100.0 |
|  | | | | | | | |
|  | | | | | | | |
| ------------------ | | | | | | | |
|  | | | | | | | |
|  | | | | | | | |
| **Input Sequence** | SM-Roxy-RH01221661_S26_L001_R1_001_(paired)_contig_29_31 # 30466 # 31887 # 1 # ID=29_31;partial=00;start_type=ATG;rbs_motif=AGGAG;rbs_spacer=5-10bp;gc_cont=0.506 | | | | | | |
|  | PROJECT ID | ACCESSION ID | ORGANISMS | CLASS | PROTEIN FUNCTION | PROTEIN ID | %IDENTITY |
| **Matched Family** | [20993](http://www.ncbi.nlm.nih.gov/bioproject?db=bioproject&cmd=ShowDetailView&TermToSearch=20993) | [CP000857](http://www.ncbi.nlm.nih.gov/nuccore/CP000857) | Salmonella enterica subsp. enterica serovar Paratyphi C strain RKS4594, complete genome. | Gammaproteobacteria | putative amino acid transporter | [ACN45509](http://www.ncbi.nlm.nih.gov/protein/ACN45509.1) | 100.0 |
|  | | | | | | | |
|  | | | | | | | |
| ------------------ | | | | | | | |
|  | | | | | | | |
|  | | | | | | | |
| **Input Sequence** | SM-Roxy-RH01221661_S26_L001_R1_001_(paired)_contig_11_214 # 220799 # 222217 # -1 # ID=11_214;partial=00;start_type=ATG;rbs_motif=GGAG/GAGG;rbs_spacer=5-10bp;gc_cont=0.586 | | | | | | |
|  | PROJECT ID | ACCESSION ID | ORGANISMS | CLASS | PROTEIN FUNCTION | PROTEIN ID | %IDENTITY |
| **Matched Family** | [19467](http://www.ncbi.nlm.nih.gov/bioproject?db=bioproject&cmd=ShowDetailView&TermToSearch=19467) | [CP001144](http://www.ncbi.nlm.nih.gov/nuccore/CP001144) | Salmonella enterica subsp. enterica serovar Dublin str. C | Gammaproteobacteria | 2021853, complete genome. | [ACH74553](http://www.ncbi.nlm.nih.gov/protein/ACH74553.1) | 100.0 |
|  | | | | | | | |
|  | | | | | | | |
| ------------------ | | | | | | | |
|  | | | | | | | |
|  | | | | | | | |
| **Input Sequence** | SM-Roxy-RH01221661_S26_L001_R1_001_(paired)_contig_35_25 # 29262 # 30635 # -1 # ID=35_25;partial=00;start_type=ATG;rbs_motif=GGAG/GAGG;rbs_spacer=5-10bp;gc_cont=0.493 | | | | | | |
|  | PROJECT ID | ACCESSION ID | ORGANISMS | CLASS | PROTEIN FUNCTION | PROTEIN ID | %IDENTITY |
| **Matched Family** | [18747](http://www.ncbi.nlm.nih.gov/bioproject?db=bioproject&cmd=ShowDetailView&TermToSearch=18747) | [CP001113](http://www.ncbi.nlm.nih.gov/nuccore/CP001113) | Salmonella enterica subsp. enterica serovar Newport str. SL254, complete genome. | Gammaproteobacteria | xylose-proton symporter | [ACF64477](http://www.ncbi.nlm.nih.gov/protein/ACF64477.1) | 100.0 |
|  | | | | | | | |
|  | | | | | | | |
| ------------------ | | | | | | | |
|  | | | | | | | |
|  | | | | | | | |
| **Input Sequence** | SM-Roxy-RH01221661_S26_L001_R1_001_(paired)_contig_11_18 # 17104 # 18516 # 1 # ID=11_18;partial=00;start_type=ATG;rbs_motif=GGAG/GAGG;rbs_spacer=5-10bp;gc_cont=0.589 | | | | | | |
|  | PROJECT ID | ACCESSION ID | ORGANISMS | CLASS | PROTEIN FUNCTION | PROTEIN ID | %IDENTITY |
| **Matched Family** | [13086](http://www.ncbi.nlm.nih.gov/bioproject?db=bioproject&cmd=ShowDetailView&TermToSearch=13086) | [CP000026](http://www.ncbi.nlm.nih.gov/nuccore/CP000026) | Salmonella enterica subsp. enterica serovar Paratyphi A str. ATCC 9150, complete genome. | Gammaproteobacteria | SufI protein | [AAV78876](http://www.ncbi.nlm.nih.gov/protein/AAV78876.1) | 100.0 |
|  | | | | | | | |
|  | | | | | | | |
| ------------------ | | | | | | | |
|  | | | | | | | |
|  | | | | | | | |
| **Input Sequence** | SM-Roxy-RH01221661_S26_L001_R1_001_(paired)_contig_40_44 # 45255 # 46664 # -1 # ID=40_44;partial=00;start_type=ATG;rbs_motif=GGA/GAG/AGG;rbs_spacer=5-10bp;gc_cont=0.571 | | | | | | |
|  | PROJECT ID | ACCESSION ID | ORGANISMS | CLASS | PROTEIN FUNCTION | PROTEIN ID | %IDENTITY |
| **Matched Family** | [30687](http://www.ncbi.nlm.nih.gov/bioproject?db=bioproject&cmd=ShowDetailView&TermToSearch=30687) | [AM933172](http://www.ncbi.nlm.nih.gov/nuccore/AM933172) | Salmonella enterica subsp. enterica serovar Enteritidis str. P125109 complete genome. | Gammaproteobacteria | putative type I secretion protein | [CAR34192](http://www.ncbi.nlm.nih.gov/protein/CAR34192.1) | 100.0 |
|  | | | | | | | |
|  | | | | | | | |
| ------------------ | | | | | | | |
|  | | | | | | | |
|  | | | | | | | |
| **Input Sequence** | SM-Roxy-RH01221661_S26_L001_R1_001_(paired)_contig_14_48 # 53128 # 54534 # 1 # ID=14_48;partial=00;start_type=ATG;rbs_motif=GGAG/GAGG;rbs_spacer=5-10bp;gc_cont=0.515 | | | | | | |
|  | PROJECT ID | ACCESSION ID | ORGANISMS | CLASS | PROTEIN FUNCTION | PROTEIN ID | %IDENTITY |
| **Matched Family** | [13086](http://www.ncbi.nlm.nih.gov/bioproject?db=bioproject&cmd=ShowDetailView&TermToSearch=13086) | [CP000026](http://www.ncbi.nlm.nih.gov/nuccore/CP000026) | Salmonella enterica subsp. enterica serovar Paratyphi A str. ATCC 9150, complete genome. | Gammaproteobacteria | putative outer membrane protein | [AAV77956](http://www.ncbi.nlm.nih.gov/protein/AAV77956.1) | 100.0 |
|  | | | | | | | |
|  | | | | | | | |
| ------------------ | | | | | | | |
|  | | | | | | | |
|  | | | | | | | |
| **Input Sequence** | SM-Roxy-RH01221661_S26_L001_R1_001_(paired)_contig_29_33 # 33056 # 34462 # 1 # ID=29_33;partial=00;start_type=ATG;rbs_motif=GGAG/GAGG;rbs_spacer=5-10bp;gc_cont=0.530 | | | | | | |
|  | PROJECT ID | ACCESSION ID | ORGANISMS | CLASS | PROTEIN FUNCTION | PROTEIN ID | %IDENTITY |
| **Matched Family** | [19467](http://www.ncbi.nlm.nih.gov/bioproject?db=bioproject&cmd=ShowDetailView&TermToSearch=19467) | [CP001144](http://www.ncbi.nlm.nih.gov/nuccore/CP001144) | Salmonella enterica subsp. enterica serovar Dublin str. C | Gammaproteobacteria | 2021853, complete genome. | [ACH76925](http://www.ncbi.nlm.nih.gov/protein/ACH76925.1) | 100.0 |
|  | | | | | | | |
|  | | | | | | | |
| ------------------ | | | | | | | |
|  | | | | | | | |
|  | | | | | | | |
| **Input Sequence** | SM-Roxy-RH01221661_S26_L001_R1_001_(paired)_contig_21_183 # 170357 # 171760 # -1 # ID=21_183;partial=00;start_type=ATG;rbs_motif=GGA/GAG/AGG;rbs_spacer=5-10bp;gc_cont=0.511 | | | | | | |
|  | PROJECT ID | ACCESSION ID | ORGANISMS | CLASS | PROTEIN FUNCTION | PROTEIN ID | %IDENTITY |
| **Matched Family** | [30687](http://www.ncbi.nlm.nih.gov/bioproject?db=bioproject&cmd=ShowDetailView&TermToSearch=30687) | [AM933172](http://www.ncbi.nlm.nih.gov/nuccore/AM933172) | Salmonella enterica subsp. enterica serovar Enteritidis str. P125109 complete genome. | Gammaproteobacteria | putative colanic acid biosynthesis protein | [CAR33678](http://www.ncbi.nlm.nih.gov/protein/CAR33678.1) | 100.0 |
|  | | | | | | | |
|  | | | | | | | |
| ------------------ | | | | | | | |
|  | | | | | | | |
|  | | | | | | | |
| **Input Sequence** | SM-Roxy-RH01221661_S26_L001_R1_001_(paired)_contig_10_2 # 1066 # 2466 # 1 # ID=10_2;partial=00;start_type=ATG;rbs_motif=None;rbs_spacer=None;gc_cont=0.531 | | | | | | |
|  | PROJECT ID | ACCESSION ID | ORGANISMS | CLASS | PROTEIN FUNCTION | PROTEIN ID | %IDENTITY |
| **Matched Family** | [30687](http://www.ncbi.nlm.nih.gov/bioproject?db=bioproject&cmd=ShowDetailView&TermToSearch=30687) | [AM933172](http://www.ncbi.nlm.nih.gov/nuccore/AM933172) | Salmonella enterica subsp. enterica serovar Enteritidis str. P125109 complete genome. | Gammaproteobacteria | putative exported protein | [CAR35642](http://www.ncbi.nlm.nih.gov/protein/CAR35642.1) | 100.0 |
|  | | | | | | | |
|  | | | | | | | |
| ------------------ | | | | | | | |
|  | | | | | | | |
|  | | | | | | | |
| **Input Sequence** | SM-Roxy-RH01221661_S26_L001_R1_001_(paired)_contig_29_30 # 28978 # 30375 # 1 # ID=29_30;partial=00;start_type=ATG;rbs_motif=GGAG/GAGG;rbs_spacer=5-10bp;gc_cont=0.496 | | | | | | |
|  | PROJECT ID | ACCESSION ID | ORGANISMS | CLASS | PROTEIN FUNCTION | PROTEIN ID | %IDENTITY |
| **Matched Family** | [19467](http://www.ncbi.nlm.nih.gov/bioproject?db=bioproject&cmd=ShowDetailView&TermToSearch=19467) | [CP001144](http://www.ncbi.nlm.nih.gov/nuccore/CP001144) | Salmonella enterica subsp. enterica serovar Dublin str. C | Gammaproteobacteria | 2021853, complete genome. | [ACH74154](http://www.ncbi.nlm.nih.gov/protein/ACH74154.1) | 100.0 |
|  | | | | | | | |
|  | | | | | | | |
| ------------------ | | | | | | | |
|  | | | | | | | |
|  | | | | | | | |
| **Input Sequence** | SM-Roxy-RH01221661_S26_L001_R1_001_(paired)_contig_21_134 # 115323 # 116717 # 1 # ID=21_134;partial=00;start_type=ATG;rbs_motif=GGA/GAG/AGG;rbs_spacer=5-10bp;gc_cont=0.591 | | | | | | |
|  | PROJECT ID | ACCESSION ID | ORGANISMS | CLASS | PROTEIN FUNCTION | PROTEIN ID | %IDENTITY |
| **Matched Family** | [30687](http://www.ncbi.nlm.nih.gov/bioproject?db=bioproject&cmd=ShowDetailView&TermToSearch=30687) | [AM933172](http://www.ncbi.nlm.nih.gov/nuccore/AM933172) | Salmonella enterica subsp. enterica serovar Enteritidis str. P125109 complete genome. | Gammaproteobacteria | putative CoA-dependent proprionaldehyde dehydrogenase | [CAR33629](http://www.ncbi.nlm.nih.gov/protein/CAR33629.1) | 100.0 |
|  | | | | | | | |
|  | | | | | | | |
| ------------------ | | | | | | | |
|  | | | | | | | |
|  | | | | | | | |
| **Input Sequence** | SM-Roxy-RH01221661_S26_L001_R1_001_(paired)_contig_42_5 # 5485 # 6876 # 1 # ID=42_5;partial=00;start_type=ATG;rbs_motif=AGGAG;rbs_spacer=5-10bp;gc_cont=0.596 | | | | | | |
|  | PROJECT ID | ACCESSION ID | ORGANISMS | CLASS | PROTEIN FUNCTION | PROTEIN ID | %IDENTITY |
| **Matched Family** | [30687](http://www.ncbi.nlm.nih.gov/bioproject?db=bioproject&cmd=ShowDetailView&TermToSearch=30687) | [AM933172](http://www.ncbi.nlm.nih.gov/nuccore/AM933172) | Salmonella enterica subsp. enterica serovar Enteritidis str. P125109 complete genome. | Gammaproteobacteria | putative membrane protein | [CAR32586](http://www.ncbi.nlm.nih.gov/protein/CAR32586.1) | 100.0 |
|  | | | | | | | |
|  | | | | | | | |
| ------------------ | | | | | | | |
|  | | | | | | | |
|  | | | | | | | |
| **Input Sequence** | SM-Roxy-RH01221661_S26_L001_R1_001_(paired)_contig_3_44 # 45583 # 46974 # -1 # ID=3_44;partial=00;start_type=ATG;rbs_motif=GGA/GAG/AGG;rbs_spacer=5-10bp;gc_cont=0.498 | | | | | | |
|  | PROJECT ID | ACCESSION ID | ORGANISMS | CLASS | PROTEIN FUNCTION | PROTEIN ID | %IDENTITY |
| **Matched Family** | [18747](http://www.ncbi.nlm.nih.gov/bioproject?db=bioproject&cmd=ShowDetailView&TermToSearch=18747) | [CP001113](http://www.ncbi.nlm.nih.gov/nuccore/CP001113) | Salmonella enterica subsp. enterica serovar Newport str. SL254, complete genome. | Gammaproteobacteria | phosphoglycerate transport: transporter | [ACF63285](http://www.ncbi.nlm.nih.gov/protein/ACF63285.1) | 100.0 |
|  | | | | | | | |
|  | | | | | | | |
| ------------------ | | | | | | | |
|  | | | | | | | |
|  | | | | | | | |
| **Input Sequence** | SM-Roxy-RH01221661_S26_L001_R1_001_(paired)_contig_31_24 # 22680 # 24068 # 1 # ID=31_24;partial=00;start_type=GTG;rbs_motif=GGA/GAG/AGG;rbs_spacer=5-10bp;gc_cont=0.368 | | | | | | |
|  | PROJECT ID | ACCESSION ID | ORGANISMS | CLASS | PROTEIN FUNCTION | PROTEIN ID | %IDENTITY |
| **Matched Family** | [30687](http://www.ncbi.nlm.nih.gov/bioproject?db=bioproject&cmd=ShowDetailView&TermToSearch=30687) | [AM933172](http://www.ncbi.nlm.nih.gov/nuccore/AM933172) | Salmonella enterica subsp. enterica serovar Enteritidis str. P125109 complete genome. | Gammaproteobacteria | putative integral membrane protein | [CAR35590](http://www.ncbi.nlm.nih.gov/protein/CAR35590.1) | 100.0 |
|  | | | | | | | |
|  | | | | | | | |
| ------------------ | | | | | | | |
|  | | | | | | | |
|  | | | | | | | |
| **Input Sequence** | SM-Roxy-RH01221661_S26_L001_R1_001_(paired)_contig_5_26 # 25970 # 27352 # -1 # ID=5_26;partial=00;start_type=ATG;rbs_motif=GGAG/GAGG;rbs_spacer=5-10bp;gc_cont=0.526 | | | | | | |
|  | PROJECT ID | ACCESSION ID | ORGANISMS | CLASS | PROTEIN FUNCTION | PROTEIN ID | %IDENTITY |
| **Matched Family** | [19467](http://www.ncbi.nlm.nih.gov/bioproject?db=bioproject&cmd=ShowDetailView&TermToSearch=19467) | [CP001144](http://www.ncbi.nlm.nih.gov/nuccore/CP001144) | Salmonella enterica subsp. enterica serovar Dublin str. C | Gammaproteobacteria | 2021853, complete genome. | [ACH78007](http://www.ncbi.nlm.nih.gov/protein/ACH78007.1) | 100.0 |
|  | | | | | | | |
|  | | | | | | | |
| ------------------ | | | | | | | |
|  | | | | | | | |
|  | | | | | | | |
| **Input Sequence** | SM-Roxy-RH01221661_S26_L001_R1_001_(paired)_contig_42_42 # 39622 # 40998 # -1 # ID=42_42;partial=00;start_type=ATG;rbs_motif=GGAG/GAGG;rbs_spacer=5-10bp;gc_cont=0.532 | | | | | | |
|  | PROJECT ID | ACCESSION ID | ORGANISMS | CLASS | PROTEIN FUNCTION | PROTEIN ID | %IDENTITY |
| **Matched Family** | [13086](http://www.ncbi.nlm.nih.gov/bioproject?db=bioproject&cmd=ShowDetailView&TermToSearch=13086) | [CP000026](http://www.ncbi.nlm.nih.gov/nuccore/CP000026) | Salmonella enterica subsp. enterica serovar Paratyphi A str. ATCC 9150, complete genome. | Gammaproteobacteria | putative 4-hydroxyphenylacetate permease | [AAV77663](http://www.ncbi.nlm.nih.gov/protein/AAV77663.1) | 100.0 |
|  | | | | | | | |
|  | | | | | | | |
| ------------------ | | | | | | | |
|  | | | | | | | |
|  | | | | | | | |
| **Input Sequence** | SM-Roxy-RH01221661_S26_L001_R1_001_(paired)_contig_20_9 # 10006 # 11379 # 1 # ID=20_9;partial=00;start_type=ATG;rbs_motif=GGA/GAG/AGG;rbs_spacer=5-10bp;gc_cont=0.557 | | | | | | |
|  | PROJECT ID | ACCESSION ID | ORGANISMS | CLASS | PROTEIN FUNCTION | PROTEIN ID | %IDENTITY |
| **Matched Family** | [18747](http://www.ncbi.nlm.nih.gov/bioproject?db=bioproject&cmd=ShowDetailView&TermToSearch=18747) | [CP001113](http://www.ncbi.nlm.nih.gov/nuccore/CP001113) | Salmonella enterica subsp. enterica serovar Newport str. SL254, complete genome. | Gammaproteobacteria | pts system fructose-specific eiibc component | [ACF61597](http://www.ncbi.nlm.nih.gov/protein/ACF61597.1) | 100.0 |
|  | | | | | | | |
|  | | | | | | | |
| ------------------ | | | | | | | |
|  | | | | | | | |
|  | | | | | | | |
| **Input Sequence** | SM-Roxy-RH01221661_S26_L001_R1_001_(paired)_contig_25_27 # 18610 # 19983 # -1 # ID=25_27;partial=00;start_type=GTG;rbs_motif=GGA/GAG/AGG;rbs_spacer=5-10bp;gc_cont=0.536 | | | | | | |
|  | PROJECT ID | ACCESSION ID | ORGANISMS | CLASS | PROTEIN FUNCTION | PROTEIN ID | %IDENTITY |
| **Matched Family** | [30687](http://www.ncbi.nlm.nih.gov/bioproject?db=bioproject&cmd=ShowDetailView&TermToSearch=30687) | [AM933172](http://www.ncbi.nlm.nih.gov/nuccore/AM933172) | Salmonella enterica subsp. enterica serovar Enteritidis str. P125109 complete genome. | Gammaproteobacteria | Multidrug-efflux transporter. | [CAR33204](http://www.ncbi.nlm.nih.gov/protein/CAR33204.1) | 100.0 |
|  | | | | | | | |
|  | | | | | | | |
| ------------------ | | | | | | | |
|  | | | | | | | |
|  | | | | | | | |
| **Input Sequence** | SM-Roxy-RH01221661_S26_L001_R1_001_(paired)_contig_30_92 # 89630 # 91003 # -1 # ID=30_92;partial=00;start_type=ATG;rbs_motif=None;rbs_spacer=None;gc_cont=0.549 | | | | | | |
|  | PROJECT ID | ACCESSION ID | ORGANISMS | CLASS | PROTEIN FUNCTION | PROTEIN ID | %IDENTITY |
| **Matched Family** | [18747](http://www.ncbi.nlm.nih.gov/bioproject?db=bioproject&cmd=ShowDetailView&TermToSearch=18747) | [CP001113](http://www.ncbi.nlm.nih.gov/nuccore/CP001113) | Salmonella enterica subsp. enterica serovar Newport str. SL254, complete genome. | Gammaproteobacteria | PTS family galactitol-specific enzyme IIC | [ACF65271](http://www.ncbi.nlm.nih.gov/protein/ACF65271.1) | 100.0 |
|  | | | | | | | |
|  | | | | | | | |
| ------------------ | | | | | | | |
|  | | | | | | | |
|  | | | | | | | |
| **Input Sequence** | SM-Roxy-RH01221661_S26_L001_R1_001_(paired)_contig_45_1 # 715 # 2088 # -1 # ID=45_1;partial=00;start_type=GTG;rbs_motif=GGA/GAG/AGG;rbs_spacer=5-10bp;gc_cont=0.580 | | | | | | |
|  | PROJECT ID | ACCESSION ID | ORGANISMS | CLASS | PROTEIN FUNCTION | PROTEIN ID | %IDENTITY |
| **Matched Family** | [20993](http://www.ncbi.nlm.nih.gov/bioproject?db=bioproject&cmd=ShowDetailView&TermToSearch=20993) | [CP000857](http://www.ncbi.nlm.nih.gov/nuccore/CP000857) | Salmonella enterica subsp. enterica serovar Paratyphi C strain RKS4594, complete genome. | Gammaproteobacteria | siroheme synthase | [ACN47630](http://www.ncbi.nlm.nih.gov/protein/ACN47630.1) | 100.0 |
|  | | | | | | | |
|  | | | | | | | |
| ------------------ | | | | | | | |
|  | | | | | | | |
|  | | | | | | | |
| **Input Sequence** | SM-Roxy-RH01221661_S26_L001_R1_001_(paired)_contig_44_60 # 61738 # 63111 # 1 # ID=44_60;partial=00;start_type=ATG;rbs_motif=GGAG/GAGG;rbs_spacer=5-10bp;gc_cont=0.400 | | | | | | |
|  | PROJECT ID | ACCESSION ID | ORGANISMS | CLASS | PROTEIN FUNCTION | PROTEIN ID | %IDENTITY |
| **Matched Family** | [30687](http://www.ncbi.nlm.nih.gov/bioproject?db=bioproject&cmd=ShowDetailView&TermToSearch=30687) | [AM933172](http://www.ncbi.nlm.nih.gov/nuccore/AM933172) | Salmonella enterica subsp. enterica serovar Enteritidis str. P125109 complete genome. | Gammaproteobacteria | hypothetical protein | [CAR32912](http://www.ncbi.nlm.nih.gov/protein/CAR32912.1) | 100.0 |
|  | | | | | | | |
|  | | | | | | | |
| ------------------ | | | | | | | |
|  | | | | | | | |
|  | | | | | | | |
| **Input Sequence** | SM-Roxy-RH01221661_S26_L001_R1_001_(paired)_contig_48_20 # 17863 # 19233 # 1 # ID=48_20;partial=00;start_type=ATG;rbs_motif=GGA/GAG/AGG;rbs_spacer=5-10bp;gc_cont=0.472 | | | | | | |
|  | PROJECT ID | ACCESSION ID | ORGANISMS | CLASS | PROTEIN FUNCTION | PROTEIN ID | %IDENTITY |
| **Matched Family** | [18747](http://www.ncbi.nlm.nih.gov/bioproject?db=bioproject&cmd=ShowDetailView&TermToSearch=18747) | [CP001113](http://www.ncbi.nlm.nih.gov/nuccore/CP001113) | Salmonella enterica subsp. enterica serovar Newport str. SL254, complete genome. | Gammaproteobacteria | putative glycoporin | [ACF64926](http://www.ncbi.nlm.nih.gov/protein/ACF64926.1) | 100.0 |
|  | | | | | | | |
|  | | | | | | | |
| ------------------ | | | | | | | |
|  | | | | | | | |
|  | | | | | | | |
| **Input Sequence** | SM-Roxy-RH01221661_S26_L001_R1_001_(paired)_contig_26_22 # 24996 # 26363 # 1 # ID=26_22;partial=00;start_type=ATG;rbs_motif=GGA/GAG/AGG;rbs_spacer=5-10bp;gc_cont=0.606 | | | | | | |
|  | PROJECT ID | ACCESSION ID | ORGANISMS | CLASS | PROTEIN FUNCTION | PROTEIN ID | %IDENTITY |
| **Matched Family** | [30687](http://www.ncbi.nlm.nih.gov/bioproject?db=bioproject&cmd=ShowDetailView&TermToSearch=30687) | [AM933172](http://www.ncbi.nlm.nih.gov/nuccore/AM933172) | Salmonella enterica subsp. enterica serovar Enteritidis str. P125109 complete genome. | Gammaproteobacteria | O-succinylbenzoic acid-CoA ligase | [CAR33871](http://www.ncbi.nlm.nih.gov/protein/CAR33871.1) | 100.0 |
|  | | | | | | | |
|  | | | | | | | |
| ------------------ | | | | | | | |
|  | | | | | | | |
|  | | | | | | | |
| **Input Sequence** | SM-Roxy-RH01221661_S26_L001_R1_001_(paired)_contig_42_54 # 51391 # 52755 # 1 # ID=42_54;partial=00;start_type=ATG;rbs_motif=GGA/GAG/AGG;rbs_spacer=11-12bp;gc_cont=0.512 | | | | | | |
|  | PROJECT ID | ACCESSION ID | ORGANISMS | CLASS | PROTEIN FUNCTION | PROTEIN ID | %IDENTITY |
| **Matched Family** | [27803](http://www.ncbi.nlm.nih.gov/bioproject?db=bioproject&cmd=ShowDetailView&TermToSearch=27803) | [CP000886](http://www.ncbi.nlm.nih.gov/nuccore/CP000886) | Salmonella enterica subsp. enterica serovar Paratyphi B str. SPB7, complete genome. | Gammaproteobacteria | hypothetical protein | [ABX67841](http://www.ncbi.nlm.nih.gov/protein/ABX67841.1) | 100.0 |
|  | | | | | | | |
|  | | | | | | | |
| ------------------ | | | | | | | |
|  | | | | | | | |
|  | | | | | | | |
| **Input Sequence** | SM-Roxy-RH01221661_S26_L001_R1_001_(paired)_contig_54_33 # 31357 # 32718 # 1 # ID=54_33;partial=00;start_type=ATG;rbs_motif=GGAG/GAGG;rbs_spacer=5-10bp;gc_cont=0.560 | | | | | | |
|  | PROJECT ID | ACCESSION ID | ORGANISMS | CLASS | PROTEIN FUNCTION | PROTEIN ID | %IDENTITY |
| **Matched Family** | [30687](http://www.ncbi.nlm.nih.gov/bioproject?db=bioproject&cmd=ShowDetailView&TermToSearch=30687) | [AM933172](http://www.ncbi.nlm.nih.gov/nuccore/AM933172) | Salmonella enterica subsp. enterica serovar Enteritidis str. P125109 complete genome. | Gammaproteobacteria | putative PTS system IIBC component | [CAR34132](http://www.ncbi.nlm.nih.gov/protein/CAR34132.1) | 100.0 |
|  | | | | | | | |
|  | | | | | | | |
| ------------------ | | | | | | | |
|  | | | | | | | |
|  | | | | | | | |
| **Input Sequence** | SM-Roxy-RH01221661_S26_L001_R1_001_(paired)_contig_12_183 # 176942 # 178300 # 1 # ID=12_183;partial=00;start_type=ATG;rbs_motif=GGA/GAG/AGG;rbs_spacer=5-10bp;gc_cont=0.510 | | | | | | |
|  | PROJECT ID | ACCESSION ID | ORGANISMS | CLASS | PROTEIN FUNCTION | PROTEIN ID | %IDENTITY |
| **Matched Family** | [19459](http://www.ncbi.nlm.nih.gov/bioproject?db=bioproject&cmd=ShowDetailView&TermToSearch=19459) | [CP001127](http://www.ncbi.nlm.nih.gov/nuccore/CP001127) | Salmonella enterica subsp. enterica serovar Schwarzengrund str. CVM19633, complete genome. | Gammaproteobacteria | PTS system, N,N'-diacetylchitobiose-specific IIC component | [ACF91099](http://www.ncbi.nlm.nih.gov/protein/ACF91099.1) | 100.0 |
|  | | | | | | | |
|  | | | | | | | |
| ------------------ | | | | | | | |
|  | | | | | | | |
|  | | | | | | | |
| **Input Sequence** | SM-Roxy-RH01221661_S26_L001_R1_001_(paired)_contig_21_262 # 264619 # 265977 # -1 # ID=21_262;partial=00;start_type=ATG;rbs_motif=GGAG/GAGG;rbs_spacer=5-10bp;gc_cont=0.588 | | | | | | |
|  | PROJECT ID | ACCESSION ID | ORGANISMS | CLASS | PROTEIN FUNCTION | PROTEIN ID | %IDENTITY |
| **Matched Family** | [20063](http://www.ncbi.nlm.nih.gov/bioproject?db=bioproject&cmd=ShowDetailView&TermToSearch=20063) | [CP001138](http://www.ncbi.nlm.nih.gov/nuccore/CP001138) | Salmonella enterica subsp. enterica serovar Agona str. SL483, complete genome. | Gammaproteobacteria | 4-hydroxybenzoate transporter | [ACH51569](http://www.ncbi.nlm.nih.gov/protein/ACH51569.1) | 100.0 |
|  | | | | | | | |
|  | | | | | | | |
| ------------------ | | | | | | | |
|  | | | | | | | |
|  | | | | | | | |
| **Input Sequence** | SM-Roxy-RH01221661_S26_L001_R1_001_(paired)_contig_12_186 # 179676 # 181031 # 1 # ID=12_186;partial=00;start_type=ATG;rbs_motif=GGAG/GAGG;rbs_spacer=5-10bp;gc_cont=0.480 | | | | | | |
|  | PROJECT ID | ACCESSION ID | ORGANISMS | CLASS | PROTEIN FUNCTION | PROTEIN ID | %IDENTITY |
| **Matched Family** | [30687](http://www.ncbi.nlm.nih.gov/bioproject?db=bioproject&cmd=ShowDetailView&TermToSearch=30687) | [AM933172](http://www.ncbi.nlm.nih.gov/nuccore/AM933172) | Salmonella enterica subsp. enterica serovar Enteritidis str. P125109 complete genome. | Gammaproteobacteria | phospho-beta-glucosidase B | [CAR33308](http://www.ncbi.nlm.nih.gov/protein/CAR33308.1) | 100.0 |
|  | | | | | | | |
|  | | | | | | | |
| ------------------ | | | | | | | |
|  | | | | | | | |
|  | | | | | | | |
| **Input Sequence** | SM-Roxy-RH01221661_S26_L001_R1_001_(paired)_contig_21_136 # 117838 # 119193 # 1 # ID=21_136;partial=00;start_type=ATG;rbs_motif=GGAGG;rbs_spacer=5-10bp;gc_cont=0.617 | | | | | | |
|  | PROJECT ID | ACCESSION ID | ORGANISMS | CLASS | PROTEIN FUNCTION | PROTEIN ID | %IDENTITY |
| **Matched Family** | [30687](http://www.ncbi.nlm.nih.gov/bioproject?db=bioproject&cmd=ShowDetailView&TermToSearch=30687) | [AM933172](http://www.ncbi.nlm.nih.gov/nuccore/AM933172) | Salmonella enterica subsp. enterica serovar Enteritidis str. P125109 complete genome. | Gammaproteobacteria | propanediol utilization ferredoxin | [CAR33631](http://www.ncbi.nlm.nih.gov/protein/CAR33631.1) | 100.0 |
|  | | | | | | | |
|  | | | | | | | |
| ------------------ | | | | | | | |
|  | | | | | | | |
|  | | | | | | | |
| **Input Sequence** | SM-Roxy-RH01221661_S26_L001_R1_001_(paired)_contig_23_84 # 85923 # 87272 # 1 # ID=23_84;partial=00;start_type=ATG;rbs_motif=AGGAG;rbs_spacer=5-10bp;gc_cont=0.534 | | | | | | |
|  | PROJECT ID | ACCESSION ID | ORGANISMS | CLASS | PROTEIN FUNCTION | PROTEIN ID | %IDENTITY |
| **Matched Family** | [30687](http://www.ncbi.nlm.nih.gov/bioproject?db=bioproject&cmd=ShowDetailView&TermToSearch=30687) | [AM933172](http://www.ncbi.nlm.nih.gov/nuccore/AM933172) | Salmonella enterica subsp. enterica serovar Enteritidis str. P125109 complete genome. | Gammaproteobacteria | inner membrane protein | [CAR35899](http://www.ncbi.nlm.nih.gov/protein/CAR35899.1) | 100.0 |
|  | | | | | | | |
|  | | | | | | | |
| ------------------ | | | | | | | |
|  | | | | | | | |
|  | | | | | | | |
| **Input Sequence** | SM-Roxy-RH01221661_S26_L001_R1_001_(paired)_contig_53_2 # 431 # 1771 # -1 # ID=53_2;partial=00;start_type=ATG;rbs_motif=AGGA;rbs_spacer=5-10bp;gc_cont=0.491 | | | | | | |
|  | PROJECT ID | ACCESSION ID | ORGANISMS | CLASS | PROTEIN FUNCTION | PROTEIN ID | %IDENTITY |
| **Matched Family** | [20993](http://www.ncbi.nlm.nih.gov/bioproject?db=bioproject&cmd=ShowDetailView&TermToSearch=20993) | [CP000857](http://www.ncbi.nlm.nih.gov/nuccore/CP000857) | Salmonella enterica subsp. enterica serovar Paratyphi C strain RKS4594, complete genome. | Gammaproteobacteria | citrate-sodium symporter | [ACN44253](http://www.ncbi.nlm.nih.gov/protein/ACN44253.1) | 100.0 |
|  | | | | | | | |
|  | | | | | | | |
| ------------------ | | | | | | | |
|  | | | | | | | |
|  | | | | | | | |
| **Input Sequence** | SM-Roxy-RH01221661_S26_L001_R1_001_(paired)_contig_42_178 # 198113 # 199456 # -1 # ID=42_178;partial=00;start_type=ATG;rbs_motif=GGA/GAG/AGG;rbs_spacer=5-10bp;gc_cont=0.459 | | | | | | |
|  | PROJECT ID | ACCESSION ID | ORGANISMS | CLASS | PROTEIN FUNCTION | PROTEIN ID | %IDENTITY |
| **Matched Family** | [18747](http://www.ncbi.nlm.nih.gov/bioproject?db=bioproject&cmd=ShowDetailView&TermToSearch=18747) | [CP001113](http://www.ncbi.nlm.nih.gov/nuccore/CP001113) | Salmonella enterica subsp. enterica serovar Newport str. SL254, complete genome. | Gammaproteobacteria | ascorbate-specific PTS system enzyme IIC | [ACF63181](http://www.ncbi.nlm.nih.gov/protein/ACF63181.1) | 100.0 |
|  | | | | | | | |
|  | | | | | | | |
| ------------------ | | | | | | | |
|  | | | | | | | |
|  | | | | | | | |
| **Input Sequence** | SM-Roxy-RH01221661_S26_L001_R1_001_(paired)_contig_12_247 # 246610 # 247953 # -1 # ID=12_247;partial=00;start_type=ATG;rbs_motif=GGA/GAG/AGG;rbs_spacer=5-10bp;gc_cont=0.509 | | | | | | |
|  | PROJECT ID | ACCESSION ID | ORGANISMS | CLASS | PROTEIN FUNCTION | PROTEIN ID | %IDENTITY |
| **Matched Family** | [20063](http://www.ncbi.nlm.nih.gov/bioproject?db=bioproject&cmd=ShowDetailView&TermToSearch=20063) | [CP001138](http://www.ncbi.nlm.nih.gov/nuccore/CP001138) | Salmonella enterica subsp. enterica serovar Agona str. SL483, complete genome. | Gammaproteobacteria | amino acid transporter | [ACH50402](http://www.ncbi.nlm.nih.gov/protein/ACH50402.1) | 100.0 |
|  | | | | | | | |
|  | | | | | | | |
| ------------------ | | | | | | | |
|  | | | | | | | |
|  | | | | | | | |
| **Input Sequence** | SM-Roxy-RH01221661_S26_L001_R1_001_(paired)_contig_10_17 # 21454 # 22791 # 1 # ID=10_17;partial=00;start_type=ATG;rbs_motif=AGGAG;rbs_spacer=5-10bp;gc_cont=0.567 | | | | | | |
|  | PROJECT ID | ACCESSION ID | ORGANISMS | CLASS | PROTEIN FUNCTION | PROTEIN ID | %IDENTITY |
| **Matched Family** | [13086](http://www.ncbi.nlm.nih.gov/bioproject?db=bioproject&cmd=ShowDetailView&TermToSearch=13086) | [CP000026](http://www.ncbi.nlm.nih.gov/nuccore/CP000026) | Salmonella enterica subsp. enterica serovar Paratyphi A str. ATCC 9150, complete genome. | Gammaproteobacteria | putative amino acid permease | [AAV79856](http://www.ncbi.nlm.nih.gov/protein/AAV79856.1) | 100.0 |
|  | | | | | | | |
|  | | | | | | | |
| ------------------ | | | | | | | |
|  | | | | | | | |
|  | | | | | | | |
| **Input Sequence** | SM-Roxy-RH01221661_S26_L001_R1_001_(paired)_contig_4_121 # 142686 # 144020 # -1 # ID=4_121;partial=00;start_type=ATG;rbs_motif=GGA/GAG/AGG;rbs_spacer=5-10bp;gc_cont=0.542 | | | | | | |
|  | PROJECT ID | ACCESSION ID | ORGANISMS | CLASS | PROTEIN FUNCTION | PROTEIN ID | %IDENTITY |
| **Matched Family** | [18747](http://www.ncbi.nlm.nih.gov/bioproject?db=bioproject&cmd=ShowDetailView&TermToSearch=18747) | [CP001113](http://www.ncbi.nlm.nih.gov/nuccore/CP001113) | Salmonella enterica subsp. enterica serovar Newport str. SL254, complete genome. | Gammaproteobacteria | major facilitator superfamily MF | [ACF63973](http://www.ncbi.nlm.nih.gov/protein/ACF63973.1) | 100.0 |
|  | | | | | | | |
|  | | | | | | | |
| ------------------ | | | | | | | |
|  | | | | | | | |
|  | | | | | | | |
| **Input Sequence** | SM-Roxy-RH01221661_S26_L001_R1_001_(paired)_contig_12_94 # 90016 # 91350 # 1 # ID=12_94;partial=00;start_type=ATG;rbs_motif=None;rbs_spacer=None;gc_cont=0.476 | | | | | | |
|  | PROJECT ID | ACCESSION ID | ORGANISMS | CLASS | PROTEIN FUNCTION | PROTEIN ID | %IDENTITY |
| **Matched Family** | [30687](http://www.ncbi.nlm.nih.gov/bioproject?db=bioproject&cmd=ShowDetailView&TermToSearch=30687) | [AM933172](http://www.ncbi.nlm.nih.gov/nuccore/AM933172) | Salmonella enterica subsp. enterica serovar Enteritidis str. P125109 complete genome. | Gammaproteobacteria | hypothetical protein | [CAR33401](http://www.ncbi.nlm.nih.gov/protein/CAR33401.1) | 100.0 |
|  | | | | | | | |
|  | | | | | | | |
| ------------------ | | | | | | | |
|  | | | | | | | |
|  | | | | | | | |
| **Input Sequence** | SM-Roxy-RH01221661_S26_L001_R1_001_(paired)_contig_2_13 # 9676 # 11001 # -1 # ID=2_13;partial=00;start_type=ATG;rbs_motif=AGGA;rbs_spacer=5-10bp;gc_cont=0.521 | | | | | | |
|  | PROJECT ID | ACCESSION ID | ORGANISMS | CLASS | PROTEIN FUNCTION | PROTEIN ID | %IDENTITY |
| **Matched Family** | [30687](http://www.ncbi.nlm.nih.gov/bioproject?db=bioproject&cmd=ShowDetailView&TermToSearch=30687) | [AM933172](http://www.ncbi.nlm.nih.gov/nuccore/AM933172) | Salmonella enterica subsp. enterica serovar Enteritidis str. P125109 complete genome. | Gammaproteobacteria | putative fimbrial protein | [CAR31906](http://www.ncbi.nlm.nih.gov/protein/CAR31906.1) | 100.0 |
|  | | | | | | | |
|  | | | | | | | |
| ------------------ | | | | | | | |
|  | | | | | | | |
|  | | | | | | | |
| **Input Sequence** | SM-Roxy-RH01221661_S26_L001_R1_001_(paired)_contig_11_73 # 74831 # 76156 # 1 # ID=11_73;partial=00;start_type=ATG;rbs_motif=GGAG/GAGG;rbs_spacer=5-10bp;gc_cont=0.452 | | | | | | |
|  | PROJECT ID | ACCESSION ID | ORGANISMS | CLASS | PROTEIN FUNCTION | PROTEIN ID | %IDENTITY |
| **Matched Family** | [18747](http://www.ncbi.nlm.nih.gov/bioproject?db=bioproject&cmd=ShowDetailView&TermToSearch=18747) | [CP001113](http://www.ncbi.nlm.nih.gov/nuccore/CP001113) | Salmonella enterica subsp. enterica serovar Newport str. SL254, complete genome. | Gammaproteobacteria | 4-hydroxybutyrate coenzyme A transferase | [ACF61587](http://www.ncbi.nlm.nih.gov/protein/ACF61587.1) | 100.0 |
|  | | | | | | | |
|  | | | | | | | |
| ------------------ | | | | | | | |
|  | | | | | | | |
|  | | | | | | | |
| **Input Sequence** | SM-Roxy-RH01221661_S26_L001_R1_001_(paired)_contig_4_20 # 19767 # 21092 # 1 # ID=4_20;partial=00;start_type=ATG;rbs_motif=GGAG/GAGG;rbs_spacer=5-10bp;gc_cont=0.550 | | | | | | |
|  | PROJECT ID | ACCESSION ID | ORGANISMS | CLASS | PROTEIN FUNCTION | PROTEIN ID | %IDENTITY |
| **Matched Family** | [241](http://www.ncbi.nlm.nih.gov/bioproject?db=bioproject&cmd=ShowDetailView&TermToSearch=241) | [AE006468](http://www.ncbi.nlm.nih.gov/nuccore/AE006468) | Salmonella enterica subsp. enterica serovar Typhimurium str. LT2, complete genome. | Gammaproteobacteria | putative xanthine permease | [AAL22492](http://www.ncbi.nlm.nih.gov/protein/AAL22492.1) | 100.0 |
|  | | | | | | | |
|  | | | | | | | |
| ------------------ | | | | | | | |
|  | | | | | | | |
|  | | | | | | | |
| **Input Sequence** | SM-Roxy-RH01221661_S26_L001_R1_001_(paired)_contig_24_19 # 21688 # 23013 # -1 # ID=24_19;partial=00;start_type=ATG;rbs_motif=GGAG/GAGG;rbs_spacer=5-10bp;gc_cont=0.529 | | | | | | |
|  | PROJECT ID | ACCESSION ID | ORGANISMS | CLASS | PROTEIN FUNCTION | PROTEIN ID | %IDENTITY |
| **Matched Family** | [30687](http://www.ncbi.nlm.nih.gov/bioproject?db=bioproject&cmd=ShowDetailView&TermToSearch=30687) | [AM933172](http://www.ncbi.nlm.nih.gov/nuccore/AM933172) | Salmonella enterica subsp. enterica serovar Enteritidis str. P125109 complete genome. | Gammaproteobacteria | probable pyridine nucleotide-disulfide oxidoreductase | [CAR32128](http://www.ncbi.nlm.nih.gov/protein/CAR32128.1) | 100.0 |
|  | | | | | | | |
|  | | | | | | | |
| ------------------ | | | | | | | |
|  | | | | | | | |
|  | | | | | | | |
| **Input Sequence** | SM-Roxy-RH01221661_S26_L001_R1_001_(paired)_contig_65_9 # 14905 # 16230 # -1 # ID=65_9;partial=00;start_type=ATG;rbs_motif=GGA/GAG/AGG;rbs_spacer=5-10bp;gc_cont=0.419 | | | | | | |
|  | PROJECT ID | ACCESSION ID | ORGANISMS | CLASS | PROTEIN FUNCTION | PROTEIN ID | %IDENTITY |
| **Matched Family** | [30687](http://www.ncbi.nlm.nih.gov/bioproject?db=bioproject&cmd=ShowDetailView&TermToSearch=30687) | [AM933172](http://www.ncbi.nlm.nih.gov/nuccore/AM933172) | Salmonella enterica subsp. enterica serovar Enteritidis str. P125109 complete genome. | Gammaproteobacteria | putative transmembrane transpot protein | [CAR33840](http://www.ncbi.nlm.nih.gov/protein/CAR33840.1) | 100.0 |
|  | | | | | | | |
|  | | | | | | | |
| ------------------ | | | | | | | |
|  | | | | | | | |
|  | | | | | | | |
| **Input Sequence** | SM-Roxy-RH01221661_S26_L001_R1_001_(paired)_contig_31_25 # 24058 # 25377 # 1 # ID=31_25;partial=00;start_type=ATG;rbs_motif=AGGAG;rbs_spacer=5-10bp;gc_cont=0.367 | | | | | | |
|  | PROJECT ID | ACCESSION ID | ORGANISMS | CLASS | PROTEIN FUNCTION | PROTEIN ID | %IDENTITY |
| **Matched Family** | [20993](http://www.ncbi.nlm.nih.gov/bioproject?db=bioproject&cmd=ShowDetailView&TermToSearch=20993) | [CP000857](http://www.ncbi.nlm.nih.gov/nuccore/CP000857) | Salmonella enterica subsp. enterica serovar Paratyphi C strain RKS4594, complete genome. | Gammaproteobacteria | putative ABC exporter outer membrane component-like protein | [ACN48385](http://www.ncbi.nlm.nih.gov/protein/ACN48385.1) | 100.0 |
|  | | | | | | | |
|  | | | | | | | |
| ------------------ | | | | | | | |
|  | | | | | | | |
|  | | | | | | | |
| **Input Sequence** | SM-Roxy-RH01221661_S26_L001_R1_001_(paired)_contig_81_1 # 107 # 880 # 1 # ID=81_1;partial=00;start_type=ATG;rbs_motif=GGA/GAG/AGG;rbs_spacer=5-10bp;gc_cont=0.557 | | | | | | |
|  | PROJECT ID | ACCESSION ID | ORGANISMS | CLASS | PROTEIN FUNCTION | PROTEIN ID | %IDENTITY |
| **Matched Family** | [13086](http://www.ncbi.nlm.nih.gov/bioproject?db=bioproject&cmd=ShowDetailView&TermToSearch=13086) | [CP000026](http://www.ncbi.nlm.nih.gov/nuccore/CP000026) | Salmonella enterica subsp. enterica serovar Paratyphi A str. ATCC 9150, complete genome. | Gammaproteobacteria | probable permease | [AAV79436](http://www.ncbi.nlm.nih.gov/protein/AAV79436.1) | 100.0 |
|  | | | | | | | |
|  | | | | | | | |
| ------------------ | | | | | | | |
|  | | | | | | | |
|  | | | | | | | |
| **Input Sequence** | SM-Roxy-RH01221661_S26_L001_R1_001_(paired)_contig_25_211 # 215256 # 216569 # 1 # ID=25_211;partial=00;start_type=ATG;rbs_motif=AGxAGG/AGGxGG;rbs_spacer=3-4bp;gc_cont=0.521 | | | | | | |
|  | PROJECT ID | ACCESSION ID | ORGANISMS | CLASS | PROTEIN FUNCTION | PROTEIN ID | %IDENTITY |
| **Matched Family** | [13086](http://www.ncbi.nlm.nih.gov/bioproject?db=bioproject&cmd=ShowDetailView&TermToSearch=13086) | [CP000026](http://www.ncbi.nlm.nih.gov/nuccore/CP000026) | Salmonella enterica subsp. enterica serovar Paratyphi A str. ATCC 9150, complete genome. | Gammaproteobacteria | putative phosphotransferase enzyme | [AAV77206](http://www.ncbi.nlm.nih.gov/protein/AAV77206.1) | 100.0 |
|  | | | | | | | |
|  | | | | | | | |
| ------------------ | | | | | | | |
|  | | | | | | | |
|  | | | | | | | |
| **Input Sequence** | SM-Roxy-RH01221661_S26_L001_R1_001_(paired)_contig_21_174 # 161254 # 162567 # -1 # ID=21_174;partial=00;start_type=ATG;rbs_motif=AGGA;rbs_spacer=5-10bp;gc_cont=0.444 | | | | | | |
|  | PROJECT ID | ACCESSION ID | ORGANISMS | CLASS | PROTEIN FUNCTION | PROTEIN ID | %IDENTITY |
| **Matched Family** | [19459](http://www.ncbi.nlm.nih.gov/bioproject?db=bioproject&cmd=ShowDetailView&TermToSearch=19459) | [CP001127](http://www.ncbi.nlm.nih.gov/nuccore/CP001127) | Salmonella enterica subsp. enterica serovar Schwarzengrund str. CVM19633, complete genome. | Gammaproteobacteria | lipopolysaccharide biosynthesis protein RfbH | [ACF89467](http://www.ncbi.nlm.nih.gov/protein/ACF89467.1) | 100.0 |
|  | | | | | | | |
|  | | | | | | | |
| ------------------ | | | | | | | |
|  | | | | | | | |
|  | | | | | | | |
| **Input Sequence** | SM-Roxy-RH01221661_S26_L001_R1_001_(paired)_contig_31_9 # 6756 # 8066 # 1 # ID=31_9;partial=00;start_type=GTG;rbs_motif=None;rbs_spacer=None;gc_cont=0.523 | | | | | | |
|  | PROJECT ID | ACCESSION ID | ORGANISMS | CLASS | PROTEIN FUNCTION | PROTEIN ID | %IDENTITY |
| **Matched Family** | [18747](http://www.ncbi.nlm.nih.gov/bioproject?db=bioproject&cmd=ShowDetailView&TermToSearch=18747) | [CP001113](http://www.ncbi.nlm.nih.gov/nuccore/CP001113) | Salmonella enterica subsp. enterica serovar Newport str. SL254, complete genome. | Gammaproteobacteria | conjugative transfer protein | [ACF63659](http://www.ncbi.nlm.nih.gov/protein/ACF63659.1) | 100.0 |
|  | | | | | | | |
|  | | | | | | | |
| ------------------ | | | | | | | |
|  | | | | | | | |
|  | | | | | | | |
| **Input Sequence** | SM-Roxy-RH01221661_S26_L001_R1_001_(paired)_contig_36_24 # 28294 # 29592 # -1 # ID=36_24;partial=00;start_type=ATG;rbs_motif=GGAG/GAGG;rbs_spacer=5-10bp;gc_cont=0.460 | | | | | | |
|  | PROJECT ID | ACCESSION ID | ORGANISMS | CLASS | PROTEIN FUNCTION | PROTEIN ID | %IDENTITY |
| **Matched Family** | [30687](http://www.ncbi.nlm.nih.gov/bioproject?db=bioproject&cmd=ShowDetailView&TermToSearch=30687) | [AM933172](http://www.ncbi.nlm.nih.gov/nuccore/AM933172) | Salmonella enterica subsp. enterica serovar Enteritidis str. P125109 complete genome. | Gammaproteobacteria | putative exported protein | [CAR34042](http://www.ncbi.nlm.nih.gov/protein/CAR34042.1) | 100.0 |
|  | | | | | | | |
|  | | | | | | | |
| ------------------ | | | | | | | |
|  | | | | | | | |
|  | | | | | | | |
| **Input Sequence** | SM-Roxy-RH01221661_S26_L001_R1_001_(paired)_contig_24_56 # 56624 # 57922 # -1 # ID=24_56;partial=00;start_type=ATG;rbs_motif=GGA/GAG/AGG;rbs_spacer=5-10bp;gc_cont=0.513 | | | | | | |
|  | PROJECT ID | ACCESSION ID | ORGANISMS | CLASS | PROTEIN FUNCTION | PROTEIN ID | %IDENTITY |
| **Matched Family** | [19467](http://www.ncbi.nlm.nih.gov/bioproject?db=bioproject&cmd=ShowDetailView&TermToSearch=19467) | [CP001144](http://www.ncbi.nlm.nih.gov/nuccore/CP001144) | Salmonella enterica subsp. enterica serovar Dublin str. C | Gammaproteobacteria | 2021853, complete genome. | [ACH74024](http://www.ncbi.nlm.nih.gov/protein/ACH74024.1) | 100.0 |
|  | | | | | | | |
|  | | | | | | | |
| ------------------ | | | | | | | |
|  | | | | | | | |
|  | | | | | | | |
| **Input Sequence** | SM-Roxy-RH01221661_S26_L001_R1_001_(paired)_contig_3_11 # 9293 # 10585 # -1 # ID=3_11;partial=00;start_type=ATG;rbs_motif=GGA/GAG/AGG;rbs_spacer=5-10bp;gc_cont=0.589 | | | | | | |
|  | PROJECT ID | ACCESSION ID | ORGANISMS | CLASS | PROTEIN FUNCTION | PROTEIN ID | %IDENTITY |
| **Matched Family** | [30687](http://www.ncbi.nlm.nih.gov/bioproject?db=bioproject&cmd=ShowDetailView&TermToSearch=30687) | [AM933172](http://www.ncbi.nlm.nih.gov/nuccore/AM933172) | Salmonella enterica subsp. enterica serovar Enteritidis str. P125109 complete genome. | Gammaproteobacteria | putative transcriptional regulator | [CAR34002](http://www.ncbi.nlm.nih.gov/protein/CAR34002.1) | 100.0 |
|  | | | | | | | |
|  | | | | | | | |
| ------------------ | | | | | | | |
|  | | | | | | | |
|  | | | | | | | |
| **Input Sequence** | SM-Roxy-RH01221661_S26_L001_R1_001_(paired)_contig_25_91 # 83675 # 84928 # -1 # ID=25_91;partial=00;start_type=ATG;rbs_motif=GGA/GAG/AGG;rbs_spacer=5-10bp;gc_cont=0.581 | | | | | | |
|  | PROJECT ID | ACCESSION ID | ORGANISMS | CLASS | PROTEIN FUNCTION | PROTEIN ID | %IDENTITY |
| **Matched Family** | [30687](http://www.ncbi.nlm.nih.gov/bioproject?db=bioproject&cmd=ShowDetailView&TermToSearch=30687) | [AM933172](http://www.ncbi.nlm.nih.gov/nuccore/AM933172) | Salmonella enterica subsp. enterica serovar Enteritidis str. P125109 complete genome. | Gammaproteobacteria | putative voltage gated chloride channel protein | [CAR33140](http://www.ncbi.nlm.nih.gov/protein/CAR33140.1) | 100.0 |
|  | | | | | | | |
|  | | | | | | | |
| ------------------ | | | | | | | |
|  | | | | | | | |
|  | | | | | | | |
| **Input Sequence** | SM-Roxy-RH01221661_S26_L001_R1_001_(paired)_contig_36_9 # 8891 # 10159 # 1 # ID=36_9;partial=00;start_type=ATG;rbs_motif=AGGAG;rbs_spacer=5-10bp;gc_cont=0.565 | | | | | | |
|  | PROJECT ID | ACCESSION ID | ORGANISMS | CLASS | PROTEIN FUNCTION | PROTEIN ID | %IDENTITY |
| **Matched Family** | [30687](http://www.ncbi.nlm.nih.gov/bioproject?db=bioproject&cmd=ShowDetailView&TermToSearch=30687) | [AM933172](http://www.ncbi.nlm.nih.gov/nuccore/AM933172) | Salmonella enterica subsp. enterica serovar Enteritidis str. P125109 complete genome. | Gammaproteobacteria | putative permease | [CAR34057](http://www.ncbi.nlm.nih.gov/protein/CAR34057.1) | 100.0 |
|  | | | | | | | |
|  | | | | | | | |
| ------------------ | | | | | | | |
|  | | | | | | | |
|  | | | | | | | |
| **Input Sequence** | SM-Roxy-RH01221661_S26_L001_R1_001_(paired)_contig_42_48 # 45525 # 46814 # -1 # ID=42_48;partial=00;start_type=ATG;rbs_motif=AGGAG;rbs_spacer=5-10bp;gc_cont=0.560 | | | | | | |
|  | PROJECT ID | ACCESSION ID | ORGANISMS | CLASS | PROTEIN FUNCTION | PROTEIN ID | %IDENTITY |
| **Matched Family** | [19459](http://www.ncbi.nlm.nih.gov/bioproject?db=bioproject&cmd=ShowDetailView&TermToSearch=19459) | [CP001127](http://www.ncbi.nlm.nih.gov/nuccore/CP001127) | Salmonella enterica subsp. enterica serovar Schwarzengrund str. CVM19633, complete genome. | Gammaproteobacteria | 4-hydroxyphenylacetate degradation bifunctionalisomerase/decarboxylase | [ACF92812](http://www.ncbi.nlm.nih.gov/protein/ACF92812.1) | 100.0 |
|  | | | | | | | |
|  | | | | | | | |
| ------------------ | | | | | | | |
|  | | | | | | | |
|  | | | | | | | |
| **Input Sequence** | SM-Roxy-RH01221661_S26_L001_R1_001_(paired)_contig_25_144 # 134893 # 136179 # -1 # ID=25_144;partial=00;start_type=ATG;rbs_motif=GGAG/GAGG;rbs_spacer=5-10bp;gc_cont=0.466 | | | | | | |
|  | PROJECT ID | ACCESSION ID | ORGANISMS | CLASS | PROTEIN FUNCTION | PROTEIN ID | %IDENTITY |
| **Matched Family** | [19467](http://www.ncbi.nlm.nih.gov/bioproject?db=bioproject&cmd=ShowDetailView&TermToSearch=19467) | [CP001144](http://www.ncbi.nlm.nih.gov/nuccore/CP001144) | Salmonella enterica subsp. enterica serovar Dublin str. C | Gammaproteobacteria | 2021853, complete genome. | [ACH76212](http://www.ncbi.nlm.nih.gov/protein/ACH76212.1) | 100.0 |
|  | | | | | | | |
|  | | | | | | | |
| ------------------ | | | | | | | |
|  | | | | | | | |
|  | | | | | | | |
| **Input Sequence** | SM-Roxy-RH01221661_S26_L001_R1_001_(paired)_contig_38_18 # 17409 # 18692 # -1 # ID=38_18;partial=00;start_type=GTG;rbs_motif=GGAG/GAGG;rbs_spacer=5-10bp;gc_cont=0.575 | | | | | | |
|  | PROJECT ID | ACCESSION ID | ORGANISMS | CLASS | PROTEIN FUNCTION | PROTEIN ID | %IDENTITY |
| **Matched Family** | [13086](http://www.ncbi.nlm.nih.gov/bioproject?db=bioproject&cmd=ShowDetailView&TermToSearch=13086) | [CP000026](http://www.ncbi.nlm.nih.gov/nuccore/CP000026) | Salmonella enterica subsp. enterica serovar Paratyphi A str. ATCC 9150, complete genome. | Gammaproteobacteria | possible pectinesterase precursor | [AAV77874](http://www.ncbi.nlm.nih.gov/protein/AAV77874.1) | 100.0 |
|  | | | | | | | |
|  | | | | | | | |
| ------------------ | | | | | | | |
|  | | | | | | | |
|  | | | | | | | |
| **Input Sequence** | SM-Roxy-RH01221661_S26_L001_R1_001_(paired)_contig_21_269 # 270614 # 271855 # 1 # ID=21_269;partial=00;start_type=ATG;rbs_motif=AGGA;rbs_spacer=5-10bp;gc_cont=0.503 | | | | | | |
|  | PROJECT ID | ACCESSION ID | ORGANISMS | CLASS | PROTEIN FUNCTION | PROTEIN ID | %IDENTITY |
| **Matched Family** | [20993](http://www.ncbi.nlm.nih.gov/bioproject?db=bioproject&cmd=ShowDetailView&TermToSearch=20993) | [CP000857](http://www.ncbi.nlm.nih.gov/nuccore/CP000857) | Salmonella enterica subsp. enterica serovar Paratyphi C strain RKS4594, complete genome. | Gammaproteobacteria | putative oxidoreductase | [ACN45670](http://www.ncbi.nlm.nih.gov/protein/ACN45670.1) | 100.0 |
|  | | | | | | | |
|  | | | | | | | |
| ------------------ | | | | | | | |
|  | | | | | | | |
|  | | | | | | | |
| **Input Sequence** | SM-Roxy-RH01221661_S26_L001_R1_001_(paired)_contig_4_165 # 196792 # 198069 # 1 # ID=4_165;partial=00;start_type=ATG;rbs_motif=GGA/GAG/AGG;rbs_spacer=11-12bp;gc_cont=0.570 | | | | | | |
|  | PROJECT ID | ACCESSION ID | ORGANISMS | CLASS | PROTEIN FUNCTION | PROTEIN ID | %IDENTITY |
| **Matched Family** | [20063](http://www.ncbi.nlm.nih.gov/bioproject?db=bioproject&cmd=ShowDetailView&TermToSearch=20063) | [CP001138](http://www.ncbi.nlm.nih.gov/nuccore/CP001138) | Salmonella enterica subsp. enterica serovar Agona str. SL483, complete genome. | Gammaproteobacteria | protein DamX | [ACH49306](http://www.ncbi.nlm.nih.gov/protein/ACH49306.1) | 100.0 |
|  | | | | | | | |
|  | | | | | | | |
| ------------------ | | | | | | | |
|  | | | | | | | |
|  | | | | | | | |
| **Input Sequence** | SM-Roxy-RH01221661_S26_L001_R1_001_(paired)_contig_31_26 # 25374 # 26651 # 1 # ID=31_26;partial=00;start_type=ATG;rbs_motif=3Base/5BMM;rbs_spacer=13-15bp;gc_cont=0.345 | | | | | | |
|  | PROJECT ID | ACCESSION ID | ORGANISMS | CLASS | PROTEIN FUNCTION | PROTEIN ID | %IDENTITY |
| **Matched Family** | [13086](http://www.ncbi.nlm.nih.gov/bioproject?db=bioproject&cmd=ShowDetailView&TermToSearch=13086) | [CP000026](http://www.ncbi.nlm.nih.gov/nuccore/CP000026) | Salmonella enterica subsp. enterica serovar Paratyphi A str. ATCC 9150, complete genome. | Gammaproteobacteria | putative type-I secretion protein | [AAV79822](http://www.ncbi.nlm.nih.gov/protein/AAV79822.1) | 100.0 |
|  | | | | | | | |
|  | | | | | | | |
| ------------------ | | | | | | | |
|  | | | | | | | |
|  | | | | | | | |
| **Input Sequence** | SM-Roxy-RH01221661_S26_L001_R1_001_(paired)_contig_68_1 # 170 # 1375 # 1 # ID=68_1;partial=00;start_type=ATG;rbs_motif=GGAG/GAGG;rbs_spacer=5-10bp;gc_cont=0.572 | | | | | | |
|  | PROJECT ID | ACCESSION ID | ORGANISMS | CLASS | PROTEIN FUNCTION | PROTEIN ID | %IDENTITY |
| **Matched Family** | [30687](http://www.ncbi.nlm.nih.gov/bioproject?db=bioproject&cmd=ShowDetailView&TermToSearch=30687) | [AM933172](http://www.ncbi.nlm.nih.gov/nuccore/AM933172) | Salmonella enterica subsp. enterica serovar Enteritidis str. P125109 complete genome. | Gammaproteobacteria | putative membrane protein | [CAR32628](http://www.ncbi.nlm.nih.gov/protein/CAR32628.1) | 100.0 |
|  | | | | | | | |
|  | | | | | | | |
| ------------------ | | | | | | | |
|  | | | | | | | |
|  | | | | | | | |
| **Input Sequence** | SM-Roxy-RH01221661_S26_L001_R1_001_(paired)_contig_20_30 # 35235 # 36497 # -1 # ID=20_30;partial=00;start_type=ATG;rbs_motif=AGGAG;rbs_spacer=5-10bp;gc_cont=0.474 | | | | | | |
|  | PROJECT ID | ACCESSION ID | ORGANISMS | CLASS | PROTEIN FUNCTION | PROTEIN ID | %IDENTITY |
| **Matched Family** | [19467](http://www.ncbi.nlm.nih.gov/bioproject?db=bioproject&cmd=ShowDetailView&TermToSearch=19467) | [CP001144](http://www.ncbi.nlm.nih.gov/nuccore/CP001144) | Salmonella enterica subsp. enterica serovar Dublin str. C | Gammaproteobacteria | 2021853, complete genome. | [ACH74256](http://www.ncbi.nlm.nih.gov/protein/ACH74256.1) | 100.0 |
|  | | | | | | | |
|  | | | | | | | |
| ------------------ | | | | | | | |
|  | | | | | | | |
|  | | | | | | | |
| **Input Sequence** | SM-Roxy-RH01221661_S26_L001_R1_001_(paired)_contig_24_50 # 49022 # 50281 # -1 # ID=24_50;partial=00;start_type=ATG;rbs_motif=GGA/GAG/AGG;rbs_spacer=5-10bp;gc_cont=0.571 | | | | | | |
|  | PROJECT ID | ACCESSION ID | ORGANISMS | CLASS | PROTEIN FUNCTION | PROTEIN ID | %IDENTITY |
| **Matched Family** | [18747](http://www.ncbi.nlm.nih.gov/bioproject?db=bioproject&cmd=ShowDetailView&TermToSearch=18747) | [CP001113](http://www.ncbi.nlm.nih.gov/nuccore/CP001113) | Salmonella enterica subsp. enterica serovar Newport str. SL254, complete genome. | Gammaproteobacteria | conserved hypothetical protein | [ACF64658](http://www.ncbi.nlm.nih.gov/protein/ACF64658.1) | 100.0 |
|  | | | | | | | |
|  | | | | | | | |
| ------------------ | | | | | | | |
|  | | | | | | | |
|  | | | | | | | |
| **Input Sequence** | SM-Roxy-RH01221661_S26_L001_R1_001_(paired)_contig_11_77 # 80270 # 81274 # -1 # ID=11_77;partial=00;start_type=ATG;rbs_motif=None;rbs_spacer=None;gc_cont=0.522 | | | | | | |
|  | PROJECT ID | ACCESSION ID | ORGANISMS | CLASS | PROTEIN FUNCTION | PROTEIN ID | %IDENTITY |
| **Matched Family** | [13086](http://www.ncbi.nlm.nih.gov/bioproject?db=bioproject&cmd=ShowDetailView&TermToSearch=13086) | [CP000026](http://www.ncbi.nlm.nih.gov/nuccore/CP000026) | Salmonella enterica subsp. enterica serovar Paratyphi A str. ATCC 9150, complete genome. | Gammaproteobacteria | nucleoside permease | [AAV78814](http://www.ncbi.nlm.nih.gov/protein/AAV78814.1) | 100.0 |
|  | | | | | | | |
|  | | | | | | | |
| ------------------ | | | | | | | |
|  | | | | | | | |
|  | | | | | | | |
| **Input Sequence** | SM-Roxy-RH01221661_S26_L001_R1_001_(paired)_contig_3_26 # 24300 # 25556 # 1 # ID=3_26;partial=00;start_type=ATG;rbs_motif=AGGA;rbs_spacer=5-10bp;gc_cont=0.491 | | | | | | |
|  | PROJECT ID | ACCESSION ID | ORGANISMS | CLASS | PROTEIN FUNCTION | PROTEIN ID | %IDENTITY |
| **Matched Family** | [18747](http://www.ncbi.nlm.nih.gov/bioproject?db=bioproject&cmd=ShowDetailView&TermToSearch=18747) | [CP001113](http://www.ncbi.nlm.nih.gov/nuccore/CP001113) | Salmonella enterica subsp. enterica serovar Newport str. SL254, complete genome. | Gammaproteobacteria | nucleoside permease NupG | [ACF61709](http://www.ncbi.nlm.nih.gov/protein/ACF61709.1) | 100.0 |
|  | | | | | | | |
|  | | | | | | | |
| ------------------ | | | | | | | |
|  | | | | | | | |
|  | | | | | | | |
| **Input Sequence** | SM-Roxy-RH01221661_S26_L001_R1_001_(paired)_contig_35_20 # 21749 # 22996 # 1 # ID=35_20;partial=00;start_type=ATG;rbs_motif=GGAG/GAGG;rbs_spacer=5-10bp;gc_cont=0.502 | | | | | | |
|  | PROJECT ID | ACCESSION ID | ORGANISMS | CLASS | PROTEIN FUNCTION | PROTEIN ID | %IDENTITY |
| **Matched Family** | [30687](http://www.ncbi.nlm.nih.gov/bioproject?db=bioproject&cmd=ShowDetailView&TermToSearch=30687) | [AM933172](http://www.ncbi.nlm.nih.gov/nuccore/AM933172) | Salmonella enterica subsp. enterica serovar Enteritidis str. P125109 complete genome. | Gammaproteobacteria | conserved hypothetical protein | [CAR31628](http://www.ncbi.nlm.nih.gov/protein/CAR31628.1) | 100.0 |
|  | | | | | | | |
|  | | | | | | | |
| ------------------ | | | | | | | |
|  | | | | | | | |
|  | | | | | | | |
| **Input Sequence** | SM-Roxy-RH01221661_S26_L001_R1_001_(paired)_contig_24_59 # 60971 # 62218 # -1 # ID=24_59;partial=00;start_type=ATG;rbs_motif=GGA/GAG/AGG;rbs_spacer=5-10bp;gc_cont=0.464 | | | | | | |
|  | PROJECT ID | ACCESSION ID | ORGANISMS | CLASS | PROTEIN FUNCTION | PROTEIN ID | %IDENTITY |
| **Matched Family** | [18747](http://www.ncbi.nlm.nih.gov/bioproject?db=bioproject&cmd=ShowDetailView&TermToSearch=18747) | [CP001113](http://www.ncbi.nlm.nih.gov/nuccore/CP001113) | Salmonella enterica subsp. enterica serovar Newport str. SL254, complete genome. | Gammaproteobacteria | major facilitator family transporter | [ACF62031](http://www.ncbi.nlm.nih.gov/protein/ACF62031.1) | 100.0 |
|  | | | | | | | |
|  | | | | | | | |
| ------------------ | | | | | | | |
|  | | | | | | | |
|  | | | | | | | |
| **Input Sequence** | SM-Roxy-RH01221661_S26_L001_R1_001_(paired)_contig_40_34 # 30382 # 31626 # -1 # ID=40_34;partial=00;start_type=ATG;rbs_motif=GGA/GAG/AGG;rbs_spacer=5-10bp;gc_cont=0.623 | | | | | | |
|  | PROJECT ID | ACCESSION ID | ORGANISMS | CLASS | PROTEIN FUNCTION | PROTEIN ID | %IDENTITY |
| **Matched Family** | [30687](http://www.ncbi.nlm.nih.gov/bioproject?db=bioproject&cmd=ShowDetailView&TermToSearch=30687) | [AM933172](http://www.ncbi.nlm.nih.gov/nuccore/AM933172) | Salmonella enterica subsp. enterica serovar Enteritidis str. P125109 complete genome. | Gammaproteobacteria | ferric enterochelin esterase | [CAR34201](http://www.ncbi.nlm.nih.gov/protein/CAR34201.1) | 100.0 |
|  | | | | | | | |
|  | | | | | | | |
| ------------------ | | | | | | | |
|  | | | | | | | |
|  | | | | | | | |
| **Input Sequence** | SM-Roxy-RH01221661_S26_L001_R1_001_(paired)_contig_42_32 # 30917 # 31333 # -1 # ID=42_32;partial=00;start_type=ATG;rbs_motif=GGAG/GAGG;rbs_spacer=5-10bp;gc_cont=0.580 | | | | | | |
|  | PROJECT ID | ACCESSION ID | ORGANISMS | CLASS | PROTEIN FUNCTION | PROTEIN ID | %IDENTITY |
| **Matched Family** | [13086](http://www.ncbi.nlm.nih.gov/bioproject?db=bioproject&cmd=ShowDetailView&TermToSearch=13086) | [CP000026](http://www.ncbi.nlm.nih.gov/nuccore/CP000026) | Salmonella enterica subsp. enterica serovar Paratyphi A str. ATCC 9150, complete genome. | Gammaproteobacteria | glucose-1-phosphatase precursor (G1Pase), secreted | [AAV77654](http://www.ncbi.nlm.nih.gov/protein/AAV77654.1) | 100.0 |
|  | | | | | | | |
|  | | | | | | | |
| ------------------ | | | | | | | |
|  | | | | | | | |
|  | | | | | | | |
| **Input Sequence** | SM-Roxy-RH01221661_S26_L001_R1_001_(paired)_contig_42_31 # 30092 # 30910 # -1 # ID=42_31;partial=00;start_type=ATG;rbs_motif=GGA/GAG/AGG;rbs_spacer=5-10bp;gc_cont=0.541 | | | | | | |
|  | PROJECT ID | ACCESSION ID | ORGANISMS | CLASS | PROTEIN FUNCTION | PROTEIN ID | %IDENTITY |
| **Matched Family** | [18747](http://www.ncbi.nlm.nih.gov/bioproject?db=bioproject&cmd=ShowDetailView&TermToSearch=18747) | [CP001113](http://www.ncbi.nlm.nih.gov/nuccore/CP001113) | Salmonella enterica subsp. enterica serovar Newport str. SL254, complete genome. | Gammaproteobacteria | glucose-1-phosphatase | [ACF63833](http://www.ncbi.nlm.nih.gov/protein/ACF63833.1) | 100.0 |
|  | | | | | | | |
|  | | | | | | | |
| ------------------ | | | | | | | |
|  | | | | | | | |
|  | | | | | | | |
| **Input Sequence** | SM-Roxy-RH01221661_S26_L001_R1_001_(paired)_contig_23_18 # 17064 # 18305 # -1 # ID=23_18;partial=00;start_type=ATG;rbs_motif=GGAG/GAGG;rbs_spacer=5-10bp;gc_cont=0.572 | | | | | | |
|  | PROJECT ID | ACCESSION ID | ORGANISMS | CLASS | PROTEIN FUNCTION | PROTEIN ID | %IDENTITY |
| **Matched Family** | [18747](http://www.ncbi.nlm.nih.gov/bioproject?db=bioproject&cmd=ShowDetailView&TermToSearch=18747) | [CP001113](http://www.ncbi.nlm.nih.gov/nuccore/CP001113) | Salmonella enterica subsp. enterica serovar Newport str. SL254, complete genome. | Gammaproteobacteria | multidrug resistance protein MdtM | [ACF61354](http://www.ncbi.nlm.nih.gov/protein/ACF61354.1) | 100.0 |
|  | | | | | | | |
|  | | | | | | | |
| ------------------ | | | | | | | |
|  | | | | | | | |
|  | | | | | | | |
| **Input Sequence** | SM-Roxy-RH01221661_S26_L001_R1_001_(paired)_contig_5_19 # 19590 # 20831 # -1 # ID=5_19;partial=00;start_type=ATG;rbs_motif=3Base/5BMM;rbs_spacer=13-15bp;gc_cont=0.482 | | | | | | |
|  | PROJECT ID | ACCESSION ID | ORGANISMS | CLASS | PROTEIN FUNCTION | PROTEIN ID | %IDENTITY |
| **Matched Family** | [30687](http://www.ncbi.nlm.nih.gov/bioproject?db=bioproject&cmd=ShowDetailView&TermToSearch=30687) | [AM933172](http://www.ncbi.nlm.nih.gov/nuccore/AM933172) | Salmonella enterica subsp. enterica serovar Enteritidis str. P125109 complete genome. | Gammaproteobacteria | putative coproporphyrinogen III oxidase | [CAR35374](http://www.ncbi.nlm.nih.gov/protein/CAR35374.1) | 100.0 |
|  | | | | | | | |
|  | | | | | | | |
| ------------------ | | | | | | | |
|  | | | | | | | |
|  | | | | | | | |
| **Input Sequence** | SM-Roxy-RH01221661_S26_L001_R1_001_(paired)_contig_25_146 # 137982 # 139223 # -1 # ID=25_146;partial=00;start_type=ATG;rbs_motif=GGA/GAG/AGG;rbs_spacer=5-10bp;gc_cont=0.494 | | | | | | |
|  | PROJECT ID | ACCESSION ID | ORGANISMS | CLASS | PROTEIN FUNCTION | PROTEIN ID | %IDENTITY |
| **Matched Family** | [241](http://www.ncbi.nlm.nih.gov/bioproject?db=bioproject&cmd=ShowDetailView&TermToSearch=241) | [AE006468](http://www.ncbi.nlm.nih.gov/nuccore/AE006468) | Salmonella enterica subsp. enterica serovar Typhimurium str. LT2, complete genome. | Gammaproteobacteria | putative multidrug efflux protein | [AAL20464](http://www.ncbi.nlm.nih.gov/protein/AAL20464.1) | 100.0 |
|  | | | | | | | |
|  | | | | | | | |
| ------------------ | | | | | | | |
|  | | | | | | | |
|  | | | | | | | |
| **Input Sequence** | SM-Roxy-RH01221661_S26_L001_R1_001_(paired)_contig_14_27 # 27999 # 28646 # -1 # ID=14_27;partial=00;start_type=ATG;rbs_motif=None;rbs_spacer=None;gc_cont=0.549 | | | | | | |
|  | PROJECT ID | ACCESSION ID | ORGANISMS | CLASS | PROTEIN FUNCTION | PROTEIN ID | %IDENTITY |
| **Matched Family** | [30689](http://www.ncbi.nlm.nih.gov/bioproject?db=bioproject&cmd=ShowDetailView&TermToSearch=30689) | [AM933173](http://www.ncbi.nlm.nih.gov/nuccore/AM933173) | Salmonella enterica subsp. enterica serovar Gallinarum str. 287/91 complete genome. | Gammaproteobacteria | hypothetical protein | [CAR36560](http://www.ncbi.nlm.nih.gov/protein/CAR36560.1) | 100.0 |
|  | | | | | | | |
|  | | | | | | | |
| ------------------ | | | | | | | |
|  | | | | | | | |
|  | | | | | | | |
| **Input Sequence** | SM-Roxy-RH01221661_S26_L001_R1_001_(paired)_contig_3_38 # 38735 # 39970 # -1 # ID=3_38;partial=00;start_type=ATG;rbs_motif=AGGA;rbs_spacer=5-10bp;gc_cont=0.591 | | | | | | |
|  | PROJECT ID | ACCESSION ID | ORGANISMS | CLASS | PROTEIN FUNCTION | PROTEIN ID | %IDENTITY |
| **Matched Family** | [18747](http://www.ncbi.nlm.nih.gov/bioproject?db=bioproject&cmd=ShowDetailView&TermToSearch=18747) | [CP001113](http://www.ncbi.nlm.nih.gov/nuccore/CP001113) | Salmonella enterica subsp. enterica serovar Newport str. SL254, complete genome. | Gammaproteobacteria | Cl- channel, voltage-gated family protein | [ACF61790](http://www.ncbi.nlm.nih.gov/protein/ACF61790.1) | 100.0 |
|  | | | | | | | |
|  | | | | | | | |
| ------------------ | | | | | | | |
|  | | | | | | | |
|  | | | | | | | |
| **Input Sequence** | SM-Roxy-RH01221661_S26_L001_R1_001_(paired)_contig_24_53 # 53342 # 54577 # 1 # ID=24_53;partial=00;start_type=ATG;rbs_motif=AGGA;rbs_spacer=5-10bp;gc_cont=0.536 | | | | | | |
|  | PROJECT ID | ACCESSION ID | ORGANISMS | CLASS | PROTEIN FUNCTION | PROTEIN ID | %IDENTITY |
| **Matched Family** | [30687](http://www.ncbi.nlm.nih.gov/bioproject?db=bioproject&cmd=ShowDetailView&TermToSearch=30687) | [AM933172](http://www.ncbi.nlm.nih.gov/nuccore/AM933172) | Salmonella enterica subsp. enterica serovar Enteritidis str. P125109 complete genome. | Gammaproteobacteria | allantoate amidohydrolase | [CAR32093](http://www.ncbi.nlm.nih.gov/protein/CAR32093.1) | 100.0 |
|  | | | | | | | |
|  | | | | | | | |
| ------------------ | | | | | | | |
|  | | | | | | | |
|  | | | | | | | |
| **Input Sequence** | SM-Roxy-RH01221661_S26_L001_R1_001_(paired)_contig_9_72 # 81535 # 82764 # -1 # ID=9_72;partial=00;start_type=ATG;rbs_motif=None;rbs_spacer=None;gc_cont=0.502 | | | | | | |
|  | PROJECT ID | ACCESSION ID | ORGANISMS | CLASS | PROTEIN FUNCTION | PROTEIN ID | %IDENTITY |
| **Matched Family** | [30687](http://www.ncbi.nlm.nih.gov/bioproject?db=bioproject&cmd=ShowDetailView&TermToSearch=30687) | [AM933172](http://www.ncbi.nlm.nih.gov/nuccore/AM933172) | Salmonella enterica subsp. enterica serovar Enteritidis str. P125109 complete genome. | Gammaproteobacteria | conserved hypothetical protein | [CAR31772](http://www.ncbi.nlm.nih.gov/protein/CAR31772.1) | 100.0 |
|  | | | | | | | |
|  | | | | | | | |
| ------------------ | | | | | | | |
|  | | | | | | | |
|  | | | | | | | |
| **Input Sequence** | SM-Roxy-RH01221661_S26_L001_R1_001_(paired)_contig_11_310 # 327306 # 328535 # 1 # ID=11_310;partial=00;start_type=ATG;rbs_motif=GGAG/GAGG;rbs_spacer=5-10bp;gc_cont=0.474 | | | | | | |
|  | PROJECT ID | ACCESSION ID | ORGANISMS | CLASS | PROTEIN FUNCTION | PROTEIN ID | %IDENTITY |
| **Matched Family** | [241](http://www.ncbi.nlm.nih.gov/bioproject?db=bioproject&cmd=ShowDetailView&TermToSearch=241) | [AE006468](http://www.ncbi.nlm.nih.gov/nuccore/AE006468) | Salmonella enterica subsp. enterica serovar Typhimurium str. LT2, complete genome. | Gammaproteobacteria | cell invasion protein | [AAL21764](http://www.ncbi.nlm.nih.gov/protein/AAL21764.1) | 100.0 |
|  | | | | | | | |
|  | | | | | | | |
| ------------------ | | | | | | | |
|  | | | | | | | |
|  | | | | | | | |
| **Input Sequence** | SM-Roxy-RH01221661_S26_L001_R1_001_(paired)_contig_11_170 # 168560 # 169789 # -1 # ID=11_170;partial=00;start_type=ATG;rbs_motif=None;rbs_spacer=None;gc_cont=0.472 | | | | | | |
|  | PROJECT ID | ACCESSION ID | ORGANISMS | CLASS | PROTEIN FUNCTION | PROTEIN ID | %IDENTITY |
| **Matched Family** | [30687](http://www.ncbi.nlm.nih.gov/bioproject?db=bioproject&cmd=ShowDetailView&TermToSearch=30687) | [AM933172](http://www.ncbi.nlm.nih.gov/nuccore/AM933172) | Salmonella enterica subsp. enterica serovar Enteritidis str. P125109 complete genome. | Gammaproteobacteria | probable amino acid transport protein | [CAR34443](http://www.ncbi.nlm.nih.gov/protein/CAR34443.1) | 100.0 |
|  | | | | | | | |
|  | | | | | | | |
| ------------------ | | | | | | | |
|  | | | | | | | |
|  | | | | | | | |
| **Input Sequence** | SM-Roxy-RH01221661_S26_L001_R1_001_(paired)_contig_25_232 # 238075 # 239301 # 1 # ID=25_232;partial=00;start_type=ATG;rbs_motif=AGGAG;rbs_spacer=5-10bp;gc_cont=0.392 | | | | | | |
|  | PROJECT ID | ACCESSION ID | ORGANISMS | CLASS | PROTEIN FUNCTION | PROTEIN ID | %IDENTITY |
| **Matched Family** | [19467](http://www.ncbi.nlm.nih.gov/bioproject?db=bioproject&cmd=ShowDetailView&TermToSearch=19467) | [CP001144](http://www.ncbi.nlm.nih.gov/nuccore/CP001144) | Salmonella enterica subsp. enterica serovar Dublin str. C | Gammaproteobacteria | 2021853, complete genome. | [ACH78013](http://www.ncbi.nlm.nih.gov/protein/ACH78013.1) | 100.0 |
|  | | | | | | | |
|  | | | | | | | |
| ------------------ | | | | | | | |
|  | | | | | | | |
|  | | | | | | | |
| **Input Sequence** | SM-Roxy-RH01221661_S26_L001_R1_001_(paired)_contig_2_27 # 27030 # 28256 # -1 # ID=2_27;partial=00;start_type=ATG;rbs_motif=GGAG/GAGG;rbs_spacer=5-10bp;gc_cont=0.602 | | | | | | |
|  | PROJECT ID | ACCESSION ID | ORGANISMS | CLASS | PROTEIN FUNCTION | PROTEIN ID | %IDENTITY |
| **Matched Family** | [30687](http://www.ncbi.nlm.nih.gov/bioproject?db=bioproject&cmd=ShowDetailView&TermToSearch=30687) | [AM933172](http://www.ncbi.nlm.nih.gov/nuccore/AM933172) | Salmonella enterica subsp. enterica serovar Enteritidis str. P125109 complete genome. | Gammaproteobacteria | putative cation efflux pump | [CAR31921](http://www.ncbi.nlm.nih.gov/protein/CAR31921.1) | 100.0 |
|  | | | | | | | |
|  | | | | | | | |
| ------------------ | | | | | | | |
|  | | | | | | | |
|  | | | | | | | |
| **Input Sequence** | SM-Roxy-RH01221661_S26_L001_R1_001_(paired)_contig_42_98 # 98442 # 99071 # -1 # ID=42_98;partial=00;start_type=ATG;rbs_motif=None;rbs_spacer=None;gc_cont=0.440 | | | | | | |
|  | PROJECT ID | ACCESSION ID | ORGANISMS | CLASS | PROTEIN FUNCTION | PROTEIN ID | %IDENTITY |
| **Matched Family** | [19467](http://www.ncbi.nlm.nih.gov/bioproject?db=bioproject&cmd=ShowDetailView&TermToSearch=19467) | [CP001144](http://www.ncbi.nlm.nih.gov/nuccore/CP001144) | Salmonella enterica subsp. enterica serovar Dublin str. C | Gammaproteobacteria | 2021853, complete genome. | [ACH75346](http://www.ncbi.nlm.nih.gov/protein/ACH75346.1) | 100.0 |
|  | | | | | | | |
|  | | | | | | | |
| ------------------ | | | | | | | |
|  | | | | | | | |
|  | | | | | | | |
| **Input Sequence** | SM-Roxy-RH01221661_S26_L001_R1_001_(paired)_contig_21_190 # 180238 # 181461 # -1 # ID=21_190;partial=00;start_type=ATG;rbs_motif=GGxGG;rbs_spacer=5-10bp;gc_cont=0.577 | | | | | | |
|  | PROJECT ID | ACCESSION ID | ORGANISMS | CLASS | PROTEIN FUNCTION | PROTEIN ID | %IDENTITY |
| **Matched Family** | [19467](http://www.ncbi.nlm.nih.gov/bioproject?db=bioproject&cmd=ShowDetailView&TermToSearch=19467) | [CP001144](http://www.ncbi.nlm.nih.gov/nuccore/CP001144) | Salmonella enterica subsp. enterica serovar Dublin str. C | Gammaproteobacteria | 2021853, complete genome. | [ACH75131](http://www.ncbi.nlm.nih.gov/protein/ACH75131.1) | 100.0 |
|  | | | | | | | |
|  | | | | | | | |
| ------------------ | | | | | | | |
|  | | | | | | | |
|  | | | | | | | |
| **Input Sequence** | SM-Roxy-RH01221661_S26_L001_R1_001_(paired)_contig_12_201 # 194801 # 196024 # -1 # ID=12_201;partial=00;start_type=ATG;rbs_motif=GGA/GAG/AGG;rbs_spacer=5-10bp;gc_cont=0.339 | | | | | | |
|  | PROJECT ID | ACCESSION ID | ORGANISMS | CLASS | PROTEIN FUNCTION | PROTEIN ID | %IDENTITY |
| **Matched Family** | [19467](http://www.ncbi.nlm.nih.gov/bioproject?db=bioproject&cmd=ShowDetailView&TermToSearch=19467) | [CP001144](http://www.ncbi.nlm.nih.gov/nuccore/CP001144) | Salmonella enterica subsp. enterica serovar Dublin str. C | Gammaproteobacteria | 2021853, complete genome. | [ACH74809](http://www.ncbi.nlm.nih.gov/protein/ACH74809.1) | 100.0 |
|  | | | | | | | |
|  | | | | | | | |
| ------------------ | | | | | | | |
|  | | | | | | | |
|  | | | | | | | |
| **Input Sequence** | SM-Roxy-RH01221661_S26_L001_R1_001_(paired)_contig_65_15 # 22115 # 23332 # -1 # ID=65_15;partial=00;start_type=GTG;rbs_motif=GGA/GAG/AGG;rbs_spacer=5-10bp;gc_cont=0.500 | | | | | | |
|  | PROJECT ID | ACCESSION ID | ORGANISMS | CLASS | PROTEIN FUNCTION | PROTEIN ID | %IDENTITY |
| **Matched Family** | [19467](http://www.ncbi.nlm.nih.gov/bioproject?db=bioproject&cmd=ShowDetailView&TermToSearch=19467) | [CP001144](http://www.ncbi.nlm.nih.gov/nuccore/CP001144) | Salmonella enterica subsp. enterica serovar Dublin str. C | Gammaproteobacteria | 2021853, complete genome. | [ACH77052](http://www.ncbi.nlm.nih.gov/protein/ACH77052.1) | 100.0 |
|  | | | | | | | |
|  | | | | | | | |
| ------------------ | | | | | | | |
|  | | | | | | | |
|  | | | | | | | |
| **Input Sequence** | SM-Roxy-RH01221661_S26_L001_R1_001_(paired)_contig_68_9 # 8109 # 9326 # 1 # ID=68_9;partial=00;start_type=ATG;rbs_motif=GGAG/GAGG;rbs_spacer=5-10bp;gc_cont=0.615 | | | | | | |
|  | PROJECT ID | ACCESSION ID | ORGANISMS | CLASS | PROTEIN FUNCTION | PROTEIN ID | %IDENTITY |
| **Matched Family** | [30687](http://www.ncbi.nlm.nih.gov/bioproject?db=bioproject&cmd=ShowDetailView&TermToSearch=30687) | [AM933172](http://www.ncbi.nlm.nih.gov/nuccore/AM933172) | Salmonella enterica subsp. enterica serovar Enteritidis str. P125109 complete genome. | Gammaproteobacteria | flagellar hook-length control protein | [CAR32619](http://www.ncbi.nlm.nih.gov/protein/CAR32619.1) | 100.0 |
|  | | | | | | | |
|  | | | | | | | |
| ------------------ | | | | | | | |
|  | | | | | | | |
|  | | | | | | | |
| **Input Sequence** | SM-Roxy-RH01221661_S26_L001_R1_001_(paired)_contig_21_197 # 186565 # 187782 # -1 # ID=21_197;partial=00;start_type=ATG;rbs_motif=GGA/GAG/AGG;rbs_spacer=5-10bp;gc_cont=0.561 | | | | | | |
|  | PROJECT ID | ACCESSION ID | ORGANISMS | CLASS | PROTEIN FUNCTION | PROTEIN ID | %IDENTITY |
| **Matched Family** | [19467](http://www.ncbi.nlm.nih.gov/bioproject?db=bioproject&cmd=ShowDetailView&TermToSearch=19467) | [CP001144](http://www.ncbi.nlm.nih.gov/nuccore/CP001144) | Salmonella enterica subsp. enterica serovar Dublin str. C | Gammaproteobacteria | 2021853, complete genome. | [ACH75534](http://www.ncbi.nlm.nih.gov/protein/ACH75534.1) | 100.0 |
|  | | | | | | | |
|  | | | | | | | |
| ------------------ | | | | | | | |
|  | | | | | | | |
|  | | | | | | | |
| **Input Sequence** | SM-Roxy-RH01221661_S26_L001_R1_001_(paired)_contig_21_196 # 185376 # 186590 # -1 # ID=21_196;partial=00;start_type=ATG;rbs_motif=GGA/GAG/AGG;rbs_spacer=5-10bp;gc_cont=0.430 | | | | | | |
|  | PROJECT ID | ACCESSION ID | ORGANISMS | CLASS | PROTEIN FUNCTION | PROTEIN ID | %IDENTITY |
| **Matched Family** | [13086](http://www.ncbi.nlm.nih.gov/bioproject?db=bioproject&cmd=ShowDetailView&TermToSearch=13086) | [CP000026](http://www.ncbi.nlm.nih.gov/nuccore/CP000026) | Salmonella enterica subsp. enterica serovar Paratyphi A str. ATCC 9150, complete genome. | Gammaproteobacteria | putative colanic acid polymerase | [AAV76750](http://www.ncbi.nlm.nih.gov/protein/AAV76750.1) | 100.0 |
|  | | | | | | | |
|  | | | | | | | |
| ------------------ | | | | | | | |
|  | | | | | | | |
|  | | | | | | | |
| **Input Sequence** | SM-Roxy-RH01221661_S26_L001_R1_001_(paired)_contig_47_22 # 21207 # 22421 # 1 # ID=47_22;partial=00;start_type=ATG;rbs_motif=GGAG/GAGG;rbs_spacer=5-10bp;gc_cont=0.390 | | | | | | |
|  | PROJECT ID | ACCESSION ID | ORGANISMS | CLASS | PROTEIN FUNCTION | PROTEIN ID | %IDENTITY |
| **Matched Family** | [19467](http://www.ncbi.nlm.nih.gov/bioproject?db=bioproject&cmd=ShowDetailView&TermToSearch=19467) | [CP001144](http://www.ncbi.nlm.nih.gov/nuccore/CP001144) | Salmonella enterica subsp. enterica serovar Dublin str. C | Gammaproteobacteria | 2021853, complete genome. | [ACH77456](http://www.ncbi.nlm.nih.gov/protein/ACH77456.1) | 100.0 |
|  | | | | | | | |
|  | | | | | | | |
| ------------------ | | | | | | | |
|  | | | | | | | |
|  | | | | | | | |
| **Input Sequence** | SM-Roxy-RH01221661_S26_L001_R1_001_(paired)_contig_12_263 # 266771 # 267982 # 1 # ID=12_263;partial=00;start_type=ATG;rbs_motif=AGxAG;rbs_spacer=5-10bp;gc_cont=0.432 | | | | | | |
|  | PROJECT ID | ACCESSION ID | ORGANISMS | CLASS | PROTEIN FUNCTION | PROTEIN ID | %IDENTITY |
| **Matched Family** | [30687](http://www.ncbi.nlm.nih.gov/bioproject?db=bioproject&cmd=ShowDetailView&TermToSearch=30687) | [AM933172](http://www.ncbi.nlm.nih.gov/nuccore/AM933172) | Salmonella enterica subsp. enterica serovar Enteritidis str. P125109 complete genome. | Gammaproteobacteria | putative pathogenicity island protein | [CAR33232](http://www.ncbi.nlm.nih.gov/protein/CAR33232.1) | 100.0 |
|  | | | | | | | |
|  | | | | | | | |
| ------------------ | | | | | | | |
|  | | | | | | | |
|  | | | | | | | |
| **Input Sequence** | SM-Roxy-RH01221661_S26_L001_R1_001_(paired)_contig_11_285 # 301753 # 302961 # 1 # ID=11_285;partial=00;start_type=ATG;rbs_motif=GGAGG;rbs_spacer=5-10bp;gc_cont=0.543 | | | | | | |
|  | PROJECT ID | ACCESSION ID | ORGANISMS | CLASS | PROTEIN FUNCTION | PROTEIN ID | %IDENTITY |
| **Matched Family** | [30687](http://www.ncbi.nlm.nih.gov/bioproject?db=bioproject&cmd=ShowDetailView&TermToSearch=30687) | [AM933172](http://www.ncbi.nlm.nih.gov/nuccore/AM933172) | Salmonella enterica subsp. enterica serovar Enteritidis str. P125109 complete genome. | Gammaproteobacteria | possible membrane transport protein | [CAR34329](http://www.ncbi.nlm.nih.gov/protein/CAR34329.1) | 100.0 |
|  | | | | | | | |
|  | | | | | | | |
| ------------------ | | | | | | | |
|  | | | | | | | |
|  | | | | | | | |
| **Input Sequence** | SM-Roxy-RH01221661_S26_L001_R1_001_(paired)_contig_13_64 # 64498 # 65706 # -1 # ID=13_64;partial=00;start_type=ATG;rbs_motif=GGAG/GAGG;rbs_spacer=5-10bp;gc_cont=0.468 | | | | | | |
|  | PROJECT ID | ACCESSION ID | ORGANISMS | CLASS | PROTEIN FUNCTION | PROTEIN ID | %IDENTITY |
| **Matched Family** | [30687](http://www.ncbi.nlm.nih.gov/bioproject?db=bioproject&cmd=ShowDetailView&TermToSearch=30687) | [AM933172](http://www.ncbi.nlm.nih.gov/nuccore/AM933172) | Salmonella enterica subsp. enterica serovar Enteritidis str. P125109 complete genome. | Gammaproteobacteria | putative sugar transporter | [CAR35740](http://www.ncbi.nlm.nih.gov/protein/CAR35740.1) | 100.0 |
|  | | | | | | | |
|  | | | | | | | |
| ------------------ | | | | | | | |
|  | | | | | | | |
|  | | | | | | | |
| **Input Sequence** | SM-Roxy-RH01221661_S26_L001_R1_001_(paired)_contig_16_34 # 27681 # 28886 # 1 # ID=16_34;partial=00;start_type=ATG;rbs_motif=GGAG/GAGG;rbs_spacer=5-10bp;gc_cont=0.470 | | | | | | |
|  | PROJECT ID | ACCESSION ID | ORGANISMS | CLASS | PROTEIN FUNCTION | PROTEIN ID | %IDENTITY |
| **Matched Family** | [20993](http://www.ncbi.nlm.nih.gov/bioproject?db=bioproject&cmd=ShowDetailView&TermToSearch=20993) | [CP000858](http://www.ncbi.nlm.nih.gov/nuccore/CP000858) | Salmonella enterica subsp. enterica serovar Paratyphi C strain RKS4594 plasmid pSPCV, complete sequence. | Gammaproteobacteria | ParA | [ACN48823](http://www.ncbi.nlm.nih.gov/protein/ACN48823.1) | 100.0 |
|  | | | | | | | |
|  | | | | | | | |
| ------------------ | | | | | | | |
|  | | | | | | | |
|  | | | | | | | |
| **Input Sequence** | SM-Roxy-RH01221661_S26_L001_R1_001_(paired)_contig_22_219 # 213992 # 215197 # -1 # ID=22_219;partial=00;start_type=ATG;rbs_motif=GGAG/GAGG;rbs_spacer=5-10bp;gc_cont=0.469 | | | | | | |
|  | PROJECT ID | ACCESSION ID | ORGANISMS | CLASS | PROTEIN FUNCTION | PROTEIN ID | %IDENTITY |
| **Matched Family** | [19467](http://www.ncbi.nlm.nih.gov/bioproject?db=bioproject&cmd=ShowDetailView&TermToSearch=19467) | [CP001144](http://www.ncbi.nlm.nih.gov/nuccore/CP001144) | Salmonella enterica subsp. enterica serovar Dublin str. C | Gammaproteobacteria | 2021853, complete genome. | [ACH73937](http://www.ncbi.nlm.nih.gov/protein/ACH73937.1) | 100.0 |
|  | | | | | | | |
|  | | | | | | | |
| ------------------ | | | | | | | |
|  | | | | | | | |
|  | | | | | | | |
| **Input Sequence** | SM-Roxy-RH01221661_S26_L001_R1_001_(paired)_contig_21_217 # 215827 # 217029 # 1 # ID=21_217;partial=00;start_type=ATG;rbs_motif=GGA/GAG/AGG;rbs_spacer=5-10bp;gc_cont=0.492 | | | | | | |
|  | PROJECT ID | ACCESSION ID | ORGANISMS | CLASS | PROTEIN FUNCTION | PROTEIN ID | %IDENTITY |
| **Matched Family** | [30687](http://www.ncbi.nlm.nih.gov/bioproject?db=bioproject&cmd=ShowDetailView&TermToSearch=30687) | [AM933172](http://www.ncbi.nlm.nih.gov/nuccore/AM933172) | Salmonella enterica subsp. enterica serovar Enteritidis str. P125109 complete genome. | Gammaproteobacteria | Conserved hypothetical protein | [CAR33711](http://www.ncbi.nlm.nih.gov/protein/CAR33711.1) | 100.0 |
|  | | | | | | | |
|  | | | | | | | |
| ------------------ | | | | | | | |
|  | | | | | | | |
|  | | | | | | | |
| **Input Sequence** | SM-Roxy-RH01221661_S26_L001_R1_001_(paired)_contig_54_29 # 27315 # 28511 # 1 # ID=54_29;partial=00;start_type=ATG;rbs_motif=GGA/GAG/AGG;rbs_spacer=5-10bp;gc_cont=0.462 | | | | | | |
|  | PROJECT ID | ACCESSION ID | ORGANISMS | CLASS | PROTEIN FUNCTION | PROTEIN ID | %IDENTITY |
| **Matched Family** | [18747](http://www.ncbi.nlm.nih.gov/bioproject?db=bioproject&cmd=ShowDetailView&TermToSearch=18747) | [CP001113](http://www.ncbi.nlm.nih.gov/nuccore/CP001113) | Salmonella enterica subsp. enterica serovar Newport str. SL254, complete genome. | Gammaproteobacteria | major facilitator family transporter | [ACF64882](http://www.ncbi.nlm.nih.gov/protein/ACF64882.1) | 100.0 |
|  | | | | | | | |
|  | | | | | | | |
| ------------------ | | | | | | | |
|  | | | | | | | |
|  | | | | | | | |
| **Input Sequence** | SM-Roxy-RH01221661_S26_L001_R1_001_(paired)_contig_21_258 # 260992 # 262185 # -1 # ID=21_258;partial=00;start_type=ATG;rbs_motif=AGGAG;rbs_spacer=5-10bp;gc_cont=0.589 | | | | | | |
|  | PROJECT ID | ACCESSION ID | ORGANISMS | CLASS | PROTEIN FUNCTION | PROTEIN ID | %IDENTITY |
| **Matched Family** | [18747](http://www.ncbi.nlm.nih.gov/bioproject?db=bioproject&cmd=ShowDetailView&TermToSearch=18747) | [CP001113](http://www.ncbi.nlm.nih.gov/nuccore/CP001113) | Salmonella enterica subsp. enterica serovar Newport str. SL254, complete genome. | Gammaproteobacteria | 3-hydroxybenzoate-6-hydroxylase | [ACF62669](http://www.ncbi.nlm.nih.gov/protein/ACF62669.1) | 100.0 |
|  | | | | | | | |
|  | | | | | | | |
| ------------------ | | | | | | | |
|  | | | | | | | |
|  | | | | | | | |
| **Input Sequence** | SM-Roxy-RH01221661_S26_L001_R1_001_(paired)_contig_35_19 # 20534 # 21724 # 1 # ID=35_19;partial=00;start_type=ATG;rbs_motif=GGA/GAG/AGG;rbs_spacer=5-10bp;gc_cont=0.495 | | | | | | |
|  | PROJECT ID | ACCESSION ID | ORGANISMS | CLASS | PROTEIN FUNCTION | PROTEIN ID | %IDENTITY |
| **Matched Family** | [19467](http://www.ncbi.nlm.nih.gov/bioproject?db=bioproject&cmd=ShowDetailView&TermToSearch=19467) | [CP001144](http://www.ncbi.nlm.nih.gov/nuccore/CP001144) | Salmonella enterica subsp. enterica serovar Dublin str. C | Gammaproteobacteria | 2021853, complete genome. | [ACH75064](http://www.ncbi.nlm.nih.gov/protein/ACH75064.1) | 100.0 |
|  | | | | | | | |
|  | | | | | | | |
| ------------------ | | | | | | | |
|  | | | | | | | |
|  | | | | | | | |
| **Input Sequence** | SM-Roxy-RH01221661_S26_L001_R1_001_(paired)_contig_36_40 # 44191 # 45378 # 1 # ID=36_40;partial=00;start_type=ATG;rbs_motif=GGAGG;rbs_spacer=5-10bp;gc_cont=0.632 | | | | | | |
|  | PROJECT ID | ACCESSION ID | ORGANISMS | CLASS | PROTEIN FUNCTION | PROTEIN ID | %IDENTITY |
| **Matched Family** | [30687](http://www.ncbi.nlm.nih.gov/bioproject?db=bioproject&cmd=ShowDetailView&TermToSearch=30687) | [AM933172](http://www.ncbi.nlm.nih.gov/nuccore/AM933172) | Salmonella enterica subsp. enterica serovar Enteritidis str. P125109 complete genome. | Gammaproteobacteria | putative alchohol dehydrogenase | [CAR34026](http://www.ncbi.nlm.nih.gov/protein/CAR34026.1) | 100.0 |
|  | | | | | | | |
|  | | | | | | | |
| ------------------ | | | | | | | |
|  | | | | | | | |
|  | | | | | | | |
| **Input Sequence** | SM-Roxy-RH01221661_S26_L001_R1_001_(paired)_contig_28_28 # 25364 # 26551 # -1 # ID=28_28;partial=00;start_type=ATG;rbs_motif=GGA/GAG/AGG;rbs_spacer=5-10bp;gc_cont=0.443 | | | | | | |
|  | PROJECT ID | ACCESSION ID | ORGANISMS | CLASS | PROTEIN FUNCTION | PROTEIN ID | %IDENTITY |
| **Matched Family** | [241](http://www.ncbi.nlm.nih.gov/bioproject?db=bioproject&cmd=ShowDetailView&TermToSearch=241) | [AE006468](http://www.ncbi.nlm.nih.gov/nuccore/AE006468) | Salmonella enterica subsp. enterica serovar Typhimurium str. LT2, complete genome. | Gammaproteobacteria | putative inner membrane protein | [AAL23293](http://www.ncbi.nlm.nih.gov/protein/AAL23293.1) | 100.0 |
|  | | | | | | | |
|  | | | | | | | |
| ------------------ | | | | | | | |
|  | | | | | | | |
|  | | | | | | | |
| **Input Sequence** | SM-Roxy-RH01221661_S26_L001_R1_001_(paired)_contig_25_118 # 110990 # 112177 # -1 # ID=25_118;partial=00;start_type=ATG;rbs_motif=GGA/GAG/AGG;rbs_spacer=5-10bp;gc_cont=0.519 | | | | | | |
|  | PROJECT ID | ACCESSION ID | ORGANISMS | CLASS | PROTEIN FUNCTION | PROTEIN ID | %IDENTITY |
| **Matched Family** | [30687](http://www.ncbi.nlm.nih.gov/bioproject?db=bioproject&cmd=ShowDetailView&TermToSearch=30687) | [AM933172](http://www.ncbi.nlm.nih.gov/nuccore/AM933172) | Salmonella enterica subsp. enterica serovar Enteritidis str. P125109 complete genome. | Gammaproteobacteria | putative membrane protein | [CAR33114](http://www.ncbi.nlm.nih.gov/protein/CAR33114.1) | 100.0 |
|  | | | | | | | |
|  | | | | | | | |
| ------------------ | | | | | | | |
|  | | | | | | | |
|  | | | | | | | |
| **Input Sequence** | SM-Roxy-RH01221661_S26_L001_R1_001_(paired)_contig_20_20 # 20906 # 22093 # -1 # ID=20_20;partial=00;start_type=ATG;rbs_motif=GGAG/GAGG;rbs_spacer=5-10bp;gc_cont=0.551 | | | | | | |
|  | PROJECT ID | ACCESSION ID | ORGANISMS | CLASS | PROTEIN FUNCTION | PROTEIN ID | %IDENTITY |
| **Matched Family** | [19467](http://www.ncbi.nlm.nih.gov/bioproject?db=bioproject&cmd=ShowDetailView&TermToSearch=19467) | [CP001144](http://www.ncbi.nlm.nih.gov/nuccore/CP001144) | Salmonella enterica subsp. enterica serovar Dublin str. C | Gammaproteobacteria | 2021853, complete genome. | [ACH76694](http://www.ncbi.nlm.nih.gov/protein/ACH76694.1) | 100.0 |
|  | | | | | | | |
|  | | | | | | | |
| ------------------ | | | | | | | |
|  | | | | | | | |
|  | | | | | | | |
| **Input Sequence** | SM-Roxy-RH01221661_S26_L001_R1_001_(paired)_contig_12_139 # 130275 # 131459 # -1 # ID=12_139;partial=00;start_type=ATG;rbs_motif=GGA/GAG/AGG;rbs_spacer=5-10bp;gc_cont=0.585 | | | | | | |
|  | PROJECT ID | ACCESSION ID | ORGANISMS | CLASS | PROTEIN FUNCTION | PROTEIN ID | %IDENTITY |
| **Matched Family** | [30687](http://www.ncbi.nlm.nih.gov/bioproject?db=bioproject&cmd=ShowDetailView&TermToSearch=30687) | [AM933172](http://www.ncbi.nlm.nih.gov/nuccore/AM933172) | Salmonella enterica subsp. enterica serovar Enteritidis str. P125109 complete genome. | Gammaproteobacteria | putative membrane protein | [CAR33354](http://www.ncbi.nlm.nih.gov/protein/CAR33354.1) | 100.0 |
|  | | | | | | | |
|  | | | | | | | |
| ------------------ | | | | | | | |
|  | | | | | | | |
|  | | | | | | | |
| **Input Sequence** | SM-Roxy-RH01221661_S26_L001_R1_001_(paired)_contig_11_68 # 69403 # 70587 # 1 # ID=11_68;partial=00;start_type=ATG;rbs_motif=AGGA;rbs_spacer=5-10bp;gc_cont=0.500 | | | | | | |
|  | PROJECT ID | ACCESSION ID | ORGANISMS | CLASS | PROTEIN FUNCTION | PROTEIN ID | %IDENTITY |
| **Matched Family** | [30687](http://www.ncbi.nlm.nih.gov/bioproject?db=bioproject&cmd=ShowDetailView&TermToSearch=30687) | [AM933172](http://www.ncbi.nlm.nih.gov/nuccore/AM933172) | Salmonella enterica subsp. enterica serovar Enteritidis str. P125109 complete genome. | Gammaproteobacteria | arylsulfatase regulator | [CAR34542](http://www.ncbi.nlm.nih.gov/protein/CAR34542.1) | 100.0 |
|  | | | | | | | |
|  | | | | | | | |
| ------------------ | | | | | | | |
|  | | | | | | | |
|  | | | | | | | |
| **Input Sequence** | SM-Roxy-RH01221661_S26_L001_R1_001_(paired)_contig_29_18 # 15872 # 17047 # 1 # ID=29_18;partial=00;start_type=ATG;rbs_motif=GGAG/GAGG;rbs_spacer=5-10bp;gc_cont=0.608 | | | | | | |
|  | PROJECT ID | ACCESSION ID | ORGANISMS | CLASS | PROTEIN FUNCTION | PROTEIN ID | %IDENTITY |
| **Matched Family** | [30687](http://www.ncbi.nlm.nih.gov/bioproject?db=bioproject&cmd=ShowDetailView&TermToSearch=30687) | [AM933172](http://www.ncbi.nlm.nih.gov/nuccore/AM933172) | Salmonella enterica subsp. enterica serovar Enteritidis str. P125109 complete genome. | Gammaproteobacteria | putative transmembrane transporter | [CAR33938](http://www.ncbi.nlm.nih.gov/protein/CAR33938.1) | 100.0 |
|  | | | | | | | |
|  | | | | | | | |
| ------------------ | | | | | | | |
|  | | | | | | | |
|  | | | | | | | |
| **Input Sequence** | SM-Roxy-RH01221661_S26_L001_R1_001_(paired)_contig_24_16 # 19460 # 20635 # 1 # ID=24_16;partial=00;start_type=ATG;rbs_motif=GGA/GAG/AGG;rbs_spacer=5-10bp;gc_cont=0.537 | | | | | | |
|  | PROJECT ID | ACCESSION ID | ORGANISMS | CLASS | PROTEIN FUNCTION | PROTEIN ID | %IDENTITY |
| **Matched Family** | [19467](http://www.ncbi.nlm.nih.gov/bioproject?db=bioproject&cmd=ShowDetailView&TermToSearch=19467) | [CP001144](http://www.ncbi.nlm.nih.gov/nuccore/CP001144) | Salmonella enterica subsp. enterica serovar Dublin str. C | Gammaproteobacteria | 2021853, complete genome. | [ACH74864](http://www.ncbi.nlm.nih.gov/protein/ACH74864.1) | 100.0 |
|  | | | | | | | |
|  | | | | | | | |
| ------------------ | | | | | | | |
|  | | | | | | | |
|  | | | | | | | |
| **Input Sequence** | SM-Roxy-RH01221661_S26_L001_R1_001_(paired)_contig_2_69 # 74943 # 76115 # -1 # ID=2_69;partial=00;start_type=ATG;rbs_motif=GGA/GAG/AGG;rbs_spacer=3-4bp;gc_cont=0.559 | | | | | | |
|  | PROJECT ID | ACCESSION ID | ORGANISMS | CLASS | PROTEIN FUNCTION | PROTEIN ID | %IDENTITY |
| **Matched Family** | [30687](http://www.ncbi.nlm.nih.gov/bioproject?db=bioproject&cmd=ShowDetailView&TermToSearch=30687) | [AM933172](http://www.ncbi.nlm.nih.gov/nuccore/AM933172) | Salmonella enterica subsp. enterica serovar Enteritidis str. P125109 complete genome. | Gammaproteobacteria | transmembrane efflux protein | [CAR31963](http://www.ncbi.nlm.nih.gov/protein/CAR31963.1) | 100.0 |
|  | | | | | | | |
|  | | | | | | | |
| ------------------ | | | | | | | |
|  | | | | | | | |
|  | | | | | | | |
| **Input Sequence** | SM-Roxy-RH01221661_S26_L001_R1_001_(paired)_contig_21_248 # 248122 # 249291 # -1 # ID=21_248;partial=00;start_type=GTG;rbs_motif=None;rbs_spacer=None;gc_cont=0.592 | | | | | | |
|  | PROJECT ID | ACCESSION ID | ORGANISMS | CLASS | PROTEIN FUNCTION | PROTEIN ID | %IDENTITY |
| **Matched Family** | [30687](http://www.ncbi.nlm.nih.gov/bioproject?db=bioproject&cmd=ShowDetailView&TermToSearch=30687) | [AM933172](http://www.ncbi.nlm.nih.gov/nuccore/AM933172) | Salmonella enterica subsp. enterica serovar Enteritidis str. P125109 complete genome. | Gammaproteobacteria | putative permease transmembrane component | [CAR33743](http://www.ncbi.nlm.nih.gov/protein/CAR33743.1) | 100.0 |
|  | | | | | | | |
|  | | | | | | | |
| ------------------ | | | | | | | |
|  | | | | | | | |
|  | | | | | | | |
| **Input Sequence** | SM-Roxy-RH01221661_S26_L001_R1_001_(paired)_contig_13_65 # 65703 # 66863 # -1 # ID=13_65;partial=00;start_type=ATG;rbs_motif=GGA/GAG/AGG;rbs_spacer=5-10bp;gc_cont=0.477 | | | | | | |
|  | PROJECT ID | ACCESSION ID | ORGANISMS | CLASS | PROTEIN FUNCTION | PROTEIN ID | %IDENTITY |
| **Matched Family** | [30687](http://www.ncbi.nlm.nih.gov/bioproject?db=bioproject&cmd=ShowDetailView&TermToSearch=30687) | [AM933172](http://www.ncbi.nlm.nih.gov/nuccore/AM933172) | Salmonella enterica subsp. enterica serovar Enteritidis str. P125109 complete genome. | Gammaproteobacteria | hypothetical protein | [CAR35741](http://www.ncbi.nlm.nih.gov/protein/CAR35741.1) | 100.0 |
|  | | | | | | | |
|  | | | | | | | |
| ------------------ | | | | | | | |
|  | | | | | | | |
|  | | | | | | | |
| **Input Sequence** | SM-Roxy-RH01221661_S26_L001_R1_001_(paired)_contig_21_44 # 34630 # 35787 # -1 # ID=21_44;partial=00;start_type=ATG;rbs_motif=AGGA/GGAG/GAGG;rbs_spacer=11-12bp;gc_cont=0.491 | | | | | | |
|  | PROJECT ID | ACCESSION ID | ORGANISMS | CLASS | PROTEIN FUNCTION | PROTEIN ID | %IDENTITY |
| **Matched Family** | [30687](http://www.ncbi.nlm.nih.gov/bioproject?db=bioproject&cmd=ShowDetailView&TermToSearch=30687) | [AM933172](http://www.ncbi.nlm.nih.gov/nuccore/AM933172) | Salmonella enterica subsp. enterica serovar Enteritidis str. P125109 complete genome. | Gammaproteobacteria | phage immunity protein | [CAR33537](http://www.ncbi.nlm.nih.gov/protein/CAR33537.1) | 100.0 |
|  | | | | | | | |
|  | | | | | | | |
| ------------------ | | | | | | | |
|  | | | | | | | |
|  | | | | | | | |
| **Input Sequence** | SM-Roxy-RH01221661_S26_L001_R1_001_(paired)_contig_42_18 # 14399 # 15541 # -1 # ID=42_18;partial=00;start_type=ATG;rbs_motif=GGA/GAG/AGG;rbs_spacer=5-10bp;gc_cont=0.401 | | | | | | |
|  | PROJECT ID | ACCESSION ID | ORGANISMS | CLASS | PROTEIN FUNCTION | PROTEIN ID | %IDENTITY |
| **Matched Family** | [30687](http://www.ncbi.nlm.nih.gov/bioproject?db=bioproject&cmd=ShowDetailView&TermToSearch=30687) | [AM933172](http://www.ncbi.nlm.nih.gov/nuccore/AM933172) | Salmonella enterica subsp. enterica serovar Enteritidis str. P125109 complete genome. | Gammaproteobacteria | exported protein | [CAR32575](http://www.ncbi.nlm.nih.gov/protein/CAR32575.1) | 100.0 |
|  | | | | | | | |
|  | | | | | | | |
| ------------------ | | | | | | | |
|  | | | | | | | |
|  | | | | | | | |
| **Input Sequence** | SM-Roxy-RH01221661_S26_L001_R1_001_(paired)_contig_44_101 # 100829 # 101983 # 1 # ID=44_101;partial=00;start_type=ATG;rbs_motif=GGA/GAG/AGG;rbs_spacer=5-10bp;gc_cont=0.489 | | | | | | |
|  | PROJECT ID | ACCESSION ID | ORGANISMS | CLASS | PROTEIN FUNCTION | PROTEIN ID | %IDENTITY |
| **Matched Family** | [30687](http://www.ncbi.nlm.nih.gov/bioproject?db=bioproject&cmd=ShowDetailView&TermToSearch=30687) | [AM933172](http://www.ncbi.nlm.nih.gov/nuccore/AM933172) | Salmonella enterica subsp. enterica serovar Enteritidis str. P125109 complete genome. | Gammaproteobacteria | putative chemo-receptor protein | [CAR32953](http://www.ncbi.nlm.nih.gov/protein/CAR32953.1) | 100.0 |
|  | | | | | | | |
|  | | | | | | | |
| ------------------ | | | | | | | |
|  | | | | | | | |
|  | | | | | | | |
| **Input Sequence** | SM-Roxy-RH01221661_S26_L001_R1_001_(paired)_contig_6_11 # 9663 # 10802 # -1 # ID=6_11;partial=00;start_type=ATG;rbs_motif=GGA/GAG/AGG;rbs_spacer=5-10bp;gc_cont=0.570 | | | | | | |
|  | PROJECT ID | ACCESSION ID | ORGANISMS | CLASS | PROTEIN FUNCTION | PROTEIN ID | %IDENTITY |
| **Matched Family** | [30687](http://www.ncbi.nlm.nih.gov/bioproject?db=bioproject&cmd=ShowDetailView&TermToSearch=30687) | [AM933172](http://www.ncbi.nlm.nih.gov/nuccore/AM933172) | Salmonella enterica subsp. enterica serovar Enteritidis str. P125109 complete genome. | Gammaproteobacteria | conserved hypothetical protein | [CAR31884](http://www.ncbi.nlm.nih.gov/protein/CAR31884.1) | 100.0 |
|  | | | | | | | |
|  | | | | | | | |
| ------------------ | | | | | | | |
|  | | | | | | | |
|  | | | | | | | |
| **Input Sequence** | SM-Roxy-RH01221661_S26_L001_R1_001_(paired)_contig_42_136 # 146972 # 148120 # -1 # ID=42_136;partial=00;start_type=ATG;rbs_motif=None;rbs_spacer=None;gc_cont=0.520 | | | | | | |
|  | PROJECT ID | ACCESSION ID | ORGANISMS | CLASS | PROTEIN FUNCTION | PROTEIN ID | %IDENTITY |
| **Matched Family** | [30687](http://www.ncbi.nlm.nih.gov/bioproject?db=bioproject&cmd=ShowDetailView&TermToSearch=30687) | [AM933172](http://www.ncbi.nlm.nih.gov/nuccore/AM933172) | Salmonella enterica subsp. enterica serovar Enteritidis str. P125109 complete genome. | Gammaproteobacteria | probable transport protein | [CAR32455](http://www.ncbi.nlm.nih.gov/protein/CAR32455.1) | 100.0 |
|  | | | | | | | |
|  | | | | | | | |
| ------------------ | | | | | | | |
|  | | | | | | | |
|  | | | | | | | |
| **Input Sequence** | SM-Roxy-RH01221661_S26_L001_R1_001_(paired)_contig_30_48 # 45867 # 46979 # -1 # ID=30_48;partial=00;start_type=ATG;rbs_motif=None;rbs_spacer=None;gc_cont=0.519 | | | | | | |
|  | PROJECT ID | ACCESSION ID | ORGANISMS | CLASS | PROTEIN FUNCTION | PROTEIN ID | %IDENTITY |
| **Matched Family** | [9618](http://www.ncbi.nlm.nih.gov/bioproject?db=bioproject&cmd=ShowDetailView&TermToSearch=9618) | [AE017220](http://www.ncbi.nlm.nih.gov/nuccore/AE017220) | Salmonella enterica subsp. enterica serovar Choleraesuis str. SC-B67, complete genome. | Gammaproteobacteria | octaprenyl diphosphate synthase | [AAX67149](http://www.ncbi.nlm.nih.gov/protein/AAX67149.1) | 100.0 |
|  | | | | | | | |
|  | | | | | | | |
| ------------------ | | | | | | | |
|  | | | | | | | |
|  | | | | | | | |
| **Input Sequence** | SM-Roxy-RH01221661_S26_L001_R1_001_(paired)_contig_47_23 # 22479 # 23624 # -1 # ID=47_23;partial=00;start_type=ATG;rbs_motif=GGA/GAG/AGG;rbs_spacer=11-12bp;gc_cont=0.413 | | | | | | |
|  | PROJECT ID | ACCESSION ID | ORGANISMS | CLASS | PROTEIN FUNCTION | PROTEIN ID | %IDENTITY |
| **Matched Family** | [19467](http://www.ncbi.nlm.nih.gov/bioproject?db=bioproject&cmd=ShowDetailView&TermToSearch=19467) | [CP001144](http://www.ncbi.nlm.nih.gov/nuccore/CP001144) | Salmonella enterica subsp. enterica serovar Dublin str. C | Gammaproteobacteria | 2021853, complete genome. | [ACH76509](http://www.ncbi.nlm.nih.gov/protein/ACH76509.1) | 100.0 |
|  | | | | | | | |
|  | | | | | | | |
| ------------------ | | | | | | | |
|  | | | | | | | |
|  | | | | | | | |
| **Input Sequence** | SM-Roxy-RH01221661_S26_L001_R1_001_(paired)_contig_13_49 # 46295 # 47434 # 1 # ID=13_49;partial=00;start_type=ATG;rbs_motif=None;rbs_spacer=None;gc_cont=0.496 | | | | | | |
|  | PROJECT ID | ACCESSION ID | ORGANISMS | CLASS | PROTEIN FUNCTION | PROTEIN ID | %IDENTITY |
| **Matched Family** | [18747](http://www.ncbi.nlm.nih.gov/bioproject?db=bioproject&cmd=ShowDetailView&TermToSearch=18747) | [CP001113](http://www.ncbi.nlm.nih.gov/nuccore/CP001113) | Salmonella enterica subsp. enterica serovar Newport str. SL254, complete genome. | Gammaproteobacteria | conserved hypothetical protein | [ACF64443](http://www.ncbi.nlm.nih.gov/protein/ACF64443.1) | 100.0 |
|  | | | | | | | |
|  | | | | | | | |
| ------------------ | | | | | | | |
|  | | | | | | | |
|  | | | | | | | |
| **Input Sequence** | SM-Roxy-RH01221661_S26_L001_R1_001_(paired)_contig_14_51 # 56320 # 57459 # -1 # ID=14_51;partial=00;start_type=ATG;rbs_motif=AGGAG;rbs_spacer=5-10bp;gc_cont=0.574 | | | | | | |
|  | PROJECT ID | ACCESSION ID | ORGANISMS | CLASS | PROTEIN FUNCTION | PROTEIN ID | %IDENTITY |
| **Matched Family** | [19467](http://www.ncbi.nlm.nih.gov/bioproject?db=bioproject&cmd=ShowDetailView&TermToSearch=19467) | [CP001144](http://www.ncbi.nlm.nih.gov/nuccore/CP001144) | Salmonella enterica subsp. enterica serovar Dublin str. C | Gammaproteobacteria | 2021853, complete genome. | [ACH74240](http://www.ncbi.nlm.nih.gov/protein/ACH74240.1) | 100.0 |
|  | | | | | | | |
|  | | | | | | | |
| ------------------ | | | | | | | |
|  | | | | | | | |
|  | | | | | | | |
| **Input Sequence** | SM-Roxy-RH01221661_S26_L001_R1_001_(paired)_contig_24_1 # 199 # 1335 # -1 # ID=24_1;partial=00;start_type=ATG;rbs_motif=GGA/GAG/AGG;rbs_spacer=5-10bp;gc_cont=0.490 | | | | | | |
|  | PROJECT ID | ACCESSION ID | ORGANISMS | CLASS | PROTEIN FUNCTION | PROTEIN ID | %IDENTITY |
| **Matched Family** | [30687](http://www.ncbi.nlm.nih.gov/bioproject?db=bioproject&cmd=ShowDetailView&TermToSearch=30687) | [AM933172](http://www.ncbi.nlm.nih.gov/nuccore/AM933172) | Salmonella enterica subsp. enterica serovar Enteritidis str. P125109 complete genome. | Gammaproteobacteria | ferric enterobactin transport protein | [CAR32146](http://www.ncbi.nlm.nih.gov/protein/CAR32146.1) | 100.0 |
|  | | | | | | | |
|  | | | | | | | |
| ------------------ | | | | | | | |
|  | | | | | | | |
|  | | | | | | | |
| **Input Sequence** | SM-Roxy-RH01221661_S26_L001_R1_001_(paired)_contig_12_123 # 119358 # 120491 # -1 # ID=12_123;partial=00;start_type=ATG;rbs_motif=GGAGG;rbs_spacer=3-4bp;gc_cont=0.484 | | | | | | |
|  | PROJECT ID | ACCESSION ID | ORGANISMS | CLASS | PROTEIN FUNCTION | PROTEIN ID | %IDENTITY |
| **Matched Family** | [30687](http://www.ncbi.nlm.nih.gov/bioproject?db=bioproject&cmd=ShowDetailView&TermToSearch=30687) | [AM933172](http://www.ncbi.nlm.nih.gov/nuccore/AM933172) | Salmonella enterica subsp. enterica serovar Enteritidis str. P125109 complete genome. | Gammaproteobacteria | putative membrane protein | [CAR33371](http://www.ncbi.nlm.nih.gov/protein/CAR33371.1) | 100.0 |
|  | | | | | | | |
|  | | | | | | | |
| ------------------ | | | | | | | |
|  | | | | | | | |
|  | | | | | | | |
| **Input Sequence** | SM-Roxy-RH01221661_S26_L001_R1_001_(paired)_contig_44_58 # 59012 # 60145 # -1 # ID=44_58;partial=00;start_type=ATG;rbs_motif=GGA/GAG/AGG;rbs_spacer=5-10bp;gc_cont=0.554 | | | | | | |
|  | PROJECT ID | ACCESSION ID | ORGANISMS | CLASS | PROTEIN FUNCTION | PROTEIN ID | %IDENTITY |
| **Matched Family** | [30687](http://www.ncbi.nlm.nih.gov/bioproject?db=bioproject&cmd=ShowDetailView&TermToSearch=30687) | [AM933172](http://www.ncbi.nlm.nih.gov/nuccore/AM933172) | Salmonella enterica subsp. enterica serovar Enteritidis str. P125109 complete genome. | Gammaproteobacteria | conserved hypothetical protein | [CAR32909](http://www.ncbi.nlm.nih.gov/protein/CAR32909.1) | 100.0 |
|  | | | | | | | |
|  | | | | | | | |
| ------------------ | | | | | | | |
|  | | | | | | | |
|  | | | | | | | |
| **Input Sequence** | SM-Roxy-RH01221661_S26_L001_R1_001_(paired)_contig_12_105 # 101462 # 102586 # 1 # ID=12_105;partial=00;start_type=ATG;rbs_motif=AGGA;rbs_spacer=5-10bp;gc_cont=0.386 | | | | | | |
|  | PROJECT ID | ACCESSION ID | ORGANISMS | CLASS | PROTEIN FUNCTION | PROTEIN ID | %IDENTITY |
| **Matched Family** | [30687](http://www.ncbi.nlm.nih.gov/bioproject?db=bioproject&cmd=ShowDetailView&TermToSearch=30687) | [AM933172](http://www.ncbi.nlm.nih.gov/nuccore/AM933172) | Salmonella enterica subsp. enterica serovar Enteritidis str. P125109 complete genome. | Gammaproteobacteria | putative bacteriophage protein | [CAR33390](http://www.ncbi.nlm.nih.gov/protein/CAR33390.1) | 100.0 |
|  | | | | | | | |
|  | | | | | | | |
| ------------------ | | | | | | | |
|  | | | | | | | |
|  | | | | | | | |
| **Input Sequence** | SM-Roxy-RH01221661_S26_L001_R1_001_(paired)_contig_4_19 # 18585 # 19709 # 1 # ID=4_19;partial=00;start_type=GTG;rbs_motif=AGGAGG;rbs_spacer=3-4bp;gc_cont=0.574 | | | | | | |
|  | PROJECT ID | ACCESSION ID | ORGANISMS | CLASS | PROTEIN FUNCTION | PROTEIN ID | %IDENTITY |
| **Matched Family** | [19467](http://www.ncbi.nlm.nih.gov/bioproject?db=bioproject&cmd=ShowDetailView&TermToSearch=19467) | [CP001144](http://www.ncbi.nlm.nih.gov/nuccore/CP001144) | Salmonella enterica subsp. enterica serovar Dublin str. C | Gammaproteobacteria | 2021853, complete genome. | [ACH76127](http://www.ncbi.nlm.nih.gov/protein/ACH76127.1) | 100.0 |
|  | | | | | | | |
|  | | | | | | | |
| ------------------ | | | | | | | |
|  | | | | | | | |
|  | | | | | | | |
| **Input Sequence** | SM-Roxy-RH01221661_S26_L001_R1_001_(paired)_contig_60_3 # 2241 # 3365 # -1 # ID=60_3;partial=00;start_type=ATG;rbs_motif=None;rbs_spacer=None;gc_cont=0.519 | | | | | | |
|  | PROJECT ID | ACCESSION ID | ORGANISMS | CLASS | PROTEIN FUNCTION | PROTEIN ID | %IDENTITY |
| **Matched Family** | [20045](http://www.ncbi.nlm.nih.gov/bioproject?db=bioproject&cmd=ShowDetailView&TermToSearch=20045) | [CP001120](http://www.ncbi.nlm.nih.gov/nuccore/CP001120) | Salmonella enterica subsp. enterica serovar Heidelberg str. SL476, complete genome. | Gammaproteobacteria | putative cytoplasmic protein | [ACF67371](http://www.ncbi.nlm.nih.gov/protein/ACF67371.1) | 100.0 |
|  | | | | | | | |
|  | | | | | | | |
| ------------------ | | | | | | | |
|  | | | | | | | |
|  | | | | | | | |
| **Input Sequence** | SM-Roxy-RH01221661_S26_L001_R1_001_(paired)_contig_42_158 # 176976 # 178094 # -1 # ID=42_158;partial=00;start_type=ATG;rbs_motif=GGA/GAG/AGG;rbs_spacer=5-10bp;gc_cont=0.533 | | | | | | |
|  | PROJECT ID | ACCESSION ID | ORGANISMS | CLASS | PROTEIN FUNCTION | PROTEIN ID | %IDENTITY |
| **Matched Family** | [20045](http://www.ncbi.nlm.nih.gov/bioproject?db=bioproject&cmd=ShowDetailView&TermToSearch=20045) | [CP001120](http://www.ncbi.nlm.nih.gov/nuccore/CP001120) | Salmonella enterica subsp. enterica serovar Heidelberg str. SL476, complete genome. | Gammaproteobacteria | macrolide-specific efflux protein MacA | [ACF68953](http://www.ncbi.nlm.nih.gov/protein/ACF68953.1) | 100.0 |
|  | | | | | | | |
|  | | | | | | | |
| ------------------ | | | | | | | |
|  | | | | | | | |
|  | | | | | | | |
| **Input Sequence** | SM-Roxy-RH01221661_S26_L001_R1_001_(paired)_contig_24_7 # 10135 # 11253 # 1 # ID=24_7;partial=00;start_type=ATG;rbs_motif=GGA/GAG/AGG;rbs_spacer=5-10bp;gc_cont=0.539 | | | | | | |
|  | PROJECT ID | ACCESSION ID | ORGANISMS | CLASS | PROTEIN FUNCTION | PROTEIN ID | %IDENTITY |
| **Matched Family** | [30687](http://www.ncbi.nlm.nih.gov/bioproject?db=bioproject&cmd=ShowDetailView&TermToSearch=30687) | [AM933172](http://www.ncbi.nlm.nih.gov/nuccore/AM933172) | Salmonella enterica subsp. enterica serovar Enteritidis str. P125109 complete genome. | Gammaproteobacteria | conserved hypothetical protein | [CAR32140](http://www.ncbi.nlm.nih.gov/protein/CAR32140.1) | 100.0 |
|  | | | | | | | |
|  | | | | | | | |
| ------------------ | | | | | | | |
|  | | | | | | | |
|  | | | | | | | |
| **Input Sequence** | SM-Roxy-RH01221661_S26_L001_R1_001_(paired)_contig_11_297 # 314792 # 315910 # 1 # ID=11_297;partial=00;start_type=ATG;rbs_motif=AGGAG;rbs_spacer=5-10bp;gc_cont=0.453 | | | | | | |
|  | PROJECT ID | ACCESSION ID | ORGANISMS | CLASS | PROTEIN FUNCTION | PROTEIN ID | %IDENTITY |
| **Matched Family** | [18747](http://www.ncbi.nlm.nih.gov/bioproject?db=bioproject&cmd=ShowDetailView&TermToSearch=18747) | [CP001113](http://www.ncbi.nlm.nih.gov/nuccore/CP001113) | Salmonella enterica subsp. enterica serovar Newport str. SL254, complete genome. | Gammaproteobacteria | invasion protein InvE | [ACF63497](http://www.ncbi.nlm.nih.gov/protein/ACF63497.1) | 100.0 |
|  | | | | | | | |
|  | | | | | | | |
| ------------------ | | | | | | | |
|  | | | | | | | |
|  | | | | | | | |
| **Input Sequence** | SM-Roxy-RH01221661_S26_L001_R1_001_(paired)_contig_40_36 # 35473 # 36588 # -1 # ID=40_36;partial=00;start_type=ATG;rbs_motif=GGAG/GAGG;rbs_spacer=5-10bp;gc_cont=0.566 | | | | | | |
|  | PROJECT ID | ACCESSION ID | ORGANISMS | CLASS | PROTEIN FUNCTION | PROTEIN ID | %IDENTITY |
| **Matched Family** | [19467](http://www.ncbi.nlm.nih.gov/bioproject?db=bioproject&cmd=ShowDetailView&TermToSearch=19467) | [CP001144](http://www.ncbi.nlm.nih.gov/nuccore/CP001144) | Salmonella enterica subsp. enterica serovar Dublin str. C | Gammaproteobacteria | 2021853, complete genome. | [ACH74467](http://www.ncbi.nlm.nih.gov/protein/ACH74467.1) | 100.0 |
|  | | | | | | | |
|  | | | | | | | |
| ------------------ | | | | | | | |
|  | | | | | | | |
|  | | | | | | | |
| **Input Sequence** | SM-Roxy-RH01221661_S26_L001_R1_001_(paired)_contig_2_60 # 68176 # 69288 # 1 # ID=2_60;partial=00;start_type=ATG;rbs_motif=3Base/5BMM;rbs_spacer=13-15bp;gc_cont=0.553 | | | | | | |
|  | PROJECT ID | ACCESSION ID | ORGANISMS | CLASS | PROTEIN FUNCTION | PROTEIN ID | %IDENTITY |
| **Matched Family** | [30687](http://www.ncbi.nlm.nih.gov/bioproject?db=bioproject&cmd=ShowDetailView&TermToSearch=30687) | [AM933172](http://www.ncbi.nlm.nih.gov/nuccore/AM933172) | Salmonella enterica subsp. enterica serovar Enteritidis str. P125109 complete genome. | Gammaproteobacteria | adrA protein | [CAR31954](http://www.ncbi.nlm.nih.gov/protein/CAR31954.1) | 100.0 |
|  | | | | | | | |
|  | | | | | | | |
| ------------------ | | | | | | | |
|  | | | | | | | |
|  | | | | | | | |
| **Input Sequence** | SM-Roxy-RH01221661_S26_L001_R1_001_(paired)_contig_21_135 # 116729 # 117841 # 1 # ID=21_135;partial=00;start_type=ATG;rbs_motif=GGAG/GAGG;rbs_spacer=5-10bp;gc_cont=0.606 | | | | | | |
|  | PROJECT ID | ACCESSION ID | ORGANISMS | CLASS | PROTEIN FUNCTION | PROTEIN ID | %IDENTITY |
| **Matched Family** | [30687](http://www.ncbi.nlm.nih.gov/bioproject?db=bioproject&cmd=ShowDetailView&TermToSearch=30687) | [AM933172](http://www.ncbi.nlm.nih.gov/nuccore/AM933172) | Salmonella enterica subsp. enterica serovar Enteritidis str. P125109 complete genome. | Gammaproteobacteria | putative propanol dehydrogenase | [CAR33630](http://www.ncbi.nlm.nih.gov/protein/CAR33630.1) | 100.0 |
|  | | | | | | | |
|  | | | | | | | |
| ------------------ | | | | | | | |
|  | | | | | | | |
|  | | | | | | | |
| **Input Sequence** | SM-Roxy-RH01221661_S26_L001_R1_001_(paired)_contig_38_69 # 69782 # 70894 # 1 # ID=38_69;partial=00;start_type=ATG;rbs_motif=GGA/GAG/AGG;rbs_spacer=11-12bp;gc_cont=0.546 | | | | | | |
|  | PROJECT ID | ACCESSION ID | ORGANISMS | CLASS | PROTEIN FUNCTION | PROTEIN ID | %IDENTITY |
| **Matched Family** | [20045](http://www.ncbi.nlm.nih.gov/bioproject?db=bioproject&cmd=ShowDetailView&TermToSearch=20045) | [CP001120](http://www.ncbi.nlm.nih.gov/nuccore/CP001120) | Salmonella enterica subsp. enterica serovar Heidelberg str. SL476, complete genome. | Gammaproteobacteria | citrate transporter family protein | [ACF67648](http://www.ncbi.nlm.nih.gov/protein/ACF67648.1) | 100.0 |
|  | | | | | | | |
|  | | | | | | | |
| ------------------ | | | | | | | |
|  | | | | | | | |
|  | | | | | | | |
| **Input Sequence** | SM-Roxy-RH01221661_S26_L001_R1_001_(paired)_contig_21_120 # 104629 # 105423 # -1 # ID=21_120;partial=00;start_type=ATG;rbs_motif=AGxAGG/AGGxGG;rbs_spacer=5-10bp;gc_cont=0.507 | | | | | | |
|  | PROJECT ID | ACCESSION ID | ORGANISMS | CLASS | PROTEIN FUNCTION | PROTEIN ID | %IDENTITY |
| **Matched Family** | [9618](http://www.ncbi.nlm.nih.gov/bioproject?db=bioproject&cmd=ShowDetailView&TermToSearch=9618) | [AE017220](http://www.ncbi.nlm.nih.gov/nuccore/AE017220) | Salmonella enterica subsp. enterica serovar Choleraesuis str. SC-B67, complete genome. | Gammaproteobacteria | Propanediol utilization: propanediol diffusion facilitator | [AAX65951](http://www.ncbi.nlm.nih.gov/protein/AAX65951.1) | 100.0 |
|  | | | | | | | |
|  | | | | | | | |
| ------------------ | | | | | | | |
|  | | | | | | | |
|  | | | | | | | |
| **Input Sequence** | SM-Roxy-RH01221661_S26_L001_R1_001_(paired)_contig_44_86 # 86510 # 87565 # -1 # ID=44_86;partial=00;start_type=ATG;rbs_motif=GGA/GAG/AGG;rbs_spacer=5-10bp;gc_cont=0.426 | | | | | | |
|  | PROJECT ID | ACCESSION ID | ORGANISMS | CLASS | PROTEIN FUNCTION | PROTEIN ID | %IDENTITY |
| **Matched Family** | [30687](http://www.ncbi.nlm.nih.gov/bioproject?db=bioproject&cmd=ShowDetailView&TermToSearch=30687) | [AM933172](http://www.ncbi.nlm.nih.gov/nuccore/AM933172) | Salmonella enterica subsp. enterica serovar Enteritidis str. P125109 complete genome. | Gammaproteobacteria | hypothetical protein | [CAR32938](http://www.ncbi.nlm.nih.gov/protein/CAR32938.1) | 100.0 |
|  | | | | | | | |
|  | | | | | | | |
| ------------------ | | | | | | | |
|  | | | | | | | |
|  | | | | | | | |
| **Input Sequence** | SM-Roxy-RH01221661_S26_L001_R1_001_(paired)_contig_2_103 # 111070 # 112179 # -1 # ID=2_103;partial=00;start_type=ATG;rbs_motif=GGA/GAG/AGG;rbs_spacer=5-10bp;gc_cont=0.586 | | | | | | |
|  | PROJECT ID | ACCESSION ID | ORGANISMS | CLASS | PROTEIN FUNCTION | PROTEIN ID | %IDENTITY |
| **Matched Family** | [30687](http://www.ncbi.nlm.nih.gov/bioproject?db=bioproject&cmd=ShowDetailView&TermToSearch=30687) | [AM933172](http://www.ncbi.nlm.nih.gov/nuccore/AM933172) | Salmonella enterica subsp. enterica serovar Enteritidis str. P125109 complete genome. | Gammaproteobacteria | probable ATP-binding component of 2-aminoethylphosphonate transporter | [CAR31996](http://www.ncbi.nlm.nih.gov/protein/CAR31996.1) | 100.0 |
|  | | | | | | | |
|  | | | | | | | |
| ------------------ | | | | | | | |
|  | | | | | | | |
|  | | | | | | | |
| **Input Sequence** | SM-Roxy-RH01221661_S26_L001_R1_001_(paired)_contig_60_6 # 6557 # 7663 # 1 # ID=60_6;partial=00;start_type=ATG;rbs_motif=None;rbs_spacer=None;gc_cont=0.594 | | | | | | |
|  | PROJECT ID | ACCESSION ID | ORGANISMS | CLASS | PROTEIN FUNCTION | PROTEIN ID | %IDENTITY |
| **Matched Family** | [30687](http://www.ncbi.nlm.nih.gov/bioproject?db=bioproject&cmd=ShowDetailView&TermToSearch=30687) | [AM933172](http://www.ncbi.nlm.nih.gov/nuccore/AM933172) | Salmonella enterica subsp. enterica serovar Enteritidis str. P125109 complete genome. | Gammaproteobacteria | alanine racemase | [CAR35471](http://www.ncbi.nlm.nih.gov/protein/CAR35471.1) | 100.0 |
|  | | | | | | | |
|  | | | | | | | |
| ------------------ | | | | | | | |
|  | | | | | | | |
|  | | | | | | | |
| **Input Sequence** | SM-Roxy-RH01221661_S26_L001_R1_001_(paired)_contig_42_15 # 10981 # 12084 # -1 # ID=42_15;partial=00;start_type=ATG;rbs_motif=GGAG/GAGG;rbs_spacer=5-10bp;gc_cont=0.464 | | | | | | |
|  | PROJECT ID | ACCESSION ID | ORGANISMS | CLASS | PROTEIN FUNCTION | PROTEIN ID | %IDENTITY |
| **Matched Family** | [30687](http://www.ncbi.nlm.nih.gov/bioproject?db=bioproject&cmd=ShowDetailView&TermToSearch=30687) | [AM933172](http://www.ncbi.nlm.nih.gov/nuccore/AM933172) | Salmonella enterica subsp. enterica serovar Enteritidis str. P125109 complete genome. | Gammaproteobacteria | putative oxidoreductase | [CAR32578](http://www.ncbi.nlm.nih.gov/protein/CAR32578.1) | 100.0 |
|  | | | | | | | |
|  | | | | | | | |
| ------------------ | | | | | | | |
|  | | | | | | | |
|  | | | | | | | |
| **Input Sequence** | SM-Roxy-RH01221661_S26_L001_R1_001_(paired)_contig_29_32 # 31889 # 32992 # 1 # ID=29_32;partial=00;start_type=ATG;rbs_motif=GGAGG;rbs_spacer=5-10bp;gc_cont=0.509 | | | | | | |
|  | PROJECT ID | ACCESSION ID | ORGANISMS | CLASS | PROTEIN FUNCTION | PROTEIN ID | %IDENTITY |
| **Matched Family** | [13086](http://www.ncbi.nlm.nih.gov/bioproject?db=bioproject&cmd=ShowDetailView&TermToSearch=13086) | [CP000026](http://www.ncbi.nlm.nih.gov/nuccore/CP000026) | Salmonella enterica subsp. enterica serovar Paratyphi A str. ATCC 9150, complete genome. | Gammaproteobacteria | conserved hypothetical protein | [AAV76508](http://www.ncbi.nlm.nih.gov/protein/AAV76508.1) | 100.0 |
|  | | | | | | | |
|  | | | | | | | |
| ------------------ | | | | | | | |
|  | | | | | | | |
|  | | | | | | | |
| **Input Sequence** | SM-Roxy-RH01221661_S26_L001_R1_001_(paired)_contig_40_30 # 25114 # 26166 # 1 # ID=40_30;partial=00;start_type=ATG;rbs_motif=GGAG/GAGG;rbs_spacer=5-10bp;gc_cont=0.443 | | | | | | |
|  | PROJECT ID | ACCESSION ID | ORGANISMS | CLASS | PROTEIN FUNCTION | PROTEIN ID | %IDENTITY |
| **Matched Family** | [19467](http://www.ncbi.nlm.nih.gov/bioproject?db=bioproject&cmd=ShowDetailView&TermToSearch=19467) | [CP001144](http://www.ncbi.nlm.nih.gov/nuccore/CP001144) | Salmonella enterica subsp. enterica serovar Dublin str. C | Gammaproteobacteria | 2021853, complete genome. | [ACH75240](http://www.ncbi.nlm.nih.gov/protein/ACH75240.1) | 100.0 |
|  | | | | | | | |
|  | | | | | | | |
| ------------------ | | | | | | | |
|  | | | | | | | |
|  | | | | | | | |
| **Input Sequence** | SM-Roxy-RH01221661_S26_L001_R1_001_(paired)_contig_25_141 # 131909 # 132997 # 1 # ID=25_141;partial=00;start_type=ATG;rbs_motif=AGGAG;rbs_spacer=5-10bp;gc_cont=0.475 | | | | | | |
|  | PROJECT ID | ACCESSION ID | ORGANISMS | CLASS | PROTEIN FUNCTION | PROTEIN ID | %IDENTITY |
| **Matched Family** | [19467](http://www.ncbi.nlm.nih.gov/bioproject?db=bioproject&cmd=ShowDetailView&TermToSearch=19467) | [CP001144](http://www.ncbi.nlm.nih.gov/nuccore/CP001144) | Salmonella enterica subsp. enterica serovar Dublin str. C | Gammaproteobacteria | 2021853, complete genome. | [ACH74585](http://www.ncbi.nlm.nih.gov/protein/ACH74585.1) | 100.0 |
|  | | | | | | | |
|  | | | | | | | |
| ------------------ | | | | | | | |
|  | | | | | | | |
|  | | | | | | | |
| **Input Sequence** | SM-Roxy-RH01221661_S26_L001_R1_001_(paired)_contig_19_33 # 31864 # 32952 # 1 # ID=19_33;partial=00;start_type=ATG;rbs_motif=AGGA;rbs_spacer=5-10bp;gc_cont=0.579 | | | | | | |
|  | PROJECT ID | ACCESSION ID | ORGANISMS | CLASS | PROTEIN FUNCTION | PROTEIN ID | %IDENTITY |
| **Matched Family** | [20993](http://www.ncbi.nlm.nih.gov/bioproject?db=bioproject&cmd=ShowDetailView&TermToSearch=20993) | [CP000857](http://www.ncbi.nlm.nih.gov/nuccore/CP000857) | Salmonella enterica subsp. enterica serovar Paratyphi C strain RKS4594, complete genome. | Gammaproteobacteria | putative oxidoreductase | [ACN44792](http://www.ncbi.nlm.nih.gov/protein/ACN44792.1) | 100.0 |
|  | | | | | | | |
|  | | | | | | | |
| ------------------ | | | | | | | |
|  | | | | | | | |
|  | | | | | | | |
| **Input Sequence** | SM-Roxy-RH01221661_S26_L001_R1_001_(paired)_contig_23_85 # 87330 # 88415 # -1 # ID=23_85;partial=00;start_type=ATG;rbs_motif=AGGA;rbs_spacer=5-10bp;gc_cont=0.521 | | | | | | |
|  | PROJECT ID | ACCESSION ID | ORGANISMS | CLASS | PROTEIN FUNCTION | PROTEIN ID | %IDENTITY |
| **Matched Family** | [30687](http://www.ncbi.nlm.nih.gov/bioproject?db=bioproject&cmd=ShowDetailView&TermToSearch=30687) | [AM933172](http://www.ncbi.nlm.nih.gov/nuccore/AM933172) | Salmonella enterica subsp. enterica serovar Enteritidis str. P125109 complete genome. | Gammaproteobacteria | putative fimbrial subunit | [CAR35900](http://www.ncbi.nlm.nih.gov/protein/CAR35900.1) | 100.0 |
|  | | | | | | | |
|  | | | | | | | |
| ------------------ | | | | | | | |
|  | | | | | | | |
|  | | | | | | | |
| **Input Sequence** | SM-Roxy-RH01221661_S26_L001_R1_001_(paired)_contig_11_251 # 269166 # 270119 # -1 # ID=11_251;partial=00;start_type=ATG;rbs_motif=AGGA;rbs_spacer=5-10bp;gc_cont=0.413 | | | | | | |
|  | PROJECT ID | ACCESSION ID | ORGANISMS | CLASS | PROTEIN FUNCTION | PROTEIN ID | %IDENTITY |
| **Matched Family** | [19467](http://www.ncbi.nlm.nih.gov/bioproject?db=bioproject&cmd=ShowDetailView&TermToSearch=19467) | [CP001144](http://www.ncbi.nlm.nih.gov/nuccore/CP001144) | Salmonella enterica subsp. enterica serovar Dublin str. C | Gammaproteobacteria | 2021853, complete genome. | [ACH75270](http://www.ncbi.nlm.nih.gov/protein/ACH75270.1) | 100.0 |
|  | | | | | | | |
|  | | | | | | | |
| ------------------ | | | | | | | |
|  | | | | | | | |
|  | | | | | | | |
| **Input Sequence** | SM-Roxy-RH01221661_S26_L001_R1_001_(paired)_contig_60_14 # 18141 # 19220 # 1 # ID=60_14;partial=00;start_type=ATG;rbs_motif=GGAG/GAGG;rbs_spacer=5-10bp;gc_cont=0.569 | | | | | | |
|  | PROJECT ID | ACCESSION ID | ORGANISMS | CLASS | PROTEIN FUNCTION | PROTEIN ID | %IDENTITY |
| **Matched Family** | [18747](http://www.ncbi.nlm.nih.gov/bioproject?db=bioproject&cmd=ShowDetailView&TermToSearch=18747) | [CP001113](http://www.ncbi.nlm.nih.gov/nuccore/CP001113) | Salmonella enterica subsp. enterica serovar Newport str. SL254, complete genome. | Gammaproteobacteria | PTS system, fructose family, IIC component | [ACF61367](http://www.ncbi.nlm.nih.gov/protein/ACF61367.1) | 100.0 |
|  | | | | | | | |
|  | | | | | | | |
| ------------------ | | | | | | | |
|  | | | | | | | |
|  | | | | | | | |
| **Input Sequence** | SM-Roxy-RH01221661_S26_L001_R1_001_(paired)_contig_22_140 # 135843 # 136922 # 1 # ID=22_140;partial=00;start_type=GTG;rbs_motif=GGAG/GAGG;rbs_spacer=5-10bp;gc_cont=0.481 | | | | | | |
|  | PROJECT ID | ACCESSION ID | ORGANISMS | CLASS | PROTEIN FUNCTION | PROTEIN ID | %IDENTITY |
| **Matched Family** | [30687](http://www.ncbi.nlm.nih.gov/bioproject?db=bioproject&cmd=ShowDetailView&TermToSearch=30687) | [AM933172](http://www.ncbi.nlm.nih.gov/nuccore/AM933172) | Salmonella enterica subsp. enterica serovar Enteritidis str. P125109 complete genome. | Gammaproteobacteria | putative integrase | [CAR32714](http://www.ncbi.nlm.nih.gov/protein/CAR32714.1) | 100.0 |
|  | | | | | | | |
|  | | | | | | | |
| ------------------ | | | | | | | |
|  | | | | | | | |
|  | | | | | | | |
| **Input Sequence** | SM-Roxy-RH01221661_S26_L001_R1_001_(paired)_contig_9_77 # 87488 # 88567 # 1 # ID=9_77;partial=00;start_type=ATG;rbs_motif=GGAG/GAGG;rbs_spacer=5-10bp;gc_cont=0.528 | | | | | | |
|  | PROJECT ID | ACCESSION ID | ORGANISMS | CLASS | PROTEIN FUNCTION | PROTEIN ID | %IDENTITY |
| **Matched Family** | [30687](http://www.ncbi.nlm.nih.gov/bioproject?db=bioproject&cmd=ShowDetailView&TermToSearch=30687) | [AM933172](http://www.ncbi.nlm.nih.gov/nuccore/AM933172) | Salmonella enterica subsp. enterica serovar Enteritidis str. P125109 complete genome. | Gammaproteobacteria | putative fimbriae | [CAR31767](http://www.ncbi.nlm.nih.gov/protein/CAR31767.1) | 100.0 |
|  | | | | | | | |
|  | | | | | | | |
| ------------------ | | | | | | | |
|  | | | | | | | |
|  | | | | | | | |
| **Input Sequence** | SM-Roxy-RH01221661_S26_L001_R1_001_(paired)_contig_4_15 # 13425 # 14504 # 1 # ID=4_15;partial=00;start_type=ATG;rbs_motif=AGGA;rbs_spacer=5-10bp;gc_cont=0.468 | | | | | | |
|  | PROJECT ID | ACCESSION ID | ORGANISMS | CLASS | PROTEIN FUNCTION | PROTEIN ID | %IDENTITY |
| **Matched Family** | [30687](http://www.ncbi.nlm.nih.gov/bioproject?db=bioproject&cmd=ShowDetailView&TermToSearch=30687) | [AM933172](http://www.ncbi.nlm.nih.gov/nuccore/AM933172) | Salmonella enterica subsp. enterica serovar Enteritidis str. P125109 complete genome. | Gammaproteobacteria | fimbrial protein (LpfD) | [CAR35037](http://www.ncbi.nlm.nih.gov/protein/CAR35037.1) | 100.0 |
|  | | | | | | | |
|  | | | | | | | |
| ------------------ | | | | | | | |
|  | | | | | | | |
|  | | | | | | | |
| **Input Sequence** | SM-Roxy-RH01221661_S26_L001_R1_001_(paired)_contig_55_12 # 13505 # 14548 # -1 # ID=55_12;partial=00;start_type=ATG;rbs_motif=GGAG/GAGG;rbs_spacer=5-10bp;gc_cont=0.528 | | | | | | |
|  | PROJECT ID | ACCESSION ID | ORGANISMS | CLASS | PROTEIN FUNCTION | PROTEIN ID | %IDENTITY |
| **Matched Family** | [20993](http://www.ncbi.nlm.nih.gov/bioproject?db=bioproject&cmd=ShowDetailView&TermToSearch=20993) | [CP000857](http://www.ncbi.nlm.nih.gov/nuccore/CP000857) | Salmonella enterica subsp. enterica serovar Paratyphi C strain RKS4594, complete genome. | Gammaproteobacteria | anaerobic sulfide reductase | [ACN45269](http://www.ncbi.nlm.nih.gov/protein/ACN45269.1) | 100.0 |
|  | | | | | | | |
|  | | | | | | | |
| ------------------ | | | | | | | |
|  | | | | | | | |
|  | | | | | | | |
| **Input Sequence** | SM-Roxy-RH01221661_S26_L001_R1_001_(paired)_contig_11_164 # 164541 # 165617 # 1 # ID=11_164;partial=00;start_type=ATG;rbs_motif=GGA/GAG/AGG;rbs_spacer=5-10bp;gc_cont=0.398 | | | | | | |
|  | PROJECT ID | ACCESSION ID | ORGANISMS | CLASS | PROTEIN FUNCTION | PROTEIN ID | %IDENTITY |
| **Matched Family** | [30687](http://www.ncbi.nlm.nih.gov/bioproject?db=bioproject&cmd=ShowDetailView&TermToSearch=30687) | [AM933172](http://www.ncbi.nlm.nih.gov/nuccore/AM933172) | Salmonella enterica subsp. enterica serovar Enteritidis str. P125109 complete genome. | Gammaproteobacteria | hypothetical protein | [CAR34448](http://www.ncbi.nlm.nih.gov/protein/CAR34448.1) | 100.0 |
|  | | | | | | | |
|  | | | | | | | |
| ------------------ | | | | | | | |
|  | | | | | | | |
|  | | | | | | | |
| **Input Sequence** | SM-Roxy-RH01221661_S26_L001_R1_001_(paired)_contig_20_26 # 29482 # 30555 # 1 # ID=20_26;partial=00;start_type=ATG;rbs_motif=3Base/5BMM;rbs_spacer=13-15bp;gc_cont=0.551 | | | | | | |
|  | PROJECT ID | ACCESSION ID | ORGANISMS | CLASS | PROTEIN FUNCTION | PROTEIN ID | %IDENTITY |
| **Matched Family** | [19467](http://www.ncbi.nlm.nih.gov/bioproject?db=bioproject&cmd=ShowDetailView&TermToSearch=19467) | [CP001144](http://www.ncbi.nlm.nih.gov/nuccore/CP001144) | Salmonella enterica subsp. enterica serovar Dublin str. C | Gammaproteobacteria | 2021853, complete genome. | [ACH76784](http://www.ncbi.nlm.nih.gov/protein/ACH76784.1) | 100.0 |
|  | | | | | | | |
|  | | | | | | | |
| ------------------ | | | | | | | |
|  | | | | | | | |
|  | | | | | | | |
| **Input Sequence** | SM-Roxy-RH01221661_S26_L001_R1_001_(paired)_contig_25_149 # 141447 # 142520 # 1 # ID=25_149;partial=00;start_type=ATG;rbs_motif=GGA/GAG/AGG;rbs_spacer=5-10bp;gc_cont=0.393 | | | | | | |
|  | PROJECT ID | ACCESSION ID | ORGANISMS | CLASS | PROTEIN FUNCTION | PROTEIN ID | %IDENTITY |
| **Matched Family** | [20045](http://www.ncbi.nlm.nih.gov/bioproject?db=bioproject&cmd=ShowDetailView&TermToSearch=20045) | [CP001120](http://www.ncbi.nlm.nih.gov/nuccore/CP001120) | Salmonella enterica subsp. enterica serovar Heidelberg str. SL476, complete genome. | Gammaproteobacteria | putative S-adenosylmethionine/tRNA-ribosyltransferase-isomerase | [ACF69408](http://www.ncbi.nlm.nih.gov/protein/ACF69408.1) | 100.0 |
|  | | | | | | | |
|  | | | | | | | |
| ------------------ | | | | | | | |
|  | | | | | | | |
|  | | | | | | | |
| **Input Sequence** | SM-Roxy-RH01221661_S26_L001_R1_001_(paired)_contig_11_307 # 323789 # 324859 # 1 # ID=11_307;partial=00;start_type=ATG;rbs_motif=GGAG/GAGG;rbs_spacer=5-10bp;gc_cont=0.432 | | | | | | |
|  | PROJECT ID | ACCESSION ID | ORGANISMS | CLASS | PROTEIN FUNCTION | PROTEIN ID | %IDENTITY |
| **Matched Family** | [18747](http://www.ncbi.nlm.nih.gov/bioproject?db=bioproject&cmd=ShowDetailView&TermToSearch=18747) | [CP001113](http://www.ncbi.nlm.nih.gov/nuccore/CP001113) | Salmonella enterica subsp. enterica serovar Newport str. SL254, complete genome. | Gammaproteobacteria | surface presentation of antigens protein SpaS | [ACF62907](http://www.ncbi.nlm.nih.gov/protein/ACF62907.1) | 100.0 |
|  | | | | | | | |
|  | | | | | | | |
| ------------------ | | | | | | | |
|  | | | | | | | |
|  | | | | | | | |
| **Input Sequence** | SM-Roxy-RH01221661_S26_L001_R1_001_(paired)_contig_28_42 # 37332 # 38267 # 1 # ID=28_42;partial=00;start_type=ATG;rbs_motif=AGGAG;rbs_spacer=5-10bp;gc_cont=0.548 | | | | | | |
|  | PROJECT ID | ACCESSION ID | ORGANISMS | CLASS | PROTEIN FUNCTION | PROTEIN ID | %IDENTITY |
| **Matched Family** | [27803](http://www.ncbi.nlm.nih.gov/bioproject?db=bioproject&cmd=ShowDetailView&TermToSearch=27803) | [CP000886](http://www.ncbi.nlm.nih.gov/nuccore/CP000886) | Salmonella enterica subsp. enterica serovar Paratyphi B str. SPB7, complete genome. | Gammaproteobacteria | hypothetical protein | [ABX70883](http://www.ncbi.nlm.nih.gov/protein/ABX70883.1) | 100.0 |
|  | | | | | | | |
|  | | | | | | | |
| ------------------ | | | | | | | |
|  | | | | | | | |
|  | | | | | | | |
| **Input Sequence** | SM-Roxy-RH01221661_S26_L001_R1_001_(paired)_contig_21_169 # 155359 # 156420 # -1 # ID=21_169;partial=00;start_type=ATG;rbs_motif=GGAG/GAGG;rbs_spacer=5-10bp;gc_cont=0.336 | | | | | | |
|  | PROJECT ID | ACCESSION ID | ORGANISMS | CLASS | PROTEIN FUNCTION | PROTEIN ID | %IDENTITY |
| **Matched Family** | [19467](http://www.ncbi.nlm.nih.gov/bioproject?db=bioproject&cmd=ShowDetailView&TermToSearch=19467) | [CP001144](http://www.ncbi.nlm.nih.gov/nuccore/CP001144) | Salmonella enterica subsp. enterica serovar Dublin str. C | Gammaproteobacteria | 2021853, complete genome. | [ACH75867](http://www.ncbi.nlm.nih.gov/protein/ACH75867.1) | 100.0 |
|  | | | | | | | |
|  | | | | | | | |
| ------------------ | | | | | | | |
|  | | | | | | | |
|  | | | | | | | |
| **Input Sequence** | SM-Roxy-RH01221661_S26_L001_R1_001_(paired)_contig_25_184 # 184654 # 185715 # -1 # ID=25_184;partial=00;start_type=ATG;rbs_motif=GGAG/GAGG;rbs_spacer=5-10bp;gc_cont=0.517 | | | | | | |
|  | PROJECT ID | ACCESSION ID | ORGANISMS | CLASS | PROTEIN FUNCTION | PROTEIN ID | %IDENTITY |
| **Matched Family** | [241](http://www.ncbi.nlm.nih.gov/bioproject?db=bioproject&cmd=ShowDetailView&TermToSearch=241) | [AE006468](http://www.ncbi.nlm.nih.gov/nuccore/AE006468) | Salmonella enterica subsp. enterica serovar Typhimurium str. LT2, complete genome. | Gammaproteobacteria | putative periplasmic protein | [AAL20504](http://www.ncbi.nlm.nih.gov/protein/AAL20504.1) | 100.0 |
|  | | | | | | | |
|  | | | | | | | |
| ------------------ | | | | | | | |
|  | | | | | | | |
|  | | | | | | | |
| **Input Sequence** | SM-Roxy-RH01221661_S26_L001_R1_001_(paired)_contig_25_134 # 125477 # 126538 # -1 # ID=25_134;partial=00;start_type=GTG;rbs_motif=GGA/GAG/AGG;rbs_spacer=5-10bp;gc_cont=0.502 | | | | | | |
|  | PROJECT ID | ACCESSION ID | ORGANISMS | CLASS | PROTEIN FUNCTION | PROTEIN ID | %IDENTITY |
| **Matched Family** | [19467](http://www.ncbi.nlm.nih.gov/bioproject?db=bioproject&cmd=ShowDetailView&TermToSearch=19467) | [CP001144](http://www.ncbi.nlm.nih.gov/nuccore/CP001144) | Salmonella enterica subsp. enterica serovar Dublin str. C | Gammaproteobacteria | 2021853, complete genome. | [ACH77067](http://www.ncbi.nlm.nih.gov/protein/ACH77067.1) | 100.0 |
|  | | | | | | | |
|  | | | | | | | |
| ------------------ | | | | | | | |
|  | | | | | | | |
|  | | | | | | | |
| **Input Sequence** | SM-Roxy-RH01221661_S26_L001_R1_001_(paired)_contig_23_39 # 41145 # 42203 # 1 # ID=23_39;partial=00;start_type=ATG;rbs_motif=GGAG/GAGG;rbs_spacer=5-10bp;gc_cont=0.531 | | | | | | |
|  | PROJECT ID | ACCESSION ID | ORGANISMS | CLASS | PROTEIN FUNCTION | PROTEIN ID | %IDENTITY |
| **Matched Family** | [20993](http://www.ncbi.nlm.nih.gov/bioproject?db=bioproject&cmd=ShowDetailView&TermToSearch=20993) | [CP000857](http://www.ncbi.nlm.nih.gov/nuccore/CP000857) | Salmonella enterica subsp. enterica serovar Paratyphi C strain RKS4594, complete genome. | Gammaproteobacteria | putative glucosamine-fructose-6-phosphate aminotransferase | [ACN48721](http://www.ncbi.nlm.nih.gov/protein/ACN48721.1) | 100.0 |
|  | | | | | | | |
|  | | | | | | | |
| ------------------ | | | | | | | |
|  | | | | | | | |
|  | | | | | | | |
| **Input Sequence** | SM-Roxy-RH01221661_S26_L001_R1_001_(paired)_contig_25_26 # 17079 # 18137 # 1 # ID=25_26;partial=00;start_type=ATG;rbs_motif=None;rbs_spacer=None;gc_cont=0.395 | | | | | | |
|  | PROJECT ID | ACCESSION ID | ORGANISMS | CLASS | PROTEIN FUNCTION | PROTEIN ID | %IDENTITY |
| **Matched Family** | [30687](http://www.ncbi.nlm.nih.gov/bioproject?db=bioproject&cmd=ShowDetailView&TermToSearch=30687) | [AM933172](http://www.ncbi.nlm.nih.gov/nuccore/AM933172) | Salmonella enterica subsp. enterica serovar Enteritidis str. P125109 complete genome. | Gammaproteobacteria | putative type III secretion protein | [CAR33205](http://www.ncbi.nlm.nih.gov/protein/CAR33205.1) | 100.0 |
|  | | | | | | | |
|  | | | | | | | |
| ------------------ | | | | | | | |
|  | | | | | | | |
|  | | | | | | | |
| **Input Sequence** | SM-Roxy-RH01221661_S26_L001_R1_001_(paired)_contig_11_53 # 51307 # 52362 # 1 # ID=11_53;partial=00;start_type=ATG;rbs_motif=AGGAG;rbs_spacer=5-10bp;gc_cont=0.428 | | | | | | |
|  | PROJECT ID | ACCESSION ID | ORGANISMS | CLASS | PROTEIN FUNCTION | PROTEIN ID | %IDENTITY |
| **Matched Family** | [19467](http://www.ncbi.nlm.nih.gov/bioproject?db=bioproject&cmd=ShowDetailView&TermToSearch=19467) | [CP001144](http://www.ncbi.nlm.nih.gov/nuccore/CP001144) | Salmonella enterica subsp. enterica serovar Dublin str. C | Gammaproteobacteria | 2021853, complete genome. | [ACH75868](http://www.ncbi.nlm.nih.gov/protein/ACH75868.1) | 100.0 |
|  | | | | | | | |
|  | | | | | | | |
| ------------------ | | | | | | | |
|  | | | | | | | |
|  | | | | | | | |
| **Input Sequence** | SM-Roxy-RH01221661_S26_L001_R1_001_(paired)_contig_8_22 # 20287 # 21342 # 1 # ID=8_22;partial=00;start_type=ATG;rbs_motif=GGA/GAG/AGG;rbs_spacer=5-10bp;gc_cont=0.617 | | | | | | |
|  | PROJECT ID | ACCESSION ID | ORGANISMS | CLASS | PROTEIN FUNCTION | PROTEIN ID | %IDENTITY |
| **Matched Family** | [30687](http://www.ncbi.nlm.nih.gov/bioproject?db=bioproject&cmd=ShowDetailView&TermToSearch=30687) | [AM933172](http://www.ncbi.nlm.nih.gov/nuccore/AM933172) | Salmonella enterica subsp. enterica serovar Enteritidis str. P125109 complete genome. | Gammaproteobacteria | conserved hypothetical pathogenicity island protein | [CAR31854](http://www.ncbi.nlm.nih.gov/protein/CAR31854.1) | 100.0 |
|  | | | | | | | |
|  | | | | | | | |
| ------------------ | | | | | | | |
|  | | | | | | | |
|  | | | | | | | |
| **Input Sequence** | SM-Roxy-RH01221661_S26_L001_R1_001_(paired)_contig_11_49 # 45858 # 46910 # 1 # ID=11_49;partial=00;start_type=ATG;rbs_motif=None;rbs_spacer=None;gc_cont=0.539 | | | | | | |
|  | PROJECT ID | ACCESSION ID | ORGANISMS | CLASS | PROTEIN FUNCTION | PROTEIN ID | %IDENTITY |
| **Matched Family** | [30687](http://www.ncbi.nlm.nih.gov/bioproject?db=bioproject&cmd=ShowDetailView&TermToSearch=30687) | [AM933172](http://www.ncbi.nlm.nih.gov/nuccore/AM933172) | Salmonella enterica subsp. enterica serovar Enteritidis str. P125109 complete genome. | Gammaproteobacteria | possible ABC-transport protein, periplasmic-binding component | [CAR34561](http://www.ncbi.nlm.nih.gov/protein/CAR34561.1) | 100.0 |
|  | | | | | | | |
|  | | | | | | | |
| ------------------ | | | | | | | |
|  | | | | | | | |
|  | | | | | | | |
| **Input Sequence** | SM-Roxy-RH01221661_S26_L001_R1_001_(paired)_contig_4_70 # 87059 # 88108 # -1 # ID=4_70;partial=00;start_type=ATG;rbs_motif=AGGA;rbs_spacer=5-10bp;gc_cont=0.516 | | | | | | |
|  | PROJECT ID | ACCESSION ID | ORGANISMS | CLASS | PROTEIN FUNCTION | PROTEIN ID | %IDENTITY |
| **Matched Family** | [30687](http://www.ncbi.nlm.nih.gov/bioproject?db=bioproject&cmd=ShowDetailView&TermToSearch=30687) | [AM933172](http://www.ncbi.nlm.nih.gov/nuccore/AM933172) | Salmonella enterica subsp. enterica serovar Enteritidis str. P125109 complete genome. | Gammaproteobacteria | putative membrane protein | [CAR34981](http://www.ncbi.nlm.nih.gov/protein/CAR34981.1) | 100.0 |
|  | | | | | | | |
|  | | | | | | | |
| ------------------ | | | | | | | |
|  | | | | | | | |
|  | | | | | | | |
| **Input Sequence** | SM-Roxy-RH01221661_S26_L001_R1_001_(paired)_contig_24_52 # 52271 # 53320 # 1 # ID=24_52;partial=00;start_type=ATG;rbs_motif=AGGA;rbs_spacer=5-10bp;gc_cont=0.499 | | | | | | |
|  | PROJECT ID | ACCESSION ID | ORGANISMS | CLASS | PROTEIN FUNCTION | PROTEIN ID | %IDENTITY |
| **Matched Family** | [30687](http://www.ncbi.nlm.nih.gov/bioproject?db=bioproject&cmd=ShowDetailView&TermToSearch=30687) | [AM933172](http://www.ncbi.nlm.nih.gov/nuccore/AM933172) | Salmonella enterica subsp. enterica serovar Enteritidis str. P125109 complete genome. | Gammaproteobacteria | ureidoglycolate dehydrogenase | [CAR32094](http://www.ncbi.nlm.nih.gov/protein/CAR32094.1) | 100.0 |
|  | | | | | | | |
|  | | | | | | | |
| ------------------ | | | | | | | |
|  | | | | | | | |
|  | | | | | | | |
| **Input Sequence** | SM-Roxy-RH01221661_S26_L001_R1_001_(paired)_contig_12_88 # 83044 # 84090 # -1 # ID=12_88;partial=00;start_type=ATG;rbs_motif=GGA/GAG/AGG;rbs_spacer=5-10bp;gc_cont=0.512 | | | | | | |
|  | PROJECT ID | ACCESSION ID | ORGANISMS | CLASS | PROTEIN FUNCTION | PROTEIN ID | %IDENTITY |
| **Matched Family** | [13086](http://www.ncbi.nlm.nih.gov/bioproject?db=bioproject&cmd=ShowDetailView&TermToSearch=13086) | [CP000026](http://www.ncbi.nlm.nih.gov/nuccore/CP000026) | Salmonella enterica subsp. enterica serovar Paratyphi A str. ATCC 9150, complete genome. | Gammaproteobacteria | spermidine/putrescine-binding periplasmic protein precursor | [AAV77555](http://www.ncbi.nlm.nih.gov/protein/AAV77555.1) | 100.0 |
|  | | | | | | | |
|  | | | | | | | |
| ------------------ | | | | | | | |
|  | | | | | | | |
|  | | | | | | | |
| **Input Sequence** | SM-Roxy-RH01221661_S26_L001_R1_001_(paired)_contig_5_88 # 85224 # 86246 # 1 # ID=5_88;partial=00;start_type=ATG;rbs_motif=GGAG/GAGG;rbs_spacer=5-10bp;gc_cont=0.503 | | | | | | |
|  | PROJECT ID | ACCESSION ID | ORGANISMS | CLASS | PROTEIN FUNCTION | PROTEIN ID | %IDENTITY |
| **Matched Family** | [19467](http://www.ncbi.nlm.nih.gov/bioproject?db=bioproject&cmd=ShowDetailView&TermToSearch=19467) | [CP001144](http://www.ncbi.nlm.nih.gov/nuccore/CP001144) | Salmonella enterica subsp. enterica serovar Dublin str. C | Gammaproteobacteria | 2021853, complete genome. | [ACH77690](http://www.ncbi.nlm.nih.gov/protein/ACH77690.1) | 100.0 |
|  | | | | | | | |
|  | | | | | | | |
| ------------------ | | | | | | | |
|  | | | | | | | |
|  | | | | | | | |
| **Input Sequence** | SM-Roxy-RH01221661_S26_L001_R1_001_(paired)_contig_36_26 # 30496 # 31539 # 1 # ID=36_26;partial=00;start_type=ATG;rbs_motif=None;rbs_spacer=None;gc_cont=0.563 | | | | | | |
|  | PROJECT ID | ACCESSION ID | ORGANISMS | CLASS | PROTEIN FUNCTION | PROTEIN ID | %IDENTITY |
| **Matched Family** | [30687](http://www.ncbi.nlm.nih.gov/bioproject?db=bioproject&cmd=ShowDetailView&TermToSearch=30687) | [AM933172](http://www.ncbi.nlm.nih.gov/nuccore/AM933172) | Salmonella enterica subsp. enterica serovar Enteritidis str. P125109 complete genome. | Gammaproteobacteria | putative exported protein | [CAR34040](http://www.ncbi.nlm.nih.gov/protein/CAR34040.1) | 100.0 |
|  | | | | | | | |
|  | | | | | | | |
| ------------------ | | | | | | | |
|  | | | | | | | |
|  | | | | | | | |
| **Input Sequence** | SM-Roxy-RH01221661_S26_L001_R1_001_(paired)_contig_5_86 # 83154 # 84197 # 1 # ID=5_86;partial=00;start_type=ATG;rbs_motif=GGAGG;rbs_spacer=3-4bp;gc_cont=0.532 | | | | | | |
|  | PROJECT ID | ACCESSION ID | ORGANISMS | CLASS | PROTEIN FUNCTION | PROTEIN ID | %IDENTITY |
| **Matched Family** | [13086](http://www.ncbi.nlm.nih.gov/bioproject?db=bioproject&cmd=ShowDetailView&TermToSearch=13086) | [CP000026](http://www.ncbi.nlm.nih.gov/nuccore/CP000026) | Salmonella enterica subsp. enterica serovar Paratyphi A str. ATCC 9150, complete genome. | Gammaproteobacteria | putative ABC transporter permease protein | [AAV79683](http://www.ncbi.nlm.nih.gov/protein/AAV79683.1) | 100.0 |
|  | | | | | | | |
|  | | | | | | | |
| ------------------ | | | | | | | |
|  | | | | | | | |
|  | | | | | | | |
| **Input Sequence** | SM-Roxy-RH01221661_S26_L001_R1_001_(paired)_contig_53_3 # 2091 # 3059 # 1 # ID=53_3;partial=00;start_type=ATG;rbs_motif=GGA/GAG/AGG;rbs_spacer=5-10bp;gc_cont=0.547 | | | | | | |
|  | PROJECT ID | ACCESSION ID | ORGANISMS | CLASS | PROTEIN FUNCTION | PROTEIN ID | %IDENTITY |
| **Matched Family** | [30687](http://www.ncbi.nlm.nih.gov/bioproject?db=bioproject&cmd=ShowDetailView&TermToSearch=30687) | [AM933172](http://www.ncbi.nlm.nih.gov/nuccore/AM933172) | Salmonella enterica subsp. enterica serovar Enteritidis str. P125109 complete genome. | Gammaproteobacteria | [citrate (PRO-3S)-lyase] ligase | [CAR31649](http://www.ncbi.nlm.nih.gov/protein/CAR31649.1) | 100.0 |
|  | | | | | | | |
|  | | | | | | | |
| ------------------ | | | | | | | |
|  | | | | | | | |
|  | | | | | | | |
| **Input Sequence** | SM-Roxy-RH01221661_S26_L001_R1_001_(paired)_contig_30_91 # 88543 # 89586 # -1 # ID=30_91;partial=00;start_type=ATG;rbs_motif=GGAG/GAGG;rbs_spacer=5-10bp;gc_cont=0.548 | | | | | | |
|  | PROJECT ID | ACCESSION ID | ORGANISMS | CLASS | PROTEIN FUNCTION | PROTEIN ID | %IDENTITY |
| **Matched Family** | [30687](http://www.ncbi.nlm.nih.gov/bioproject?db=bioproject&cmd=ShowDetailView&TermToSearch=30687) | [AM933172](http://www.ncbi.nlm.nih.gov/nuccore/AM933172) | Salmonella enterica subsp. enterica serovar Enteritidis str. P125109 complete genome. | Gammaproteobacteria | galactitol-1-phosphate dehydrogenase | [CAR34672](http://www.ncbi.nlm.nih.gov/protein/CAR34672.1) | 100.0 |
|  | | | | | | | |
|  | | | | | | | |
| ------------------ | | | | | | | |
|  | | | | | | | |
|  | | | | | | | |
| **Input Sequence** | SM-Roxy-RH01221661_S26_L001_R1_001_(paired)_contig_20_38 # 45481 # 46521 # 1 # ID=20_38;partial=00;start_type=ATG;rbs_motif=GGxGG;rbs_spacer=5-10bp;gc_cont=0.547 | | | | | | |
|  | PROJECT ID | ACCESSION ID | ORGANISMS | CLASS | PROTEIN FUNCTION | PROTEIN ID | %IDENTITY |
| **Matched Family** | [27803](http://www.ncbi.nlm.nih.gov/bioproject?db=bioproject&cmd=ShowDetailView&TermToSearch=27803) | [CP000886](http://www.ncbi.nlm.nih.gov/nuccore/CP000886) | Salmonella enterica subsp. enterica serovar Paratyphi B str. SPB7, complete genome. | Gammaproteobacteria | hypothetical protein | [ABX70072](http://www.ncbi.nlm.nih.gov/protein/ABX70072.1) | 100.0 |
|  | | | | | | | |
|  | | | | | | | |
| ------------------ | | | | | | | |
|  | | | | | | | |
|  | | | | | | | |
| **Input Sequence** | SM-Roxy-RH01221661_S26_L001_R1_001_(paired)_contig_5_79 # 76036 # 77076 # 1 # ID=5_79;partial=00;start_type=ATG;rbs_motif=AGGAG;rbs_spacer=5-10bp;gc_cont=0.567 | | | | | | |
|  | PROJECT ID | ACCESSION ID | ORGANISMS | CLASS | PROTEIN FUNCTION | PROTEIN ID | %IDENTITY |
| **Matched Family** | [13086](http://www.ncbi.nlm.nih.gov/bioproject?db=bioproject&cmd=ShowDetailView&TermToSearch=13086) | [CP000026](http://www.ncbi.nlm.nih.gov/nuccore/CP000026) | Salmonella enterica subsp. enterica serovar Paratyphi A str. ATCC 9150, complete genome. | Gammaproteobacteria | hypothetical protein | [AAV79675](http://www.ncbi.nlm.nih.gov/protein/AAV79675.1) | 100.0 |
|  | | | | | | | |
|  | | | | | | | |
| ------------------ | | | | | | | |
|  | | | | | | | |
|  | | | | | | | |
| **Input Sequence** | SM-Roxy-RH01221661_S26_L001_R1_001_(paired)_contig_21_261 # 263570 # 264607 # -1 # ID=21_261;partial=00;start_type=ATG;rbs_motif=AGGAG;rbs_spacer=5-10bp;gc_cont=0.571 | | | | | | |
|  | PROJECT ID | ACCESSION ID | ORGANISMS | CLASS | PROTEIN FUNCTION | PROTEIN ID | %IDENTITY |
| **Matched Family** | [30687](http://www.ncbi.nlm.nih.gov/bioproject?db=bioproject&cmd=ShowDetailView&TermToSearch=30687) | [AM933172](http://www.ncbi.nlm.nih.gov/nuccore/AM933172) | Salmonella enterica subsp. enterica serovar Enteritidis str. P125109 complete genome. | Gammaproteobacteria | putative gentisate 1,2-dioxygenase | [CAR33756](http://www.ncbi.nlm.nih.gov/protein/CAR33756.1) | 100.0 |
|  | | | | | | | |
|  | | | | | | | |
| ------------------ | | | | | | | |
|  | | | | | | | |
|  | | | | | | | |
| **Input Sequence** | SM-Roxy-RH01221661_S26_L001_R1_001_(paired)_contig_4_106 # 122571 # 123608 # 1 # ID=4_106;partial=00;start_type=ATG;rbs_motif=AGGA;rbs_spacer=5-10bp;gc_cont=0.501 | | | | | | |
|  | PROJECT ID | ACCESSION ID | ORGANISMS | CLASS | PROTEIN FUNCTION | PROTEIN ID | %IDENTITY |
| **Matched Family** | [30687](http://www.ncbi.nlm.nih.gov/bioproject?db=bioproject&cmd=ShowDetailView&TermToSearch=30687) | [AM933172](http://www.ncbi.nlm.nih.gov/nuccore/AM933172) | Salmonella enterica subsp. enterica serovar Enteritidis str. P125109 complete genome. | Gammaproteobacteria | putative oxidoreductase | [CAR34944](http://www.ncbi.nlm.nih.gov/protein/CAR34944.1) | 100.0 |
|  | | | | | | | |
|  | | | | | | | |
| ------------------ | | | | | | | |
|  | | | | | | | |
|  | | | | | | | |
| **Input Sequence** | SM-Roxy-RH01221661_S26_L001_R1_001_(paired)_contig_4_101 # 117303 # 118337 # -1 # ID=4_101;partial=00;start_type=ATG;rbs_motif=GGAG/GAGG;rbs_spacer=5-10bp;gc_cont=0.509 | | | | | | |
|  | PROJECT ID | ACCESSION ID | ORGANISMS | CLASS | PROTEIN FUNCTION | PROTEIN ID | %IDENTITY |
| **Matched Family** | [30687](http://www.ncbi.nlm.nih.gov/bioproject?db=bioproject&cmd=ShowDetailView&TermToSearch=30687) | [AM933172](http://www.ncbi.nlm.nih.gov/nuccore/AM933172) | Salmonella enterica subsp. enterica serovar Enteritidis str. P125109 complete genome. | Gammaproteobacteria | puative phophotriesterase | [CAR34949](http://www.ncbi.nlm.nih.gov/protein/CAR34949.1) | 100.0 |
|  | | | | | | | |
|  | | | | | | | |
| ------------------ | | | | | | | |
|  | | | | | | | |
|  | | | | | | | |
| **Input Sequence** | SM-Roxy-RH01221661_S26_L001_R1_001_(paired)_contig_47_16 # 14471 # 15505 # -1 # ID=47_16;partial=00;start_type=ATG;rbs_motif=None;rbs_spacer=None;gc_cont=0.486 | | | | | | |
|  | PROJECT ID | ACCESSION ID | ORGANISMS | CLASS | PROTEIN FUNCTION | PROTEIN ID | %IDENTITY |
| **Matched Family** | [30687](http://www.ncbi.nlm.nih.gov/bioproject?db=bioproject&cmd=ShowDetailView&TermToSearch=30687) | [AM933172](http://www.ncbi.nlm.nih.gov/nuccore/AM933172) | Salmonella enterica subsp. enterica serovar Enteritidis str. P125109 complete genome. | Gammaproteobacteria | putative glycosyltransferase | [CAR35108](http://www.ncbi.nlm.nih.gov/protein/CAR35108.1) | 100.0 |
|  | | | | | | | |
|  | | | | | | | |
| ------------------ | | | | | | | |
|  | | | | | | | |
|  | | | | | | | |
| **Input Sequence** | SM-Roxy-RH01221661_S26_L001_R1_001_(paired)_contig_44_95 # 95458 # 96489 # -1 # ID=44_95;partial=00;start_type=ATG;rbs_motif=AGGAG/GGAGG;rbs_spacer=11-12bp;gc_cont=0.450 | | | | | | |
|  | PROJECT ID | ACCESSION ID | ORGANISMS | CLASS | PROTEIN FUNCTION | PROTEIN ID | %IDENTITY |
| **Matched Family** | [241](http://www.ncbi.nlm.nih.gov/bioproject?db=bioproject&cmd=ShowDetailView&TermToSearch=241) | [AE006468](http://www.ncbi.nlm.nih.gov/nuccore/AE006468) | Salmonella enterica subsp. enterica serovar Typhimurium str. LT2, complete genome. | Gammaproteobacteria | putative integral membrane protein | [AAL20581](http://www.ncbi.nlm.nih.gov/protein/AAL20581.1) | 100.0 |
|  | | | | | | | |
|  | | | | | | | |
| ------------------ | | | | | | | |
|  | | | | | | | |
|  | | | | | | | |
| **Input Sequence** | SM-Roxy-RH01221661_S26_L001_R1_001_(paired)_contig_11_311 # 328606 # 329637 # 1 # ID=11_311;partial=00;start_type=ATG;rbs_motif=GGA/GAG/AGG;rbs_spacer=3-4bp;gc_cont=0.472 | | | | | | |
|  | PROJECT ID | ACCESSION ID | ORGANISMS | CLASS | PROTEIN FUNCTION | PROTEIN ID | %IDENTITY |
| **Matched Family** | [20993](http://www.ncbi.nlm.nih.gov/bioproject?db=bioproject&cmd=ShowDetailView&TermToSearch=20993) | [CP000857](http://www.ncbi.nlm.nih.gov/nuccore/CP000857) | Salmonella enterica subsp. enterica serovar Paratyphi C strain RKS4594, complete genome. | Gammaproteobacteria | cell invasion protein | [ACN47018](http://www.ncbi.nlm.nih.gov/protein/ACN47018.1) | 100.0 |
|  | | | | | | | |
|  | | | | | | | |
| ------------------ | | | | | | | |
|  | | | | | | | |
|  | | | | | | | |
| **Input Sequence** | SM-Roxy-RH01221661_S26_L001_R1_001_(paired)_contig_23_40 # 42221 # 43231 # 1 # ID=23_40;partial=00;start_type=ATG;rbs_motif=AGGAG;rbs_spacer=5-10bp;gc_cont=0.512 | | | | | | |
|  | PROJECT ID | ACCESSION ID | ORGANISMS | CLASS | PROTEIN FUNCTION | PROTEIN ID | %IDENTITY |
| **Matched Family** | [241](http://www.ncbi.nlm.nih.gov/bioproject?db=bioproject&cmd=ShowDetailView&TermToSearch=241) | [AE006468](http://www.ncbi.nlm.nih.gov/nuccore/AE006468) | Salmonella enterica subsp. enterica serovar Typhimurium str. LT2, complete genome. | Gammaproteobacteria | putative glucosamine-fructose-6-phosphate aminotransferase | [AAL23358](http://www.ncbi.nlm.nih.gov/protein/AAL23358.1) | 100.0 |
|  | | | | | | | |
|  | | | | | | | |
| ------------------ | | | | | | | |
|  | | | | | | | |
|  | | | | | | | |
| **Input Sequence** | SM-Roxy-RH01221661_S26_L001_R1_001_(paired)_contig_5_49 # 47840 # 48283 # -1 # ID=5_49;partial=00;start_type=ATG;rbs_motif=GGA/GAG/AGG;rbs_spacer=5-10bp;gc_cont=0.448 | | | | | | |
|  | PROJECT ID | ACCESSION ID | ORGANISMS | CLASS | PROTEIN FUNCTION | PROTEIN ID | %IDENTITY |
| **Matched Family** | [18747](http://www.ncbi.nlm.nih.gov/bioproject?db=bioproject&cmd=ShowDetailView&TermToSearch=18747) | [CP001113](http://www.ncbi.nlm.nih.gov/nuccore/CP001113) | Salmonella enterica subsp. enterica serovar Newport str. SL254, complete genome. | Gammaproteobacteria | putative inner membrane lipoprotein | [ACF63523](http://www.ncbi.nlm.nih.gov/protein/ACF63523.1) | 100.0 |
|  | | | | | | | |
|  | | | | | | | |
| ------------------ | | | | | | | |
|  | | | | | | | |
|  | | | | | | | |
| **Input Sequence** | SM-Roxy-RH01221661_S26_L001_R1_001_(paired)_contig_11_235 # 249782 # 250813 # -1 # ID=11_235;partial=00;start_type=ATG;rbs_motif=GGA/GAG/AGG;rbs_spacer=5-10bp;gc_cont=0.374 | | | | | | |
|  | PROJECT ID | ACCESSION ID | ORGANISMS | CLASS | PROTEIN FUNCTION | PROTEIN ID | %IDENTITY |
| **Matched Family** | [18747](http://www.ncbi.nlm.nih.gov/bioproject?db=bioproject&cmd=ShowDetailView&TermToSearch=18747) | [CP001113](http://www.ncbi.nlm.nih.gov/nuccore/CP001113) | Salmonella enterica subsp. enterica serovar Newport str. SL254, complete genome. | Gammaproteobacteria | conserved hypothetical protein | [ACF65236](http://www.ncbi.nlm.nih.gov/protein/ACF65236.1) | 100.0 |
|  | | | | | | | |
|  | | | | | | | |
| ------------------ | | | | | | | |
|  | | | | | | | |
|  | | | | | | | |
| **Input Sequence** | SM-Roxy-RH01221661_S26_L001_R1_001_(paired)_contig_25_143 # 133893 # 134918 # -1 # ID=25_143;partial=00;start_type=ATG;rbs_motif=AGGA;rbs_spacer=5-10bp;gc_cont=0.479 | | | | | | |
|  | PROJECT ID | ACCESSION ID | ORGANISMS | CLASS | PROTEIN FUNCTION | PROTEIN ID | %IDENTITY |
| **Matched Family** | [18747](http://www.ncbi.nlm.nih.gov/bioproject?db=bioproject&cmd=ShowDetailView&TermToSearch=18747) | [CP001113](http://www.ncbi.nlm.nih.gov/nuccore/CP001113) | Salmonella enterica subsp. enterica serovar Newport str. SL254, complete genome. | Gammaproteobacteria | zinc-type alcohol dehydrogenase | [ACF62413](http://www.ncbi.nlm.nih.gov/protein/ACF62413.1) | 100.0 |
|  | | | | | | | |
|  | | | | | | | |
| ------------------ | | | | | | | |
|  | | | | | | | |
|  | | | | | | | |
| **Input Sequence** | SM-Roxy-RH01221661_S26_L001_R1_001_(paired)_contig_16_24 # 18016 # 18984 # -1 # ID=16_24;partial=00;start_type=ATG;rbs_motif=AGGAG;rbs_spacer=5-10bp;gc_cont=0.542 | | | | | | |
|  | PROJECT ID | ACCESSION ID | ORGANISMS | CLASS | PROTEIN FUNCTION | PROTEIN ID | %IDENTITY |
| **Matched Family** | [241](http://www.ncbi.nlm.nih.gov/bioproject?db=bioproject&cmd=ShowDetailView&TermToSearch=241) | [AE006471](http://www.ncbi.nlm.nih.gov/nuccore/AE006471) | Salmonella enterica subsp. enterica serovar Typhimurium str. LT2 plasmid pSLT, complete sequence. | Gammaproteobacteria | putative phosphoribulokinase / uridine kinase family | [AAL23533](http://www.ncbi.nlm.nih.gov/protein/AAL23533.1) | 100.0 |
|  | | | | | | | |
|  | | | | | | | |
| ------------------ | | | | | | | |
|  | | | | | | | |
|  | | | | | | | |
| **Input Sequence** | SM-Roxy-RH01221661_S26_L001_R1_001_(paired)_contig_12_252 # 254280 # 255302 # -1 # ID=12_252;partial=00;start_type=ATG;rbs_motif=AGGAG;rbs_spacer=5-10bp;gc_cont=0.553 | | | | | | |
|  | PROJECT ID | ACCESSION ID | ORGANISMS | CLASS | PROTEIN FUNCTION | PROTEIN ID | %IDENTITY |
| **Matched Family** | [19467](http://www.ncbi.nlm.nih.gov/bioproject?db=bioproject&cmd=ShowDetailView&TermToSearch=19467) | [CP001144](http://www.ncbi.nlm.nih.gov/nuccore/CP001144) | Salmonella enterica subsp. enterica serovar Dublin str. C | Gammaproteobacteria | 2021853, complete genome. | [ACH76599](http://www.ncbi.nlm.nih.gov/protein/ACH76599.1) | 100.0 |
|  | | | | | | | |
|  | | | | | | | |
| ------------------ | | | | | | | |
|  | | | | | | | |
|  | | | | | | | |
| **Input Sequence** | SM-Roxy-RH01221661_S26_L001_R1_001_(paired)_contig_21_232 # 232019 # 233041 # -1 # ID=21_232;partial=00;start_type=ATG;rbs_motif=GGA/GAG/AGG;rbs_spacer=5-10bp;gc_cont=0.418 | | | | | | |
|  | PROJECT ID | ACCESSION ID | ORGANISMS | CLASS | PROTEIN FUNCTION | PROTEIN ID | %IDENTITY |
| **Matched Family** | [18747](http://www.ncbi.nlm.nih.gov/bioproject?db=bioproject&cmd=ShowDetailView&TermToSearch=18747) | [CP001113](http://www.ncbi.nlm.nih.gov/nuccore/CP001113) | Salmonella enterica subsp. enterica serovar Newport str. SL254, complete genome. | Gammaproteobacteria | conserved hypothetical protein | [ACF63265](http://www.ncbi.nlm.nih.gov/protein/ACF63265.1) | 100.0 |
|  | | | | | | | |
|  | | | | | | | |
| ------------------ | | | | | | | |
|  | | | | | | | |
|  | | | | | | | |
| **Input Sequence** | SM-Roxy-RH01221661_S26_L001_R1_001_(paired)_contig_29_45 # 43238 # 44257 # -1 # ID=29_45;partial=00;start_type=ATG;rbs_motif=AGGA;rbs_spacer=5-10bp;gc_cont=0.556 | | | | | | |
|  | PROJECT ID | ACCESSION ID | ORGANISMS | CLASS | PROTEIN FUNCTION | PROTEIN ID | %IDENTITY |
| **Matched Family** | [18747](http://www.ncbi.nlm.nih.gov/bioproject?db=bioproject&cmd=ShowDetailView&TermToSearch=18747) | [CP001113](http://www.ncbi.nlm.nih.gov/nuccore/CP001113) | Salmonella enterica subsp. enterica serovar Newport str. SL254, complete genome. | Gammaproteobacteria | regulatory protein, LacI | [ACF65183](http://www.ncbi.nlm.nih.gov/protein/ACF65183.1) | 100.0 |
|  | | | | | | | |
|  | | | | | | | |
| ------------------ | | | | | | | |
|  | | | | | | | |
|  | | | | | | | |
| **Input Sequence** | SM-Roxy-RH01221661_S26_L001_R1_001_(paired)_contig_1_56 # 59202 # 60218 # 1 # ID=1_56;partial=00;start_type=ATG;rbs_motif=AGGA/GGAG/GAGG;rbs_spacer=11-12bp;gc_cont=0.548 | | | | | | |
|  | PROJECT ID | ACCESSION ID | ORGANISMS | CLASS | PROTEIN FUNCTION | PROTEIN ID | %IDENTITY |
| **Matched Family** | [30687](http://www.ncbi.nlm.nih.gov/bioproject?db=bioproject&cmd=ShowDetailView&TermToSearch=30687) | [AM933172](http://www.ncbi.nlm.nih.gov/nuccore/AM933172) | Salmonella enterica subsp. enterica serovar Enteritidis str. P125109 complete genome. | Gammaproteobacteria | lysophospholipase L2 | [CAR35332](http://www.ncbi.nlm.nih.gov/protein/CAR35332.1) | 100.0 |
|  | | | | | | | |
|  | | | | | | | |
| ------------------ | | | | | | | |
|  | | | | | | | |
|  | | | | | | | |
| **Input Sequence** | SM-Roxy-RH01221661_S26_L001_R1_001_(paired)_contig_25_16 # 8901 # 9893 # 1 # ID=25_16;partial=00;start_type=ATG;rbs_motif=GGAG/GAGG;rbs_spacer=5-10bp;gc_cont=0.460 | | | | | | |
|  | PROJECT ID | ACCESSION ID | ORGANISMS | CLASS | PROTEIN FUNCTION | PROTEIN ID | %IDENTITY |
| **Matched Family** | [30687](http://www.ncbi.nlm.nih.gov/bioproject?db=bioproject&cmd=ShowDetailView&TermToSearch=30687) | [AM933172](http://www.ncbi.nlm.nih.gov/nuccore/AM933172) | Salmonella enterica subsp. enterica serovar Enteritidis str. P125109 complete genome. | Gammaproteobacteria | putative secretion system protein | [CAR33215](http://www.ncbi.nlm.nih.gov/protein/CAR33215.1) | 100.0 |
|  | | | | | | | |
|  | | | | | | | |
| ------------------ | | | | | | | |
|  | | | | | | | |
|  | | | | | | | |
| **Input Sequence** | SM-Roxy-RH01221661_S26_L001_R1_001_(paired)_contig_25_206 # 210996 # 212009 # -1 # ID=25_206;partial=00;start_type=ATG;rbs_motif=None;rbs_spacer=None;gc_cont=0.549 | | | | | | |
|  | PROJECT ID | ACCESSION ID | ORGANISMS | CLASS | PROTEIN FUNCTION | PROTEIN ID | %IDENTITY |
| **Matched Family** | [20045](http://www.ncbi.nlm.nih.gov/bioproject?db=bioproject&cmd=ShowDetailView&TermToSearch=20045) | [CP001120](http://www.ncbi.nlm.nih.gov/nuccore/CP001120) | Salmonella enterica subsp. enterica serovar Heidelberg str. SL476, complete genome. | Gammaproteobacteria | tellurite resistance protein TehA | [ACF70178](http://www.ncbi.nlm.nih.gov/protein/ACF70178.1) | 100.0 |
|  | | | | | | | |
|  | | | | | | | |
| ------------------ | | | | | | | |
|  | | | | | | | |
|  | | | | | | | |
| **Input Sequence** | SM-Roxy-RH01221661_S26_L001_R1_001_(paired)_contig_42_167 # 189178 # 190191 # 1 # ID=42_167;partial=00;start_type=ATG;rbs_motif=GGA/GAG/AGG;rbs_spacer=5-10bp;gc_cont=0.543 | | | | | | |
|  | PROJECT ID | ACCESSION ID | ORGANISMS | CLASS | PROTEIN FUNCTION | PROTEIN ID | %IDENTITY |
| **Matched Family** | [19467](http://www.ncbi.nlm.nih.gov/bioproject?db=bioproject&cmd=ShowDetailView&TermToSearch=19467) | [CP001144](http://www.ncbi.nlm.nih.gov/nuccore/CP001144) | Salmonella enterica subsp. enterica serovar Dublin str. C | Gammaproteobacteria | 2021853, complete genome. | [ACH76114](http://www.ncbi.nlm.nih.gov/protein/ACH76114.1) | 100.0 |
|  | | | | | | | |
|  | | | | | | | |
| ------------------ | | | | | | | |
|  | | | | | | | |
|  | | | | | | | |
| **Input Sequence** | SM-Roxy-RH01221661_S26_L001_R1_001_(paired)_contig_40_26 # 20741 # 21754 # 1 # ID=40_26;partial=00;start_type=ATG;rbs_motif=None;rbs_spacer=None;gc_cont=0.502 | | | | | | |
|  | PROJECT ID | ACCESSION ID | ORGANISMS | CLASS | PROTEIN FUNCTION | PROTEIN ID | %IDENTITY |
| **Matched Family** | [18747](http://www.ncbi.nlm.nih.gov/bioproject?db=bioproject&cmd=ShowDetailView&TermToSearch=18747) | [CP001113](http://www.ncbi.nlm.nih.gov/nuccore/CP001113) | Salmonella enterica subsp. enterica serovar Newport str. SL254, complete genome. | Gammaproteobacteria | high-affinity nickel transport protein | [ACF64179](http://www.ncbi.nlm.nih.gov/protein/ACF64179.1) | 100.0 |
|  | | | | | | | |
|  | | | | | | | |
| ------------------ | | | | | | | |
|  | | | | | | | |
|  | | | | | | | |
| **Input Sequence** | SM-Roxy-RH01221661_S26_L001_R1_001_(paired)_contig_55_10 # 11659 # 12672 # -1 # ID=55_10;partial=00;start_type=ATG;rbs_motif=AGGAG;rbs_spacer=5-10bp;gc_cont=0.556 | | | | | | |
|  | PROJECT ID | ACCESSION ID | ORGANISMS | CLASS | PROTEIN FUNCTION | PROTEIN ID | %IDENTITY |
| **Matched Family** | [18747](http://www.ncbi.nlm.nih.gov/bioproject?db=bioproject&cmd=ShowDetailView&TermToSearch=18747) | [CP001113](http://www.ncbi.nlm.nih.gov/nuccore/CP001113) | Salmonella enterica subsp. enterica serovar Newport str. SL254, complete genome. | Gammaproteobacteria | sulfite reductase, subunit C | [ACF63694](http://www.ncbi.nlm.nih.gov/protein/ACF63694.1) | 100.0 |
|  | | | | | | | |
|  | | | | | | | |
| ------------------ | | | | | | | |
|  | | | | | | | |
|  | | | | | | | |
| **Input Sequence** | SM-Roxy-RH01221661_S26_L001_R1_001_(paired)_contig_2_104 # 112185 # 113198 # -1 # ID=2_104;partial=00;start_type=ATG;rbs_motif=GGAG/GAGG;rbs_spacer=5-10bp;gc_cont=0.567 | | | | | | |
|  | PROJECT ID | ACCESSION ID | ORGANISMS | CLASS | PROTEIN FUNCTION | PROTEIN ID | %IDENTITY |
| **Matched Family** | [13086](http://www.ncbi.nlm.nih.gov/bioproject?db=bioproject&cmd=ShowDetailView&TermToSearch=13086) | [CP000026](http://www.ncbi.nlm.nih.gov/nuccore/CP000026) | Salmonella enterica subsp. enterica serovar Paratyphi A str. ATCC 9150, complete genome. | Gammaproteobacteria | probable periplasmic binding component of 2-aminoethylphosphonate transporter | [AAV78179](http://www.ncbi.nlm.nih.gov/protein/AAV78179.1) | 100.0 |
|  | | | | | | | |
|  | | | | | | | |
| ------------------ | | | | | | | |
|  | | | | | | | |
|  | | | | | | | |
| **Input Sequence** | SM-Roxy-RH01221661_S26_L001_R1_001_(paired)_contig_21_133 # 114316 # 115326 # 1 # ID=21_133;partial=00;start_type=ATG;rbs_motif=GGA/GAG/AGG;rbs_spacer=5-10bp;gc_cont=0.609 | | | | | | |
|  | PROJECT ID | ACCESSION ID | ORGANISMS | CLASS | PROTEIN FUNCTION | PROTEIN ID | %IDENTITY |
| **Matched Family** | [20063](http://www.ncbi.nlm.nih.gov/bioproject?db=bioproject&cmd=ShowDetailView&TermToSearch=20063) | [CP001138](http://www.ncbi.nlm.nih.gov/nuccore/CP001138) | Salmonella enterica subsp. enterica serovar Agona str. SL483, complete genome. | Gammaproteobacteria | PduO | [ACH51519](http://www.ncbi.nlm.nih.gov/protein/ACH51519.1) | 100.0 |
|  | | | | | | | |
|  | | | | | | | |
| ------------------ | | | | | | | |
|  | | | | | | | |
|  | | | | | | | |
| **Input Sequence** | SM-Roxy-RH01221661_S26_L001_R1_001_(paired)_contig_7_19 # 18034 # 19044 # -1 # ID=7_19;partial=00;start_type=ATG;rbs_motif=AGGA/GGAG/GAGG;rbs_spacer=11-12bp;gc_cont=0.337 | | | | | | |
|  | PROJECT ID | ACCESSION ID | ORGANISMS | CLASS | PROTEIN FUNCTION | PROTEIN ID | %IDENTITY |
| **Matched Family** | [241](http://www.ncbi.nlm.nih.gov/bioproject?db=bioproject&cmd=ShowDetailView&TermToSearch=241) | [AE006468](http://www.ncbi.nlm.nih.gov/nuccore/AE006468) | Salmonella enterica subsp. enterica serovar Typhimurium str. LT2, complete genome. | Gammaproteobacteria | putative cytoplasmic protein | [AAL22985](http://www.ncbi.nlm.nih.gov/protein/AAL22985.1) | 100.0 |
|  | | | | | | | |
|  | | | | | | | |
| ------------------ | | | | | | | |
|  | | | | | | | |
|  | | | | | | | |
| **Input Sequence** | SM-Roxy-RH01221661_S26_L001_R1_001_(paired)_contig_11_302 # 320136 # 321146 # 1 # ID=11_302;partial=00;start_type=ATG;rbs_motif=AGGAG;rbs_spacer=5-10bp;gc_cont=0.490 | | | | | | |
|  | PROJECT ID | ACCESSION ID | ORGANISMS | CLASS | PROTEIN FUNCTION | PROTEIN ID | %IDENTITY |
| **Matched Family** | [20993](http://www.ncbi.nlm.nih.gov/bioproject?db=bioproject&cmd=ShowDetailView&TermToSearch=20993) | [CP000857](http://www.ncbi.nlm.nih.gov/nuccore/CP000857) | Salmonella enterica subsp. enterica serovar Paratyphi C strain RKS4594, complete genome. | Gammaproteobacteria | antigen presentation protein SpaN | [ACN47027](http://www.ncbi.nlm.nih.gov/protein/ACN47027.1) | 100.0 |
|  | | | | | | | |
|  | | | | | | | |
| ------------------ | | | | | | | |
|  | | | | | | | |
|  | | | | | | | |
| **Input Sequence** | SM-Roxy-RH01221661_S26_L001_R1_001_(paired)_contig_12_90 # 85210 # 86220 # -1 # ID=12_90;partial=00;start_type=ATG;rbs_motif=GGA/GAG/AGG;rbs_spacer=5-10bp;gc_cont=0.409 | | | | | | |
|  | PROJECT ID | ACCESSION ID | ORGANISMS | CLASS | PROTEIN FUNCTION | PROTEIN ID | %IDENTITY |
| **Matched Family** | [30687](http://www.ncbi.nlm.nih.gov/bioproject?db=bioproject&cmd=ShowDetailView&TermToSearch=30687) | [AM933172](http://www.ncbi.nlm.nih.gov/nuccore/AM933172) | Salmonella enterica subsp. enterica serovar Enteritidis str. P125109 complete genome. | Gammaproteobacteria | putative virulence determinant | [CAR33405](http://www.ncbi.nlm.nih.gov/protein/CAR33405.1) | 100.0 |
|  | | | | | | | |
|  | | | | | | | |
| ------------------ | | | | | | | |
|  | | | | | | | |
|  | | | | | | | |
| **Input Sequence** | SM-Roxy-RH01221661_S26_L001_R1_001_(paired)_contig_4_18 # 17428 # 18435 # -1 # ID=4_18;partial=00;start_type=ATG;rbs_motif=AGGA;rbs_spacer=5-10bp;gc_cont=0.601 | | | | | | |
|  | PROJECT ID | ACCESSION ID | ORGANISMS | CLASS | PROTEIN FUNCTION | PROTEIN ID | %IDENTITY |
| **Matched Family** | [19467](http://www.ncbi.nlm.nih.gov/bioproject?db=bioproject&cmd=ShowDetailView&TermToSearch=19467) | [CP001144](http://www.ncbi.nlm.nih.gov/nuccore/CP001144) | Salmonella enterica subsp. enterica serovar Dublin str. C | Gammaproteobacteria | 2021853, complete genome. | [ACH75706](http://www.ncbi.nlm.nih.gov/protein/ACH75706.1) | 100.0 |
|  | | | | | | | |
|  | | | | | | | |
| ------------------ | | | | | | | |
|  | | | | | | | |
|  | | | | | | | |
| **Input Sequence** | SM-Roxy-RH01221661_S26_L001_R1_001_(paired)_contig_21_4 # 4914 # 5921 # 1 # ID=21_4;partial=00;start_type=ATG;rbs_motif=GGA/GAG/AGG;rbs_spacer=5-10bp;gc_cont=0.377 | | | | | | |
|  | PROJECT ID | ACCESSION ID | ORGANISMS | CLASS | PROTEIN FUNCTION | PROTEIN ID | %IDENTITY |
| **Matched Family** | [19467](http://www.ncbi.nlm.nih.gov/bioproject?db=bioproject&cmd=ShowDetailView&TermToSearch=19467) | [CP001144](http://www.ncbi.nlm.nih.gov/nuccore/CP001144) | Salmonella enterica subsp. enterica serovar Dublin str. C | Gammaproteobacteria | 2021853, complete genome. | [ACH76035](http://www.ncbi.nlm.nih.gov/protein/ACH76035.1) | 100.0 |
|  | | | | | | | |
|  | | | | | | | |
| ------------------ | | | | | | | |
|  | | | | | | | |
|  | | | | | | | |
| **Input Sequence** | SM-Roxy-RH01221661_S26_L001_R1_001_(paired)_contig_35_6 # 7248 # 8255 # 1 # ID=35_6;partial=00;start_type=ATG;rbs_motif=GGAG/GAGG;rbs_spacer=5-10bp;gc_cont=0.501 | | | | | | |
|  | PROJECT ID | ACCESSION ID | ORGANISMS | CLASS | PROTEIN FUNCTION | PROTEIN ID | %IDENTITY |
| **Matched Family** | [30687](http://www.ncbi.nlm.nih.gov/bioproject?db=bioproject&cmd=ShowDetailView&TermToSearch=30687) | [AM933172](http://www.ncbi.nlm.nih.gov/nuccore/AM933172) | Salmonella enterica subsp. enterica serovar Enteritidis str. P125109 complete genome. | Gammaproteobacteria | fimbrial subunit | [CAR31614](http://www.ncbi.nlm.nih.gov/protein/CAR31614.1) | 100.0 |
|  | | | | | | | |
|  | | | | | | | |
| ------------------ | | | | | | | |
|  | | | | | | | |
|  | | | | | | | |
| **Input Sequence** | SM-Roxy-RH01221661_S26_L001_R1_001_(paired)_contig_24_33 # 33636 # 34643 # -1 # ID=24_33;partial=00;start_type=ATG;rbs_motif=AGGA;rbs_spacer=5-10bp;gc_cont=0.519 | | | | | | |
|  | PROJECT ID | ACCESSION ID | ORGANISMS | CLASS | PROTEIN FUNCTION | PROTEIN ID | %IDENTITY |
| **Matched Family** | [30687](http://www.ncbi.nlm.nih.gov/bioproject?db=bioproject&cmd=ShowDetailView&TermToSearch=30687) | [AM933172](http://www.ncbi.nlm.nih.gov/nuccore/AM933172) | Salmonella enterica subsp. enterica serovar Enteritidis str. P125109 complete genome. | Gammaproteobacteria | FimH protein precursor | [CAR32113](http://www.ncbi.nlm.nih.gov/protein/CAR32113.1) | 100.0 |
|  | | | | | | | |
|  | | | | | | | |
| ------------------ | | | | | | | |
|  | | | | | | | |
|  | | | | | | | |
| **Input Sequence** | SM-Roxy-RH01221661_S26_L001_R1_001_(paired)_contig_11_86 # 87632 # 88639 # 1 # ID=11_86;partial=00;start_type=GTG;rbs_motif=GGAGG;rbs_spacer=5-10bp;gc_cont=0.458 | | | | | | |
|  | PROJECT ID | ACCESSION ID | ORGANISMS | CLASS | PROTEIN FUNCTION | PROTEIN ID | %IDENTITY |
| **Matched Family** | [20045](http://www.ncbi.nlm.nih.gov/bioproject?db=bioproject&cmd=ShowDetailView&TermToSearch=20045) | [CP001120](http://www.ncbi.nlm.nih.gov/nuccore/CP001120) | Salmonella enterica subsp. enterica serovar Heidelberg str. SL476, complete genome. | Gammaproteobacteria | putative periplasmic protein | [ACF68002](http://www.ncbi.nlm.nih.gov/protein/ACF68002.1) | 100.0 |
|  | | | | | | | |
|  | | | | | | | |
| ------------------ | | | | | | | |
|  | | | | | | | |
|  | | | | | | | |
| **Input Sequence** | SM-Roxy-RH01221661_S26_L001_R1_001_(paired)_contig_11_108 # 109613 # 110620 # 1 # ID=11_108;partial=00;start_type=ATG;rbs_motif=GGA/GAG/AGG;rbs_spacer=5-10bp;gc_cont=0.514 | | | | | | |
|  | PROJECT ID | ACCESSION ID | ORGANISMS | CLASS | PROTEIN FUNCTION | PROTEIN ID | %IDENTITY |
| **Matched Family** | [18747](http://www.ncbi.nlm.nih.gov/bioproject?db=bioproject&cmd=ShowDetailView&TermToSearch=18747) | [CP001113](http://www.ncbi.nlm.nih.gov/nuccore/CP001113) | Salmonella enterica subsp. enterica serovar Newport str. SL254, complete genome. | Gammaproteobacteria | ureidoglycolate dehydrogenase | [ACF64560](http://www.ncbi.nlm.nih.gov/protein/ACF64560.1) | 100.0 |
|  | | | | | | | |
|  | | | | | | | |
| ------------------ | | | | | | | |
|  | | | | | | | |
|  | | | | | | | |
| **Input Sequence** | SM-Roxy-RH01221661_S26_L001_R1_001_(paired)_contig_35_13 # 12178 # 13182 # 1 # ID=35_13;partial=00;start_type=ATG;rbs_motif=AGGA;rbs_spacer=5-10bp;gc_cont=0.383 | | | | | | |
|  | PROJECT ID | ACCESSION ID | ORGANISMS | CLASS | PROTEIN FUNCTION | PROTEIN ID | %IDENTITY |
| **Matched Family** | [19467](http://www.ncbi.nlm.nih.gov/bioproject?db=bioproject&cmd=ShowDetailView&TermToSearch=19467) | [CP001144](http://www.ncbi.nlm.nih.gov/nuccore/CP001144) | Salmonella enterica subsp. enterica serovar Dublin str. C | Gammaproteobacteria | 2021853, complete genome. | [ACH76682](http://www.ncbi.nlm.nih.gov/protein/ACH76682.1) | 100.0 |
|  | | | | | | | |
|  | | | | | | | |
| ------------------ | | | | | | | |
|  | | | | | | | |
|  | | | | | | | |
| **Input Sequence** | SM-Roxy-RH01221661_S26_L001_R1_001_(paired)_contig_5_87 # 84197 # 85195 # 1 # ID=5_87;partial=00;start_type=ATG;rbs_motif=GGAG/GAGG;rbs_spacer=5-10bp;gc_cont=0.494 | | | | | | |
|  | PROJECT ID | ACCESSION ID | ORGANISMS | CLASS | PROTEIN FUNCTION | PROTEIN ID | %IDENTITY |
| **Matched Family** | [30687](http://www.ncbi.nlm.nih.gov/bioproject?db=bioproject&cmd=ShowDetailView&TermToSearch=30687) | [AM933172](http://www.ncbi.nlm.nih.gov/nuccore/AM933172) | Salmonella enterica subsp. enterica serovar Enteritidis str. P125109 complete genome. | Gammaproteobacteria | putative ABC transporter, membrane component | [CAR35439](http://www.ncbi.nlm.nih.gov/protein/CAR35439.1) | 100.0 |
|  | | | | | | | |
|  | | | | | | | |
| ------------------ | | | | | | | |
|  | | | | | | | |
|  | | | | | | | |
| **Input Sequence** | SM-Roxy-RH01221661_S26_L001_R1_001_(paired)_contig_29_19 # 17044 # 18045 # -1 # ID=29_19;partial=00;start_type=ATG;rbs_motif=AGGA;rbs_spacer=5-10bp;gc_cont=0.549 | | | | | | |
|  | PROJECT ID | ACCESSION ID | ORGANISMS | CLASS | PROTEIN FUNCTION | PROTEIN ID | %IDENTITY |
| **Matched Family** | [19467](http://www.ncbi.nlm.nih.gov/bioproject?db=bioproject&cmd=ShowDetailView&TermToSearch=19467) | [CP001144](http://www.ncbi.nlm.nih.gov/nuccore/CP001144) | Salmonella enterica subsp. enterica serovar Dublin str. C | Gammaproteobacteria | 2021853, complete genome. | [ACH75252](http://www.ncbi.nlm.nih.gov/protein/ACH75252.1) | 100.0 |
|  | | | | | | | |
|  | | | | | | | |
| ------------------ | | | | | | | |
|  | | | | | | | |
|  | | | | | | | |
| **Input Sequence** | SM-Roxy-RH01221661_S26_L001_R1_001_(paired)_contig_21_170 # 156995 # 157996 # -1 # ID=21_170;partial=00;start_type=ATG;rbs_motif=GGA/GAG/AGG;rbs_spacer=5-10bp;gc_cont=0.330 | | | | | | |
|  | PROJECT ID | ACCESSION ID | ORGANISMS | CLASS | PROTEIN FUNCTION | PROTEIN ID | %IDENTITY |
| **Matched Family** | [19467](http://www.ncbi.nlm.nih.gov/bioproject?db=bioproject&cmd=ShowDetailView&TermToSearch=19467) | [CP001144](http://www.ncbi.nlm.nih.gov/nuccore/CP001144) | Salmonella enterica subsp. enterica serovar Dublin str. C | Gammaproteobacteria | 2021853, complete genome. | [ACH76687](http://www.ncbi.nlm.nih.gov/protein/ACH76687.1) | 100.0 |
|  | | | | | | | |
|  | | | | | | | |
| ------------------ | | | | | | | |
|  | | | | | | | |
|  | | | | | | | |
| **Input Sequence** | SM-Roxy-RH01221661_S26_L001_R1_001_(paired)_contig_60_2 # 1008 # 2006 # -1 # ID=60_2;partial=00;start_type=ATG;rbs_motif=GGA/GAG/AGG;rbs_spacer=5-10bp;gc_cont=0.452 | | | | | | |
|  | PROJECT ID | ACCESSION ID | ORGANISMS | CLASS | PROTEIN FUNCTION | PROTEIN ID | %IDENTITY |
| **Matched Family** | [19467](http://www.ncbi.nlm.nih.gov/bioproject?db=bioproject&cmd=ShowDetailView&TermToSearch=19467) | [CP001144](http://www.ncbi.nlm.nih.gov/nuccore/CP001144) | Salmonella enterica subsp. enterica serovar Dublin str. C | Gammaproteobacteria | 2021853, complete genome. | [ACH74724](http://www.ncbi.nlm.nih.gov/protein/ACH74724.1) | 100.0 |
|  | | | | | | | |
|  | | | | | | | |
| ------------------ | | | | | | | |
|  | | | | | | | |
|  | | | | | | | |
| **Input Sequence** | SM-Roxy-RH01221661_S26_L001_R1_001_(paired)_contig_14_21 # 20789 # 21784 # -1 # ID=14_21;partial=00;start_type=ATG;rbs_motif=GGA/GAG/AGG;rbs_spacer=11-12bp;gc_cont=0.472 | | | | | | |
|  | PROJECT ID | ACCESSION ID | ORGANISMS | CLASS | PROTEIN FUNCTION | PROTEIN ID | %IDENTITY |
| **Matched Family** | [30687](http://www.ncbi.nlm.nih.gov/bioproject?db=bioproject&cmd=ShowDetailView&TermToSearch=30687) | [AM933172](http://www.ncbi.nlm.nih.gov/nuccore/AM933172) | Salmonella enterica subsp. enterica serovar Enteritidis str. P125109 complete genome. | Gammaproteobacteria | conserved hypothetical protein | [CAR32211](http://www.ncbi.nlm.nih.gov/protein/CAR32211.1) | 100.0 |
|  | | | | | | | |
|  | | | | | | | |
| ------------------ | | | | | | | |
|  | | | | | | | |
|  | | | | | | | |
| **Input Sequence** | SM-Roxy-RH01221661_S26_L001_R1_001_(paired)_contig_29_24 # 22036 # 22950 # 1 # ID=29_24;partial=00;start_type=ATG;rbs_motif=GGA/GAG/AGG;rbs_spacer=5-10bp;gc_cont=0.572 | | | | | | |
|  | PROJECT ID | ACCESSION ID | ORGANISMS | CLASS | PROTEIN FUNCTION | PROTEIN ID | %IDENTITY |
| **Matched Family** | [9618](http://www.ncbi.nlm.nih.gov/bioproject?db=bioproject&cmd=ShowDetailView&TermToSearch=9618) | [AE017220](http://www.ncbi.nlm.nih.gov/nuccore/AE017220) | Salmonella enterica subsp. enterica serovar Choleraesuis str. SC-B67, complete genome. | Gammaproteobacteria | acetylCoA carboxylase, beta subunit | [AAX66274](http://www.ncbi.nlm.nih.gov/protein/AAX66274.1) | 100.0 |
|  | | | | | | | |
|  | | | | | | | |
| ------------------ | | | | | | | |
|  | | | | | | | |
|  | | | | | | | |
| **Input Sequence** | SM-Roxy-RH01221661_S26_L001_R1_001_(paired)_contig_25_226 # 231677 # 232600 # 1 # ID=25_226;partial=00;start_type=ATG;rbs_motif=None;rbs_spacer=None;gc_cont=0.548 | | | | | | |
|  | PROJECT ID | ACCESSION ID | ORGANISMS | CLASS | PROTEIN FUNCTION | PROTEIN ID | %IDENTITY |
| **Matched Family** | [9618](http://www.ncbi.nlm.nih.gov/bioproject?db=bioproject&cmd=ShowDetailView&TermToSearch=9618) | [AE017220](http://www.ncbi.nlm.nih.gov/nuccore/AE017220) | Salmonella enterica subsp. enterica serovar Choleraesuis str. SC-B67, complete genome. | Gammaproteobacteria | putative transcriptional regulators, LysR family | [AAX65526](http://www.ncbi.nlm.nih.gov/protein/AAX65526.1) | 100.0 |
|  | | | | | | | |
|  | | | | | | | |
| ------------------ | | | | | | | |
|  | | | | | | | |
|  | | | | | | | |
| **Input Sequence** | SM-Roxy-RH01221661_S26_L001_R1_001_(paired)_contig_30_96 # 93192 # 94046 # -1 # ID=30_96;partial=00;start_type=ATG;rbs_motif=GGAGG;rbs_spacer=3-4bp;gc_cont=0.533 | | | | | | |
|  | PROJECT ID | ACCESSION ID | ORGANISMS | CLASS | PROTEIN FUNCTION | PROTEIN ID | %IDENTITY |
| **Matched Family** | [19467](http://www.ncbi.nlm.nih.gov/bioproject?db=bioproject&cmd=ShowDetailView&TermToSearch=19467) | [CP001144](http://www.ncbi.nlm.nih.gov/nuccore/CP001144) | Salmonella enterica subsp. enterica serovar Dublin str. C | Gammaproteobacteria | 2021853, complete genome. | [ACH76566](http://www.ncbi.nlm.nih.gov/protein/ACH76566.1) | 100.0 |
|  | | | | | | | |
|  | | | | | | | |
| ------------------ | | | | | | | |
|  | | | | | | | |
|  | | | | | | | |
| **Input Sequence** | SM-Roxy-RH01221661_S26_L001_R1_001_(paired)_contig_30_102 # 101597 # 102586 # 1 # ID=30_102;partial=00;start_type=ATG;rbs_motif=GGAG/GAGG;rbs_spacer=5-10bp;gc_cont=0.467 | | | | | | |
|  | PROJECT ID | ACCESSION ID | ORGANISMS | CLASS | PROTEIN FUNCTION | PROTEIN ID | %IDENTITY |
| **Matched Family** | [19459](http://www.ncbi.nlm.nih.gov/bioproject?db=bioproject&cmd=ShowDetailView&TermToSearch=19459) | [CP001127](http://www.ncbi.nlm.nih.gov/nuccore/CP001127) | Salmonella enterica subsp. enterica serovar Schwarzengrund str. CVM19633, complete genome. | Gammaproteobacteria | threonine dehydratase, catabolic | [ACF88857](http://www.ncbi.nlm.nih.gov/protein/ACF88857.1) | 100.0 |
|  | | | | | | | |
|  | | | | | | | |
| ------------------ | | | | | | | |
|  | | | | | | | |
|  | | | | | | | |
| **Input Sequence** | SM-Roxy-RH01221661_S26_L001_R1_001_(paired)_contig_21_37 # 29818 # 30807 # -1 # ID=21_37;partial=00;start_type=GTG;rbs_motif=GGAG/GAGG;rbs_spacer=5-10bp;gc_cont=0.549 | | | | | | |
|  | PROJECT ID | ACCESSION ID | ORGANISMS | CLASS | PROTEIN FUNCTION | PROTEIN ID | %IDENTITY |
| **Matched Family** | [30687](http://www.ncbi.nlm.nih.gov/bioproject?db=bioproject&cmd=ShowDetailView&TermToSearch=30687) | [AM933172](http://www.ncbi.nlm.nih.gov/nuccore/AM933172) | Salmonella enterica subsp. enterica serovar Enteritidis str. P125109 complete genome. | Gammaproteobacteria | phage protein | [CAR33531](http://www.ncbi.nlm.nih.gov/protein/CAR33531.1) | 100.0 |
|  | | | | | | | |
|  | | | | | | | |
| ------------------ | | | | | | | |
|  | | | | | | | |
|  | | | | | | | |
| **Input Sequence** | SM-Roxy-RH01221661_S26_L001_R1_001_(paired)_contig_21_162 # 145954 # 146937 # -1 # ID=21_162;partial=00;start_type=ATG;rbs_motif=GGA/GAG/AGG;rbs_spacer=5-10bp;gc_cont=0.510 | | | | | | |
|  | PROJECT ID | ACCESSION ID | ORGANISMS | CLASS | PROTEIN FUNCTION | PROTEIN ID | %IDENTITY |
| **Matched Family** | [30687](http://www.ncbi.nlm.nih.gov/bioproject?db=bioproject&cmd=ShowDetailView&TermToSearch=30687) | [AM933172](http://www.ncbi.nlm.nih.gov/nuccore/AM933172) | Salmonella enterica subsp. enterica serovar Enteritidis str. P125109 complete genome. | Gammaproteobacteria | polysaccharide chain length regulator | [CAR33657](http://www.ncbi.nlm.nih.gov/protein/CAR33657.1) | 100.0 |
|  | | | | | | | |
|  | | | | | | | |
| ------------------ | | | | | | | |
|  | | | | | | | |
|  | | | | | | | |
| **Input Sequence** | SM-Roxy-RH01221661_S26_L001_R1_001_(paired)_contig_25_207 # 212116 # 213096 # 1 # ID=25_207;partial=00;start_type=ATG;rbs_motif=GGA/GAG/AGG;rbs_spacer=5-10bp;gc_cont=0.516 | | | | | | |
|  | PROJECT ID | ACCESSION ID | ORGANISMS | CLASS | PROTEIN FUNCTION | PROTEIN ID | %IDENTITY |
| **Matched Family** | [30687](http://www.ncbi.nlm.nih.gov/bioproject?db=bioproject&cmd=ShowDetailView&TermToSearch=30687) | [AM933172](http://www.ncbi.nlm.nih.gov/nuccore/AM933172) | Salmonella enterica subsp. enterica serovar Enteritidis str. P125109 complete genome. | Gammaproteobacteria | putative transferase | [CAR33025](http://www.ncbi.nlm.nih.gov/protein/CAR33025.1) | 100.0 |
|  | | | | | | | |
|  | | | | | | | |
| ------------------ | | | | | | | |
|  | | | | | | | |
|  | | | | | | | |
| **Input Sequence** | SM-Roxy-RH01221661_S26_L001_R1_001_(paired)_contig_4_51 # 63324 # 64301 # 1 # ID=4_51;partial=00;start_type=ATG;rbs_motif=GGAG/GAGG;rbs_spacer=5-10bp;gc_cont=0.500 | | | | | | |
|  | PROJECT ID | ACCESSION ID | ORGANISMS | CLASS | PROTEIN FUNCTION | PROTEIN ID | %IDENTITY |
| **Matched Family** | [30687](http://www.ncbi.nlm.nih.gov/bioproject?db=bioproject&cmd=ShowDetailView&TermToSearch=30687) | [AM933172](http://www.ncbi.nlm.nih.gov/nuccore/AM933172) | Salmonella enterica subsp. enterica serovar Enteritidis str. P125109 complete genome. | Gammaproteobacteria | putative phosphosugar-binding protein | [CAR35000](http://www.ncbi.nlm.nih.gov/protein/CAR35000.1) | 100.0 |
|  | | | | | | | |
|  | | | | | | | |
| ------------------ | | | | | | | |
|  | | | | | | | |
|  | | | | | | | |
| **Input Sequence** | SM-Roxy-RH01221661_S26_L001_R1_001_(paired)_contig_54_21 # 21170 # 22144 # 1 # ID=54_21;partial=00;start_type=ATG;rbs_motif=AGGAG;rbs_spacer=5-10bp;gc_cont=0.532 | | | | | | |
|  | PROJECT ID | ACCESSION ID | ORGANISMS | CLASS | PROTEIN FUNCTION | PROTEIN ID | %IDENTITY |
| **Matched Family** | [30687](http://www.ncbi.nlm.nih.gov/bioproject?db=bioproject&cmd=ShowDetailView&TermToSearch=30687) | [AM933172](http://www.ncbi.nlm.nih.gov/nuccore/AM933172) | Salmonella enterica subsp. enterica serovar Enteritidis str. P125109 complete genome. | Gammaproteobacteria | signal peptidase I | [CAR34144](http://www.ncbi.nlm.nih.gov/protein/CAR34144.1) | 100.0 |
|  | | | | | | | |
|  | | | | | | | |
| ------------------ | | | | | | | |
|  | | | | | | | |
|  | | | | | | | |
| **Input Sequence** | SM-Roxy-RH01221661_S26_L001_R1_001_(paired)_contig_38_55 # 56374 # 57348 # 1 # ID=38_55;partial=00;start_type=ATG;rbs_motif=AGGAG;rbs_spacer=5-10bp;gc_cont=0.568 | | | | | | |
|  | PROJECT ID | ACCESSION ID | ORGANISMS | CLASS | PROTEIN FUNCTION | PROTEIN ID | %IDENTITY |
| **Matched Family** | [19467](http://www.ncbi.nlm.nih.gov/bioproject?db=bioproject&cmd=ShowDetailView&TermToSearch=19467) | [CP001144](http://www.ncbi.nlm.nih.gov/nuccore/CP001144) | Salmonella enterica subsp. enterica serovar Dublin str. C | Gammaproteobacteria | 2021853, complete genome. | [ACH73679](http://www.ncbi.nlm.nih.gov/protein/ACH73679.1) | 100.0 |
|  | | | | | | | |
|  | | | | | | | |
| ------------------ | | | | | | | |
|  | | | | | | | |
|  | | | | | | | |
| **Input Sequence** | SM-Roxy-RH01221661_S26_L001_R1_001_(paired)_contig_21_177 # 164467 # 165441 # -1 # ID=21_177;partial=00;start_type=GTG;rbs_motif=GGA/GAG/AGG;rbs_spacer=5-10bp;gc_cont=0.410 | | | | | | |
|  | PROJECT ID | ACCESSION ID | ORGANISMS | CLASS | PROTEIN FUNCTION | PROTEIN ID | %IDENTITY |
| **Matched Family** | [13086](http://www.ncbi.nlm.nih.gov/bioproject?db=bioproject&cmd=ShowDetailView&TermToSearch=13086) | [CP000026](http://www.ncbi.nlm.nih.gov/nuccore/CP000026) | Salmonella enterica subsp. enterica serovar Paratyphi A str. ATCC 9150, complete genome. | Gammaproteobacteria | putative reductase RfbI | [AAV76766](http://www.ncbi.nlm.nih.gov/protein/AAV76766.1) | 100.0 |
|  | | | | | | | |
|  | | | | | | | |
| ------------------ | | | | | | | |
|  | | | | | | | |
|  | | | | | | | |
| **Input Sequence** | SM-Roxy-RH01221661_S26_L001_R1_001_(paired)_contig_21_42 # 33438 # 34412 # -1 # ID=21_42;partial=00;start_type=ATG;rbs_motif=GGAGG;rbs_spacer=5-10bp;gc_cont=0.541 | | | | | | |
|  | PROJECT ID | ACCESSION ID | ORGANISMS | CLASS | PROTEIN FUNCTION | PROTEIN ID | %IDENTITY |
| **Matched Family** | [30687](http://www.ncbi.nlm.nih.gov/bioproject?db=bioproject&cmd=ShowDetailView&TermToSearch=30687) | [AM933172](http://www.ncbi.nlm.nih.gov/nuccore/AM933172) | Salmonella enterica subsp. enterica serovar Enteritidis str. P125109 complete genome. | Gammaproteobacteria | phage protein | [CAR33536](http://www.ncbi.nlm.nih.gov/protein/CAR33536.1) | 100.0 |
|  | | | | | | | |
|  | | | | | | | |
| ------------------ | | | | | | | |
|  | | | | | | | |
|  | | | | | | | |
| **Input Sequence** | SM-Roxy-RH01221661_S26_L001_R1_001_(paired)_contig_4_102 # 118334 # 119305 # -1 # ID=4_102;partial=00;start_type=ATG;rbs_motif=AGGA;rbs_spacer=5-10bp;gc_cont=0.517 | | | | | | |
|  | PROJECT ID | ACCESSION ID | ORGANISMS | CLASS | PROTEIN FUNCTION | PROTEIN ID | %IDENTITY |
| **Matched Family** | [20063](http://www.ncbi.nlm.nih.gov/bioproject?db=bioproject&cmd=ShowDetailView&TermToSearch=20063) | [CP001138](http://www.ncbi.nlm.nih.gov/nuccore/CP001138) | Salmonella enterica subsp. enterica serovar Agona str. SL483, complete genome. | Gammaproteobacteria | putative inner membrane protein | [ACH52173](http://www.ncbi.nlm.nih.gov/protein/ACH52173.1) | 100.0 |
|  | | | | | | | |
|  | | | | | | | |
| ------------------ | | | | | | | |
|  | | | | | | | |
|  | | | | | | | |
| **Input Sequence** | SM-Roxy-RH01221661_S26_L001_R1_001_(paired)_contig_24_91 # 94465 # 95436 # 1 # ID=24_91;partial=00;start_type=ATG;rbs_motif=AGGAG;rbs_spacer=5-10bp;gc_cont=0.560 | | | | | | |
|  | PROJECT ID | ACCESSION ID | ORGANISMS | CLASS | PROTEIN FUNCTION | PROTEIN ID | %IDENTITY |
| **Matched Family** | [19467](http://www.ncbi.nlm.nih.gov/bioproject?db=bioproject&cmd=ShowDetailView&TermToSearch=19467) | [CP001144](http://www.ncbi.nlm.nih.gov/nuccore/CP001144) | Salmonella enterica subsp. enterica serovar Dublin str. C | Gammaproteobacteria | 2021853, complete genome. | [ACH77562](http://www.ncbi.nlm.nih.gov/protein/ACH77562.1) | 100.0 |
|  | | | | | | | |
|  | | | | | | | |
| ------------------ | | | | | | | |
|  | | | | | | | |
|  | | | | | | | |
| **Input Sequence** | SM-Roxy-RH01221661_S26_L001_R1_001_(paired)_contig_42_159 # 178240 # 179190 # 1 # ID=42_159;partial=00;start_type=ATG;rbs_motif=GGA/GAG/AGG;rbs_spacer=5-10bp;gc_cont=0.470 | | | | | | |
|  | PROJECT ID | ACCESSION ID | ORGANISMS | CLASS | PROTEIN FUNCTION | PROTEIN ID | %IDENTITY |
| **Matched Family** | [30687](http://www.ncbi.nlm.nih.gov/bioproject?db=bioproject&cmd=ShowDetailView&TermToSearch=30687) | [AM933172](http://www.ncbi.nlm.nih.gov/nuccore/AM933172) | Salmonella enterica subsp. enterica serovar Enteritidis str. P125109 complete genome. | Gammaproteobacteria | putative virK protein | [CAR32431](http://www.ncbi.nlm.nih.gov/protein/CAR32431.1) | 100.0 |
|  | | | | | | | |
|  | | | | | | | |
| ------------------ | | | | | | | |
|  | | | | | | | |
|  | | | | | | | |
| **Input Sequence** | SM-Roxy-RH01221661_S26_L001_R1_001_(paired)_contig_23_71 # 72743 # 73711 # 1 # ID=23_71;partial=00;start_type=ATG;rbs_motif=AGGAG;rbs_spacer=5-10bp;gc_cont=0.575 | | | | | | |
|  | PROJECT ID | ACCESSION ID | ORGANISMS | CLASS | PROTEIN FUNCTION | PROTEIN ID | %IDENTITY |
| **Matched Family** | [30943](http://www.ncbi.nlm.nih.gov/bioproject?db=bioproject&cmd=ShowDetailView&TermToSearch=30943) | [FM200053](http://www.ncbi.nlm.nih.gov/nuccore/FM200053) | Salmonella enterica subsp. enterica serovar Paratyphi A str. AK | Gammaproteobacteria | 2601 complete genome, strain AK | [CAR62370](http://www.ncbi.nlm.nih.gov/protein/CAR62370.1) | 100.0 |
|  | | | | | | | |
|  | | | | | | | |
| ------------------ | | | | | | | |
|  | | | | | | | |
|  | | | | | | | |
| **Input Sequence** | SM-Roxy-RH01221661_S26_L001_R1_001_(paired)_contig_12_176 # 171985 # 172953 # 1 # ID=12_176;partial=00;start_type=ATG;rbs_motif=GGxGG;rbs_spacer=5-10bp;gc_cont=0.579 | | | | | | |
|  | PROJECT ID | ACCESSION ID | ORGANISMS | CLASS | PROTEIN FUNCTION | PROTEIN ID | %IDENTITY |
| **Matched Family** | [30687](http://www.ncbi.nlm.nih.gov/bioproject?db=bioproject&cmd=ShowDetailView&TermToSearch=30687) | [AM933172](http://www.ncbi.nlm.nih.gov/nuccore/AM933172) | Salmonella enterica subsp. enterica serovar Enteritidis str. P125109 complete genome. | Gammaproteobacteria | succinylglutamate desuccinylase | [CAR33317](http://www.ncbi.nlm.nih.gov/protein/CAR33317.1) | 100.0 |
|  | | | | | | | |
|  | | | | | | | |
| ------------------ | | | | | | | |
|  | | | | | | | |
|  | | | | | | | |
| **Input Sequence** | SM-Roxy-RH01221661_S26_L001_R1_001_(paired)_contig_25_22 # 14356 # 15324 # 1 # ID=25_22;partial=00;start_type=ATG;rbs_motif=GGAG/GAGG;rbs_spacer=5-10bp;gc_cont=0.458 | | | | | | |
|  | PROJECT ID | ACCESSION ID | ORGANISMS | CLASS | PROTEIN FUNCTION | PROTEIN ID | %IDENTITY |
| **Matched Family** | [30687](http://www.ncbi.nlm.nih.gov/bioproject?db=bioproject&cmd=ShowDetailView&TermToSearch=30687) | [AM933172](http://www.ncbi.nlm.nih.gov/nuccore/AM933172) | Salmonella enterica subsp. enterica serovar Enteritidis str. P125109 complete genome. | Gammaproteobacteria | putative type III secretion protein | [CAR33209](http://www.ncbi.nlm.nih.gov/protein/CAR33209.1) | 100.0 |
|  | | | | | | | |
|  | | | | | | | |
| ------------------ | | | | | | | |
|  | | | | | | | |
|  | | | | | | | |
| **Input Sequence** | SM-Roxy-RH01221661_S26_L001_R1_001_(paired)_contig_42_94 # 94099 # 95067 # -1 # ID=42_94;partial=00;start_type=ATG;rbs_motif=GGA/GAG/AGG;rbs_spacer=5-10bp;gc_cont=0.411 | | | | | | |
|  | PROJECT ID | ACCESSION ID | ORGANISMS | CLASS | PROTEIN FUNCTION | PROTEIN ID | %IDENTITY |
| **Matched Family** | [30687](http://www.ncbi.nlm.nih.gov/bioproject?db=bioproject&cmd=ShowDetailView&TermToSearch=30687) | [AM933172](http://www.ncbi.nlm.nih.gov/nuccore/AM933172) | Salmonella enterica subsp. enterica serovar Enteritidis str. P125109 complete genome. | Gammaproteobacteria | putative type III secreted protein | [CAR32499](http://www.ncbi.nlm.nih.gov/protein/CAR32499.1) | 100.0 |
|  | | | | | | | |
|  | | | | | | | |
| ------------------ | | | | | | | |
|  | | | | | | | |
|  | | | | | | | |
| **Input Sequence** | SM-Roxy-RH01221661_S26_L001_R1_001_(paired)_contig_67_16 # 18510 # 19469 # 1 # ID=67_16;partial=00;start_type=ATG;rbs_motif=AGGAG;rbs_spacer=5-10bp;gc_cont=0.525 | | | | | | |
|  | PROJECT ID | ACCESSION ID | ORGANISMS | CLASS | PROTEIN FUNCTION | PROTEIN ID | %IDENTITY |
| **Matched Family** | [30687](http://www.ncbi.nlm.nih.gov/bioproject?db=bioproject&cmd=ShowDetailView&TermToSearch=30687) | [AM933172](http://www.ncbi.nlm.nih.gov/nuccore/AM933172) | Salmonella enterica subsp. enterica serovar Enteritidis str. P125109 complete genome. | Gammaproteobacteria | putative exported protein | [CAR34079](http://www.ncbi.nlm.nih.gov/protein/CAR34079.1) | 100.0 |
|  | | | | | | | |
|  | | | | | | | |
| ------------------ | | | | | | | |
|  | | | | | | | |
|  | | | | | | | |
| **Input Sequence** | SM-Roxy-RH01221661_S26_L001_R1_001_(paired)_contig_42_132 # 142708 # 143667 # -1 # ID=42_132;partial=00;start_type=ATG;rbs_motif=GGAG/GAGG;rbs_spacer=5-10bp;gc_cont=0.335 | | | | | | |
|  | PROJECT ID | ACCESSION ID | ORGANISMS | CLASS | PROTEIN FUNCTION | PROTEIN ID | %IDENTITY |
| **Matched Family** | [30687](http://www.ncbi.nlm.nih.gov/bioproject?db=bioproject&cmd=ShowDetailView&TermToSearch=30687) | [AM933172](http://www.ncbi.nlm.nih.gov/nuccore/AM933172) | Salmonella enterica subsp. enterica serovar Enteritidis str. P125109 complete genome. | Gammaproteobacteria | putative sopD2 type III secretion system effector protein | [CAR32459](http://www.ncbi.nlm.nih.gov/protein/CAR32459.1) | 100.0 |
|  | | | | | | | |
|  | | | | | | | |
| ------------------ | | | | | | | |
|  | | | | | | | |
|  | | | | | | | |
| **Input Sequence** | SM-Roxy-RH01221661_S26_L001_R1_001_(paired)_contig_12_198 # 191850 # 192809 # -1 # ID=12_198;partial=00;start_type=GTG;rbs_motif=GGA/GAG/AGG;rbs_spacer=5-10bp;gc_cont=0.475 | | | | | | |
|  | PROJECT ID | ACCESSION ID | ORGANISMS | CLASS | PROTEIN FUNCTION | PROTEIN ID | %IDENTITY |
| **Matched Family** | [18747](http://www.ncbi.nlm.nih.gov/bioproject?db=bioproject&cmd=ShowDetailView&TermToSearch=18747) | [CP001113](http://www.ncbi.nlm.nih.gov/nuccore/CP001113) | Salmonella enterica subsp. enterica serovar Newport str. SL254, complete genome. | Gammaproteobacteria | putative outer membrane protein | [ACF64448](http://www.ncbi.nlm.nih.gov/protein/ACF64448.1) | 100.0 |
|  | | | | | | | |
|  | | | | | | | |
| ------------------ | | | | | | | |
|  | | | | | | | |
|  | | | | | | | |
| **Input Sequence** | SM-Roxy-RH01221661_S26_L001_R1_001_(paired)_contig_30_62 # 58518 # 59477 # -1 # ID=30_62;partial=00;start_type=ATG;rbs_motif=GGA/GAG/AGG;rbs_spacer=5-10bp;gc_cont=0.392 | | | | | | |
|  | PROJECT ID | ACCESSION ID | ORGANISMS | CLASS | PROTEIN FUNCTION | PROTEIN ID | %IDENTITY |
| **Matched Family** | [19467](http://www.ncbi.nlm.nih.gov/bioproject?db=bioproject&cmd=ShowDetailView&TermToSearch=19467) | [CP001144](http://www.ncbi.nlm.nih.gov/nuccore/CP001144) | Salmonella enterica subsp. enterica serovar Dublin str. C | Gammaproteobacteria | 2021853, complete genome. | [ACH76255](http://www.ncbi.nlm.nih.gov/protein/ACH76255.1) | 100.0 |
|  | | | | | | | |
|  | | | | | | | |
| ------------------ | | | | | | | |
|  | | | | | | | |
|  | | | | | | | |
| **Input Sequence** | SM-Roxy-RH01221661_S26_L001_R1_001_(paired)_contig_19_41 # 40682 # 41638 # 1 # ID=19_41;partial=00;start_type=GTG;rbs_motif=AGGA;rbs_spacer=5-10bp;gc_cont=0.578 | | | | | | |
|  | PROJECT ID | ACCESSION ID | ORGANISMS | CLASS | PROTEIN FUNCTION | PROTEIN ID | %IDENTITY |
| **Matched Family** | [19467](http://www.ncbi.nlm.nih.gov/bioproject?db=bioproject&cmd=ShowDetailView&TermToSearch=19467) | [CP001144](http://www.ncbi.nlm.nih.gov/nuccore/CP001144) | Salmonella enterica subsp. enterica serovar Dublin str. C | Gammaproteobacteria | 2021853, complete genome. | [ACH77991](http://www.ncbi.nlm.nih.gov/protein/ACH77991.1) | 100.0 |
|  | | | | | | | |
|  | | | | | | | |
| ------------------ | | | | | | | |
|  | | | | | | | |
|  | | | | | | | |
| **Input Sequence** | SM-Roxy-RH01221661_S26_L001_R1_001_(paired)_contig_11_110 # 111412 # 111918 # 1 # ID=11_110;partial=00;start_type=ATG;rbs_motif=GGA/GAG/AGG;rbs_spacer=5-10bp;gc_cont=0.452 | | | | | | |
|  | PROJECT ID | ACCESSION ID | ORGANISMS | CLASS | PROTEIN FUNCTION | PROTEIN ID | %IDENTITY |
| **Matched Family** | [241](http://www.ncbi.nlm.nih.gov/bioproject?db=bioproject&cmd=ShowDetailView&TermToSearch=241) | [AE006468](http://www.ncbi.nlm.nih.gov/nuccore/AE006468) | Salmonella enterica subsp. enterica serovar Typhimurium str. LT2, complete genome. | Gammaproteobacteria | putative hydrolase or acyltransferase | [AAL21954](http://www.ncbi.nlm.nih.gov/protein/AAL21954.1) | 100.0 |
|  | | | | | | | |
|  | | | | | | | |
| ------------------ | | | | | | | |
|  | | | | | | | |
|  | | | | | | | |
| **Input Sequence** | SM-Roxy-RH01221661_S26_L001_R1_001_(paired)_contig_19_1 # 34 # 594 # 1 # ID=19_1;partial=00;start_type=ATG;rbs_motif=None;rbs_spacer=None;gc_cont=0.522 | | | | | | |
|  | PROJECT ID | ACCESSION ID | ORGANISMS | CLASS | PROTEIN FUNCTION | PROTEIN ID | %IDENTITY |
| **Matched Family** | [30687](http://www.ncbi.nlm.nih.gov/bioproject?db=bioproject&cmd=ShowDetailView&TermToSearch=30687) | [AM933172](http://www.ncbi.nlm.nih.gov/nuccore/AM933172) | Salmonella enterica subsp. enterica serovar Enteritidis str. P125109 complete genome. | Gammaproteobacteria | lysR-family transcriptional regulator | [CAR32191](http://www.ncbi.nlm.nih.gov/protein/CAR32191.1) | 100.0 |
|  | | | | | | | |
|  | | | | | | | |
| ------------------ | | | | | | | |
|  | | | | | | | |
|  | | | | | | | |
| **Input Sequence** | SM-Roxy-RH01221661_S26_L001_R1_001_(paired)_contig_29_50 # 47538 # 48491 # 1 # ID=29_50;partial=00;start_type=ATG;rbs_motif=GGAGG;rbs_spacer=5-10bp;gc_cont=0.548 | | | | | | |
|  | PROJECT ID | ACCESSION ID | ORGANISMS | CLASS | PROTEIN FUNCTION | PROTEIN ID | %IDENTITY |
| **Matched Family** | [19467](http://www.ncbi.nlm.nih.gov/bioproject?db=bioproject&cmd=ShowDetailView&TermToSearch=19467) | [CP001144](http://www.ncbi.nlm.nih.gov/nuccore/CP001144) | Salmonella enterica subsp. enterica serovar Dublin str. C | Gammaproteobacteria | 2021853, complete genome. | [ACH75227](http://www.ncbi.nlm.nih.gov/protein/ACH75227.1) | 100.0 |
|  | | | | | | | |
|  | | | | | | | |
| ------------------ | | | | | | | |
|  | | | | | | | |
|  | | | | | | | |
| **Input Sequence** | SM-Roxy-RH01221661_S26_L001_R1_001_(paired)_contig_14_18 # 17235 # 18188 # 1 # ID=14_18;partial=00;start_type=ATG;rbs_motif=GGA/GAG/AGG;rbs_spacer=5-10bp;gc_cont=0.522 | | | | | | |
|  | PROJECT ID | ACCESSION ID | ORGANISMS | CLASS | PROTEIN FUNCTION | PROTEIN ID | %IDENTITY |
| **Matched Family** | [19467](http://www.ncbi.nlm.nih.gov/bioproject?db=bioproject&cmd=ShowDetailView&TermToSearch=19467) | [CP001144](http://www.ncbi.nlm.nih.gov/nuccore/CP001144) | Salmonella enterica subsp. enterica serovar Dublin str. C | Gammaproteobacteria | 2021853, complete genome. | [ACH74477](http://www.ncbi.nlm.nih.gov/protein/ACH74477.1) | 100.0 |
|  | | | | | | | |
|  | | | | | | | |
| ------------------ | | | | | | | |
|  | | | | | | | |
|  | | | | | | | |
| **Input Sequence** | SM-Roxy-RH01221661_S26_L001_R1_001_(paired)_contig_9_90 # 101408 # 102361 # -1 # ID=9_90;partial=00;start_type=ATG;rbs_motif=AGxAGG/AGGxGG;rbs_spacer=5-10bp;gc_cont=0.574 | | | | | | |
|  | PROJECT ID | ACCESSION ID | ORGANISMS | CLASS | PROTEIN FUNCTION | PROTEIN ID | %IDENTITY |
| **Matched Family** | [30687](http://www.ncbi.nlm.nih.gov/bioproject?db=bioproject&cmd=ShowDetailView&TermToSearch=30687) | [AM933172](http://www.ncbi.nlm.nih.gov/nuccore/AM933172) | Salmonella enterica subsp. enterica serovar Enteritidis str. P125109 complete genome. | Gammaproteobacteria | 2-keto-3-deoxygluconate permease | [CAR31754](http://www.ncbi.nlm.nih.gov/protein/CAR31754.1) | 100.0 |
|  | | | | | | | |
|  | | | | | | | |
| ------------------ | | | | | | | |
|  | | | | | | | |
|  | | | | | | | |
| **Input Sequence** | SM-Roxy-RH01221661_S26_L001_R1_001_(paired)_contig_11_111 # 111970 # 112302 # 1 # ID=11_111;partial=00;start_type=ATG;rbs_motif=GGA/GAG/AGG;rbs_spacer=5-10bp;gc_cont=0.502 | | | | | | |
|  | PROJECT ID | ACCESSION ID | ORGANISMS | CLASS | PROTEIN FUNCTION | PROTEIN ID | %IDENTITY |
| **Matched Family** | [30689](http://www.ncbi.nlm.nih.gov/bioproject?db=bioproject&cmd=ShowDetailView&TermToSearch=30689) | [AM933173](http://www.ncbi.nlm.nih.gov/nuccore/AM933173) | Salmonella enterica subsp. enterica serovar Gallinarum str. 287/91 complete genome. | Gammaproteobacteria | putative hydrolase | [CAR38779](http://www.ncbi.nlm.nih.gov/protein/CAR38779.1) | 100.0 |
|  | | | | | | | |
|  | | | | | | | |
| ------------------ | | | | | | | |
|  | | | | | | | |
|  | | | | | | | |
| **Input Sequence** | SM-Roxy-RH01221661_S26_L001_R1_001_(paired)_contig_25_199 # 204230 # 205180 # 1 # ID=25_199;partial=00;start_type=ATG;rbs_motif=None;rbs_spacer=None;gc_cont=0.381 | | | | | | |
|  | PROJECT ID | ACCESSION ID | ORGANISMS | CLASS | PROTEIN FUNCTION | PROTEIN ID | %IDENTITY |
| **Matched Family** | [30687](http://www.ncbi.nlm.nih.gov/bioproject?db=bioproject&cmd=ShowDetailView&TermToSearch=30687) | [AM933172](http://www.ncbi.nlm.nih.gov/nuccore/AM933172) | Salmonella enterica subsp. enterica serovar Enteritidis str. P125109 complete genome. | Gammaproteobacteria | hypothetical protein | [CAR33033](http://www.ncbi.nlm.nih.gov/protein/CAR33033.1) | 100.0 |
|  | | | | | | | |
|  | | | | | | | |
| ------------------ | | | | | | | |
|  | | | | | | | |
|  | | | | | | | |
| **Input Sequence** | SM-Roxy-RH01221661_S26_L001_R1_001_(paired)_contig_8_3 # 1882 # 2829 # -1 # ID=8_3;partial=00;start_type=ATG;rbs_motif=GGA/GAG/AGG;rbs_spacer=5-10bp;gc_cont=0.396 | | | | | | |
|  | PROJECT ID | ACCESSION ID | ORGANISMS | CLASS | PROTEIN FUNCTION | PROTEIN ID | %IDENTITY |
| **Matched Family** | [30687](http://www.ncbi.nlm.nih.gov/bioproject?db=bioproject&cmd=ShowDetailView&TermToSearch=30687) | [AM933172](http://www.ncbi.nlm.nih.gov/nuccore/AM933172) | Salmonella enterica subsp. enterica serovar Enteritidis str. P125109 complete genome. | Gammaproteobacteria | LysR-family transcriptional regulator | [CAR31874](http://www.ncbi.nlm.nih.gov/protein/CAR31874.1) | 100.0 |
|  | | | | | | | |
|  | | | | | | | |
| ------------------ | | | | | | | |
|  | | | | | | | |
|  | | | | | | | |
| **Input Sequence** | SM-Roxy-RH01221661_S26_L001_R1_001_(paired)_contig_21_247 # 247182 # 248129 # -1 # ID=21_247;partial=00;start_type=ATG;rbs_motif=GGAG/GAGG;rbs_spacer=5-10bp;gc_cont=0.575 | | | | | | |
|  | PROJECT ID | ACCESSION ID | ORGANISMS | CLASS | PROTEIN FUNCTION | PROTEIN ID | %IDENTITY |
| **Matched Family** | [18747](http://www.ncbi.nlm.nih.gov/bioproject?db=bioproject&cmd=ShowDetailView&TermToSearch=18747) | [CP001113](http://www.ncbi.nlm.nih.gov/nuccore/CP001113) | Salmonella enterica subsp. enterica serovar Newport str. SL254, complete genome. | Gammaproteobacteria | glycine betaine/carnitine/choline transport ATP-binding protein opuCA | [ACF62094](http://www.ncbi.nlm.nih.gov/protein/ACF62094.1) | 100.0 |
|  | | | | | | | |
|  | | | | | | | |
| ------------------ | | | | | | | |
|  | | | | | | | |
|  | | | | | | | |
| **Input Sequence** | SM-Roxy-RH01221661_S26_L001_R1_001_(paired)_contig_15_18 # 17926 # 18828 # 1 # ID=15_18;partial=00;start_type=ATG;rbs_motif=GGAG/GAGG;rbs_spacer=5-10bp;gc_cont=0.535 | | | | | | |
|  | PROJECT ID | ACCESSION ID | ORGANISMS | CLASS | PROTEIN FUNCTION | PROTEIN ID | %IDENTITY |
| **Matched Family** | [18747](http://www.ncbi.nlm.nih.gov/bioproject?db=bioproject&cmd=ShowDetailView&TermToSearch=18747) | [CP001113](http://www.ncbi.nlm.nih.gov/nuccore/CP001113) | Salmonella enterica subsp. enterica serovar Newport str. SL254, complete genome. | Gammaproteobacteria | carboxylate/amino acid/amine transporter | [ACF64978](http://www.ncbi.nlm.nih.gov/protein/ACF64978.1) | 100.0 |
|  | | | | | | | |
|  | | | | | | | |
| ------------------ | | | | | | | |
|  | | | | | | | |
|  | | | | | | | |
| **Input Sequence** | SM-Roxy-RH01221661_S26_L001_R1_001_(paired)_contig_23_107 # 111967 # 112914 # 1 # ID=23_107;partial=00;start_type=ATG;rbs_motif=GGAGG;rbs_spacer=5-10bp;gc_cont=0.404 | | | | | | |
|  | PROJECT ID | ACCESSION ID | ORGANISMS | CLASS | PROTEIN FUNCTION | PROTEIN ID | %IDENTITY |
| **Matched Family** | [19467](http://www.ncbi.nlm.nih.gov/bioproject?db=bioproject&cmd=ShowDetailView&TermToSearch=19467) | [CP001144](http://www.ncbi.nlm.nih.gov/nuccore/CP001144) | Salmonella enterica subsp. enterica serovar Dublin str. C | Gammaproteobacteria | 2021853, complete genome. | [ACH77560](http://www.ncbi.nlm.nih.gov/protein/ACH77560.1) | 100.0 |
|  | | | | | | | |
|  | | | | | | | |
| ------------------ | | | | | | | |
|  | | | | | | | |
|  | | | | | | | |
| **Input Sequence** | SM-Roxy-RH01221661_S26_L001_R1_001_(paired)_contig_23_46 # 48294 # 49238 # -1 # ID=23_46;partial=00;start_type=GTG;rbs_motif=AGGA;rbs_spacer=5-10bp;gc_cont=0.521 | | | | | | |
|  | PROJECT ID | ACCESSION ID | ORGANISMS | CLASS | PROTEIN FUNCTION | PROTEIN ID | %IDENTITY |
| **Matched Family** | [19467](http://www.ncbi.nlm.nih.gov/bioproject?db=bioproject&cmd=ShowDetailView&TermToSearch=19467) | [CP001144](http://www.ncbi.nlm.nih.gov/nuccore/CP001144) | Salmonella enterica subsp. enterica serovar Dublin str. C | Gammaproteobacteria | 2021853, complete genome. | [ACH73981](http://www.ncbi.nlm.nih.gov/protein/ACH73981.1) | 100.0 |
|  | | | | | | | |
|  | | | | | | | |
| ------------------ | | | | | | | |
|  | | | | | | | |
|  | | | | | | | |
| **Input Sequence** | SM-Roxy-RH01221661_S26_L001_R1_001_(paired)_contig_53_83 # 96384 # 97307 # 1 # ID=53_83;partial=00;start_type=ATG;rbs_motif=GGA/GAG/AGG;rbs_spacer=11-12bp;gc_cont=0.519 | | | | | | |
|  | PROJECT ID | ACCESSION ID | ORGANISMS | CLASS | PROTEIN FUNCTION | PROTEIN ID | %IDENTITY |
| **Matched Family** | [20993](http://www.ncbi.nlm.nih.gov/bioproject?db=bioproject&cmd=ShowDetailView&TermToSearch=20993) | [CP000857](http://www.ncbi.nlm.nih.gov/nuccore/CP000857) | Salmonella enterica subsp. enterica serovar Paratyphi C strain RKS4594, complete genome. | Gammaproteobacteria | putative LysR-family transcriptional regulator | [ACN44339](http://www.ncbi.nlm.nih.gov/protein/ACN44339.1) | 100.0 |
|  | | | | | | | |
|  | | | | | | | |
| ------------------ | | | | | | | |
|  | | | | | | | |
|  | | | | | | | |
| **Input Sequence** | SM-Roxy-RH01221661_S26_L001_R1_001_(paired)_contig_3_48 # 52111 # 53049 # 1 # ID=3_48;partial=00;start_type=ATG;rbs_motif=GGAG/GAGG;rbs_spacer=5-10bp;gc_cont=0.469 | | | | | | |
|  | PROJECT ID | ACCESSION ID | ORGANISMS | CLASS | PROTEIN FUNCTION | PROTEIN ID | %IDENTITY |
| **Matched Family** | [19467](http://www.ncbi.nlm.nih.gov/bioproject?db=bioproject&cmd=ShowDetailView&TermToSearch=19467) | [CP001144](http://www.ncbi.nlm.nih.gov/nuccore/CP001144) | Salmonella enterica subsp. enterica serovar Dublin str. C | Gammaproteobacteria | 2021853, complete genome. | [ACH77008](http://www.ncbi.nlm.nih.gov/protein/ACH77008.1) | 100.0 |
|  | | | | | | | |
|  | | | | | | | |
| ------------------ | | | | | | | |
|  | | | | | | | |
|  | | | | | | | |
| **Input Sequence** | SM-Roxy-RH01221661_S26_L001_R1_001_(paired)_contig_30_101 # 100561 # 101499 # 1 # ID=30_101;partial=00;start_type=ATG;rbs_motif=GGA/GAG/AGG;rbs_spacer=5-10bp;gc_cont=0.445 | | | | | | |
|  | PROJECT ID | ACCESSION ID | ORGANISMS | CLASS | PROTEIN FUNCTION | PROTEIN ID | %IDENTITY |
| **Matched Family** | [18747](http://www.ncbi.nlm.nih.gov/bioproject?db=bioproject&cmd=ShowDetailView&TermToSearch=18747) | [CP001113](http://www.ncbi.nlm.nih.gov/nuccore/CP001113) | Salmonella enterica subsp. enterica serovar Newport str. SL254, complete genome. | Gammaproteobacteria | TDC operon transcriptional activator | [ACF62236](http://www.ncbi.nlm.nih.gov/protein/ACF62236.1) | 100.0 |
|  | | | | | | | |
|  | | | | | | | |
| ------------------ | | | | | | | |
|  | | | | | | | |
|  | | | | | | | |
| **Input Sequence** | SM-Roxy-RH01221661_S26_L001_R1_001_(paired)_contig_41_23 # 29180 # 30112 # -1 # ID=41_23;partial=00;start_type=ATG;rbs_motif=GGAG/GAGG;rbs_spacer=5-10bp;gc_cont=0.519 | | | | | | |
|  | PROJECT ID | ACCESSION ID | ORGANISMS | CLASS | PROTEIN FUNCTION | PROTEIN ID | %IDENTITY |
| **Matched Family** | [19467](http://www.ncbi.nlm.nih.gov/bioproject?db=bioproject&cmd=ShowDetailView&TermToSearch=19467) | [CP001144](http://www.ncbi.nlm.nih.gov/nuccore/CP001144) | Salmonella enterica subsp. enterica serovar Dublin str. C | Gammaproteobacteria | 2021853, complete genome. | [ACH75445](http://www.ncbi.nlm.nih.gov/protein/ACH75445.1) | 100.0 |
|  | | | | | | | |
|  | | | | | | | |
| ------------------ | | | | | | | |
|  | | | | | | | |
|  | | | | | | | |
| **Input Sequence** | SM-Roxy-RH01221661_S26_L001_R1_001_(paired)_contig_11_284 # 300733 # 301644 # -1 # ID=11_284;partial=00;start_type=ATG;rbs_motif=AGGAG;rbs_spacer=5-10bp;gc_cont=0.553 | | | | | | |
|  | PROJECT ID | ACCESSION ID | ORGANISMS | CLASS | PROTEIN FUNCTION | PROTEIN ID | %IDENTITY |
| **Matched Family** | [30687](http://www.ncbi.nlm.nih.gov/bioproject?db=bioproject&cmd=ShowDetailView&TermToSearch=30687) | [AM933172](http://www.ncbi.nlm.nih.gov/nuccore/AM933172) | Salmonella enterica subsp. enterica serovar Enteritidis str. P125109 complete genome. | Gammaproteobacteria | possible LysR-family transcriptional regulator | [CAR34330](http://www.ncbi.nlm.nih.gov/protein/CAR34330.1) | 100.0 |
|  | | | | | | | |
|  | | | | | | | |
| ------------------ | | | | | | | |
|  | | | | | | | |
|  | | | | | | | |
| **Input Sequence** | SM-Roxy-RH01221661_S26_L001_R1_001_(paired)_contig_25_265 # 270598 # 271530 # 1 # ID=25_265;partial=00;start_type=ATG;rbs_motif=AGGA;rbs_spacer=5-10bp;gc_cont=0.317 | | | | | | |
|  | PROJECT ID | ACCESSION ID | ORGANISMS | CLASS | PROTEIN FUNCTION | PROTEIN ID | %IDENTITY |
| **Matched Family** | [30687](http://www.ncbi.nlm.nih.gov/bioproject?db=bioproject&cmd=ShowDetailView&TermToSearch=30687) | [AM933172](http://www.ncbi.nlm.nih.gov/nuccore/AM933172) | Salmonella enterica subsp. enterica serovar Enteritidis str. P125109 complete genome. | Gammaproteobacteria | predicted phage protein | [CAR32965](http://www.ncbi.nlm.nih.gov/protein/CAR32965.1) | 100.0 |
|  | | | | | | | |
|  | | | | | | | |
| ------------------ | | | | | | | |
|  | | | | | | | |
|  | | | | | | | |
| **Input Sequence** | SM-Roxy-RH01221661_S26_L001_R1_001_(paired)_contig_40_33 # 29421 # 30350 # -1 # ID=40_33;partial=00;start_type=ATG;rbs_motif=AGGA;rbs_spacer=5-10bp;gc_cont=0.535 | | | | | | |
|  | PROJECT ID | ACCESSION ID | ORGANISMS | CLASS | PROTEIN FUNCTION | PROTEIN ID | %IDENTITY |
| **Matched Family** | [19467](http://www.ncbi.nlm.nih.gov/bioproject?db=bioproject&cmd=ShowDetailView&TermToSearch=19467) | [CP001144](http://www.ncbi.nlm.nih.gov/nuccore/CP001144) | Salmonella enterica subsp. enterica serovar Dublin str. C | Gammaproteobacteria | 2021853, complete genome. | [ACH75563](http://www.ncbi.nlm.nih.gov/protein/ACH75563.1) | 100.0 |
|  | | | | | | | |
|  | | | | | | | |
| ------------------ | | | | | | | |
|  | | | | | | | |
|  | | | | | | | |
| **Input Sequence** | SM-Roxy-RH01221661_S26_L001_R1_001_(paired)_contig_40_29 # 23666 # 24595 # -1 # ID=40_29;partial=00;start_type=ATG;rbs_motif=AGxAGG/AGGxGG;rbs_spacer=5-10bp;gc_cont=0.444 | | | | | | |
|  | PROJECT ID | ACCESSION ID | ORGANISMS | CLASS | PROTEIN FUNCTION | PROTEIN ID | %IDENTITY |
| **Matched Family** | [19467](http://www.ncbi.nlm.nih.gov/bioproject?db=bioproject&cmd=ShowDetailView&TermToSearch=19467) | [CP001144](http://www.ncbi.nlm.nih.gov/nuccore/CP001144) | Salmonella enterica subsp. enterica serovar Dublin str. C | Gammaproteobacteria | 2021853, complete genome. | [ACH77970](http://www.ncbi.nlm.nih.gov/protein/ACH77970.1) | 100.0 |
|  | | | | | | | |
|  | | | | | | | |
| ------------------ | | | | | | | |
|  | | | | | | | |
|  | | | | | | | |
| **Input Sequence** | SM-Roxy-RH01221661_S26_L001_R1_001_(paired)_contig_11_319 # 337628 # 338557 # -1 # ID=11_319;partial=00;start_type=ATG;rbs_motif=GGA/GAG/AGG;rbs_spacer=5-10bp;gc_cont=0.409 | | | | | | |
|  | PROJECT ID | ACCESSION ID | ORGANISMS | CLASS | PROTEIN FUNCTION | PROTEIN ID | %IDENTITY |
| **Matched Family** | [13086](http://www.ncbi.nlm.nih.gov/bioproject?db=bioproject&cmd=ShowDetailView&TermToSearch=13086) | [CP000026](http://www.ncbi.nlm.nih.gov/nuccore/CP000026) | Salmonella enterica subsp. enterica serovar Paratyphi A str. ATCC 9150, complete genome. | Gammaproteobacteria | AraC-family transcriptional regulator | [AAV78590](http://www.ncbi.nlm.nih.gov/protein/AAV78590.1) | 100.0 |
|  | | | | | | | |
|  | | | | | | | |
| ------------------ | | | | | | | |
|  | | | | | | | |
|  | | | | | | | |
| **Input Sequence** | SM-Roxy-RH01221661_S26_L001_R1_001_(paired)_contig_24_65 # 67524 # 68450 # 1 # ID=24_65;partial=00;start_type=ATG;rbs_motif=AGGAG;rbs_spacer=5-10bp;gc_cont=0.532 | | | | | | |
|  | PROJECT ID | ACCESSION ID | ORGANISMS | CLASS | PROTEIN FUNCTION | PROTEIN ID | %IDENTITY |
| **Matched Family** | [13086](http://www.ncbi.nlm.nih.gov/bioproject?db=bioproject&cmd=ShowDetailView&TermToSearch=13086) | [CP000026](http://www.ncbi.nlm.nih.gov/nuccore/CP000026) | Salmonella enterica subsp. enterica serovar Paratyphi A str. ATCC 9150, complete genome. | Gammaproteobacteria | hypothetical lysR-family transcriptional regulator | [AAV78095](http://www.ncbi.nlm.nih.gov/protein/AAV78095.1) | 100.0 |
|  | | | | | | | |
|  | | | | | | | |
| ------------------ | | | | | | | |
|  | | | | | | | |
|  | | | | | | | |
| **Input Sequence** | SM-Roxy-RH01221661_S26_L001_R1_001_(paired)_contig_14_53 # 58947 # 59873 # -1 # ID=14_53;partial=00;start_type=ATG;rbs_motif=AGGAG;rbs_spacer=5-10bp;gc_cont=0.561 | | | | | | |
|  | PROJECT ID | ACCESSION ID | ORGANISMS | CLASS | PROTEIN FUNCTION | PROTEIN ID | %IDENTITY |
| **Matched Family** | [18747](http://www.ncbi.nlm.nih.gov/bioproject?db=bioproject&cmd=ShowDetailView&TermToSearch=18747) | [CP001113](http://www.ncbi.nlm.nih.gov/nuccore/CP001113) | Salmonella enterica subsp. enterica serovar Newport str. SL254, complete genome. | Gammaproteobacteria | LysR-family transcriptional regulator | [ACF65410](http://www.ncbi.nlm.nih.gov/protein/ACF65410.1) | 100.0 |
|  | | | | | | | |
|  | | | | | | | |
| ------------------ | | | | | | | |
|  | | | | | | | |
|  | | | | | | | |
| **Input Sequence** | SM-Roxy-RH01221661_S26_L001_R1_001_(paired)_contig_54_28 # 26273 # 27199 # -1 # ID=54_28;partial=00;start_type=ATG;rbs_motif=None;rbs_spacer=None;gc_cont=0.430 | | | | | | |
|  | PROJECT ID | ACCESSION ID | ORGANISMS | CLASS | PROTEIN FUNCTION | PROTEIN ID | %IDENTITY |
| **Matched Family** | [19467](http://www.ncbi.nlm.nih.gov/bioproject?db=bioproject&cmd=ShowDetailView&TermToSearch=19467) | [CP001144](http://www.ncbi.nlm.nih.gov/nuccore/CP001144) | Salmonella enterica subsp. enterica serovar Dublin str. C | Gammaproteobacteria | 2021853, complete genome. | [ACH76836](http://www.ncbi.nlm.nih.gov/protein/ACH76836.1) | 100.0 |
|  | | | | | | | |
|  | | | | | | | |
| ------------------ | | | | | | | |
|  | | | | | | | |
|  | | | | | | | |
| **Input Sequence** | SM-Roxy-RH01221661_S26_L001_R1_001_(paired)_contig_11_344 # 359852 # 360775 # 1 # ID=11_344;partial=00;start_type=ATG;rbs_motif=AGGAG;rbs_spacer=5-10bp;gc_cont=0.588 | | | | | | |
|  | PROJECT ID | ACCESSION ID | ORGANISMS | CLASS | PROTEIN FUNCTION | PROTEIN ID | %IDENTITY |
| **Matched Family** | [30687](http://www.ncbi.nlm.nih.gov/bioproject?db=bioproject&cmd=ShowDetailView&TermToSearch=30687) | [AM933172](http://www.ncbi.nlm.nih.gov/nuccore/AM933172) | Salmonella enterica subsp. enterica serovar Enteritidis str. P125109 complete genome. | Gammaproteobacteria | formate hydrogenlyase subunit 4 | [CAR34270](http://www.ncbi.nlm.nih.gov/protein/CAR34270.1) | 100.0 |
|  | | | | | | | |
|  | | | | | | | |
| ------------------ | | | | | | | |
|  | | | | | | | |
|  | | | | | | | |
| **Input Sequence** | SM-Roxy-RH01221661_S26_L001_R1_001_(paired)_contig_3_25 # 23412 # 24245 # 1 # ID=3_25;partial=00;start_type=ATG;rbs_motif=AGGA;rbs_spacer=5-10bp;gc_cont=0.530 | | | | | | |
|  | PROJECT ID | ACCESSION ID | ORGANISMS | CLASS | PROTEIN FUNCTION | PROTEIN ID | %IDENTITY |
| **Matched Family** | [30687](http://www.ncbi.nlm.nih.gov/bioproject?db=bioproject&cmd=ShowDetailView&TermToSearch=30687) | [AM933172](http://www.ncbi.nlm.nih.gov/nuccore/AM933172) | Salmonella enterica subsp. enterica serovar Enteritidis str. P125109 complete genome. | Gammaproteobacteria | xanthosine phosphorylase | [CAR33988](http://www.ncbi.nlm.nih.gov/protein/CAR33988.1) | 100.0 |
|  | | | | | | | |
|  | | | | | | | |
| ------------------ | | | | | | | |
|  | | | | | | | |
|  | | | | | | | |
| **Input Sequence** | SM-Roxy-RH01221661_S26_L001_R1_001_(paired)_contig_41_30 # 37034 # 37858 # 1 # ID=41_30;partial=00;start_type=ATG;rbs_motif=AGGAG;rbs_spacer=5-10bp;gc_cont=0.522 | | | | | | |
|  | PROJECT ID | ACCESSION ID | ORGANISMS | CLASS | PROTEIN FUNCTION | PROTEIN ID | %IDENTITY |
| **Matched Family** | [18747](http://www.ncbi.nlm.nih.gov/bioproject?db=bioproject&cmd=ShowDetailView&TermToSearch=18747) | [CP001113](http://www.ncbi.nlm.nih.gov/nuccore/CP001113) | Salmonella enterica subsp. enterica serovar Newport str. SL254, complete genome. | Gammaproteobacteria | protein bax | [ACF62390](http://www.ncbi.nlm.nih.gov/protein/ACF62390.1) | 100.0 |
|  | | | | | | | |
|  | | | | | | | |
| ------------------ | | | | | | | |
|  | | | | | | | |
|  | | | | | | | |
| **Input Sequence** | SM-Roxy-RH01221661_S26_L001_R1_001_(paired)_contig_82_1 # 145 # 486 # 1 # ID=82_1;partial=00;start_type=ATG;rbs_motif=GGA/GAG/AGG;rbs_spacer=11-12bp;gc_cont=0.526 | | | | | | |
|  | PROJECT ID | ACCESSION ID | ORGANISMS | CLASS | PROTEIN FUNCTION | PROTEIN ID | %IDENTITY |
| **Matched Family** | [13086](http://www.ncbi.nlm.nih.gov/bioproject?db=bioproject&cmd=ShowDetailView&TermToSearch=13086) | [CP000026](http://www.ncbi.nlm.nih.gov/nuccore/CP000026) | Salmonella enterica subsp. enterica serovar Paratyphi A str. ATCC 9150, complete genome. | Gammaproteobacteria | putative carbohydrate kinase | [AAV79437](http://www.ncbi.nlm.nih.gov/protein/AAV79437.1) | 100.0 |
|  | | | | | | | |
|  | | | | | | | |
| ------------------ | | | | | | | |
|  | | | | | | | |
|  | | | | | | | |
| **Input Sequence** | SM-Roxy-RH01221661_S26_L001_R1_001_(paired)_contig_23_22 # 21734 # 22654 # -1 # ID=23_22;partial=00;start_type=ATG;rbs_motif=AGGAG;rbs_spacer=5-10bp;gc_cont=0.496 | | | | | | |
|  | PROJECT ID | ACCESSION ID | ORGANISMS | CLASS | PROTEIN FUNCTION | PROTEIN ID | %IDENTITY |
| **Matched Family** | [19467](http://www.ncbi.nlm.nih.gov/bioproject?db=bioproject&cmd=ShowDetailView&TermToSearch=19467) | [CP001144](http://www.ncbi.nlm.nih.gov/nuccore/CP001144) | Salmonella enterica subsp. enterica serovar Dublin str. C | Gammaproteobacteria | 2021853, complete genome. | [ACH77784](http://www.ncbi.nlm.nih.gov/protein/ACH77784.1) | 100.0 |
|  | | | | | | | |
|  | | | | | | | |
| ------------------ | | | | | | | |
|  | | | | | | | |
|  | | | | | | | |
| **Input Sequence** | SM-Roxy-RH01221661_S26_L001_R1_001_(paired)_contig_21_249 # 249295 # 250212 # -1 # ID=21_249;partial=00;start_type=ATG;rbs_motif=GGAG/GAGG;rbs_spacer=5-10bp;gc_cont=0.551 | | | | | | |
|  | PROJECT ID | ACCESSION ID | ORGANISMS | CLASS | PROTEIN FUNCTION | PROTEIN ID | %IDENTITY |
| **Matched Family** | [18747](http://www.ncbi.nlm.nih.gov/bioproject?db=bioproject&cmd=ShowDetailView&TermToSearch=18747) | [CP001113](http://www.ncbi.nlm.nih.gov/nuccore/CP001113) | Salmonella enterica subsp. enterica serovar Newport str. SL254, complete genome. | Gammaproteobacteria | amine ABC transporter, periplasmic amine-binding protein | [ACF61236](http://www.ncbi.nlm.nih.gov/protein/ACF61236.1) | 100.0 |
|  | | | | | | | |
|  | | | | | | | |
| ------------------ | | | | | | | |
|  | | | | | | | |
|  | | | | | | | |
| **Input Sequence** | SM-Roxy-RH01221661_S26_L001_R1_001_(paired)_contig_54_30 # 28533 # 29450 # 1 # ID=54_30;partial=00;start_type=ATG;rbs_motif=AGGA;rbs_spacer=5-10bp;gc_cont=0.492 | | | | | | |
|  | PROJECT ID | ACCESSION ID | ORGANISMS | CLASS | PROTEIN FUNCTION | PROTEIN ID | %IDENTITY |
| **Matched Family** | [19467](http://www.ncbi.nlm.nih.gov/bioproject?db=bioproject&cmd=ShowDetailView&TermToSearch=19467) | [CP001144](http://www.ncbi.nlm.nih.gov/nuccore/CP001144) | Salmonella enterica subsp. enterica serovar Dublin str. C | Gammaproteobacteria | 2021853, complete genome. | [ACH77751](http://www.ncbi.nlm.nih.gov/protein/ACH77751.1) | 100.0 |
|  | | | | | | | |
|  | | | | | | | |
| ------------------ | | | | | | | |
|  | | | | | | | |
|  | | | | | | | |
| **Input Sequence** | SM-Roxy-RH01221661_S26_L001_R1_001_(paired)_contig_21_152 # 136596 # 137510 # -1 # ID=21_152;partial=00;start_type=ATG;rbs_motif=GGA/GAG/AGG;rbs_spacer=5-10bp;gc_cont=0.527 | | | | | | |
|  | PROJECT ID | ACCESSION ID | ORGANISMS | CLASS | PROTEIN FUNCTION | PROTEIN ID | %IDENTITY |
| **Matched Family** | [30687](http://www.ncbi.nlm.nih.gov/bioproject?db=bioproject&cmd=ShowDetailView&TermToSearch=30687) | [AM933172](http://www.ncbi.nlm.nih.gov/nuccore/AM933172) | Salmonella enterica subsp. enterica serovar Enteritidis str. P125109 complete genome. | Gammaproteobacteria | putative transcriptional regulator | [CAR33647](http://www.ncbi.nlm.nih.gov/protein/CAR33647.1) | 100.0 |
|  | | | | | | | |
|  | | | | | | | |
| ------------------ | | | | | | | |
|  | | | | | | | |
|  | | | | | | | |
| **Input Sequence** | SM-Roxy-RH01221661_S26_L001_R1_001_(paired)_contig_21_119 # 103502 # 104413 # -1 # ID=21_119;partial=00;start_type=ATG;rbs_motif=GGAG/GAGG;rbs_spacer=5-10bp;gc_cont=0.459 | | | | | | |
|  | PROJECT ID | ACCESSION ID | ORGANISMS | CLASS | PROTEIN FUNCTION | PROTEIN ID | %IDENTITY |
| **Matched Family** | [18747](http://www.ncbi.nlm.nih.gov/bioproject?db=bioproject&cmd=ShowDetailView&TermToSearch=18747) | [CP001113](http://www.ncbi.nlm.nih.gov/nuccore/CP001113) | Salmonella enterica subsp. enterica serovar Newport str. SL254, complete genome. | Gammaproteobacteria | regulatory protein PocR | [ACF62922](http://www.ncbi.nlm.nih.gov/protein/ACF62922.1) | 100.0 |
|  | | | | | | | |
|  | | | | | | | |
| ------------------ | | | | | | | |
|  | | | | | | | |
|  | | | | | | | |
| **Input Sequence** | SM-Roxy-RH01221661_S26_L001_R1_001_(paired)_contig_21_199 # 188270 # 189181 # -1 # ID=21_199;partial=00;start_type=ATG;rbs_motif=GGA/GAG/AGG;rbs_spacer=5-10bp;gc_cont=0.518 | | | | | | |
|  | PROJECT ID | ACCESSION ID | ORGANISMS | CLASS | PROTEIN FUNCTION | PROTEIN ID | %IDENTITY |
| **Matched Family** | [19459](http://www.ncbi.nlm.nih.gov/bioproject?db=bioproject&cmd=ShowDetailView&TermToSearch=19459) | [CP001127](http://www.ncbi.nlm.nih.gov/nuccore/CP001127) | Salmonella enterica subsp. enterica serovar Schwarzengrund str. CVM19633, complete genome. | Gammaproteobacteria | putative colanic acid biosynthesis glycosyl transferase WcaA | [ACF92253](http://www.ncbi.nlm.nih.gov/protein/ACF92253.1) | 100.0 |
|  | | | | | | | |
|  | | | | | | | |
| ------------------ | | | | | | | |
|  | | | | | | | |
|  | | | | | | | |
| **Input Sequence** | SM-Roxy-RH01221661_S26_L001_R1_001_(paired)_contig_11_303 # 321146 # 322057 # 1 # ID=11_303;partial=00;start_type=ATG;rbs_motif=AGGAGG;rbs_spacer=5-10bp;gc_cont=0.526 | | | | | | |
|  | PROJECT ID | ACCESSION ID | ORGANISMS | CLASS | PROTEIN FUNCTION | PROTEIN ID | %IDENTITY |
| **Matched Family** | [18747](http://www.ncbi.nlm.nih.gov/bioproject?db=bioproject&cmd=ShowDetailView&TermToSearch=18747) | [CP001113](http://www.ncbi.nlm.nih.gov/nuccore/CP001113) | Salmonella enterica subsp. enterica serovar Newport str. SL254, complete genome. | Gammaproteobacteria | type III secretion apparatus protein, YscQ/HrcQ family | [ACF61111](http://www.ncbi.nlm.nih.gov/protein/ACF61111.1) | 100.0 |
|  | | | | | | | |
|  | | | | | | | |
| ------------------ | | | | | | | |
|  | | | | | | | |
|  | | | | | | | |
| **Input Sequence** | SM-Roxy-RH01221661_S26_L001_R1_001_(paired)_contig_23_5 # 3465 # 4376 # 1 # ID=23_5;partial=00;start_type=ATG;rbs_motif=AGxAGG/AGGxGG;rbs_spacer=3-4bp;gc_cont=0.368 | | | | | | |
|  | PROJECT ID | ACCESSION ID | ORGANISMS | CLASS | PROTEIN FUNCTION | PROTEIN ID | %IDENTITY |
| **Matched Family** | [30687](http://www.ncbi.nlm.nih.gov/bioproject?db=bioproject&cmd=ShowDetailView&TermToSearch=30687) | [AM933172](http://www.ncbi.nlm.nih.gov/nuccore/AM933172) | Salmonella enterica subsp. enterica serovar Enteritidis str. P125109 complete genome. | Gammaproteobacteria | hypothetical protein | [CAR35817](http://www.ncbi.nlm.nih.gov/protein/CAR35817.1) | 100.0 |
|  | | | | | | | |
|  | | | | | | | |
| ------------------ | | | | | | | |
|  | | | | | | | |
|  | | | | | | | |
| **Input Sequence** | SM-Roxy-RH01221661_S26_L001_R1_001_(paired)_contig_21_263 # 266103 # 267011 # 1 # ID=21_263;partial=00;start_type=ATG;rbs_motif=GGAGG;rbs_spacer=5-10bp;gc_cont=0.548 | | | | | | |
|  | PROJECT ID | ACCESSION ID | ORGANISMS | CLASS | PROTEIN FUNCTION | PROTEIN ID | %IDENTITY |
| **Matched Family** | [20993](http://www.ncbi.nlm.nih.gov/bioproject?db=bioproject&cmd=ShowDetailView&TermToSearch=20993) | [CP000857](http://www.ncbi.nlm.nih.gov/nuccore/CP000857) | Salmonella enterica subsp. enterica serovar Paratyphi C strain RKS4594, complete genome. | Gammaproteobacteria | putative transcriptional regulator | [ACN45676](http://www.ncbi.nlm.nih.gov/protein/ACN45676.1) | 100.0 |
|  | | | | | | | |
|  | | | | | | | |
| ------------------ | | | | | | | |
|  | | | | | | | |
|  | | | | | | | |
| **Input Sequence** | SM-Roxy-RH01221661_S26_L001_R1_001_(paired)_contig_11_329 # 345629 # 346495 # 1 # ID=11_329;partial=00;start_type=ATG;rbs_motif=GGAGG;rbs_spacer=5-10bp;gc_cont=0.419 | | | | | | |
|  | PROJECT ID | ACCESSION ID | ORGANISMS | CLASS | PROTEIN FUNCTION | PROTEIN ID | %IDENTITY |
| **Matched Family** | [30687](http://www.ncbi.nlm.nih.gov/bioproject?db=bioproject&cmd=ShowDetailView&TermToSearch=30687) | [AM933172](http://www.ncbi.nlm.nih.gov/nuccore/AM933172) | Salmonella enterica subsp. enterica serovar Enteritidis str. P125109 complete genome. | Gammaproteobacteria | pathogenicity island membrane protein | [CAR34285](http://www.ncbi.nlm.nih.gov/protein/CAR34285.1) | 100.0 |
|  | | | | | | | |
|  | | | | | | | |
| ------------------ | | | | | | | |
|  | | | | | | | |
|  | | | | | | | |
| **Input Sequence** | SM-Roxy-RH01221661_S26_L001_R1_001_(paired)_contig_23_12 # 10088 # 10996 # -1 # ID=23_12;partial=00;start_type=ATG;rbs_motif=AGGA;rbs_spacer=5-10bp;gc_cont=0.524 | | | | | | |
|  | PROJECT ID | ACCESSION ID | ORGANISMS | CLASS | PROTEIN FUNCTION | PROTEIN ID | %IDENTITY |
| **Matched Family** | [13086](http://www.ncbi.nlm.nih.gov/bioproject?db=bioproject&cmd=ShowDetailView&TermToSearch=13086) | [CP000026](http://www.ncbi.nlm.nih.gov/nuccore/CP000026) | Salmonella enterica subsp. enterica serovar Paratyphi A str. ATCC 9150, complete genome. | Gammaproteobacteria | probable transcriptional activator | [AAV80058](http://www.ncbi.nlm.nih.gov/protein/AAV80058.1) | 100.0 |
|  | | | | | | | |
|  | | | | | | | |
| ------------------ | | | | | | | |
|  | | | | | | | |
|  | | | | | | | |
| **Input Sequence** | SM-Roxy-RH01221661_S26_L001_R1_001_(paired)_contig_38_9 # 8909 # 9817 # -1 # ID=38_9;partial=00;start_type=ATG;rbs_motif=GGA/GAG/AGG;rbs_spacer=5-10bp;gc_cont=0.472 | | | | | | |
|  | PROJECT ID | ACCESSION ID | ORGANISMS | CLASS | PROTEIN FUNCTION | PROTEIN ID | %IDENTITY |
| **Matched Family** | [30687](http://www.ncbi.nlm.nih.gov/bioproject?db=bioproject&cmd=ShowDetailView&TermToSearch=30687) | [AM933172](http://www.ncbi.nlm.nih.gov/nuccore/AM933172) | Salmonella enterica subsp. enterica serovar Enteritidis str. P125109 complete genome. | Gammaproteobacteria | putative membrane protein | [CAR32308](http://www.ncbi.nlm.nih.gov/protein/CAR32308.1) | 100.0 |
|  | | | | | | | |
|  | | | | | | | |
| ------------------ | | | | | | | |
|  | | | | | | | |
|  | | | | | | | |
| **Input Sequence** | SM-Roxy-RH01221661_S26_L001_R1_001_(paired)_contig_19_29 # 27683 # 28585 # 1 # ID=19_29;partial=00;start_type=ATG;rbs_motif=GGA/GAG/AGG;rbs_spacer=5-10bp;gc_cont=0.391 | | | | | | |
|  | PROJECT ID | ACCESSION ID | ORGANISMS | CLASS | PROTEIN FUNCTION | PROTEIN ID | %IDENTITY |
| **Matched Family** | [20045](http://www.ncbi.nlm.nih.gov/bioproject?db=bioproject&cmd=ShowDetailView&TermToSearch=20045) | [CP001120](http://www.ncbi.nlm.nih.gov/nuccore/CP001120) | Salmonella enterica subsp. enterica serovar Heidelberg str. SL476, complete genome. | Gammaproteobacteria | transcriptional regulator, LysR family | [ACF69019](http://www.ncbi.nlm.nih.gov/protein/ACF69019.1) | 100.0 |
|  | | | | | | | |
|  | | | | | | | |
| ------------------ | | | | | | | |
|  | | | | | | | |
|  | | | | | | | |
| **Input Sequence** | SM-Roxy-RH01221661_S26_L001_R1_001_(paired)_contig_5_32 # 33558 # 34454 # 1 # ID=5_32;partial=00;start_type=ATG;rbs_motif=GGAG/GAGG;rbs_spacer=5-10bp;gc_cont=0.570 | | | | | | |
|  | PROJECT ID | ACCESSION ID | ORGANISMS | CLASS | PROTEIN FUNCTION | PROTEIN ID | %IDENTITY |
| **Matched Family** | [30687](http://www.ncbi.nlm.nih.gov/bioproject?db=bioproject&cmd=ShowDetailView&TermToSearch=30687) | [AM933172](http://www.ncbi.nlm.nih.gov/nuccore/AM933172) | Salmonella enterica subsp. enterica serovar Enteritidis str. P125109 complete genome. | Gammaproteobacteria | putative sugar kinase | [CAR35386](http://www.ncbi.nlm.nih.gov/protein/CAR35386.1) | 100.0 |
|  | | | | | | | |
|  | | | | | | | |
| ------------------ | | | | | | | |
|  | | | | | | | |
|  | | | | | | | |
| **Input Sequence** | SM-Roxy-RH01221661_S26_L001_R1_001_(paired)_contig_47_45 # 41354 # 42256 # -1 # ID=47_45;partial=00;start_type=ATG;rbs_motif=GGA/GAG/AGG;rbs_spacer=5-10bp;gc_cont=0.530 | | | | | | |
|  | PROJECT ID | ACCESSION ID | ORGANISMS | CLASS | PROTEIN FUNCTION | PROTEIN ID | %IDENTITY |
| **Matched Family** | [20063](http://www.ncbi.nlm.nih.gov/bioproject?db=bioproject&cmd=ShowDetailView&TermToSearch=20063) | [CP001138](http://www.ncbi.nlm.nih.gov/nuccore/CP001138) | Salmonella enterica subsp. enterica serovar Agona str. SL483, complete genome. | Gammaproteobacteria | transcriptional regulator, LysR family | [ACH49739](http://www.ncbi.nlm.nih.gov/protein/ACH49739.1) | 100.0 |
|  | | | | | | | |
|  | | | | | | | |
| ------------------ | | | | | | | |
|  | | | | | | | |
|  | | | | | | | |
| **Input Sequence** | SM-Roxy-RH01221661_S26_L001_R1_001_(paired)_contig_38_77 # 80617 # 81516 # -1 # ID=38_77;partial=00;start_type=ATG;rbs_motif=GGAG/GAGG;rbs_spacer=5-10bp;gc_cont=0.556 | | | | | | |
|  | PROJECT ID | ACCESSION ID | ORGANISMS | CLASS | PROTEIN FUNCTION | PROTEIN ID | %IDENTITY |
| **Matched Family** | [18747](http://www.ncbi.nlm.nih.gov/bioproject?db=bioproject&cmd=ShowDetailView&TermToSearch=18747) | [CP001113](http://www.ncbi.nlm.nih.gov/nuccore/CP001113) | Salmonella enterica subsp. enterica serovar Newport str. SL254, complete genome. | Gammaproteobacteria | glycyl-radical enzyme activating protein family | [ACF64546](http://www.ncbi.nlm.nih.gov/protein/ACF64546.1) | 100.0 |
|  | | | | | | | |
|  | | | | | | | |
| ------------------ | | | | | | | |
|  | | | | | | | |
|  | | | | | | | |
| **Input Sequence** | SM-Roxy-RH01221661_S26_L001_R1_001_(paired)_contig_54_12 # 11263 # 12162 # 1 # ID=54_12;partial=00;start_type=ATG;rbs_motif=GGA/GAG/AGG;rbs_spacer=5-10bp;gc_cont=0.513 | | | | | | |
|  | PROJECT ID | ACCESSION ID | ORGANISMS | CLASS | PROTEIN FUNCTION | PROTEIN ID | %IDENTITY |
| **Matched Family** | [30687](http://www.ncbi.nlm.nih.gov/bioproject?db=bioproject&cmd=ShowDetailView&TermToSearch=30687) | [AM933172](http://www.ncbi.nlm.nih.gov/nuccore/AM933172) | Salmonella enterica subsp. enterica serovar Enteritidis str. P125109 complete genome. | Gammaproteobacteria | LysR-family transcriptional regulator | [CAR34153](http://www.ncbi.nlm.nih.gov/protein/CAR34153.1) | 100.0 |
|  | | | | | | | |
|  | | | | | | | |
| ------------------ | | | | | | | |
|  | | | | | | | |
|  | | | | | | | |
| **Input Sequence** | SM-Roxy-RH01221661_S26_L001_R1_001_(paired)_contig_5_31 # 32497 # 33393 # -1 # ID=5_31;partial=00;start_type=ATG;rbs_motif=AGGAG;rbs_spacer=5-10bp;gc_cont=0.560 | | | | | | |
|  | PROJECT ID | ACCESSION ID | ORGANISMS | CLASS | PROTEIN FUNCTION | PROTEIN ID | %IDENTITY |
| **Matched Family** | [30687](http://www.ncbi.nlm.nih.gov/bioproject?db=bioproject&cmd=ShowDetailView&TermToSearch=30687) | [AM933172](http://www.ncbi.nlm.nih.gov/nuccore/AM933172) | Salmonella enterica subsp. enterica serovar Enteritidis str. P125109 complete genome. | Gammaproteobacteria | putative oxidoreductase | [CAR35385](http://www.ncbi.nlm.nih.gov/protein/CAR35385.1) | 100.0 |
|  | | | | | | | |
|  | | | | | | | |
| ------------------ | | | | | | | |
|  | | | | | | | |
|  | | | | | | | |
| **Input Sequence** | SM-Roxy-RH01221661_S26_L001_R1_001_(paired)_contig_25_45 # 35235 # 36095 # -1 # ID=25_45;partial=00;start_type=ATG;rbs_motif=None;rbs_spacer=None;gc_cont=0.501 | | | | | | |
|  | PROJECT ID | ACCESSION ID | ORGANISMS | CLASS | PROTEIN FUNCTION | PROTEIN ID | %IDENTITY |
| **Matched Family** | [13086](http://www.ncbi.nlm.nih.gov/bioproject?db=bioproject&cmd=ShowDetailView&TermToSearch=13086) | [CP000026](http://www.ncbi.nlm.nih.gov/nuccore/CP000026) | Salmonella enterica subsp. enterica serovar Paratyphi A str. ATCC 9150, complete genome. | Gammaproteobacteria | putative HlyD-family protein | [AAV77352](http://www.ncbi.nlm.nih.gov/protein/AAV77352.1) | 100.0 |
|  | | | | | | | |
|  | | | | | | | |
| ------------------ | | | | | | | |
|  | | | | | | | |
|  | | | | | | | |
| **Input Sequence** | SM-Roxy-RH01221661_S26_L001_R1_001_(paired)_contig_20_28 # 33017 # 33913 # -1 # ID=20_28;partial=00;start_type=ATG;rbs_motif=GGA/GAG/AGG;rbs_spacer=5-10bp;gc_cont=0.532 | | | | | | |
|  | PROJECT ID | ACCESSION ID | ORGANISMS | CLASS | PROTEIN FUNCTION | PROTEIN ID | %IDENTITY |
| **Matched Family** | [241](http://www.ncbi.nlm.nih.gov/bioproject?db=bioproject&cmd=ShowDetailView&TermToSearch=241) | [AE006468](http://www.ncbi.nlm.nih.gov/nuccore/AE006468) | Salmonella enterica subsp. enterica serovar Typhimurium str. LT2, complete genome. | Gammaproteobacteria | putative LysR family transcriptional regulator | [AAL22693](http://www.ncbi.nlm.nih.gov/protein/AAL22693.1) | 100.0 |
|  | | | | | | | |
|  | | | | | | | |
| ------------------ | | | | | | | |
|  | | | | | | | |
|  | | | | | | | |
| **Input Sequence** | SM-Roxy-RH01221661_S26_L001_R1_001_(paired)_contig_38_72 # 73916 # 74812 # 1 # ID=38_72;partial=00;start_type=ATG;rbs_motif=AGGA;rbs_spacer=5-10bp;gc_cont=0.421 | | | | | | |
|  | PROJECT ID | ACCESSION ID | ORGANISMS | CLASS | PROTEIN FUNCTION | PROTEIN ID | %IDENTITY |
| **Matched Family** | [30687](http://www.ncbi.nlm.nih.gov/bioproject?db=bioproject&cmd=ShowDetailView&TermToSearch=30687) | [AM933172](http://www.ncbi.nlm.nih.gov/nuccore/AM933172) | Salmonella enterica subsp. enterica serovar Enteritidis str. P125109 complete genome. | Gammaproteobacteria | conserved hypothetical protein | [CAR32369](http://www.ncbi.nlm.nih.gov/protein/CAR32369.1) | 100.0 |
|  | | | | | | | |
|  | | | | | | | |
| ------------------ | | | | | | | |
|  | | | | | | | |
|  | | | | | | | |
| **Input Sequence** | SM-Roxy-RH01221661_S26_L001_R1_001_(paired)_contig_11_138 # 141273 # 142166 # 1 # ID=11_138;partial=00;start_type=ATG;rbs_motif=GGAGG;rbs_spacer=5-10bp;gc_cont=0.435 | | | | | | |
|  | PROJECT ID | ACCESSION ID | ORGANISMS | CLASS | PROTEIN FUNCTION | PROTEIN ID | %IDENTITY |
| **Matched Family** | [18747](http://www.ncbi.nlm.nih.gov/bioproject?db=bioproject&cmd=ShowDetailView&TermToSearch=18747) | [CP001113](http://www.ncbi.nlm.nih.gov/nuccore/CP001113) | Salmonella enterica subsp. enterica serovar Newport str. SL254, complete genome. | Gammaproteobacteria | putative outer membrane protein | [ACF62703](http://www.ncbi.nlm.nih.gov/protein/ACF62703.1) | 100.0 |
|  | | | | | | | |
|  | | | | | | | |
| ------------------ | | | | | | | |
|  | | | | | | | |
|  | | | | | | | |
| **Input Sequence** | SM-Roxy-RH01221661_S26_L001_R1_001_(paired)_contig_30_13 # 11173 # 12066 # 1 # ID=30_13;partial=00;start_type=ATG;rbs_motif=GGAG/GAGG;rbs_spacer=5-10bp;gc_cont=0.538 | | | | | | |
|  | PROJECT ID | ACCESSION ID | ORGANISMS | CLASS | PROTEIN FUNCTION | PROTEIN ID | %IDENTITY |
| **Matched Family** | [18747](http://www.ncbi.nlm.nih.gov/bioproject?db=bioproject&cmd=ShowDetailView&TermToSearch=18747) | [CP001113](http://www.ncbi.nlm.nih.gov/nuccore/CP001113) | Salmonella enterica subsp. enterica serovar Newport str. SL254, complete genome. | Gammaproteobacteria | N-acetylneuraminate lyase | [ACF64250](http://www.ncbi.nlm.nih.gov/protein/ACF64250.1) | 100.0 |
|  | | | | | | | |
|  | | | | | | | |
| ------------------ | | | | | | | |
|  | | | | | | | |
|  | | | | | | | |
| **Input Sequence** | SM-Roxy-RH01221661_S26_L001_R1_001_(paired)_contig_25_179 # 179516 # 180409 # 1 # ID=25_179;partial=00;start_type=ATG;rbs_motif=AGGAG;rbs_spacer=5-10bp;gc_cont=0.531 | | | | | | |
|  | PROJECT ID | ACCESSION ID | ORGANISMS | CLASS | PROTEIN FUNCTION | PROTEIN ID | %IDENTITY |
| **Matched Family** | [18747](http://www.ncbi.nlm.nih.gov/bioproject?db=bioproject&cmd=ShowDetailView&TermToSearch=18747) | [CP001113](http://www.ncbi.nlm.nih.gov/nuccore/CP001113) | Salmonella enterica subsp. enterica serovar Newport str. SL254, complete genome. | Gammaproteobacteria | phenazine biosynthesis PhzC/PhzF protein | [ACF63331](http://www.ncbi.nlm.nih.gov/protein/ACF63331.1) | 100.0 |
|  | | | | | | | |
|  | | | | | | | |
| ------------------ | | | | | | | |
|  | | | | | | | |
|  | | | | | | | |
| **Input Sequence** | SM-Roxy-RH01221661_S26_L001_R1_001_(paired)_contig_42_115 # 121214 # 122107 # -1 # ID=42_115;partial=00;start_type=ATG;rbs_motif=GGxGG;rbs_spacer=5-10bp;gc_cont=0.554 | | | | | | |
|  | PROJECT ID | ACCESSION ID | ORGANISMS | CLASS | PROTEIN FUNCTION | PROTEIN ID | %IDENTITY |
| **Matched Family** | [9618](http://www.ncbi.nlm.nih.gov/bioproject?db=bioproject&cmd=ShowDetailView&TermToSearch=9618) | [AE017220](http://www.ncbi.nlm.nih.gov/nuccore/AE017220) | Salmonella enterica subsp. enterica serovar Choleraesuis str. SC-B67, complete genome. | Gammaproteobacteria | mukF protein (killing factor KicB) | [AAX64853](http://www.ncbi.nlm.nih.gov/protein/AAX64853.1) | 100.0 |
|  | | | | | | | |
|  | | | | | | | |
| ------------------ | | | | | | | |
|  | | | | | | | |
|  | | | | | | | |
| **Input Sequence** | SM-Roxy-RH01221661_S26_L001_R1_001_(paired)_contig_38_94 # 98860 # 99750 # -1 # ID=38_94;partial=00;start_type=ATG;rbs_motif=GGA/GAG/AGG;rbs_spacer=5-10bp;gc_cont=0.403 | | | | | | |
|  | PROJECT ID | ACCESSION ID | ORGANISMS | CLASS | PROTEIN FUNCTION | PROTEIN ID | %IDENTITY |
| **Matched Family** | [18747](http://www.ncbi.nlm.nih.gov/bioproject?db=bioproject&cmd=ShowDetailView&TermToSearch=18747) | [CP001113](http://www.ncbi.nlm.nih.gov/nuccore/CP001113) | Salmonella enterica subsp. enterica serovar Newport str. SL254, complete genome. | Gammaproteobacteria | transcriptional regulator, LysR family | [ACF63983](http://www.ncbi.nlm.nih.gov/protein/ACF63983.1) | 100.0 |
|  | | | | | | | |
|  | | | | | | | |
| ------------------ | | | | | | | |
|  | | | | | | | |
|  | | | | | | | |
| **Input Sequence** | SM-Roxy-RH01221661_S26_L001_R1_001_(paired)_contig_14_106 # 116949 # 117839 # 1 # ID=14_106;partial=00;start_type=ATG;rbs_motif=GGA/GAG/AGG;rbs_spacer=5-10bp;gc_cont=0.349 | | | | | | |
|  | PROJECT ID | ACCESSION ID | ORGANISMS | CLASS | PROTEIN FUNCTION | PROTEIN ID | %IDENTITY |
| **Matched Family** | [19467](http://www.ncbi.nlm.nih.gov/bioproject?db=bioproject&cmd=ShowDetailView&TermToSearch=19467) | [CP001144](http://www.ncbi.nlm.nih.gov/nuccore/CP001144) | Salmonella enterica subsp. enterica serovar Dublin str. C | Gammaproteobacteria | 2021853, complete genome. | [ACH75979](http://www.ncbi.nlm.nih.gov/protein/ACH75979.1) | 100.0 |
|  | | | | | | | |
|  | | | | | | | |
| ------------------ | | | | | | | |
|  | | | | | | | |
|  | | | | | | | |
| **Input Sequence** | SM-Roxy-RH01221661_S26_L001_R1_001_(paired)_contig_21_280 # 283487 # 284374 # 1 # ID=21_280;partial=00;start_type=ATG;rbs_motif=GGAG/GAGG;rbs_spacer=5-10bp;gc_cont=0.447 | | | | | | |
|  | PROJECT ID | ACCESSION ID | ORGANISMS | CLASS | PROTEIN FUNCTION | PROTEIN ID | %IDENTITY |
| **Matched Family** | [20993](http://www.ncbi.nlm.nih.gov/bioproject?db=bioproject&cmd=ShowDetailView&TermToSearch=20993) | [CP000857](http://www.ncbi.nlm.nih.gov/nuccore/CP000857) | Salmonella enterica subsp. enterica serovar Paratyphi C strain RKS4594, complete genome. | Gammaproteobacteria | putative hydrolase | [ACN45662](http://www.ncbi.nlm.nih.gov/protein/ACN45662.1) | 100.0 |
|  | | | | | | | |
|  | | | | | | | |
| ------------------ | | | | | | | |
|  | | | | | | | |
|  | | | | | | | |
| **Input Sequence** | SM-Roxy-RH01221661_S26_L001_R1_001_(paired)_contig_11_327 # 343399 # 344286 # 1 # ID=11_327;partial=00;start_type=ATG;rbs_motif=AGGA;rbs_spacer=5-10bp;gc_cont=0.372 | | | | | | |
|  | PROJECT ID | ACCESSION ID | ORGANISMS | CLASS | PROTEIN FUNCTION | PROTEIN ID | %IDENTITY |
| **Matched Family** | [13086](http://www.ncbi.nlm.nih.gov/bioproject?db=bioproject&cmd=ShowDetailView&TermToSearch=13086) | [CP000026](http://www.ncbi.nlm.nih.gov/nuccore/CP000026) | Salmonella enterica subsp. enterica serovar Paratyphi A str. ATCC 9150, complete genome. | Gammaproteobacteria | possible AraC-family transcriptional regulator | [AAV78582](http://www.ncbi.nlm.nih.gov/protein/AAV78582.1) | 100.0 |
|  | | | | | | | |
|  | | | | | | | |
| ------------------ | | | | | | | |
|  | | | | | | | |
|  | | | | | | | |
| **Input Sequence** | SM-Roxy-RH01221661_S26_L001_R1_001_(paired)_contig_25_133 # 124602 # 125489 # -1 # ID=25_133;partial=00;start_type=GTG;rbs_motif=GGA/GAG/AGG;rbs_spacer=5-10bp;gc_cont=0.544 | | | | | | |
|  | PROJECT ID | ACCESSION ID | ORGANISMS | CLASS | PROTEIN FUNCTION | PROTEIN ID | %IDENTITY |
| **Matched Family** | [20993](http://www.ncbi.nlm.nih.gov/bioproject?db=bioproject&cmd=ShowDetailView&TermToSearch=20993) | [CP000857](http://www.ncbi.nlm.nih.gov/nuccore/CP000857) | Salmonella enterica subsp. enterica serovar Paratyphi C strain RKS4594, complete genome. | Gammaproteobacteria | putative dehydrogenase protein | [ACN46320](http://www.ncbi.nlm.nih.gov/protein/ACN46320.1) | 100.0 |
|  | | | | | | | |
|  | | | | | | | |
| ------------------ | | | | | | | |
|  | | | | | | | |
|  | | | | | | | |
| **Input Sequence** | SM-Roxy-RH01221661_S26_L001_R1_001_(paired)_contig_11_34 # 33520 # 34404 # -1 # ID=11_34;partial=00;start_type=ATG;rbs_motif=GGAG/GAGG;rbs_spacer=5-10bp;gc_cont=0.566 | | | | | | |
|  | PROJECT ID | ACCESSION ID | ORGANISMS | CLASS | PROTEIN FUNCTION | PROTEIN ID | %IDENTITY |
| **Matched Family** | [18747](http://www.ncbi.nlm.nih.gov/bioproject?db=bioproject&cmd=ShowDetailView&TermToSearch=18747) | [CP001113](http://www.ncbi.nlm.nih.gov/nuccore/CP001113) | Salmonella enterica subsp. enterica serovar Newport str. SL254, complete genome. | Gammaproteobacteria | general stress protein 39 | [ACF64666](http://www.ncbi.nlm.nih.gov/protein/ACF64666.1) | 100.0 |
|  | | | | | | | |
|  | | | | | | | |
| ------------------ | | | | | | | |
|  | | | | | | | |
|  | | | | | | | |
| **Input Sequence** | SM-Roxy-RH01221661_S26_L001_R1_001_(paired)_contig_49_20 # 22447 # 23331 # 1 # ID=49_20;partial=00;start_type=ATG;rbs_motif=GGA/GAG/AGG;rbs_spacer=5-10bp;gc_cont=0.454 | | | | | | |
|  | PROJECT ID | ACCESSION ID | ORGANISMS | CLASS | PROTEIN FUNCTION | PROTEIN ID | %IDENTITY |
| **Matched Family** | [18747](http://www.ncbi.nlm.nih.gov/bioproject?db=bioproject&cmd=ShowDetailView&TermToSearch=18747) | [CP001113](http://www.ncbi.nlm.nih.gov/nuccore/CP001113) | Salmonella enterica subsp. enterica serovar Newport str. SL254, complete genome. | Gammaproteobacteria | DNA methylase | [ACF61188](http://www.ncbi.nlm.nih.gov/protein/ACF61188.1) | 100.0 |
|  | | | | | | | |
|  | | | | | | | |
| ------------------ | | | | | | | |
|  | | | | | | | |
|  | | | | | | | |
| **Input Sequence** | SM-Roxy-RH01221661_S26_L001_R1_001_(paired)_contig_47_46 # 42330 # 43211 # 1 # ID=47_46;partial=00;start_type=ATG;rbs_motif=AGGAG;rbs_spacer=5-10bp;gc_cont=0.520 | | | | | | |
|  | PROJECT ID | ACCESSION ID | ORGANISMS | CLASS | PROTEIN FUNCTION | PROTEIN ID | %IDENTITY |
| **Matched Family** | [30687](http://www.ncbi.nlm.nih.gov/bioproject?db=bioproject&cmd=ShowDetailView&TermToSearch=30687) | [AM933172](http://www.ncbi.nlm.nih.gov/nuccore/AM933172) | Salmonella enterica subsp. enterica serovar Enteritidis str. P125109 complete genome. | Gammaproteobacteria | putative beta-lactamase | [CAR35138](http://www.ncbi.nlm.nih.gov/protein/CAR35138.1) | 100.0 |
|  | | | | | | | |
|  | | | | | | | |
| ------------------ | | | | | | | |
|  | | | | | | | |
|  | | | | | | | |
| **Input Sequence** | SM-Roxy-RH01221661_S26_L001_R1_001_(paired)_contig_42_21 # 18858 # 19739 # 1 # ID=42_21;partial=00;start_type=ATG;rbs_motif=AGGA;rbs_spacer=5-10bp;gc_cont=0.497 | | | | | | |
|  | PROJECT ID | ACCESSION ID | ORGANISMS | CLASS | PROTEIN FUNCTION | PROTEIN ID | %IDENTITY |
| **Matched Family** | [18747](http://www.ncbi.nlm.nih.gov/bioproject?db=bioproject&cmd=ShowDetailView&TermToSearch=18747) | [CP001113](http://www.ncbi.nlm.nih.gov/nuccore/CP001113) | Salmonella enterica subsp. enterica serovar Newport str. SL254, complete genome. | Gammaproteobacteria | transcriptional regulator, RpiR family | [ACF64150](http://www.ncbi.nlm.nih.gov/protein/ACF64150.1) | 100.0 |
|  | | | | | | | |
|  | | | | | | | |
| ------------------ | | | | | | | |
|  | | | | | | | |
|  | | | | | | | |
| **Input Sequence** | SM-Roxy-RH01221661_S26_L001_R1_001_(paired)_contig_41_12 # 16688 # 17569 # -1 # ID=41_12;partial=00;start_type=ATG;rbs_motif=GGAG/GAGG;rbs_spacer=5-10bp;gc_cont=0.566 | | | | | | |
|  | PROJECT ID | ACCESSION ID | ORGANISMS | CLASS | PROTEIN FUNCTION | PROTEIN ID | %IDENTITY |
| **Matched Family** | [30687](http://www.ncbi.nlm.nih.gov/bioproject?db=bioproject&cmd=ShowDetailView&TermToSearch=30687) | [AM933172](http://www.ncbi.nlm.nih.gov/nuccore/AM933172) | Salmonella enterica subsp. enterica serovar Enteritidis str. P125109 complete genome. | Gammaproteobacteria | putative sugar kinase | [CAR35082](http://www.ncbi.nlm.nih.gov/protein/CAR35082.1) | 100.0 |
|  | | | | | | | |
|  | | | | | | | |
| ------------------ | | | | | | | |
|  | | | | | | | |
|  | | | | | | | |
| **Input Sequence** | SM-Roxy-RH01221661_S26_L001_R1_001_(paired)_contig_21_40 # 32090 # 32971 # -1 # ID=21_40;partial=00;start_type=ATG;rbs_motif=GGA/GAG/AGG;rbs_spacer=5-10bp;gc_cont=0.529 | | | | | | |
|  | PROJECT ID | ACCESSION ID | ORGANISMS | CLASS | PROTEIN FUNCTION | PROTEIN ID | %IDENTITY |
| **Matched Family** | [30687](http://www.ncbi.nlm.nih.gov/bioproject?db=bioproject&cmd=ShowDetailView&TermToSearch=30687) | [AM933172](http://www.ncbi.nlm.nih.gov/nuccore/AM933172) | Salmonella enterica subsp. enterica serovar Enteritidis str. P125109 complete genome. | Gammaproteobacteria | putative DNA methylase | [CAR33534](http://www.ncbi.nlm.nih.gov/protein/CAR33534.1) | 100.0 |
|  | | | | | | | |
|  | | | | | | | |
| ------------------ | | | | | | | |
|  | | | | | | | |
|  | | | | | | | |
| **Input Sequence** | SM-Roxy-RH01221661_S26_L001_R1_001_(paired)_contig_14_105 # 115974 # 116819 # -1 # ID=14_105;partial=00;start_type=ATG;rbs_motif=GGAGG;rbs_spacer=3-4bp;gc_cont=0.409 | | | | | | |
|  | PROJECT ID | ACCESSION ID | ORGANISMS | CLASS | PROTEIN FUNCTION | PROTEIN ID | %IDENTITY |
| **Matched Family** | [19467](http://www.ncbi.nlm.nih.gov/bioproject?db=bioproject&cmd=ShowDetailView&TermToSearch=19467) | [CP001144](http://www.ncbi.nlm.nih.gov/nuccore/CP001144) | Salmonella enterica subsp. enterica serovar Dublin str. C | Gammaproteobacteria | 2021853, complete genome. | [ACH76483](http://www.ncbi.nlm.nih.gov/protein/ACH76483.1) | 100.0 |
|  | | | | | | | |
|  | | | | | | | |
| ------------------ | | | | | | | |
|  | | | | | | | |
|  | | | | | | | |
| **Input Sequence** | SM-Roxy-RH01221661_S26_L001_R1_001_(paired)_contig_11_70 # 72443 # 73321 # -1 # ID=11_70;partial=00;start_type=ATG;rbs_motif=GGAG/GAGG;rbs_spacer=5-10bp;gc_cont=0.522 | | | | | | |
|  | PROJECT ID | ACCESSION ID | ORGANISMS | CLASS | PROTEIN FUNCTION | PROTEIN ID | %IDENTITY |
| **Matched Family** | [18747](http://www.ncbi.nlm.nih.gov/bioproject?db=bioproject&cmd=ShowDetailView&TermToSearch=18747) | [CP001113](http://www.ncbi.nlm.nih.gov/nuccore/CP001113) | Salmonella enterica subsp. enterica serovar Newport str. SL254, complete genome. | Gammaproteobacteria | StmR | [ACF63498](http://www.ncbi.nlm.nih.gov/protein/ACF63498.1) | 100.0 |
|  | | | | | | | |
|  | | | | | | | |
| ------------------ | | | | | | | |
|  | | | | | | | |
|  | | | | | | | |
| **Input Sequence** | SM-Roxy-RH01221661_S26_L001_R1_001_(paired)_contig_5_30 # 31596 # 32474 # -1 # ID=5_30;partial=00;start_type=ATG;rbs_motif=AGGA;rbs_spacer=5-10bp;gc_cont=0.545 | | | | | | |
|  | PROJECT ID | ACCESSION ID | ORGANISMS | CLASS | PROTEIN FUNCTION | PROTEIN ID | %IDENTITY |
| **Matched Family** | [19459](http://www.ncbi.nlm.nih.gov/bioproject?db=bioproject&cmd=ShowDetailView&TermToSearch=19459) | [CP001127](http://www.ncbi.nlm.nih.gov/nuccore/CP001127) | Salmonella enterica subsp. enterica serovar Schwarzengrund str. CVM19633, complete genome. | Gammaproteobacteria | deoxyribose-phosphate aldolase | [ACF91058](http://www.ncbi.nlm.nih.gov/protein/ACF91058.1) | 100.0 |
|  | | | | | | | |
|  | | | | | | | |
| ------------------ | | | | | | | |
|  | | | | | | | |
|  | | | | | | | |
| **Input Sequence** | SM-Roxy-RH01221661_S26_L001_R1_001_(paired)_contig_11_58 # 58958 # 59836 # 1 # ID=11_58;partial=00;start_type=ATG;rbs_motif=GGA/GAG/AGG;rbs_spacer=5-10bp;gc_cont=0.436 | | | | | | |
|  | PROJECT ID | ACCESSION ID | ORGANISMS | CLASS | PROTEIN FUNCTION | PROTEIN ID | %IDENTITY |
| **Matched Family** | [19467](http://www.ncbi.nlm.nih.gov/bioproject?db=bioproject&cmd=ShowDetailView&TermToSearch=19467) | [CP001144](http://www.ncbi.nlm.nih.gov/nuccore/CP001144) | Salmonella enterica subsp. enterica serovar Dublin str. C | Gammaproteobacteria | 2021853, complete genome. | [ACH74360](http://www.ncbi.nlm.nih.gov/protein/ACH74360.1) | 100.0 |
|  | | | | | | | |
|  | | | | | | | |
| ------------------ | | | | | | | |
|  | | | | | | | |
|  | | | | | | | |
| **Input Sequence** | SM-Roxy-RH01221661_S26_L001_R1_001_(paired)_contig_55_13 # 14730 # 15608 # -1 # ID=55_13;partial=00;start_type=ATG;rbs_motif=AGGA;rbs_spacer=5-10bp;gc_cont=0.520 | | | | | | |
|  | PROJECT ID | ACCESSION ID | ORGANISMS | CLASS | PROTEIN FUNCTION | PROTEIN ID | %IDENTITY |
| **Matched Family** | [13086](http://www.ncbi.nlm.nih.gov/bioproject?db=bioproject&cmd=ShowDetailView&TermToSearch=13086) | [CP000026](http://www.ncbi.nlm.nih.gov/nuccore/CP000026) | Salmonella enterica subsp. enterica serovar Paratyphi A str. ATCC 9150, complete genome. | Gammaproteobacteria | putative membrane protein | [AAV76338](http://www.ncbi.nlm.nih.gov/protein/AAV76338.1) | 100.0 |
|  | | | | | | | |
|  | | | | | | | |
| ------------------ | | | | | | | |
|  | | | | | | | |
|  | | | | | | | |
| **Input Sequence** | SM-Roxy-RH01221661_S26_L001_R1_001_(paired)_contig_5_18 # 18715 # 19593 # -1 # ID=5_18;partial=00;start_type=ATG;rbs_motif=AGGA/GGAG/GAGG;rbs_spacer=11-12bp;gc_cont=0.495 | | | | | | |
|  | PROJECT ID | ACCESSION ID | ORGANISMS | CLASS | PROTEIN FUNCTION | PROTEIN ID | %IDENTITY |
| **Matched Family** | [30687](http://www.ncbi.nlm.nih.gov/bioproject?db=bioproject&cmd=ShowDetailView&TermToSearch=30687) | [AM933172](http://www.ncbi.nlm.nih.gov/nuccore/AM933172) | Salmonella enterica subsp. enterica serovar Enteritidis str. P125109 complete genome. | Gammaproteobacteria | hypothetical protein | [CAR35373](http://www.ncbi.nlm.nih.gov/protein/CAR35373.1) | 100.0 |
|  | | | | | | | |
|  | | | | | | | |
| ------------------ | | | | | | | |
|  | | | | | | | |
|  | | | | | | | |
| **Input Sequence** | SM-Roxy-RH01221661_S26_L001_R1_001_(paired)_contig_42_134 # 144243 # 145040 # 1 # ID=42_134;partial=00;start_type=ATG;rbs_motif=3Base/5BMM;rbs_spacer=13-15bp;gc_cont=0.485 | | | | | | |
|  | PROJECT ID | ACCESSION ID | ORGANISMS | CLASS | PROTEIN FUNCTION | PROTEIN ID | %IDENTITY |
| **Matched Family** | [9618](http://www.ncbi.nlm.nih.gov/bioproject?db=bioproject&cmd=ShowDetailView&TermToSearch=9618) | [AE017220](http://www.ncbi.nlm.nih.gov/nuccore/AE017220) | Salmonella enterica subsp. enterica serovar Choleraesuis str. SC-B67, complete genome. | Gammaproteobacteria | pyruvate formate lyase activating enzyme 1 | [AAX64830](http://www.ncbi.nlm.nih.gov/protein/AAX64830.1) | 100.0 |
|  | | | | | | | |
|  | | | | | | | |
| ------------------ | | | | | | | |
|  | | | | | | | |
|  | | | | | | | |
| **Input Sequence** | SM-Roxy-RH01221661_S26_L001_R1_001_(paired)_contig_1_62 # 67135 # 67947 # -1 # ID=1_62;partial=00;start_type=ATG;rbs_motif=GGAG/GAGG;rbs_spacer=5-10bp;gc_cont=0.569 | | | | | | |
|  | PROJECT ID | ACCESSION ID | ORGANISMS | CLASS | PROTEIN FUNCTION | PROTEIN ID | %IDENTITY |
| **Matched Family** | [18747](http://www.ncbi.nlm.nih.gov/bioproject?db=bioproject&cmd=ShowDetailView&TermToSearch=18747) | [CP001113](http://www.ncbi.nlm.nih.gov/nuccore/CP001113) | Salmonella enterica subsp. enterica serovar Newport str. SL254, complete genome. | Gammaproteobacteria | dienelactone hydrolase | [ACF61956](http://www.ncbi.nlm.nih.gov/protein/ACF61956.1) | 100.0 |
|  | | | | | | | |
|  | | | | | | | |
| ------------------ | | | | | | | |
|  | | | | | | | |
|  | | | | | | | |
| **Input Sequence** | SM-Roxy-RH01221661_S26_L001_R1_001_(paired)_contig_30_16 # 14405 # 15280 # 1 # ID=30_16;partial=00;start_type=ATG;rbs_motif=AGxAGG/AGGxGG;rbs_spacer=3-4bp;gc_cont=0.596 | | | | | | |
|  | PROJECT ID | ACCESSION ID | ORGANISMS | CLASS | PROTEIN FUNCTION | PROTEIN ID | %IDENTITY |
| **Matched Family** | [18747](http://www.ncbi.nlm.nih.gov/bioproject?db=bioproject&cmd=ShowDetailView&TermToSearch=18747) | [CP001113](http://www.ncbi.nlm.nih.gov/nuccore/CP001113) | Salmonella enterica subsp. enterica serovar Newport str. SL254, complete genome. | Gammaproteobacteria | putative N-acetylmannosamine kinase | [ACF61505](http://www.ncbi.nlm.nih.gov/protein/ACF61505.1) | 100.0 |
|  | | | | | | | |
|  | | | | | | | |
| ------------------ | | | | | | | |
|  | | | | | | | |
|  | | | | | | | |
| **Input Sequence** | SM-Roxy-RH01221661_S26_L001_R1_001_(paired)_contig_5_89 # 86275 # 87150 # 1 # ID=5_89;partial=00;start_type=ATG;rbs_motif=GGAG/GAGG;rbs_spacer=5-10bp;gc_cont=0.471 | | | | | | |
|  | PROJECT ID | ACCESSION ID | ORGANISMS | CLASS | PROTEIN FUNCTION | PROTEIN ID | %IDENTITY |
| **Matched Family** | [13086](http://www.ncbi.nlm.nih.gov/bioproject?db=bioproject&cmd=ShowDetailView&TermToSearch=13086) | [CP000026](http://www.ncbi.nlm.nih.gov/nuccore/CP000026) | Salmonella enterica subsp. enterica serovar Paratyphi A str. ATCC 9150, complete genome. | Gammaproteobacteria | putative aldolase | [AAV79686](http://www.ncbi.nlm.nih.gov/protein/AAV79686.1) | 100.0 |
|  | | | | | | | |
|  | | | | | | | |
| ------------------ | | | | | | | |
|  | | | | | | | |
|  | | | | | | | |
| **Input Sequence** | SM-Roxy-RH01221661_S26_L001_R1_001_(paired)_contig_42_59 # 57710 # 58585 # 1 # ID=42_59;partial=00;start_type=ATG;rbs_motif=AGGA;rbs_spacer=5-10bp;gc_cont=0.397 | | | | | | |
|  | PROJECT ID | ACCESSION ID | ORGANISMS | CLASS | PROTEIN FUNCTION | PROTEIN ID | %IDENTITY |
| **Matched Family** | [30687](http://www.ncbi.nlm.nih.gov/bioproject?db=bioproject&cmd=ShowDetailView&TermToSearch=30687) | [AM933172](http://www.ncbi.nlm.nih.gov/nuccore/AM933172) | Salmonella enterica subsp. enterica serovar Enteritidis str. P125109 complete genome. | Gammaproteobacteria | pathogenicity island protein | [CAR32535](http://www.ncbi.nlm.nih.gov/protein/CAR32535.1) | 100.0 |
|  | | | | | | | |
|  | | | | | | | |
| ------------------ | | | | | | | |
|  | | | | | | | |
|  | | | | | | | |
| **Input Sequence** | SM-Roxy-RH01221661_S26_L001_R1_001_(paired)_contig_57_29 # 27277 # 28152 # 1 # ID=57_29;partial=00;start_type=ATG;rbs_motif=AGGA;rbs_spacer=5-10bp;gc_cont=0.347 | | | | | | |
|  | PROJECT ID | ACCESSION ID | ORGANISMS | CLASS | PROTEIN FUNCTION | PROTEIN ID | %IDENTITY |
| **Matched Family** | [18747](http://www.ncbi.nlm.nih.gov/bioproject?db=bioproject&cmd=ShowDetailView&TermToSearch=18747) | [CP001113](http://www.ncbi.nlm.nih.gov/nuccore/CP001113) | Salmonella enterica subsp. enterica serovar Newport str. SL254, complete genome. | Gammaproteobacteria | AraC family regulatory protein | [ACF61677](http://www.ncbi.nlm.nih.gov/protein/ACF61677.1) | 100.0 |
|  | | | | | | | |
|  | | | | | | | |
| ------------------ | | | | | | | |
|  | | | | | | | |
|  | | | | | | | |
| **Input Sequence** | SM-Roxy-RH01221661_S26_L001_R1_001_(paired)_contig_5_20 # 20833 # 21708 # -1 # ID=5_20;partial=00;start_type=ATG;rbs_motif=GGAG/GAGG;rbs_spacer=5-10bp;gc_cont=0.458 | | | | | | |
|  | PROJECT ID | ACCESSION ID | ORGANISMS | CLASS | PROTEIN FUNCTION | PROTEIN ID | %IDENTITY |
| **Matched Family** | [19467](http://www.ncbi.nlm.nih.gov/bioproject?db=bioproject&cmd=ShowDetailView&TermToSearch=19467) | [CP001144](http://www.ncbi.nlm.nih.gov/nuccore/CP001144) | Salmonella enterica subsp. enterica serovar Dublin str. C | Gammaproteobacteria | 2021853, complete genome. | [ACH76885](http://www.ncbi.nlm.nih.gov/protein/ACH76885.1) | 100.0 |
|  | | | | | | | |
|  | | | | | | | |
| ------------------ | | | | | | | |
|  | | | | | | | |
|  | | | | | | | |
| **Input Sequence** | SM-Roxy-RH01221661_S26_L001_R1_001_(paired)_contig_50_29 # 33498 # 34322 # 1 # ID=50_29;partial=00;start_type=ATG;rbs_motif=AGGAG;rbs_spacer=5-10bp;gc_cont=0.590 | | | | | | |
|  | PROJECT ID | ACCESSION ID | ORGANISMS | CLASS | PROTEIN FUNCTION | PROTEIN ID | %IDENTITY |
| **Matched Family** | [20993](http://www.ncbi.nlm.nih.gov/bioproject?db=bioproject&cmd=ShowDetailView&TermToSearch=20993) | [CP000857](http://www.ncbi.nlm.nih.gov/nuccore/CP000857) | Salmonella enterica subsp. enterica serovar Paratyphi C strain RKS4594, complete genome. | Gammaproteobacteria | acetate operon repressor | [ACN48310](http://www.ncbi.nlm.nih.gov/protein/ACN48310.1) | 100.0 |
|  | | | | | | | |
|  | | | | | | | |
| ------------------ | | | | | | | |
|  | | | | | | | |
|  | | | | | | | |
| **Input Sequence** | SM-Roxy-RH01221661_S26_L001_R1_001_(paired)_contig_44_82 # 83590 # 84459 # 1 # ID=44_82;partial=00;start_type=GTG;rbs_motif=GGA/GAG/AGG;rbs_spacer=5-10bp;gc_cont=0.462 | | | | | | |
|  | PROJECT ID | ACCESSION ID | ORGANISMS | CLASS | PROTEIN FUNCTION | PROTEIN ID | %IDENTITY |
| **Matched Family** | [19467](http://www.ncbi.nlm.nih.gov/bioproject?db=bioproject&cmd=ShowDetailView&TermToSearch=19467) | [CP001144](http://www.ncbi.nlm.nih.gov/nuccore/CP001144) | Salmonella enterica subsp. enterica serovar Dublin str. C | Gammaproteobacteria | 2021853, complete genome. | [ACH75691](http://www.ncbi.nlm.nih.gov/protein/ACH75691.1) | 100.0 |
|  | | | | | | | |
|  | | | | | | | |
| ------------------ | | | | | | | |
|  | | | | | | | |
|  | | | | | | | |
| **Input Sequence** | SM-Roxy-RH01221661_S26_L001_R1_001_(paired)_contig_21_208 # 201787 # 202656 # -1 # ID=21_208;partial=00;start_type=ATG;rbs_motif=AGGAG;rbs_spacer=5-10bp;gc_cont=0.616 | | | | | | |
|  | PROJECT ID | ACCESSION ID | ORGANISMS | CLASS | PROTEIN FUNCTION | PROTEIN ID | %IDENTITY |
| **Matched Family** | [30687](http://www.ncbi.nlm.nih.gov/bioproject?db=bioproject&cmd=ShowDetailView&TermToSearch=30687) | [AM933172](http://www.ncbi.nlm.nih.gov/nuccore/AM933172) | Salmonella enterica subsp. enterica serovar Enteritidis str. P125109 complete genome. | Gammaproteobacteria | DNA-3-methyladenine glycosidase II | [CAR33703](http://www.ncbi.nlm.nih.gov/protein/CAR33703.1) | 100.0 |
|  | | | | | | | |
|  | | | | | | | |
| ------------------ | | | | | | | |
|  | | | | | | | |
|  | | | | | | | |
| **Input Sequence** | SM-Roxy-RH01221661_S26_L001_R1_001_(paired)_contig_2_102 # 110198 # 111067 # -1 # ID=2_102;partial=00;start_type=ATG;rbs_motif=GGAG/GAGG;rbs_spacer=5-10bp;gc_cont=0.571 | | | | | | |
|  | PROJECT ID | ACCESSION ID | ORGANISMS | CLASS | PROTEIN FUNCTION | PROTEIN ID | %IDENTITY |
| **Matched Family** | [30687](http://www.ncbi.nlm.nih.gov/bioproject?db=bioproject&cmd=ShowDetailView&TermToSearch=30687) | [AM933172](http://www.ncbi.nlm.nih.gov/nuccore/AM933172) | Salmonella enterica subsp. enterica serovar Enteritidis str. P125109 complete genome. | Gammaproteobacteria | probable membrane component of 2-aminoethylphosphonate transporter | [CAR31995](http://www.ncbi.nlm.nih.gov/protein/CAR31995.1) | 100.0 |
|  | | | | | | | |
|  | | | | | | | |
| ------------------ | | | | | | | |
|  | | | | | | | |
|  | | | | | | | |
| **Input Sequence** | SM-Roxy-RH01221661_S26_L001_R1_001_(paired)_contig_11_15 # 12801 # 13667 # 1 # ID=11_15;partial=00;start_type=ATG;rbs_motif=AGGAG;rbs_spacer=5-10bp;gc_cont=0.577 | | | | | | |
|  | PROJECT ID | ACCESSION ID | ORGANISMS | CLASS | PROTEIN FUNCTION | PROTEIN ID | %IDENTITY |
| **Matched Family** | [19467](http://www.ncbi.nlm.nih.gov/bioproject?db=bioproject&cmd=ShowDetailView&TermToSearch=19467) | [CP001144](http://www.ncbi.nlm.nih.gov/nuccore/CP001144) | Salmonella enterica subsp. enterica serovar Dublin str. C | Gammaproteobacteria | 2021853, complete genome. | [ACH77668](http://www.ncbi.nlm.nih.gov/protein/ACH77668.1) | 100.0 |
|  | | | | | | | |
|  | | | | | | | |
| ------------------ | | | | | | | |
|  | | | | | | | |
|  | | | | | | | |
| **Input Sequence** | SM-Roxy-RH01221661_S26_L001_R1_001_(paired)_contig_3_12 # 10668 # 11534 # 1 # ID=3_12;partial=00;start_type=ATG;rbs_motif=GGAG/GAGG;rbs_spacer=5-10bp;gc_cont=0.571 | | | | | | |
|  | PROJECT ID | ACCESSION ID | ORGANISMS | CLASS | PROTEIN FUNCTION | PROTEIN ID | %IDENTITY |
| **Matched Family** | [20045](http://www.ncbi.nlm.nih.gov/bioproject?db=bioproject&cmd=ShowDetailView&TermToSearch=20045) | [CP001120](http://www.ncbi.nlm.nih.gov/nuccore/CP001120) | Salmonella enterica subsp. enterica serovar Heidelberg str. SL476, complete genome. | Gammaproteobacteria | pyridoxal kinase | [ACF70069](http://www.ncbi.nlm.nih.gov/protein/ACF70069.1) | 100.0 |
|  | | | | | | | |
|  | | | | | | | |
| ------------------ | | | | | | | |
|  | | | | | | | |
|  | | | | | | | |
| **Input Sequence** | SM-Roxy-RH01221661_S26_L001_R1_001_(paired)_contig_42_137 # 148470 # 149333 # -1 # ID=42_137;partial=00;start_type=ATG;rbs_motif=AGGAG;rbs_spacer=5-10bp;gc_cont=0.580 | | | | | | |
|  | PROJECT ID | ACCESSION ID | ORGANISMS | CLASS | PROTEIN FUNCTION | PROTEIN ID | %IDENTITY |
| **Matched Family** | [19467](http://www.ncbi.nlm.nih.gov/bioproject?db=bioproject&cmd=ShowDetailView&TermToSearch=19467) | [CP001144](http://www.ncbi.nlm.nih.gov/nuccore/CP001144) | Salmonella enterica subsp. enterica serovar Dublin str. C | Gammaproteobacteria | 2021853, complete genome. | [ACH77050](http://www.ncbi.nlm.nih.gov/protein/ACH77050.1) | 100.0 |
|  | | | | | | | |
|  | | | | | | | |
| ------------------ | | | | | | | |
|  | | | | | | | |
|  | | | | | | | |
| **Input Sequence** | SM-Roxy-RH01221661_S26_L001_R1_001_(paired)_contig_67_5 # 5724 # 6587 # 1 # ID=67_5;partial=00;start_type=ATG;rbs_motif=None;rbs_spacer=None;gc_cont=0.600 | | | | | | |
|  | PROJECT ID | ACCESSION ID | ORGANISMS | CLASS | PROTEIN FUNCTION | PROTEIN ID | %IDENTITY |
| **Matched Family** | [19467](http://www.ncbi.nlm.nih.gov/bioproject?db=bioproject&cmd=ShowDetailView&TermToSearch=19467) | [CP001144](http://www.ncbi.nlm.nih.gov/nuccore/CP001144) | Salmonella enterica subsp. enterica serovar Dublin str. C | Gammaproteobacteria | 2021853, complete genome. | [ACH77621](http://www.ncbi.nlm.nih.gov/protein/ACH77621.1) | 100.0 |
|  | | | | | | | |
|  | | | | | | | |
| ------------------ | | | | | | | |
|  | | | | | | | |
|  | | | | | | | |
| **Input Sequence** | SM-Roxy-RH01221661_S26_L001_R1_001_(paired)_contig_50_21 # 23246 # 24109 # 1 # ID=50_21;partial=00;start_type=ATG;rbs_motif=GGA/GAG/AGG;rbs_spacer=5-10bp;gc_cont=0.478 | | | | | | |
|  | PROJECT ID | ACCESSION ID | ORGANISMS | CLASS | PROTEIN FUNCTION | PROTEIN ID | %IDENTITY |
| **Matched Family** | [19467](http://www.ncbi.nlm.nih.gov/bioproject?db=bioproject&cmd=ShowDetailView&TermToSearch=19467) | [CP001144](http://www.ncbi.nlm.nih.gov/nuccore/CP001144) | Salmonella enterica subsp. enterica serovar Dublin str. C | Gammaproteobacteria | 2021853, complete genome. | [ACH77983](http://www.ncbi.nlm.nih.gov/protein/ACH77983.1) | 100.0 |
|  | | | | | | | |
|  | | | | | | | |
| ------------------ | | | | | | | |
|  | | | | | | | |
|  | | | | | | | |
| **Input Sequence** | SM-Roxy-RH01221661_S26_L001_R1_001_(paired)_contig_50_20 # 22390 # 23229 # 1 # ID=50_20;partial=00;start_type=ATG;rbs_motif=GGAG/GAGG;rbs_spacer=5-10bp;gc_cont=0.550 | | | | | | |
|  | PROJECT ID | ACCESSION ID | ORGANISMS | CLASS | PROTEIN FUNCTION | PROTEIN ID | %IDENTITY |
| **Matched Family** | [30687](http://www.ncbi.nlm.nih.gov/bioproject?db=bioproject&cmd=ShowDetailView&TermToSearch=30687) | [AM933172](http://www.ncbi.nlm.nih.gov/nuccore/AM933172) | Salmonella enterica subsp. enterica serovar Enteritidis str. P125109 complete genome. | Gammaproteobacteria | hypothetical protein | [CAR35548](http://www.ncbi.nlm.nih.gov/protein/CAR35548.1) | 100.0 |
|  | | | | | | | |
|  | | | | | | | |
| ------------------ | | | | | | | |
|  | | | | | | | |
|  | | | | | | | |
| **Input Sequence** | SM-Roxy-RH01221661_S26_L001_R1_001_(paired)_contig_11_331 # 347433 # 348293 # -1 # ID=11_331;partial=00;start_type=ATG;rbs_motif=GGAGG;rbs_spacer=5-10bp;gc_cont=0.549 | | | | | | |
|  | PROJECT ID | ACCESSION ID | ORGANISMS | CLASS | PROTEIN FUNCTION | PROTEIN ID | %IDENTITY |
| **Matched Family** | [18747](http://www.ncbi.nlm.nih.gov/bioproject?db=bioproject&cmd=ShowDetailView&TermToSearch=18747) | [CP001113](http://www.ncbi.nlm.nih.gov/nuccore/CP001113) | Salmonella enterica subsp. enterica serovar Newport str. SL254, complete genome. | Gammaproteobacteria | chelated iron transport system membrane protein YfeC | [ACF61682](http://www.ncbi.nlm.nih.gov/protein/ACF61682.1) | 100.0 |
|  | | | | | | | |
|  | | | | | | | |
| ------------------ | | | | | | | |
|  | | | | | | | |
|  | | | | | | | |
| **Input Sequence** | SM-Roxy-RH01221661_S26_L001_R1_001_(paired)_contig_15_22 # 21891 # 22748 # -1 # ID=15_22;partial=00;start_type=ATG;rbs_motif=AGGAG;rbs_spacer=5-10bp;gc_cont=0.538 | | | | | | |
|  | PROJECT ID | ACCESSION ID | ORGANISMS | CLASS | PROTEIN FUNCTION | PROTEIN ID | %IDENTITY |
| **Matched Family** | [19467](http://www.ncbi.nlm.nih.gov/bioproject?db=bioproject&cmd=ShowDetailView&TermToSearch=19467) | [CP001144](http://www.ncbi.nlm.nih.gov/nuccore/CP001144) | Salmonella enterica subsp. enterica serovar Dublin str. C | Gammaproteobacteria | 2021853, complete genome. | [ACH76155](http://www.ncbi.nlm.nih.gov/protein/ACH76155.1) | 100.0 |
|  | | | | | | | |
|  | | | | | | | |
| ------------------ | | | | | | | |
|  | | | | | | | |
|  | | | | | | | |
| **Input Sequence** | SM-Roxy-RH01221661_S26_L001_R1_001_(paired)_contig_21_38 # 30815 # 31675 # -1 # ID=21_38;partial=00;start_type=ATG;rbs_motif=GGAG/GAGG;rbs_spacer=5-10bp;gc_cont=0.513 | | | | | | |
|  | PROJECT ID | ACCESSION ID | ORGANISMS | CLASS | PROTEIN FUNCTION | PROTEIN ID | %IDENTITY |
| **Matched Family** | [30687](http://www.ncbi.nlm.nih.gov/bioproject?db=bioproject&cmd=ShowDetailView&TermToSearch=30687) | [AM933172](http://www.ncbi.nlm.nih.gov/nuccore/AM933172) | Salmonella enterica subsp. enterica serovar Enteritidis str. P125109 complete genome. | Gammaproteobacteria | phage protein | [CAR33532](http://www.ncbi.nlm.nih.gov/protein/CAR33532.1) | 100.0 |
|  | | | | | | | |
|  | | | | | | | |
| ------------------ | | | | | | | |
|  | | | | | | | |
|  | | | | | | | |
| **Input Sequence** | SM-Roxy-RH01221661_S26_L001_R1_001_(paired)_contig_38_16 # 15288 # 16106 # -1 # ID=38_16;partial=00;start_type=ATG;rbs_motif=GGA/GAG/AGG;rbs_spacer=5-10bp;gc_cont=0.535 | | | | | | |
|  | PROJECT ID | ACCESSION ID | ORGANISMS | CLASS | PROTEIN FUNCTION | PROTEIN ID | %IDENTITY |
| **Matched Family** | [19467](http://www.ncbi.nlm.nih.gov/bioproject?db=bioproject&cmd=ShowDetailView&TermToSearch=19467) | [CP001144](http://www.ncbi.nlm.nih.gov/nuccore/CP001144) | Salmonella enterica subsp. enterica serovar Dublin str. C | Gammaproteobacteria | 2021853, complete genome. | [ACH75879](http://www.ncbi.nlm.nih.gov/protein/ACH75879.1) | 100.0 |
|  | | | | | | | |
|  | | | | | | | |
| ------------------ | | | | | | | |
|  | | | | | | | |
|  | | | | | | | |
| **Input Sequence** | SM-Roxy-RH01221661_S26_L001_R1_001_(paired)_contig_72_1 # 223 # 1056 # -1 # ID=72_1;partial=00;start_type=ATG;rbs_motif=None;rbs_spacer=None;gc_cont=0.586 | | | | | | |
|  | PROJECT ID | ACCESSION ID | ORGANISMS | CLASS | PROTEIN FUNCTION | PROTEIN ID | %IDENTITY |
| **Matched Family** | [18747](http://www.ncbi.nlm.nih.gov/bioproject?db=bioproject&cmd=ShowDetailView&TermToSearch=18747) | [CP001113](http://www.ncbi.nlm.nih.gov/nuccore/CP001113) | Salmonella enterica subsp. enterica serovar Newport str. SL254, complete genome. | Gammaproteobacteria | integral membrane protein | [ACF64218](http://www.ncbi.nlm.nih.gov/protein/ACF64218.1) | 100.0 |
|  | | | | | | | |
|  | | | | | | | |
| ------------------ | | | | | | | |
|  | | | | | | | |
|  | | | | | | | |
| **Input Sequence** | SM-Roxy-RH01221661_S26_L001_R1_001_(paired)_contig_25_97 # 89508 # 90365 # -1 # ID=25_97;partial=00;start_type=ATG;rbs_motif=AGGAG;rbs_spacer=5-10bp;gc_cont=0.570 | | | | | | |
|  | PROJECT ID | ACCESSION ID | ORGANISMS | CLASS | PROTEIN FUNCTION | PROTEIN ID | %IDENTITY |
| **Matched Family** | [19467](http://www.ncbi.nlm.nih.gov/bioproject?db=bioproject&cmd=ShowDetailView&TermToSearch=19467) | [CP001144](http://www.ncbi.nlm.nih.gov/nuccore/CP001144) | Salmonella enterica subsp. enterica serovar Dublin str. C | Gammaproteobacteria | 2021853, complete genome. | [ACH75652](http://www.ncbi.nlm.nih.gov/protein/ACH75652.1) | 100.0 |
|  | | | | | | | |
|  | | | | | | | |
| ------------------ | | | | | | | |
|  | | | | | | | |
|  | | | | | | | |
| **Input Sequence** | SM-Roxy-RH01221661_S26_L001_R1_001_(paired)_contig_15_12 # 10490 # 11347 # -1 # ID=15_12;partial=00;start_type=ATG;rbs_motif=GGA/GAG/AGG;rbs_spacer=5-10bp;gc_cont=0.471 | | | | | | |
|  | PROJECT ID | ACCESSION ID | ORGANISMS | CLASS | PROTEIN FUNCTION | PROTEIN ID | %IDENTITY |
| **Matched Family** | [30687](http://www.ncbi.nlm.nih.gov/bioproject?db=bioproject&cmd=ShowDetailView&TermToSearch=30687) | [AM933172](http://www.ncbi.nlm.nih.gov/nuccore/AM933172) | Salmonella enterica subsp. enterica serovar Enteritidis str. P125109 complete genome. | Gammaproteobacteria | putative transcriptional regulator (MarT) | [CAR35159](http://www.ncbi.nlm.nih.gov/protein/CAR35159.1) | 100.0 |
|  | | | | | | | |
|  | | | | | | | |
| ------------------ | | | | | | | |
|  | | | | | | | |
|  | | | | | | | |
| **Input Sequence** | SM-Roxy-RH01221661_S26_L001_R1_001_(paired)_contig_57_15 # 12301 # 13158 # 1 # ID=57_15;partial=00;start_type=ATG;rbs_motif=GGAG/GAGG;rbs_spacer=5-10bp;gc_cont=0.445 | | | | | | |
|  | PROJECT ID | ACCESSION ID | ORGANISMS | CLASS | PROTEIN FUNCTION | PROTEIN ID | %IDENTITY |
| **Matched Family** | [30687](http://www.ncbi.nlm.nih.gov/bioproject?db=bioproject&cmd=ShowDetailView&TermToSearch=30687) | [AM933172](http://www.ncbi.nlm.nih.gov/nuccore/AM933172) | Salmonella enterica subsp. enterica serovar Enteritidis str. P125109 complete genome. | Gammaproteobacteria | hypothetical protein | [CAR35662](http://www.ncbi.nlm.nih.gov/protein/CAR35662.1) | 100.0 |
|  | | | | | | | |
|  | | | | | | | |
| ------------------ | | | | | | | |
|  | | | | | | | |
|  | | | | | | | |
| **Input Sequence** | SM-Roxy-RH01221661_S26_L001_R1_001_(paired)_contig_44_87 # 88011 # 88862 # -1 # ID=44_87;partial=00;start_type=ATG;rbs_motif=GGAG/GAGG;rbs_spacer=5-10bp;gc_cont=0.387 | | | | | | |
|  | PROJECT ID | ACCESSION ID | ORGANISMS | CLASS | PROTEIN FUNCTION | PROTEIN ID | %IDENTITY |
| **Matched Family** | [20993](http://www.ncbi.nlm.nih.gov/bioproject?db=bioproject&cmd=ShowDetailView&TermToSearch=20993) | [CP000857](http://www.ncbi.nlm.nih.gov/nuccore/CP000857) | Salmonella enterica subsp. enterica serovar Paratyphi C strain RKS4594, complete genome. | Gammaproteobacteria | putative transcriptional regulator | [ACN46194](http://www.ncbi.nlm.nih.gov/protein/ACN46194.1) | 100.0 |
|  | | | | | | | |
|  | | | | | | | |
| ------------------ | | | | | | | |
|  | | | | | | | |
|  | | | | | | | |
| **Input Sequence** | SM-Roxy-RH01221661_S26_L001_R1_001_(paired)_contig_42_22 # 19845 # 20633 # -1 # ID=42_22;partial=00;start_type=ATG;rbs_motif=GGA/GAG/AGG;rbs_spacer=5-10bp;gc_cont=0.507 | | | | | | |
|  | PROJECT ID | ACCESSION ID | ORGANISMS | CLASS | PROTEIN FUNCTION | PROTEIN ID | %IDENTITY |
| **Matched Family** | [30687](http://www.ncbi.nlm.nih.gov/bioproject?db=bioproject&cmd=ShowDetailView&TermToSearch=30687) | [AM933172](http://www.ncbi.nlm.nih.gov/nuccore/AM933172) | Salmonella enterica subsp. enterica serovar Enteritidis str. P125109 complete genome. | Gammaproteobacteria | PhoH protein (phosphate starvation-inducible protein PsiH) | [CAR32571](http://www.ncbi.nlm.nih.gov/protein/CAR32571.1) | 100.0 |
|  | | | | | | | |
|  | | | | | | | |
| ------------------ | | | | | | | |
|  | | | | | | | |
|  | | | | | | | |
| **Input Sequence** | SM-Roxy-RH01221661_S26_L001_R1_001_(paired)_contig_24_20 # 23229 # 24083 # 1 # ID=24_20;partial=00;start_type=ATG;rbs_motif=GGAG/GAGG;rbs_spacer=5-10bp;gc_cont=0.588 | | | | | | |
|  | PROJECT ID | ACCESSION ID | ORGANISMS | CLASS | PROTEIN FUNCTION | PROTEIN ID | %IDENTITY |
| **Matched Family** | [30687](http://www.ncbi.nlm.nih.gov/bioproject?db=bioproject&cmd=ShowDetailView&TermToSearch=30687) | [AM933172](http://www.ncbi.nlm.nih.gov/nuccore/AM933172) | Salmonella enterica subsp. enterica serovar Enteritidis str. P125109 complete genome. | Gammaproteobacteria | hypothetical araC-family transcriptional regulator | [CAR32127](http://www.ncbi.nlm.nih.gov/protein/CAR32127.1) | 100.0 |
|  | | | | | | | |
|  | | | | | | | |
| ------------------ | | | | | | | |
|  | | | | | | | |
|  | | | | | | | |
| **Input Sequence** | SM-Roxy-RH01221661_S26_L001_R1_001_(paired)_contig_13_38 # 37806 # 38660 # 1 # ID=13_38;partial=00;start_type=ATG;rbs_motif=AGGAG;rbs_spacer=5-10bp;gc_cont=0.575 | | | | | | |
|  | PROJECT ID | ACCESSION ID | ORGANISMS | CLASS | PROTEIN FUNCTION | PROTEIN ID | %IDENTITY |
| **Matched Family** | [19459](http://www.ncbi.nlm.nih.gov/bioproject?db=bioproject&cmd=ShowDetailView&TermToSearch=19459) | [CP001127](http://www.ncbi.nlm.nih.gov/nuccore/CP001127) | Salmonella enterica subsp. enterica serovar Schwarzengrund str. CVM19633, complete genome. | Gammaproteobacteria | L-ribulose-5-phosphate 3-epimerase | [ACF89614](http://www.ncbi.nlm.nih.gov/protein/ACF89614.1) | 100.0 |
|  | | | | | | | |
|  | | | | | | | |
| ------------------ | | | | | | | |
|  | | | | | | | |
|  | | | | | | | |
| **Input Sequence** | SM-Roxy-RH01221661_S26_L001_R1_001_(paired)_contig_12_200 # 193262 # 194116 # 1 # ID=12_200;partial=00;start_type=GTG;rbs_motif=AGGA;rbs_spacer=5-10bp;gc_cont=0.512 | | | | | | |
|  | PROJECT ID | ACCESSION ID | ORGANISMS | CLASS | PROTEIN FUNCTION | PROTEIN ID | %IDENTITY |
| **Matched Family** | [30687](http://www.ncbi.nlm.nih.gov/bioproject?db=bioproject&cmd=ShowDetailView&TermToSearch=30687) | [AM933172](http://www.ncbi.nlm.nih.gov/nuccore/AM933172) | Salmonella enterica subsp. enterica serovar Enteritidis str. P125109 complete genome. | Gammaproteobacteria | Putative DNA/RNA non-specific endonuclease | [CAR33295](http://www.ncbi.nlm.nih.gov/protein/CAR33295.1) | 100.0 |
|  | | | | | | | |
|  | | | | | | | |
| ------------------ | | | | | | | |
|  | | | | | | | |
|  | | | | | | | |
| **Input Sequence** | SM-Roxy-RH01221661_S26_L001_R1_001_(paired)_contig_42_46 # 43209 # 44060 # -1 # ID=42_46;partial=00;start_type=ATG;rbs_motif=GGAG/GAGG;rbs_spacer=5-10bp;gc_cont=0.538 | | | | | | |
|  | PROJECT ID | ACCESSION ID | ORGANISMS | CLASS | PROTEIN FUNCTION | PROTEIN ID | %IDENTITY |
| **Matched Family** | [13086](http://www.ncbi.nlm.nih.gov/bioproject?db=bioproject&cmd=ShowDetailView&TermToSearch=13086) | [CP000026](http://www.ncbi.nlm.nih.gov/nuccore/CP000026) | Salmonella enterica subsp. enterica serovar Paratyphi A str. ATCC 9150, complete genome. | Gammaproteobacteria | 3,4-dihydroxyphenylacetate 2,3-dioxygenase | [AAV77667](http://www.ncbi.nlm.nih.gov/protein/AAV77667.1) | 100.0 |
|  | | | | | | | |
|  | | | | | | | |
| ------------------ | | | | | | | |
|  | | | | | | | |
|  | | | | | | | |
| **Input Sequence** | SM-Roxy-RH01221661_S26_L001_R1_001_(paired)_contig_60_19 # 23137 # 23988 # -1 # ID=60_19;partial=00;start_type=ATG;rbs_motif=GGA/GAG/AGG;rbs_spacer=5-10bp;gc_cont=0.563 | | | | | | |
|  | PROJECT ID | ACCESSION ID | ORGANISMS | CLASS | PROTEIN FUNCTION | PROTEIN ID | %IDENTITY |
| **Matched Family** | [19467](http://www.ncbi.nlm.nih.gov/bioproject?db=bioproject&cmd=ShowDetailView&TermToSearch=19467) | [CP001144](http://www.ncbi.nlm.nih.gov/nuccore/CP001144) | Salmonella enterica subsp. enterica serovar Dublin str. C | Gammaproteobacteria | 2021853, complete genome. | [ACH74867](http://www.ncbi.nlm.nih.gov/protein/ACH74867.1) | 100.0 |
|  | | | | | | | |
|  | | | | | | | |
| ------------------ | | | | | | | |
|  | | | | | | | |
|  | | | | | | | |
| **Input Sequence** | SM-Roxy-RH01221661_S26_L001_R1_001_(paired)_contig_22_33 # 37080 # 37928 # 1 # ID=22_33;partial=00;start_type=ATG;rbs_motif=AGGAG;rbs_spacer=5-10bp;gc_cont=0.591 | | | | | | |
|  | PROJECT ID | ACCESSION ID | ORGANISMS | CLASS | PROTEIN FUNCTION | PROTEIN ID | %IDENTITY |
| **Matched Family** | [19467](http://www.ncbi.nlm.nih.gov/bioproject?db=bioproject&cmd=ShowDetailView&TermToSearch=19467) | [CP001144](http://www.ncbi.nlm.nih.gov/nuccore/CP001144) | Salmonella enterica subsp. enterica serovar Dublin str. C | Gammaproteobacteria | 2021853, complete genome. | [ACH74048](http://www.ncbi.nlm.nih.gov/protein/ACH74048.1) | 100.0 |
|  | | | | | | | |
|  | | | | | | | |
| ------------------ | | | | | | | |
|  | | | | | | | |
|  | | | | | | | |
| **Input Sequence** | SM-Roxy-RH01221661_S26_L001_R1_001_(paired)_contig_23_38 # 40284 # 41132 # 1 # ID=23_38;partial=00;start_type=ATG;rbs_motif=AGGAG;rbs_spacer=5-10bp;gc_cont=0.492 | | | | | | |
|  | PROJECT ID | ACCESSION ID | ORGANISMS | CLASS | PROTEIN FUNCTION | PROTEIN ID | %IDENTITY |
| **Matched Family** | [18747](http://www.ncbi.nlm.nih.gov/bioproject?db=bioproject&cmd=ShowDetailView&TermToSearch=18747) | [CP001113](http://www.ncbi.nlm.nih.gov/nuccore/CP001113) | Salmonella enterica subsp. enterica serovar Newport str. SL254, complete genome. | Gammaproteobacteria | fructose permease iid component | [ACF63440](http://www.ncbi.nlm.nih.gov/protein/ACF63440.1) | 100.0 |
|  | | | | | | | |
|  | | | | | | | |
| ------------------ | | | | | | | |
|  | | | | | | | |
|  | | | | | | | |
| **Input Sequence** | SM-Roxy-RH01221661_S26_L001_R1_001_(paired)_contig_12_128 # 123559 # 124407 # 1 # ID=12_128;partial=00;start_type=ATG;rbs_motif=GGAG/GAGG;rbs_spacer=5-10bp;gc_cont=0.464 | | | | | | |
|  | PROJECT ID | ACCESSION ID | ORGANISMS | CLASS | PROTEIN FUNCTION | PROTEIN ID | %IDENTITY |
| **Matched Family** | [13086](http://www.ncbi.nlm.nih.gov/bioproject?db=bioproject&cmd=ShowDetailView&TermToSearch=13086) | [CP000026](http://www.ncbi.nlm.nih.gov/nuccore/CP000026) | Salmonella enterica subsp. enterica serovar Paratyphi A str. ATCC 9150, complete genome. | Gammaproteobacteria | putative transcriptional regulator | [AAV77511](http://www.ncbi.nlm.nih.gov/protein/AAV77511.1) | 100.0 |
|  | | | | | | | |
|  | | | | | | | |
| ------------------ | | | | | | | |
|  | | | | | | | |
|  | | | | | | | |
| **Input Sequence** | SM-Roxy-RH01221661_S26_L001_R1_001_(paired)_contig_5_22 # 22739 # 23587 # -1 # ID=5_22;partial=00;start_type=ATG;rbs_motif=AGxAGG/AGGxGG;rbs_spacer=5-10bp;gc_cont=0.483 | | | | | | |
|  | PROJECT ID | ACCESSION ID | ORGANISMS | CLASS | PROTEIN FUNCTION | PROTEIN ID | %IDENTITY |
| **Matched Family** | [19467](http://www.ncbi.nlm.nih.gov/bioproject?db=bioproject&cmd=ShowDetailView&TermToSearch=19467) | [CP001144](http://www.ncbi.nlm.nih.gov/nuccore/CP001144) | Salmonella enterica subsp. enterica serovar Dublin str. C | Gammaproteobacteria | 2021853, complete genome. | [ACH73819](http://www.ncbi.nlm.nih.gov/protein/ACH73819.1) | 100.0 |
|  | | | | | | | |
|  | | | | | | | |
| ------------------ | | | | | | | |
|  | | | | | | | |
|  | | | | | | | |
| **Input Sequence** | SM-Roxy-RH01221661_S26_L001_R1_001_(paired)_contig_73_2 # 545 # 1390 # 1 # ID=73_2;partial=00;start_type=ATG;rbs_motif=None;rbs_spacer=None;gc_cont=0.599 | | | | | | |
|  | PROJECT ID | ACCESSION ID | ORGANISMS | CLASS | PROTEIN FUNCTION | PROTEIN ID | %IDENTITY |
| **Matched Family** | [30687](http://www.ncbi.nlm.nih.gov/bioproject?db=bioproject&cmd=ShowDetailView&TermToSearch=30687) | [AM933172](http://www.ncbi.nlm.nih.gov/nuccore/AM933172) | Salmonella enterica subsp. enterica serovar Enteritidis str. P125109 complete genome. | Gammaproteobacteria | putative outer membrane protein | [CAR35809](http://www.ncbi.nlm.nih.gov/protein/CAR35809.1) | 100.0 |
|  | | | | | | | |
|  | | | | | | | |
| ------------------ | | | | | | | |
|  | | | | | | | |
|  | | | | | | | |
| **Input Sequence** | SM-Roxy-RH01221661_S26_L001_R1_001_(paired)_contig_44_98 # 98135 # 98887 # -1 # ID=44_98;partial=00;start_type=ATG;rbs_motif=GGA/GAG/AGG;rbs_spacer=5-10bp;gc_cont=0.502 | | | | | | |
|  | PROJECT ID | ACCESSION ID | ORGANISMS | CLASS | PROTEIN FUNCTION | PROTEIN ID | %IDENTITY |
| **Matched Family** | [9618](http://www.ncbi.nlm.nih.gov/bioproject?db=bioproject&cmd=ShowDetailView&TermToSearch=9618) | [AE017220](http://www.ncbi.nlm.nih.gov/nuccore/AE017220) | Salmonella enterica subsp. enterica serovar Choleraesuis str. SC-B67, complete genome. | Gammaproteobacteria | transcriptional regulation of aerobic, anaerobic respiration, osmotic balance (CRP family) | [AAX65562](http://www.ncbi.nlm.nih.gov/protein/AAX65562.1) | 100.0 |
|  | | | | | | | |
|  | | | | | | | |
| ------------------ | | | | | | | |
|  | | | | | | | |
|  | | | | | | | |
| **Input Sequence** | SM-Roxy-RH01221661_S26_L001_R1_001_(paired)_contig_35_10 # 10096 # 10941 # 1 # ID=35_10;partial=00;start_type=ATG;rbs_motif=GGA/GAG/AGG;rbs_spacer=5-10bp;gc_cont=0.551 | | | | | | |
|  | PROJECT ID | ACCESSION ID | ORGANISMS | CLASS | PROTEIN FUNCTION | PROTEIN ID | %IDENTITY |
| **Matched Family** | [241](http://www.ncbi.nlm.nih.gov/bioproject?db=bioproject&cmd=ShowDetailView&TermToSearch=241) | [AE006468](http://www.ncbi.nlm.nih.gov/nuccore/AE006468) | Salmonella enterica subsp. enterica serovar Typhimurium str. LT2, complete genome. | Gammaproteobacteria | putative thiol-disulfide isomerase | [AAL18992](http://www.ncbi.nlm.nih.gov/protein/AAL18992.1) | 100.0 |
|  | | | | | | | |
|  | | | | | | | |
| ------------------ | | | | | | | |
|  | | | | | | | |
|  | | | | | | | |
| **Input Sequence** | SM-Roxy-RH01221661_S26_L001_R1_001_(paired)_contig_4_52 # 64365 # 65210 # 1 # ID=4_52;partial=00;start_type=ATG;rbs_motif=AGGA;rbs_spacer=5-10bp;gc_cont=0.472 | | | | | | |
|  | PROJECT ID | ACCESSION ID | ORGANISMS | CLASS | PROTEIN FUNCTION | PROTEIN ID | %IDENTITY |
| **Matched Family** | [19467](http://www.ncbi.nlm.nih.gov/bioproject?db=bioproject&cmd=ShowDetailView&TermToSearch=19467) | [CP001144](http://www.ncbi.nlm.nih.gov/nuccore/CP001144) | Salmonella enterica subsp. enterica serovar Dublin str. C | Gammaproteobacteria | 2021853, complete genome. | [ACH77996](http://www.ncbi.nlm.nih.gov/protein/ACH77996.1) | 100.0 |
|  | | | | | | | |
|  | | | | | | | |
| ------------------ | | | | | | | |
|  | | | | | | | |
|  | | | | | | | |
| **Input Sequence** | SM-Roxy-RH01221661_S26_L001_R1_001_(paired)_contig_44_88 # 89249 # 90007 # 1 # ID=44_88;partial=00;start_type=ATG;rbs_motif=GGA/GAG/AGG;rbs_spacer=5-10bp;gc_cont=0.489 | | | | | | |
|  | PROJECT ID | ACCESSION ID | ORGANISMS | CLASS | PROTEIN FUNCTION | PROTEIN ID | %IDENTITY |
| **Matched Family** | [19467](http://www.ncbi.nlm.nih.gov/bioproject?db=bioproject&cmd=ShowDetailView&TermToSearch=19467) | [CP001144](http://www.ncbi.nlm.nih.gov/nuccore/CP001144) | Salmonella enterica subsp. enterica serovar Dublin str. C | Gammaproteobacteria | 2021853, complete genome. | [ACH75973](http://www.ncbi.nlm.nih.gov/protein/ACH75973.1) | 100.0 |
|  | | | | | | | |
|  | | | | | | | |
| ------------------ | | | | | | | |
|  | | | | | | | |
|  | | | | | | | |
| **Input Sequence** | SM-Roxy-RH01221661_S26_L001_R1_001_(paired)_contig_11_71 # 73413 # 74255 # 1 # ID=11_71;partial=00;start_type=ATG;rbs_motif=AGGAG;rbs_spacer=5-10bp;gc_cont=0.495 | | | | | | |
|  | PROJECT ID | ACCESSION ID | ORGANISMS | CLASS | PROTEIN FUNCTION | PROTEIN ID | %IDENTITY |
| **Matched Family** | [18747](http://www.ncbi.nlm.nih.gov/bioproject?db=bioproject&cmd=ShowDetailView&TermToSearch=18747) | [CP001113](http://www.ncbi.nlm.nih.gov/nuccore/CP001113) | Salmonella enterica subsp. enterica serovar Newport str. SL254, complete genome. | Gammaproteobacteria | HpcH/HpaI aldolase/citrate lyase family protein | [ACF65136](http://www.ncbi.nlm.nih.gov/protein/ACF65136.1) | 100.0 |
|  | | | | | | | |
|  | | | | | | | |
| ------------------ | | | | | | | |
|  | | | | | | | |
|  | | | | | | | |
| **Input Sequence** | SM-Roxy-RH01221661_S26_L001_R1_001_(paired)_contig_12_185 # 178712 # 179551 # 1 # ID=12_185;partial=00;start_type=ATG;rbs_motif=AGxAGG/AGGxGG;rbs_spacer=5-10bp;gc_cont=0.433 | | | | | | |
|  | PROJECT ID | ACCESSION ID | ORGANISMS | CLASS | PROTEIN FUNCTION | PROTEIN ID | %IDENTITY |
| **Matched Family** | [18747](http://www.ncbi.nlm.nih.gov/bioproject?db=bioproject&cmd=ShowDetailView&TermToSearch=18747) | [CP001113](http://www.ncbi.nlm.nih.gov/nuccore/CP001113) | Salmonella enterica subsp. enterica serovar Newport str. SL254, complete genome. | Gammaproteobacteria | HTH-type transcriptional regulator ChbR | [ACF64108](http://www.ncbi.nlm.nih.gov/protein/ACF64108.1) | 100.0 |
|  | | | | | | | |
|  | | | | | | | |
| ------------------ | | | | | | | |
|  | | | | | | | |
|  | | | | | | | |
| **Input Sequence** | SM-Roxy-RH01221661_S26_L001_R1_001_(paired)_contig_55_29 # 27174 # 28016 # -1 # ID=55_29;partial=00;start_type=ATG;rbs_motif=GGAG/GAGG;rbs_spacer=5-10bp;gc_cont=0.595 | | | | | | |
|  | PROJECT ID | ACCESSION ID | ORGANISMS | CLASS | PROTEIN FUNCTION | PROTEIN ID | %IDENTITY |
| **Matched Family** | [19467](http://www.ncbi.nlm.nih.gov/bioproject?db=bioproject&cmd=ShowDetailView&TermToSearch=19467) | [CP001144](http://www.ncbi.nlm.nih.gov/nuccore/CP001144) | Salmonella enterica subsp. enterica serovar Dublin str. C | Gammaproteobacteria | 2021853, complete genome. | [ACH77121](http://www.ncbi.nlm.nih.gov/protein/ACH77121.1) | 100.0 |
|  | | | | | | | |
|  | | | | | | | |
| ------------------ | | | | | | | |
|  | | | | | | | |
|  | | | | | | | |
| **Input Sequence** | SM-Roxy-RH01221661_S26_L001_R1_001_(paired)_contig_21_173 # 160380 # 161204 # -1 # ID=21_173;partial=00;start_type=ATG;rbs_motif=None;rbs_spacer=None;gc_cont=0.333 | | | | | | |
|  | PROJECT ID | ACCESSION ID | ORGANISMS | CLASS | PROTEIN FUNCTION | PROTEIN ID | %IDENTITY |
| **Matched Family** | [19467](http://www.ncbi.nlm.nih.gov/bioproject?db=bioproject&cmd=ShowDetailView&TermToSearch=19467) | [CP001144](http://www.ncbi.nlm.nih.gov/nuccore/CP001144) | Salmonella enterica subsp. enterica serovar Dublin str. C | Gammaproteobacteria | 2021853, complete genome. | [ACH73791](http://www.ncbi.nlm.nih.gov/protein/ACH73791.1) | 100.0 |
|  | | | | | | | |
|  | | | | | | | |
| ------------------ | | | | | | | |
|  | | | | | | | |
|  | | | | | | | |
| **Input Sequence** | SM-Roxy-RH01221661_S26_L001_R1_001_(paired)_contig_44_15 # 17695 # 18531 # 1 # ID=44_15;partial=00;start_type=GTG;rbs_motif=GGAG/GAGG;rbs_spacer=5-10bp;gc_cont=0.481 | | | | | | |
|  | PROJECT ID | ACCESSION ID | ORGANISMS | CLASS | PROTEIN FUNCTION | PROTEIN ID | %IDENTITY |
| **Matched Family** | [13086](http://www.ncbi.nlm.nih.gov/bioproject?db=bioproject&cmd=ShowDetailView&TermToSearch=13086) | [CP000026](http://www.ncbi.nlm.nih.gov/nuccore/CP000026) | Salmonella enterica subsp. enterica serovar Paratyphi A str. ATCC 9150, complete genome. | Gammaproteobacteria | possible membrane transport protein | [AAV77097](http://www.ncbi.nlm.nih.gov/protein/AAV77097.1) | 100.0 |
|  | | | | | | | |
|  | | | | | | | |
| ------------------ | | | | | | | |
|  | | | | | | | |
|  | | | | | | | |
| **Input Sequence** | SM-Roxy-RH01221661_S26_L001_R1_001_(paired)_contig_53_74 # 86849 # 87679 # 1 # ID=53_74;partial=00;start_type=ATG;rbs_motif=GGA/GAG/AGG;rbs_spacer=5-10bp;gc_cont=0.525 | | | | | | |
|  | PROJECT ID | ACCESSION ID | ORGANISMS | CLASS | PROTEIN FUNCTION | PROTEIN ID | %IDENTITY |
[truncated: 550,186 more chars]
